# Inverting polarity in a cobalt MHAT reaction *via* reductive catalytic turnover

**DOI:** 10.1039/d5sc06119a

**Published:** 2025-11-10

**Authors:** Samikshan Jana, Daniel A. Kusza, Nikita Vystavkin, Danijela Lunic, Christopher J. Teskey

**Affiliations:** a Institute of Organic Chemistry, Technische Universität Braunschweig Hagenring 30 38106 Braunschweig Germany christopher.teskey@tu-braunschweig.de

## Abstract

Metal hydride hydrogen-atom-transfer (MHAT) catalysis proceeds *via* selective hydrogen atom transfer to olefins and is a key strategy in modern organic synthesis. The resulting carbon-centred radicals can engage in classical one-electron, radical transformations. Alternatively, following cage-collapse with the metalloradical and oxidation, an electrophilic alkyl-metal intermediate is formed which reacts as a carbocation equivalent. While this oxidative pathway is well-established, strategies to reverse the inherent polarity of the olefin, such that the internal carbon atom is nucleophilic, remain unexplored. The scarcity of this approach arises from challenges in integrating the classic oxidative MHAT catalytic cycle with single electron reduction of a radical reaction intermediate. Here we demonstrate that a reductive MHAT cycle enables the merger of this field for the first time with reductive radical–polar–crossover. This yields a reaction platform with inverted polarity unlocking the possibility of hydrofunctionalization with electrophiles. We have applied this approach to Markovnikov-selective hydrocarboxylation, using atmospheric carbon dioxide. Notably, this mild method proceeds *via* a mechanistically distinct pathway when compared to other metals such as nickel and can be applied in the synthesis and late-stage-functionalisation of drug-like molecules.

## Abstract 2

A reductive MHAT cycle enables radical-polar crossover to an anionic intermediate, inverting olefin polarity and allowing Markovnikov-selective hydrocarboxylation with CO_2_ under mild conditions.

## Graphical abstract

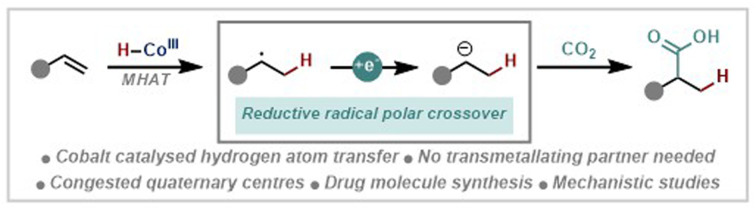

## Introduction

**Scheme 1:**
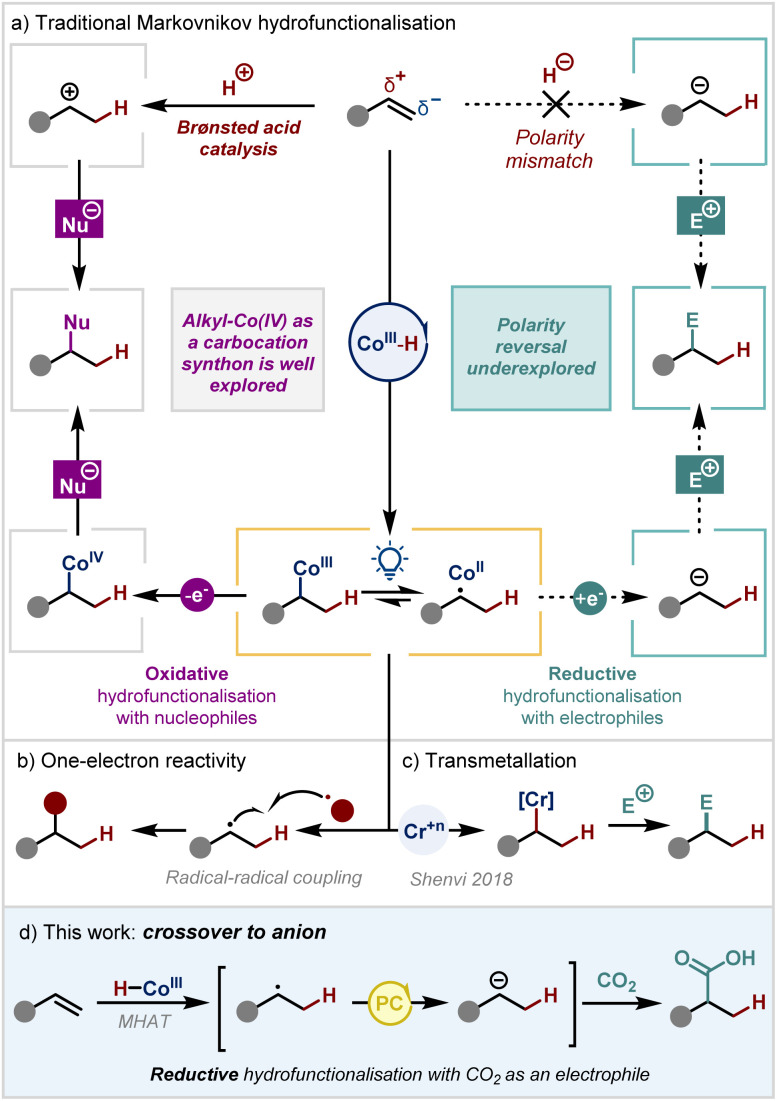
Markovnikov hydrofunctionalisation

Olefins are indispensable feedstocks that serve as crucial synthetic building blocks for a wide range of applications, from fine chemical synthesis to large-scale industrial reactions. Over the past few decades, powerful tools for transforming olefins have been developed, including the Heck reaction,^1^ Grubbs' metathesis^2^ and Diels–Alder cycloadditions,^3^ among others. Nevertheless, the classical Markovnikov addition^4^ remains at the core of every undergraduate chemistry programme. In this process, an acid reacts with an asymmetrically substituted alkene, with the proton attaching to the least substituted carbon to yield the more stable cation at the internal carbon of the alkene.^5^ This traditional pattern of olefin reactivity imparts electrophilic character to the internal position, making it susceptible to attack by nucleophiles. Contemporary research continues to develop the scope of Brønsted acid catalysis to exploit this Markovnikov reactivity (Scheme 1a, left).^6^

In recent years, metal hydride hydrogen-atom-transfer (MHAT) catalysis has become a cornerstone of modern organic synthesis.^7^ This approach allows a hydrogen atom to be selectively transferred to an olefin from a high-valent metal hydride (most commonly cobalt, manganese, or iron), forming a more stabilised carbon-centred radical.^8^ This process is analogous to Brønsted acid catalysis in terms of regiochemistry, but uses one-electron logic (Scheme 1b). The radical intermediate can then react in a classical manner – either through reaction with a radicalophilic π-system^9–14^ or through selective coupling with a persistent radical generated under the reaction conditions.^15–19^

Alternatively, the radical may undergo cage-collapse with the metalloradical to form an alkyl-metal(iii) intermediate, which is subsequently oxidised to the alkyl-metal(iv) species.^20^ This renders the carbon atom attached to the metal electrophilic, with the metal acting as a pseudo-leaving group. Such oxidative radical–polar–crossover reactions have enabled a range of different nucleophiles to be used as coupling partners, with the additional benefit of catalyst control compared to Brønsted acid catalysis.^21–39^ However, these creative strategies still exploit the natural polarity of olefins, where the internal carbon has electrophilic character and reacts with a nucleophile.

In contrast, only a single strategy from the Shenvi group has been reported that is able to reverse the inherent polarity such that the internal carbon atom is nucleophilic in character (Scheme 1c) and can subsequently form a new C–C bond with an electrophile. This creative work relies on transmetallation from an oxidatively generated alkyl-Co(iii) intermediate to a stoichiometric chromium salt.^40^

We envisioned an alternative strategy to overturn this polarity paradigm that would instead merge MHAT catalysis with reductive radical–polar–crossover (RRPC).^41^ We hypothesised that single electron reduction of the radical intermediate should yield either an anionic or alkyl-Co(ii) species where the internal carbon would have nucleophilic character. However, there have been very few previous reports on this type of reactivity, doubtless because of the incompatibility of the traditional catalytic cycle for metal hydride formation – which requires an oxidant and a hydride source – with a single electron reduction step. Examples that had been reported either demonstrate solely protonation of the anionic intermediate to yield reduction products^42,43^ or proceed through a π-allyl-Co(iii) intermediate *via* migratory insertion of a Co(iii)–H into a diene, followed by single electron reduction to form a nucleophilic π-allyl-Co(ii) species.^44^

Another solution to this problem could lie in a recent development in the field. Our group^16,17,19,45,46^ and others^15,30,47–49^ have pioneered an alternative reductive cycle for metal hydride formation. This approach involves photocatalytic or electrocatalytic reduction of a Co(ii) catalyst to Co(i) which, upon protonation, yields the Co(iii)-H.^50^ Under these conditions, we proposed that it may now be possible to unlock the proposed new reactivity with RRPC, thus enabling the overall Markovnikov hydrofunctionalisation of an alkene with an electrophile. In this way, we would be able to overturn the inherent polarity of the system relative to all other existing reports in the field.

Herein, we report our work on developing this reaction using carbon dioxide as the electrophile (Scheme 1d). The successful implementation of this strategy represents a new dimension for MHAT catalysis, yielding Markovnikov hydrocarboxylation products under mild conditions *via* a mechanistic pathway that is distinct compared to other metals such as nickel.^51,52^ For instance, pioneering work from König and coworkers had relied on 10 mol% of a nickel catalyst which is sensitive to water. The reaction is suggested to proceed *via* concerted hydrometallation and the new C–C bond formation takes place from an organonickel intermediate. Synthesis of quaternary centres or substrates containing carbon-halide bonds are not reported.

## Results and discussion

We began investigating conditions using 4CzIPN as a photocatalyst because this has been exploited for a number of radical-polar crossover reductive carboxylations^53–56^ as well as by our group for MHAT-catalysed reductive coupling of dienes with ketones and imine electrophiles.^19^ After optimisation (see SI for further details), we were delighted to obtain a good yield of the hydrocarboxylated product from 1,1-diphenylethene in Markovnikov-fashion. Our optimised conditions use just 3.0 mol% of commercially available (*S*,*S*)–Co(salen)^*t*Bu,*t*Bu^, 1.0 mol% of 4CzIPN, 2.0 equivalents of Hantzsch Ester (HEH) as a reductant and a balloon of CO_2_ with DMF as solvent. Notably all reagents and catalysts were required for formation of the product and the reaction did also not proceed without visible light.

**Scheme 2:**
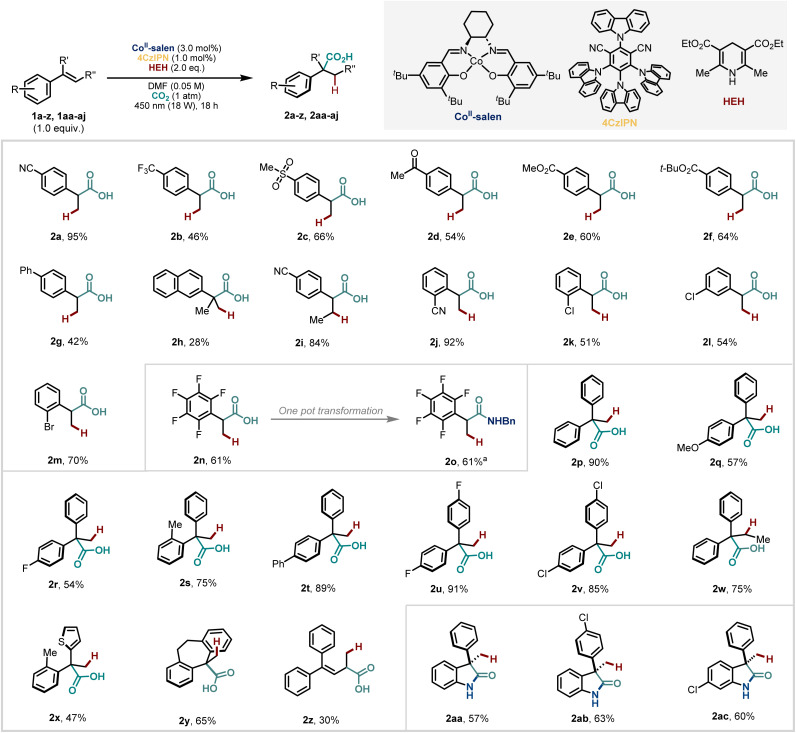
Substrate scope. Performed with alkenes 1a–1aj (0.1 mmol, 1.0 equiv.). One pot reaction using 1.1 equiv. of *N*-benzyl amine yielded amide in quantitative yield within 4 hours of hydrocarboxylation. See the SI for further details.

With these conditions in hand, we turned to the exploration of the reaction scope (Scheme 2). A range of 2-arylpropanoic acids were synthesised in moderate to excellent yields with good functional group tolerance. Notably, despite the proposed intermediate carbanion, which is generated catalytically, the reaction with CO_2_ is highly chemoselective, allowing the inclusion of various electrophilic functionalities at different positions of the aromatic ring such as nitrile (including a β-methyl substituted styrene) (2a–b, 2i–j), sulfone (2c), ketone (2d) and ester (2e–f) groups. 4CzIPN is also an effective catalyst for the dehalogenation of aryl halides to generate aryl radicals.^57^ However, in our case, halide-containing substrates (2k–n) and even electron neutral styrenes like (2g–h) were tolerated demonstrating the robustness of this method. With electron-donating substituents on the aromatic ring, no hydrocarboxylation products are observed which we suggest is a result of the higher potential needed to access the benzylic anion from the corresponding radical (see mechanistic section for further discussion).

Carboxylic acids are versatile handles that can easily be converted to other diverse functionalities. The ability to do this without additional steps such as isolation and purification saves time, cost, and energy. As such, we were pleased to show that a one-pot amidation of carboxylic acid (2n) led to an amide product (2o) in a similar good yield where reaction components from the carboxylation apparently do not interfere with the second step.

Next, we explored hydrocarboxylation of 1,1-diaryl olefins. Our strategy proved to be general to both electron-rich (2p–q, 2s–t) and electron-deficient (2r, 2u–v) systems, and β-substitution was well-tolerated (2w). Heteroarenes, for example, thiophene, could also be included (2x). These examples and others (2h, 2y) also demonstrate that our method is able to construct highly congested quaternary centres, a motif that can still challenge synthetic chemists and which has not been possible using other transition metals.^51^ We also tested a conjugated diene under our developed conditions and isolated a mixture of products from hydrocarboxylation at the allylic (2z) and benzylic positions in a 3 : 1 ratio (see SI for further details).

Given the functional group tolerance of the method, we were interested to try substrates with an unprotected pendant *ortho*-NH_2_ group, that would be able to cyclise *in situ* to form substituted 2-oxindoles^58^ even in the presence of the cobalt catalyst with vacant coordination sites. Although the presence of an amino group makes the starting styrene electron-rich, we still observed the formation of γ-lactams (2aa–ac) in synthetically useful yields under our standard conditions.

**Scheme 3:**
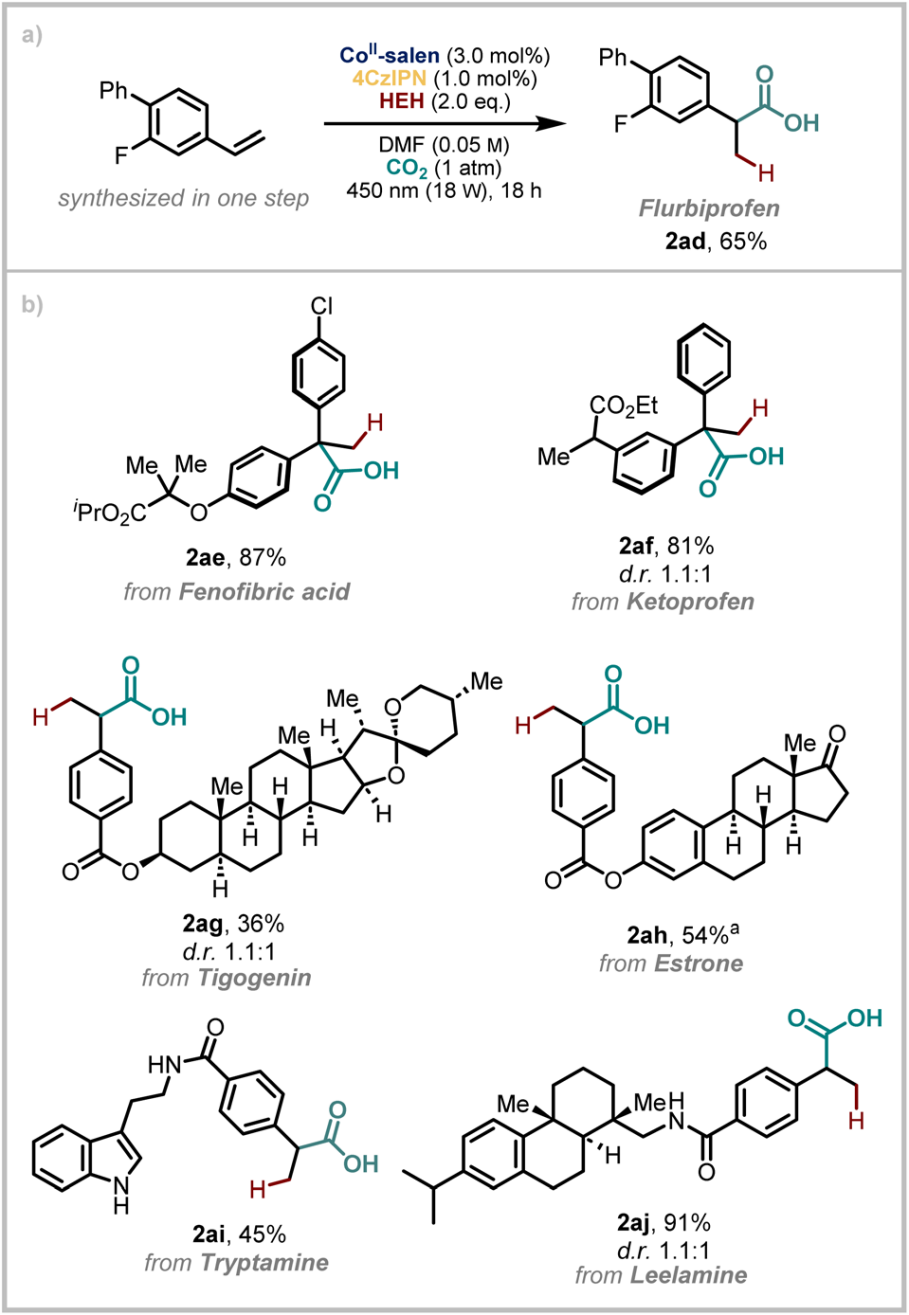
Applications of the method to drug synthesis and late-stage-functionalisation. ^a^For details on the d.r., see SI.

The importance of carboxylic acids in drug discovery comes from their ability to form significantly strong electrostatic interactions and single or bifurcated hydrogen bond bridges with protein targets which confers specific binding capability.^59^ On this basis, we wanted to demonstrate the robustness of our developed methodology in the context of complex, drug(-like) molecules (Scheme 3). We began by demonstrating that Flurbiprofen (2ad), a nonsteroidal anti-inflammatory drug (NSAID), could be synthesised from the corresponding styrene in good yield on 1.0 mmol scale. Next, we elected to take two diaryl ketone containing drugs, Fenofibric acid and Ketoprofen and performed a simple Wittig reaction. Hydrocarboxylation of the resulting olefins using our conditions then yields products 2ae and 2af which have a new quaternary centre in place of the ketone and a carbonyl that has been transposed by one atom. However, the rest of the structure remains unchanged.

We then appended styrene functional handles to other bioactive molecules, estrone (a female sex hormone), tigogenin (a gout suppressant and a plant metabolite), tryptamine (a metabolite of the essential amino acid, tryptophan) and leelamine. This yielded a diverse range of derivatives (2ag–2aj) which underscore the functional group tolerance of this method. Consistent with related studies, functional groups containing semi-labile N–H bonds can be included in the substrates.^56^

**Scheme 4:**
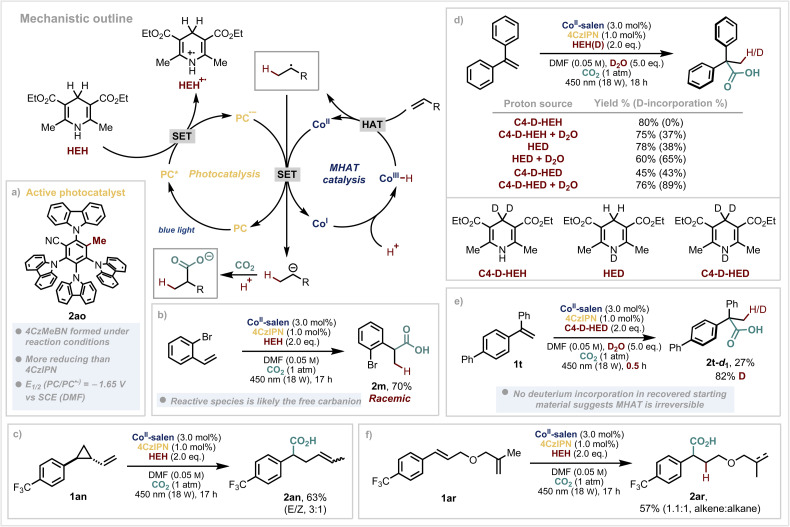
Mechanistic investigations. (a) Physical properties of 4CzMeBN. (b) Investigation of stereoselectivity. (c) Radical clock reaction. (d) Deuterium incorporation studies. (e) Reversibility analysis of MHAT. (f) Selectivity studies with tethered 1,1-disubstituted olefin.

At this point, we sought to test our initial hypothesis for the reductive radical polar reaction mechanism. When looking at the scope of substrates that are successful, the reduction of the intermediate benzylic radicals (*E*_red_ ≈ −0.7 to −1.3 V *vs.* SCE),^60^ almost matches the oxidation potential of 4CzIPN˙^−^ (*E*_ox_ = 1.21 V *vs.* SCE).^61^ However, König and coworkers have reported that benzylic radicals can substitute one of the nitrile groups of 4CzIPN and convert it into a more reducing, blue-shifted species.^62^ Since our reaction also involves the formation of benzylic radicals *via* MHAT catalysis, we anticipated similar behaviour could occur under our reaction conditions. Upon careful analysis of our reactions, we discovered that 4CzIPN is partially converted to 4CzMeBN (2ao) where one of the nitrile groups has been transformed to a methyl group (Scheme 4a). This has an estimated *E*_red_ = −1.65 V *vs.* SCE which gives an alternative route by which the Co(ii) catalyst could be reduced (*E*_red_ = −1.6 V *vs.* SCE) to Co(i) before reacting with a proton to generate the crucial Co(iii)–H species, essential for MHAT to the alkene (see SI for further details).

Given that we use an enantiopure (commercially available) cobalt catalyst, we also investigated the stereoselectivity of the reaction. The product is obtained as a racemate (Scheme 4b) – which, based on results from a previous method using the same cobalt catalyst,^63^ would imply that a Co(ii)–benzyl intermediate is unlikely to be involved in the C–C bond forming step in our case. However, we cannot conclusively rule out an alternative mechanistic scenario.^64^ Cyclopropyl containing substrate 1an opened under the reaction conditions to yield product 2an, giving additional evidence for a reductive radical–polar–crossover reaction (Scheme 4c). Although protons are present in the reaction mixture, these are likely generated in small amounts and quickly consumed by the cobalt which could explain the observation that the carbanion reacts preferentially as a nucleophile with the excess of CO_2_ (ref. 65) rather than a base.^66^ This mechanistic scenario is notably distinct to that with nickel, where key steps include hydrometallation and bond formation from an organonickel intermediate.^51,67^

Our attention next turned to the source of protons in the reaction (Scheme 4d). Use of deuterated Hantzsch Esters (either at the C4 position, nitrogen or both) demonstrated that a high incorporation of deuteration arises from the N–D derivatives. As we have found in our previous studies,^17,19,45^ addition of D_2_O further increases the deuterium percentage which suggest multiple proton sources in the reaction mixture. Stopping the reaction at partial conversion and reisolating the remaining starting material showed no deuterium incorporated, indicating a non-reversible MHAT step to the substrate 2t-d_1_ (Scheme 4e). When using a substrate which has a tethered 1,1-disubstituted olefin (2ar), classically excellent substrates for MHAT,^68^ we observe no hydrocarboxylation or cyclisation from this alkene – only partial reduction (Scheme 4f). The styrenyl olefin undergoes smooth hydrocarboxylation, however, demonstrating the unusual selectivity of photoinduced MHAT catalysis. It is also worth noting that acrylates do not yield hydrocarboxylation products using our system which is likely a result of the slow, polarity mismatched MHAT step to the olefin.^8^ This provides distinct selectivity compared to methods which involve conventional hydrometallation.

## Conclusions

In summary, we report an example of merging reductive MHAT catalysis and radical-polar-crossover chemistry to enable a mild method for the hydrocarboxylation of activated olefins with CO_2_. Low catalyst loadings and no stoichiometric metallic reductants are hallmarks of this method. The distinctive mechanistic pathway offers orthogonal selectivity compared to previously reported methods and we demonstrate applications to drug molecule synthesis and late-stage-functionalisation of drug-like molecules.

## Author contributions

S. J., D. A. K., N. V., D. L. and C. J. T. conceptualised this work and designed the experiments. S. J., D. A. K., N. V. and D. L. performed the experiments and analysed the data. S. J. and C. J. T. drafted the initial manuscript and all authors contributed to the final version. C. J. T. supervised the project and acquired funding. All authors contributed to the final version.

## Conflicts of interest

There are no conflicts to declare.

## Abbreviations

SCE: Saturated calomel electrode
DMF: *N*,*N*-dimethyl formamide
4CzIPN: 1,2,3,5-Tetrakis(carbazol-9-yl)-4,6-dicyanobenzene
4CzMeBN: (2,3,4,6-Tetra(9H-carbazol-9-yl)-5-methylbenzonitrile)

## Supplementary Material

SC-017-D5SC06119A-s001

## Data Availability

The data supporting this article have been included as part of the supplementary information (SI). Supplementary information: experimental data for this article, including analytical spectra are available. See DOI: https://doi.org/10.1039/d5sc06119a.
