# Supplementary material for: Inverting polarity in a cobalt MHAT reaction via reductive catalytic turnover
Source: Chem Sci. 2025 Nov 10;17(2):1098–104. doi: 10.1039/d5sc06119a (PMC12628006; doi:10.1039/d5sc06119a)
Supplement: SC-017-D5SC06119A-s001 [file SC-017-D5SC06119A-s001.pdf]

Supplementary Information (SI) for Chemical Science.
This journal is © The Royal Society of Chemistry 2025

ELECTRONIC SUPPLEMENTARY INFORMATION (ESI)

Inverting polarity in a cobalt MHAT reaction via reductive catalytic turnover

Samikshan Jana,[+] Daniel A. Kusza,[+] Nikita Vystavkin,[+]
Danijela Lunic and Christopher J. Teskey*

Institute of Organic Chemistry, Technische Universität Braunschweig
Hagenring 30, 38106 Braunschweig, Germany

Table of contents

Materials and Methods 7

1. Optimization of reaction conditions 8

1.1. Control reactions 9
1.2. Modifications of photocatalyst 9
1.3. Modifications of cobalt catalyst 9
1.4. Modification of Hantzsch Ester 10
1.5. Loadings of Hantzsch Ester 10
1.6. Modifications of solvents 10
1.7. Modifications of solvent concentration 10
1.8. Additive screening 11
2. General procedures 11

2.1. Preparation of substituted Hantzsch ester 11
Diethyl 4-cyclohexyl-2,6-dimethyl-1,4-dihydropyridine-3,5-dicarboxylate (HEH-Cy) 11

2.2. Preparation procedures of starting materials 11
1-(methylsulfonyl)-4-vinylbenzene (1c) 11

1-(4-vinylphenyl)ethan-1-one (1d) 12

tert-butyl 4-vinylbenzoate (1f) 12

(*E*)-4-(prop-1-en-1-yl)benzonitrile (1i) 12

2-vinylbenzonitrile (1j) 13

1-bromo-2-vinylbenzene (1m) 13

1-methoxy-4-(1-phenylvinyl)benzene (1q) 13

1-fluoro-4-(1-phenylvinyl)benzene (1r) 14

1-methyl-2-(1-phenylvinyl)benzene (1s) 14

4-(1-phenylvinyl)-1,1'-biphenyl (1t) 15

4,4'-(ethene-1,1-diyl)bis(fluorobenzene) (1u) 15

4,4'-(ethene-1,1-diyl)bis(chlorobenzene) (1v) 15

Prop-1-ene-1,1-diyldibenzene (1w) 16

2-(1-phenylvinyl)thiophene (1x) 16

5-methylene-10,11-dihydro-5*H*-dibenzo[*a*,*d*][7]annulene (1y) 16

Buta-1,3-diene-1,1-diyldibenzene (1z) 17

2-(1-phenylvinyl)aniline (1aa) 17

2-(1-(4-chlorophenyl)vinyl)aniline (1ab) 18

5-chloro-2-(1-phenylvinyl)aniline (1ac) 18

3-fluoro-4-vinyl-1,1'-biphenyl (1ad) 18

Isopropyl 2-(4-(4-chlorobenzoyl)phenoxy)-2-methylpropanoate (1ae’) 19

Isopropyl 2-(4-(1-(4-chlorophenyl)vinyl)phenoxy)-2-methylpropanoate (1ae) 19

Ethyl 2-(3-benzoylphenyl)propanoate (1af’) 20
Ethyl 2-(3-(1-phenylvinyl)phenyl)propanoate (1af) 20
(2a*S*,5'*S*,6a*S*,6b*S*,8a*S*,8b*R*,9*S*,10*S*,11a*S*,12a*S*,12b*R*)-5',6a,8a,9-tetramethyldocosahydro-spiro[naphtho[2',1':4,5]indeno[2,1-*b*]furan-10,2'-pyran]-4-yl 4-vinylbenzoate (1ag) 20
(8*R*,9*S*,13*S*,14*S*)-13-methyl-17-oxo-7,8,9,11,12,13,14,15,16,17-decahydro-6*H*-cyclopenta[*a*]phenanthren-3-yl 4-vinylbenzoate (1ah) 21
N-(2-(1*H*-indol-3-yl)ethyl)-4-vinylbenzamide (1ai) 21
N-(((1*S*,4a*R*,10a*S*)-7-isopropyl-1,4a-dimethyl-1,2,3,4,4a,9,10,10a-octahydrophenanthren-1-yl)methyl)-4-vinylbenzamide (1aj) 22
1-(trifluoromethyl)-4-(2-vinylcyclopropyl)benzene (1an) 22
[(*E*)-3-(2-methylallyloxy)prop-1-enyl]-4-(trifluoromethyl)benzene (1ar) 23
3. Products characterization data 25
3.1. Hydrocarboxylation product 25
2-(4-cyanophenyl)propanoic acid (2a) 25
2-(4-(trifluoromethyl)phenyl)propanoic acid (2b) 25
2-(4-(methylsulfonyl)phenyl)propanoic acid (2c) 25
2-(4-acetylphenyl)propanoic acid (2d) 25
2-(4-(methoxycarbonyl)phenyl)propanoic acid (2e) 26
2-(4-(*tert*-butoxycarbonyl)phenyl)propanoic acid (2f) 26
2-([1,1'-biphenyl]-4-yl)propanoic acid (2g) 26
2-methyl-2-(naphthalen-2-yl)propanoic acid (2h) 26
2-(4-cyanophenyl)butanoic acid (2i) 26
2-(2-cyanophenyl)propanoic acid (2j) 27
2-(2-chlorophenyl)propanoic acid (2k) 27
2-(3-chlorophenyl)propanoic acid (2l) 27
2-(2-bromophenyl)propanoic acid (2m) 27
2-(perfluorophenyl)propanoic acid (2n) 27
2,2-diphenylpropanoic acid (2p) 28
2-(4-methoxyphenyl)-2-phenylpropanoic acid (2q) 28
2-(4-fluorophenyl)-2-phenylpropanoic acid (2r) 28
2-phenyl-2-(*o*-tolyl) propanoic acid (2s) 28
2-([1,1'-biphenyl]-4-yl)-2-phenylpropanoic acid (2t) 28
2,2-bis(4-fluorophenyl)propanoic acid (2u) 29
2,2-bis(4-chlorophenyl)propanoic acid (2v) 29
2,2-diphenylbutanoic acid (2w) 29

2-phenyl-2-(thiophen-2-yl)propanoic acid (2x) 29

5-methyl-10,11-dihydro-5*H*-dibenzo[*a*,*d*][7]annulene-5-carboxylic acid (2y) 29

2-methyl-4,4-diphenylbut-3-enoic acid (2z major) and α-Methyl-α-[(1*E*)-3-phenyl-1-propen-1-yl]benzeneacetic acid (2z minor) 30

3-methyl-3-phenylindolin-2-one (2aa) 30

3-(4-chlorophenyl)-3-methylindolin-2-one (2ab) 30

6-Chloro-3-methyl-3-phenylindolin-2-one (2ac) 30

2-(3-fluoro-[1,1'-biphenyl]-4-yl)propanoic acid (2ad) 31

2-(4-chlorophenyl)-2-(4-((1-isopropoxy-2-methyl-1-oxopropan-2-yl)oxy)phenyl)propanoic acid (2ae) 31

2-(3-(1-ethoxy-1-oxopropan-2-yl)phenyl)-2-phenylpropanoic acid (2af) 31

(2*R*)-2-(4-((((2*aS*,5'*S*,6*aS*,6*bS*,8*aS*,8*bR*,9*S*,10*S*,11*aS*,12*aS*,12b*R*)-5',6*a*,8*a*,9-tetramethyldocosahydrospiro[naphtho[2',1':4,5]indeno[2,1-*b*]furan-10,2'-pyran]-4-yl)oxy)carbonyl)phenyl)propanoic acid (2ag) 32

2-(4-((((8*R*,9*S*,13*S*,14*S*)-13-methyl-17-oxo-7,8,9,11,12,13,14,15,16,17-decahydro-6*H*-cyclopenta[*a*]phenanthrene-3-yl)oxy)carbonyl)phenyl)-propanoic acid (2ah) 32

2-(4-((2-(1*H*-indol-3-yl)ethyl)carbamoyl)phenyl)propanoic acid (2ai) 33

2-(4-((((1*S*,4*aR*,10*aS*)-7-isopropyl-1,4a-dimethyl-1,2,3,4,4*a*,9,10,10*a*-octahydrophenanthren-1-yl)methyl)carbamoyl)phenyl)propanoic acid (2aj) 33

3.2. One-pot hydrocarboxylation-amidation reaction 33

N-benzyl-2-(perfluorophenyl)propenamide (2o) 33

4. Unsuccessful substrates 34

5.1. Investigation into stereoselectivity 35

(±)-2-(2-bromophenyl)propanoic acid (2ar) 35

Chiral high-performance liquid chromatography (HPLC) analysis: 35

5.2. Deuterium experiments 36

Diethyl 2,6-dimethyl-1,4-dihydropyridine-3,5-dicarboxylate-1-*d* (HED) 36

Diethyl 2,6-dimethyl-1,4-dihydropyridine-3,5-dicarboxylate-4,4-d_2 (*C4-D*-HEH) 36

Diethyl 2,6-dimethyl-1,4-dihydropyridine-3,5-dicarboxylate-1,4,4-d_3 (*C4-D*-HED) 36

2,2-diphenylpropanoic-3-d acid (C4-D-HEH) 37

2,2-diphenylpropanoic-3-*d* acid (*C4-D*-HE, with D_2O) 37

2,2-diphenylpropanoic-3-*d* acid (HEH with D_2O) 38

2,2-diphenylpropanoic-3-*d* acid (*N-D*-HEHH) 38

2,2-diphenylpropanoic-3-*d* acid (HED with D_2O) 39

4-(1-phenylethyl-1,2-d_2)-1,1'-biphenyl (HEH with D_2O) 39

4-(1-phenylethyl)-1,1'-biphenyl (HEH with DMF-d_7) 40

2,2-diphenylpropanoic-3-*d* acid (*C4-D*-HED) 41
4-(1-phenylethyl-1,2-d_2)-1,1'-biphenyl (*C4-D*-HED) 41
Reversibility of MHAT 42
5.3. Photosubstituted photocatalyst studies 43
2,3,4,6-Tetra(9*H*-carbazol-9-yl)-5-methylbenzonitrile (4CzMeBN) (2ao) 43
2,3,4,6-tetra(9*H*-carbazol-9-yl)-5-(methyl-*d3*)benzonitrile (4CzMe-d_3-BN) (2ao-d_3) 45
5.4. UV-Vis experiments 47
5.5. Fluorescence quenching studies 50
5.5.1. 1,2,3,5-Tetrakis(carbazol-9-yl)-4,6-dicyanobenzene (4CzIPN) 50
5.5.2. 2,3,4,6-Tetra(9*H*-carbazol-9-yl)-5-methylbenzonitrile (4CzMeBN) 54
5.6. Cyclic voltammetry experiment and redox potentials 57
5.7. Radical ring opening 59
6. Spectra 60
Starting materials 60
Isopropyl 2-(4-(1-(4-chlorophenyl)vinyl)phenoxy)-2-methylpropanoate (1ae) 60
Ethyl 2-(3-benzoylphenyl)propanoate (1af’) 61
Ethyl 2-(3-(1-phenylvinyl)phenyl)propanoate (1af) 62
Substrate scope 65
2-(4-cyanophenyl)propanoic acid (2a) 65
2-(4-(trifluoromethyl)phenyl)propanoic acid (2b) 66
2-(4-(methylsulfonyl)phenyl)propanoic acid (2c) 68
2-(4-acetylphenyl)propanoic acid (2d) 69
2-(4-(methoxycarbonyl)phenyl)propanoic acid (2e) 70
2-(4-(tert-butoxycarbonyl)phenyl)propanoic acid (2f) 71
2-([1,1'-biphenyl]-4-yl)propanoic acid (2g) 72
2-methyl-2-(naphthalen-2-yl)propanoic acid (2h) 73
2-(4-cyanophenyl)butanoic acid (2i) 74
2-(3-chlorophenyl)propanoic acid (2l) 77
2-(2-bromophenyl)propanoic acid (2m) 78
2-(perfluorophenyl)propanoic acid (2n) 79
N-benzyl-2-(perfluorophenyl)propenamide (2o) 80
2,2-diphenylpropanoic acid (2p) 82
2-(4-methoxyphenyl)-2-phenylpropanoic acid (2q) 83
2-(4-fluorophenyl)-2-phenylpropanoic acid (2r) 84
2-phenyl-2-(o-tolyl)propanoic acid (2s) 85
2-([1,1'-biphenyl]-4-yl)-2-phenylacetic acid (2t) 86

2,2-bis(4-fluorophenyl)propanoic acid (2u) 87
2-phenyl-2-(thiophen-2-yl)propanoic acid (2x) 91
5-methyl-10,11-dihydro-5H-dibenzo[*a*,*d*][7]annulene-5-carboxylic acid (2y) 92
2-methyl-4,4-diphenylbut-3-enoic acid (2z major) and α-Methyl-α-[(1*E*)-3-phenyl-1-propen-1-yl]benzeneacetic acid (2z minor) 93
3-methyl-3-phenylindolin-2-one (2aa) 94
3-(4-chlorophenyl)-3-methylindolin-2-one (2ab) 95
6-chloro-3-methyl-3-phenylindolin-2-one (2ac) 96
2-(3-fluoro-[1,1'-biphenyl]-4-yl)propanoic acid (2ad) 97
2-(4-chlorophenyl)-2-(4-((1-isopropoxy-2-methyl-1-oxopropan-2-yl)oxy)phenyl)propanoic acid (2ae) 98
2-(3-(1-ethoxy-1-oxopropan-2-yl)phenyl)-2-phenylpropanoic acid (2af) 99
2-(4-((((2aS,5'R,6aS,6bS,8aS,8bR,9S,10R,11aS,12aS,12bR)-5',6a,8a,9-tetramethyldocosahydrospiro[naphtho[2',1':4,5]indeno[2,1-b]furan-10,2'-pyran]-4-yl)oxy)carbonyl)phenyl)propanoic acid (2ag) 100
2-(4-((((8R,9S,13S,14S)-13-methyl-17-oxo-7,8,9,11,12,13,14,15,16,17-decahydro-6H-cyclopenta[a]phenanthren-3-yl)oxy)carbonyl)phenyl)propanoic acid (2ah) 101
2-(4-((2-(1H-indol-3-yl)ethyl)carbamoyl)phenyl)propanoic acid (2ai) 102
2-(4-((((1S,4aR,10aS)-7-isopropyl-1,4a-dimethyl-1,2,3,4,4a,9,10,10a-octahydrophenanthren-1-yl)methyl)carbamoyl)phenyl)propanoic acid (2aj) 103

Photosubstituted photocatalyst experiments 105
2,3,4,6-tetra(9H-carbazol-9-yl)-5-methylbenzonitrile (4CzMeBN) (2ao) 105
Oxidized-Hantzsch ester (HEH) and 2,3,4,6-tetra(9*H*-carbazol-9-yl)-5-methylbenzonitrile 4CzMeBN (7.7 : 1) 106
Reaction for the synthesis of 4CzMe(d_3)BN with DMF-d_7 107

Radical ring opening 109
2-(4-(trifluoromethyl)phenyl)hex-4-enoic acid (2an) 109

Tethered 1,1-disubstituted olefin 111
4-((2-methylallyl)oxy)-2-(4-(trifluoromethyl)phenyl)butanoic acid (2ar) 111

7. References 112

Materials and Methods

Unless otherwise stated, all reactions were performed with standard Schlenk techniques under argon atmosphere. All reagents and starting materials were purchased at reagent grade and used as received. Anhydrous solvents were dried using an Innovative Technology PS-MD-5 solvent purification system. Thin layer chromatography (TLC) was performed on Merck Kieselgel 60 F254 aluminium plates with unmodified silica and visualized either under UV light or stained with potassium permanganate, *p*-anisaldehyde stain, cerium ammonium molybdate (Hanessian's stain) or bromocresol green. Column chromatography was performed with Merck silica gel 60 (35 – 70 mesh). Preparative TLC was performed using pre-coated TLC plates SIL G-50 UV250 (Layer: 0.50 mm silica gel 60 with fluorescent indicator UV_{250}); detection under UV light.

All ^{1}H, ^{13}C NMR and ^{19}F NMR spectra were recorded at ambient temperature with the following instruments: Bruker AV II-300 (^{1}H 300 MHz, ^{13}C 75 MHz), DRX-400 (^{1}H 400 MHz, ^{13}C 100 MHz) AV IIIHD-500 (^{1}H 500 MHz, ^{13}C 125 MHz) or AV II-600 (^{1}H 600 MHz, ^{13}C 151 MHz). Chemical shifts (δ/ppm) were referenced to the residual solvent peak in ^{1}H (7.26 ppm for $CDCl_3$) and ^{13}C spectra (77.16 ppm for $CDCl_3$). Coupling constants (*J*) are given in Hz. Signals are described as br = broad, s = singlet, d = doublet, dd = doublet of doublets, ddd = doublet of doublet of doublets, dddd = doublet of doublet of doublet of doublets, dt = doublet of triplets, ddt = doublet of doublet of triplets, dq = doublet of quartets, t = triplet, tt = triplet of triplets, q = quartet, p = quintet, h = sextet and, hept = heptet, m = multiplet.

High-resolution mass spectrometry (HRMS) was performed using Thermo Scientific LTQ Orbitrap XL spectrometer. Electrospray ionization (ESI) was performed using ThermoFisher Scientific LTQ Orbitrap Velos. Electrospray measurements were performed in direct infusion mode using a custom made microspray-device mounted on a Proxeon nanospray ion source. The microspray-device allows for the sample infusion through a stainless-steel capillary (90 µm ID). Accurate mass measurements in the orbitrap were performed using the lock mass option of the instrument control software using the cation of tetradecyltrimethylammonium bromide (256.29988 amu) as internal mass reference.

IR spectra were recorded with a Bruker Tensor 27 or Bruker Alpha-P spectrometer using diamond ATR technique and signals reported as wavenumbers in reciprocal centimetres. HPLC was performed using a DAICEL® Chiralpak IC-3, (150 x 4.6) mm, 5 µm, 99:1 n-hexane/*i*PrOH at 1.0 mL/min flow rate.

All the photochemical reactions were carried out in an EvoluChem™ PhotoRedOxBox Duo equipped with two EvoluChem™ LED 450 PF lamps (λ_{max} = 450 nm, 18 W).

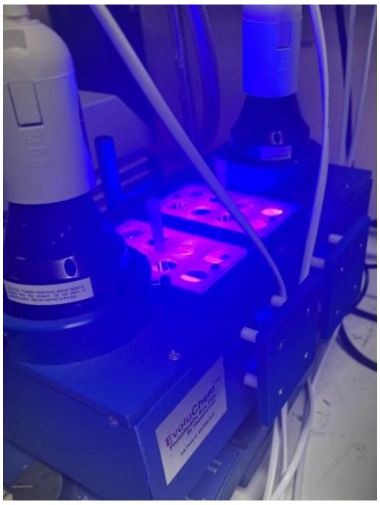

Picture S1. Photo of the set-up.

1. Optimization of reaction conditions

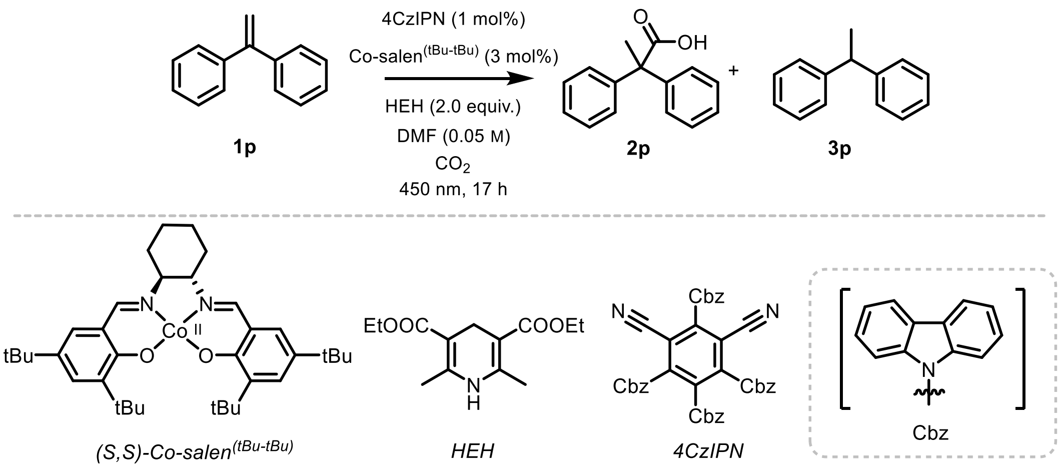

Scheme S1. A general scheme for the optimization of reaction conditions.

The optimizations of the reaction conditions were carried out on 0.1 mmol scale, using **1p** as a starting material. The yields were determined using internal standard $CHBr_3$ or CH_2Br_2 (1.0 eq, 0.1 mmol).

The optimised conditions are as follows:

An oven-dried round bottom flask was first put under an argon atmosphere and then filled with anhydrous *N*,*N*-dimethylformamide (DMF). It was then purged with argon for 15 min, followed by a CO_2 balloon for another 15 min. Then in a 4.0 mL oven-dried screw-capped vial loaded with a stirring bar – 1,1-diphenylethene (1.0 equiv., 0.1 mmol, 18.0 mg), photocatalyst (4CzIPN) (1 mol%, 1 µmol, 0.7 mg), (*S*,*S*)-Co-salen$^{(tBu,tBu)}$ (3 mol%, 3 µmol, 1.8 mg), Hantzsch ester (HEH) (2.0 equiv., 0.2 mmol, 50.1 mg) and *N*,*N*-dimethylformamide (0.05 M, 2.0 mL) previously purged with CO_2 was added. The reaction mixture was purged with a CO_2 balloon for 30 s and then irradiated with 450 nm light (18 W) for 18 h. Then the reaction was quenched by the addition of distilled water (12 mL). The organics were extracted using an ethyl acetate (EtOAc) (3 x 6 mL). Then washed with 10% lithium chloride solution (3 mL) to ensure the complete removal of DMF followed by a final wash with brine (3.0 mL). The presence of carboxylic acid product was determined using TLC analysis (R_f = approx. 0.4 – 0.5, DCM/MeOH = 95/5) and then stained with bromocresol green stain to reveal the carboxylic acid as a bright yellow spot on the TLC plate. The product was purified using silica column chromatography with 3 consecutive elutions. The first elution with 100 mL (pentane/EtOAc = 90/10), then the second elution with 100 mL (pentane/EtOAc = 75/25) and a final elution with 200 mL (DCM/MeOH = 95/5). In a few cases if residual DMF peaks were observed in the NMR then a subsequent wash with 10% lithium chloride solution was performed.

1.1. Control reactions

Entry	Variation	2p (%)[a]	3p (%)[a]	RSM (%)[a]
1	None	90	-	-
2	w/o 4CzIPN	-	-	-
3	w/o Co-salen$^{(tBu\text{-}tBu)}$	-	-	88
4	w/o HEH	-	-	89
5	w/o light	-	-	11
6	Under air	-	57	8
7	w/o CO_2	-	63	-

[a]yield determined by ^{1}H NMR analysis with bromoform or dibromomethane as an internal standard. RSM (Recovered Starting Material)

1.2. Modifications of photocatalyst

Entry	Photocatalyst (1 mol%) (Irradiation wavelength)	2p (%)[a]	3p (%)[a]	RSM (%)[a]
1	Ph-benzoPTZ (450 nm)	-	-	Dimers
2	$[Ir(dF(Me)ppy)_2(dtbbpy)]PF_6$ (450 nm)	53	27	-
3	Mes-Acr-Ph-BF_4 (450 nm)	-	-	Dimers
4	$[Ir(dtbbpy)(ppy)_2]PF_6$ (450 nm)	26	30	Dimers

[a]yield determined by ^{1}H NMR analysis with dibromomethane as an internal standard. RSM (Recovered Starting Material)

1.3. Modifications of cobalt catalyst

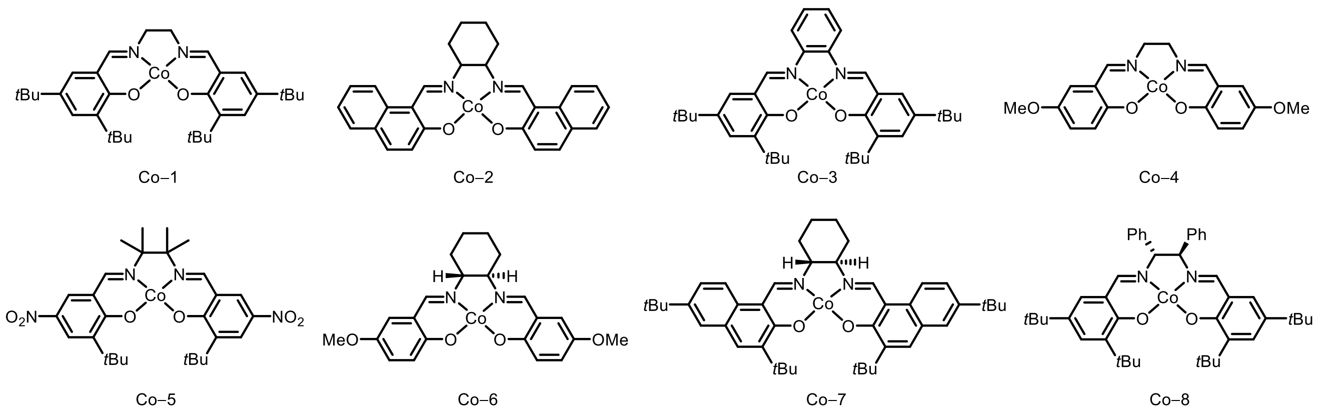

Entry	Cobalt catalyst (3 mol%)	2p (%)[a]	3p (%)[a]	RSM (%)[a]
1	Co-1	7	19	-
2	Co-2	24	22	-
3	Co-3	10	23	-
4	Co-4	4	8	-
5	Co-5	5	27	-
6	Co-6	57	19	-
7	Co-7	38	26	-
8	Co-8	45	29	-

[a]yield determined by ^{1}H NMR analysis with dibromomethane as an internal standard. RSM (Recovered Starting Material)

1.4. Modification of Hantzsch Ester

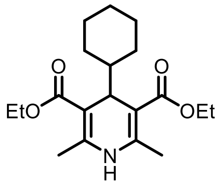

HEH-Cy

Entry	Hydride Donors	2p (%)[a]	3p (%)[a]	RSM (%)[a]
1	HEH-Cy	40	50	-

[a]yield determined by ^{1}H NMR analysis with dibromomethane as an internal standard. RSM (Recovered Starting Material)

1.5. Loadings of Hantzsch Ester

Entry	HEH(x equiv.)	2p (%)[a]	3p (%)[a]	RSM (%)[a]
1	0.1	-	-	100
2	0.3	-	-	100
3	0.5	-	14	Dimers & SM
4	1.0	-	14	Dimers & SM
5	1.5	32	24	Dimers & SM

[a]yield determined by ^{1}H NMR analysis with dibromomethane as an internal standard. RSM (Recovered Starting Material)

1.6. Modifications of solvents

Entry	Solvent (0.05 M)	2p (%)	3p (%)	RSM (%)
1	DMSO	13	40	Dimers & SM
2	THF	16	25	Dimers & SM
3	MeCN	12	44	Dimers & SM
4	Et_2O	-	18	Dimers & SM

[a]yield determined by ^{1}H NMR analysis with dibromomethane as an internal standard. RSM (Recovered Starting Material)

1.7. Modifications of solvent concentration

Entry	DMF [M]	2p(%)[a]	3p(%)[a]	RSM (%)[a]
1	0.2	17	52	Dimers & SM
2	0.1	15	45	Dimers & SM
3	0.025	15	16	Dimers & SM
4	0.0167	18	30	Dimers & SM

[a]yield determined by ^{1}H NMR analysis with dibromomethane as an internal standard. RSM (Recovered Starting Material)

1.8. Additive screening

Entry	Additive	2p (%)[a]	3p (%)[a]	RSM (%)[a]
1	Pyridine	35	28	Dimers
2	2,4,6-Collidine	31	36	Dimers

[a]yield determined by ^{1}H NMR analysis with dibromomethane as an internal standard. RSM (Recovered Starting Material)

2. General procedures

2.1. Preparation of substituted Hantzsch ester

Diethyl 4-cyclohexyl-2,6-dimethyl-1,4-dihydropyridine-3,5-dicarboxylate (HEH-Cy)

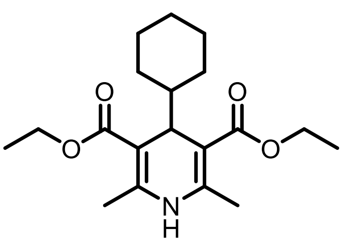

This compound was prepared according to the literature.[1] A mixture of cyclohexane carbaldehyde (1.2 mL, 10.0 mmol, 1.0 equiv.), ethyl acetoacetate (5.0 mL, 40.0 mmol, 4.0 equiv.), and ammonium acetate (1.54 g, 20.0 mmol, 2.0 equiv.) in 20 mL of distilled water was vigorously stirred at refluxing temperature for 17 h. Then, the reaction mixture was allowed to cool down to room temperature. The crude reaction mixture was extracted three times with CH_2Cl_2 and the combined organic phase was washed with brine. The organic layer was then dried over $MgSO_4$, filtered, and concentrated *in vacuo*. The desired product was obtained by recrystallization with ethanol as a yellow solid (1.59 g, 4.74 mmol, 47%).

^{1}H NMR (600 MHz, $CDCl_3$): δ 5.49 (s, 1H), 4.25 – 4.11 (m, 4H), 3.92 (d, *J* = 5.7 Hz, 1H), 2.30 (s, 6H), 1.65 (s, 2H), 1.55 – 1.48 (m, 2H), 1.30 (dt, *J* = 7.5, 6.7 Hz, 6H), 1.25 – 1.18 (m, 1H), 1.11 – 1.02 (m, 3H), 0.99 – 0.85 (m, 2H).**^{13}C NMR** (151 MHz, $CDCl_3$): δ 168.8, 144.5, 102.2, 59.7, 45.9, 38.6, 29.0,26.9, 26.8, 19.6, 14.5.

2.2. Preparation procedures of starting materials

Alkenes **1a-b**, **1e**, **1g-h**, **1k-l**, **1n** and **1p** were commercially available compounds.

1-(methylsulfonyl)-4-vinylbenzene (1c)

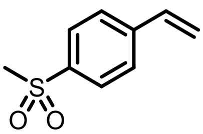

Wittig reaction was performed according to the previously reported procedure.[2]A suspension of methyltriphenylphosphonium bromide (1.4 g, 3.91 mmol, 1.1 equiv.) in 20 mL of anhydrous THF under an argon atmosphere was cooled to 0 °C on an ice bath. Then 1.6 M *n*-BuLi in hexanes (1.6 mL, 3.91 mmol, 1.1 equiv.) was added dropwise. The reaction mixture was stirred for 20 minutes at 0 °C. Then, the corresponding aldehyde was (0.65 g, 3.56 mmol, 1.0 equiv. in 4.0 mL of anhydrous THF) was added dropwise. The ice bath was removed, and the reaction mixture was stirred at room temperature overnight. After this time, the reaction was quenched with 10 mL saturated ammonium chloride solution and extracted with diethyl ether (3 x 8 mL). The collected organic phase was washed with brine and dried over $MgSO_4$. The mixture was concentrated in vacuo. Purification by flash column chromatography (pentane/Et_2O = 2/1) afforded a pale-yellow oil (0.41 g, 2.25 mmol, 63%).

^{1}H NMR (300 MHz, $CDCl_3$): δ 7.90 (d, *J* = 8.3 Hz, 2H), 7.57 (d, *J* = 8.2 Hz, 2H), 6.77 (dd, *J* = 17.6, 11.0 Hz, 1H), 5.91 (dd, *J* = 17.5, 0.6 Hz, 1H), 5.47 (dd, *J* = 10.9, 0.6 Hz, 1H), 3.05 (s, 3H). **^{13}C NMR** (75 MHz, $CDCl_3$): δ 143.1, 139.7, 135.6, 128.3, 127.9, 127.5, 127.0, 118.1, 44.6.
These data are in agreement with those reported previously in the literature.[3]

1-(4-vinylphenyl)ethan-1-one (1d)

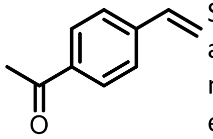

Suzuki reaction was performed according to the previously reported procedure.[4] To an oven-dried two neck round bottom flask was added 4-iodoacetophenone (492 mg, 2.00 mmol, 1.0 equiv.), vinyl boronic acid pinacol ester (0.68 mL, 4.00 mmol, 2.0 equiv.), $Pd(PPh_3)_4$ (94.0 mg, 80.0 μmol, 4 mol%), and potassium hydroxide (336 mg, 6.00 mmol, 3.0 equiv.). The flask was capped and attached to a reflux condenser and then purged with nitrogen before addition of THF (8 mL, 0.25 M), and trifluorotoluene (0.24 mL, 2.00 mmol, 1.0 equiv.). The reaction mixture was heated at 80 °C for 24 h. The vial was then decapped, diluted with CH_2Cl_2 (4.0 mL) and passed through a layer of celite. Then the product was isolated as a pale yellow solid (210 mg, 1.44 mmol, 72%) using flash column chromatography over silica gel (pentane/EtOAc = 97/3).

^{1}H NMR (300 MHz, $CDCl_3$): δ 7.92 (d, *J* = 8.3 Hz, 2H), 7.48 (d, *J* = 8.1 Hz, 2H), 6.75 (dd, *J* = 17.6, 10.9 Hz, 1H), 5.87 (d, *J* = 17.6 Hz, 1H), 5.39 (dd, *J* = 11.0, 0.7 Hz, 1H), 2.59 (s, 3H). **^{13}C NMR** (75 MHz, $CDCl_3$): δ 197.7, 142.1, 136.4, 135.9, 129.7, 126.7, 116.8, 26.5.
These data are in agreement with those reported previously in the literature. [5]

***tert*-butyl 4-vinylbenzoate (1f)**

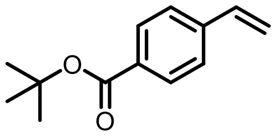

Esterification was done according to the previously reported procedure.[6] 4-vinyl benzoic acid (300 mg, 2.02 mmol, 1.0 equiv.), dicyclohexylcarbodiimide (DCC, 460 mg, 2.24 mmol, 1.12 equiv.), 4-(dimethylamino)pyridine (DMAP, 30.5 mg, 0.25 mmol, 0.12 equiv.), and *t*-butanol (0.3 g, 2.32 mmol, 1.15 equiv.) were stirred in 3.75 mL of dry CH_2Cl_2 until no further product formation was observed by thin-layer chromatography (approximately 2 h). The mixture was filtered, the precipitate was rinsed with CH_2Cl_2, and the combined filtrates were evaporated. The resulting colorless oil was purified by flash column chromatography on silica gel (pentane/EtOAc = 95/5) (175 mg, 0.86 mmol, 63%).

^{1}H NMR ($CDCl_3$, 300 MHz): δ 7.94 (d, *J* = 8.2 Hz, 2H), 7.44 (d, *J* = 8.2 Hz, 2H), 6.75 (dd, *J* = 20.5 Hz, 11.0 Hz, 1 H), 5.84 (d, *J* = 17.7 Hz,1 H), 5.36 (d, *J* = 10.7 Hz, 1 H), 1.59 ppm (s, 9 H). **^{13}C NMR** ($CDCl_3$, 75 MHz): δ 166.1, 140.9, 136.1, 131.2, 129.7, 124.8, 117.2, 80.7, 27.8.
These data are in agreement with those reported previously in the literature. [7]

(*E*)-4-(prop-1-en-1-yl)benzonitrile (1i)

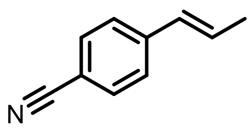

Wittig reaction was performed according to the previously reported procedure.[2] A suspension of ethyltriphenylphosphonium bromide (1.45 g, 3.91 mmol, 1.1 equiv.) in 20 mL of anhydrous THF under an argon atmosphere was cooled to 0 °C on an ice bath. Then 1.6 M *n*-BuLi in hexanes (1.6 mL, 3.91 mmol, 1.1 equiv.) was added dropwise. The reaction mixture was stirred for 20 min at 0 °C. Then, the corresponding aldehyde was (470 g, 3.56 mmol, 1.0 equiv. in 4.0 mL of anhydrous THF) was added dropwise. The ice bath was removed, and the reaction mixture was stirred at room temperature overnight. After this time, the reaction was quenched with 10 mL saturated ammonium chloride solution and extracted with diethyl ether (3 x 8 mL). The collected organic phase was washed with brine and dried over

$MgSO_4$. The mixture was concentrated *in vacuo*. Purification by flash column chromatography (pentane/EtOAc = 90/10) afforded as a colourless oil (180 mg, 1.26 mmol, 36%).

^{1}H NMR (300 MHz, $CDCl_3$): δ 7.36 (d, *J* = 8.3 Hz, 2H), 7.30 (d, *J* = 8.1 Hz, 2H), 6.46 – 6.38 (m, 2H), 1.88 (d, *J* = 4.8 Hz, 3H). **^{13}C NMR** (75 MHz, $CDCl_3$): δ 143.2, 133.6, 131.3, 129.8, 126.2, 119.8, 110.2, 18.5. These data are in agreement with those reported previously in the literature.[8]

2-vinylbenzonitrile (1j)

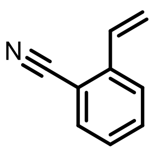

Wittig reaction was performed according to the previously reported procedure.[2] A suspension of methyltriphenylphosphonium bromide (1.4 g, 3.91 mmol, 1.1 equiv.) in 20.0 mL of anhydrous THF under an argon atmosphere was cooled to 0 °C on an ice bath. Then 1.6 M *n*-BuLi in hexanes (1.6 mL, 3.91 mmol, 1.1 equiv.) was added dropwise. The reaction mixture was stirred for 20 min at 0 °C. Then, the corresponding aldehyde was (446 mg, 3.56 mmol, 1.0 equiv. in 4.0 mL of anhydrous THF) was added dropwise. The ice bath was removed, and the reaction mixture was stirred at room temperature overnight. The reaction was quenched with 10 mL saturated ammonium chloride solution and extracted with diethyl ether (3 x 8 mL). The collected organic phase was washed with brine and dried over $MgSO_4$. The mixture was concentrated *in vacuo*. Purification by flash column chromatography (cyclohexane/EtOAc = 20/1) afforded a pale yellow oil (120 mg, 0.93 mmol, 26%).

^{1}H NMR (300 MHz, $CDCl_3$): δ 7.52-7.71 (m, 3H), 7.35 (td, *J* = 7.6 Hz, 1.3 Hz, 1H), 7.1 (dd, *J* = 10.6 Hz, 17.8 Hz, 1H), 5.96 (d, *J* = 18.0 Hz, 1H), 5.53 (d, *J* = 10.6 Hz, 1H). **^{13}C NMR** (75 MHz, $CDCl_3$): δ 140.6, 132.9, 132.8, 132.2, 127.9, 125.4, 118.5, 117.7, 111.2.
These data are in agreement with those reported previously in the literature.[9]

1-bromo-2-vinylbenzene (1m)

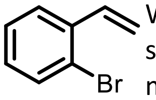

Wittig reaction was performed according to the previously reported procedure.[2] A suspension of methyltriphenylphosphonium bromide (1.4 g, 3.91 mmol, 1.1 equiv.) in 20 mL of anhydrous THF under an argon atmosphere was cooled to 0 °C on an ice bath. Then 1.6 M *n*-BuLi in hexanes (1.6 mL, 3.91 mmol, 1.1 equiv.) was added dropwise. The reaction mixture was stirred for 20 min at 0 °C. Then, the corresponding aldehyde was (0.41 mL, 3.56 mmol, 1.0 equiv. in 4.0 mL of anhydrous THF) was added dropwise. The ice bath was removed, and the reaction mixture was stirred at room temperature overnight. The reaction was quenched with 10 mL saturated ammonium chloride solution and extracted with diethyl ether (3 x 8 mL). The collected organic phase was washed with brine and dried over $MgSO_4$. The mixture was concentrated *in vacuo*. Purification by flash column chromatography (pentane/EtOAc = 99/1) afforded a colourless liquid (560 mg, 3.06 mmol, 84%).

^{1}H NMR (300 MHz, $CDCl_3$): δ 7.58 – 7.52 (m, 2H), 7.33 – 7.24 (m, 1H), 7.17 – 7.00 (m, 2H), 5.71 (dd, *J* = 17.4, 1.1 Hz, 1H), 5.37 (dd, *J* = 11.0, 1.1 Hz, 1H). **^{13}C NMR** (75MHz, $CDCl_3$): δ 137.3, 135.6, 132.6, 129.0, 127.7, 126.8, 123.5, 116.3.
These data are in agreement with those reported previously in the literature.[10]

1-methoxy-4-(1-phenylvinyl)benzene (1q)

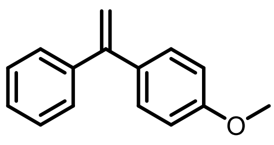

Wittig reaction was performed according to the previously reported procedure.[2] A suspension of methyltriphenylphosphonium bromide (1.4 g, 3.91 mmol, 1.1 equiv.) in 20 mL of anhydrous THF under an argon atmosphere was cooled to 0 °C on an ice bath. Then 1.6 M *n*-BuLi in hexanes (1.6 mL, 3.91 mmol, 1.1 equiv.) was added dropwise. The reaction mixture was stirred for 20 min at 0 °C. Then, the corresponding ketone was (750 mg, 3.56 mmol, 1.0 equiv. in 4.0 mL of anhydrous

THF) was added dropwise. The ice bath was removed, and the reaction mixture was stirred at room temperature overnight. The reaction was quenched with 10 mL saturated ammonium chloride solution and extracted with diethyl ether (3 x 8 mL). The collected organic phase was washed with brine and dried over $MgSO_4$. The mixture was concentrated *in vacuo*. Purification by flash column chromatography (pentane/EtOAc = 95/5) afforded a white solid (570 g, 2.71 mmol, 72%).

^{1}H NMR (300 MHz, $CDCl_3$): δ 7.37 – 7.24 (m, 7H), 6.89 – 6.82 (m, 2H), 5.39 (d, *J* = 1.0 Hz, 1H), 5.35 (d, *J* = 1.3 Hz, 1H), 3.82 (s, 3H). **^{13}C NMR** (75 MHz, $CDCl_3$): δ 159.5, 149.4, 141.7, 134.9, 129.4, 128.3, 128.1, 127.6, 113.5, 112.9, 55.5.
These data are in agreement with those reported previously in the literature.[11]

1-fluoro-4-(1-phenylvinyl)benzene (1r)

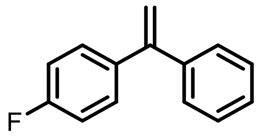

Wittig reaction was performed according to the previously reported procedure.[2] A suspension of methyltriphenylphosphonium bromide (970 mg, 2.69 mmol, 1.1 equiv.) in 20 mL of anhydrous THF under an argon atmosphere was cooled to 0 °C on an ice bath. Then 1.6 M *n*-BuLi in hexanes (1.7 mL, 2.69 mmol, 1.1 equiv.) was added dropwise. The reaction mixture was stirred for 20 min at 0 °C. Then, the corresponding ketone was (500 mg, 2.45 mmol, 1.0 equiv. in 4.0 mL of anhydrous THF) was added dropwise. The ice bath was removed, and the reaction mixture was stirred at room temperature overnight. The reaction was quenched with 10 mL saturated ammonium chloride solution and extracted with diethyl ether (3 x 8 mL). The collected organic phase was washed with brine and dried over $MgSO_4$. The mixture was concentrated *in vacuo*. Purification by flash column chromatography (pentane/EtOAc = 95/5) afforded a white solid (450 mg, 2.27 mmol, 92%).

^{1}H NMR (300 MHz, $CDCl_3$): δ 7.36 – 7.29 (m, 7H), 7.07 – 6.99 (m, 2H), 5.44 (ddd, *J* = 7.2, 1.1, 0.4 Hz, 2H). **^{13}C NMR** (75 MHz, $CDCl_3$): δ 162.5 (d, *J* = 246.7 Hz), 149.1, 141.3, 137.5 (d, *J* = 3.3 Hz), 129.9 (d, *J* = 8.0 Hz, 2C), 128.2 (2C), 128.2 (2C), 127.8, 115.0 (d, *J* = 21.4 Hz, 2C), 114.2 (d, *J* = 1.3 Hz). **^{19}F NMR** (282 MHz, $CDCl_3$): δ -115.1.
These data are in agreement with those reported previously in the literature.[12]

1-methyl-2-(1-phenylvinyl)benzene (1s)

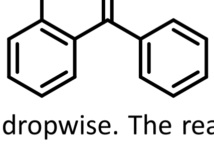

Wittig reaction was performed according to the previously reported procedure.[2] A suspension of methyltriphenylphosphonium bromide (930 g, 2.20 mmol, 1.1 equiv.) in 20 mL of anhydrous THF under an argon atmosphere was cooled to 0 °C on an ice bath. Then 1.6 M *n*-BuLi in hexanes (1.4 mL, 2.20 mmol, 1.1 equiv.) was added dropwise. The reaction mixture was stirred for 20 min at 0 °C. Then, the corresponding ketone was (390 mg, 2.00 mmol, 1.0 equiv. in 4.0 mL of anhydrous THF) was added dropwise. The ice bath was removed, and the reaction mixture was stirred at room temperature overnight. The reaction was quenched with 10 mL saturated ammonium chloride solution and extracted with diethyl ether (3 x 8 mL). The collected organic phase was washed with brine and dried over $MgSO_4$. The mixture was concentrated *in vacuo*. Purification by flash column chromatography (pentane/EtOAc = 95/5) afforded a white solid (270 mg, 1.39 mmol, 70%).

^{1}H NMR (300 MHz, $CDCl_3$): δ 7.31 – 7.15 (m, 9H), 5.76 (d, *J* = 1.5 Hz, 1H), 5.18 (d, *J* = 1.5 Hz, 1H), 2.04 (d, *J* = 1 Hz, 3H). **^{13}C NMR** (75 MHz, $CDCl_3$): δ 149.6, 141.8, 140.7, 136.3, 130.2, 130.2, 128.5 (2C), 127.7, 127.7, 126.6 (2C), 125.8, 115.0, 20.2.
These data are in agreement with those reported previously in the literature.[12]

4-(1-phenylvinyl)-1,1'-biphenyl (1t)

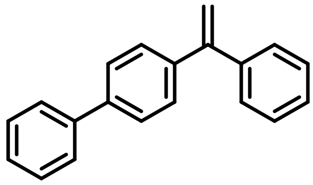

Wittig reaction was performed according to the previously reported procedure.[2] A suspension of methyltriphenylphosphonium bromide (960 mg, 2.69 mmol, 1.1 equiv.) in 20 mL of anhydrous THF under an argon atmosphere was cooled to 0 °C on an ice bath. Then 1.6 M *n*-BuLi in hexanes (1.7 mL, 2.69 mmol, 1.1 equiv.) was added dropwise. The reaction mixture was stirred for 20 min at 0 °C. Then, the corresponding ketone was (630 mg, 2.45 mmol, 1.0 equiv. in 4.0 mL of anhydrous THF) was added dropwise. The ice bath was removed, and the reaction mixture was stirred at room temperature overnight. The reaction was quenched with 10 mL saturated ammonium chloride solution and extracted with diethyl ether (3 x 8 mL). The collected organic phase was washed with brine and dried over $MgSO_4$. The mixture was concentrated *in vacuo*. Purification by flash column chromatography (pentane/EtOAc = 95/5) afforded a white solid (170 mg, 0.66 mmol, 28%).

^{1}H NMR: (300 MHz, $CDCl_3$) δ 7.65 – 7.56 (m, 4H), 7.49 – 7.33 (m, 10H), 5.52 (dd, *J* = 15.5 Hz, 1.2 Hz, 2H). **^{13}C NMR**: (76 MHz, $CDCl_3$) δ 149.6, 141.4, 140.7, 140.5, 140.4, 128.8 (2C), 128.6 (2C), 128.3 (2C), 128.2 (2C), 127.8, 127.3, 127.0 (2C), 126.9 (2C), 114.3.
These data are in agreement with those reported previously in the literature.[13]

4,4'-(ethene-1,1-diyl)bis(fluorobenzene) (1u)

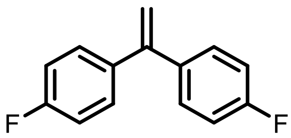

Wittig reaction was performed according to the previously reported procedure.[2] A suspension of methyltriphenylphosphonium bromide (930 mg, 2.20 mmol, 1.1 equiv.) in 20 mL of anhydrous THF under an argon atmosphere was cooled to 0 °C on an ice bath. Then 1.6 M *n*-BuLi in hexanes (1.4 mL, 2.20 mmol, 1.1 equiv.) was added dropwise. The reaction mixture was stirred for 20 min at 0 °C. Then, the corresponding ketone was (436 mg, 2.00 mmol, 1.0 equiv. in 4.0 mL of anhydrous THF) was added dropwise. The ice bath was removed, and the reaction mixture was stirred at room temperature overnight. The reaction was quenched with 10 mL saturated ammonium chloride solution and extracted with diethyl ether (3 x 8 mL). The collected organic phase was washed with brine and dried over $MgSO_4$. The mixture was concentrated *in vacuo*. Purification by flash column chromatography (pentane/EtOAc = 95/5) afforded a white solid (420 mg, 1.94 mmol, 97%).
^{1}H NMR (300 MHz, $CDCl_3$): δ 7.31 – 7.24 (m, 4H), 7.05 – 6.97 (m, 4H), 5.38 (s, 2H). **^{13}C NMR** (76 MHz, $CDCl_3$): δ 162.6 (d, *J* = 246.7 Hz), 148.1, 137.4 (d, *J* = 3.6 Hz), 129.8 (d, *J* = 7.9 Hz, 2C), 115.2 (d, *J* = 21.3 Hz, 2C), 114.1.**^{19}F NMR** (283 MHz, $CDCl_3$): δ -114.8.
These data are in agreement with those reported previously in the literature.[14]

4,4'-(ethene-1,1-diyl)bis(chlorobenzene) (1v)

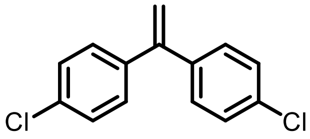

Wittig reaction was performed according to the previously reported procedure.[2]A suspension of methyltriphenylphosphonium bromide (1.40 g, 3.91 mmol, 1.1 equiv.) in 20 mL of anhydrous THF under an argon atmosphere was cooled to 0 °C on an ice bath. Then 1.6 M *n*-BuLi in hexanes (1.6 mL, 3.91 mmol, 1.1 equiv.) was added dropwise. The reaction mixture was stirred for 20 min at 0 °C. Then, the corresponding ketone was (890 mg, 3.56 mmol, 1.0 equiv. in 4.0 mL of anhydrous THF) was added dropwise. The ice bath was removed, and the reaction mixture was stirred at room temperature overnight. The reaction was quenched with 10 mL saturated ammonium chloride solution and extracted with diethyl ether (3 x 8 mL). The collected organic phase was washed with brine and

dried over $MgSO_4$. The mixture was concentrated *in vacuo*. Purification by flash column chromatography (pentane/EtOAc = 95/5) afforded as a pale yellow oil (570 mg, 2.29 mmol, 66%).

^{1}H NMR (300 MHz, $CDCl_3$): δ 7.34 – 7.27 (m, 4H), 7.27 – 7.24 (m, 4H), 5.45 (s, 2H). **^{13}C NMR** (75 MHz, $CDCl_3$): δ 148.1, 139.1, 134.3, 129.5, 126.1, 115.8.
These data are in agreement with those reported previously in the literature.[14]

Prop-1-ene-1,1-diyldibenzene (1w)

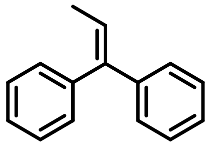

Wittig reaction was performed according to the previously reported procedure.[2] A suspension of ethyltriphenylphosphonium bromide (1.45 g, 3.91 mmol, 1.1 equiv.) in 20 mL of anhydrous THF under an argon atmosphere was cooled to 0 °C on an ice bath. Then 1.6 M *n*-BuLi in hexanes (1.6 mL, 3.91 mmol, 1.1 equiv.) was added dropwise. The reaction mixture was stirred for 20 min at 0 °C. Then, the corresponding ketone was (650 mg, 3.56 mmol, 1.0 equiv. in 4.0 mL of anhydrous THF) was added dropwise. The ice bath was removed, and the reaction mixture was stirred at room temperature overnight. After this time, the reaction was quenched with 10 mL saturated ammonium chloride solution and extracted with diethyl ether (3 x 8 mL). The collected organic phase was washed with brine and dried over $MgSO_4$. The mixture was concentrated *in vacuo*. Purification by flash column chromatography (pentane/EtOAc = 99/1) afforded as a white solid (574.5 mg, 2.96 mmol, 66%).

^{1}H NMR (300 MHz, $CDCl_3$): δ 7.39 – 7.15 (m, 10H), 6.18 (q, *J* = 7.0 Hz, 1H), 1.78 (d, *J* = 7.0 Hz, 3H). **^{13}C NMR** (75 MHz, $CDCl_3$): δ 142.8, 142.3, 140.3, 131.2, 128.0, 127.8, 127.2, 126.8, 126.8, 124.3, 15.6.
These data are in agreement with those reported previously in the literature.[15]

2-(1-phenylvinyl)thiophene (1x)

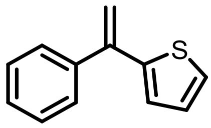

Wittig reaction was performed according to the previously reported procedure.[2]A suspension of methyltriphenylphosphonium bromide (1.05 g, 2.92 mmol, 1.1 equiv.) in 20 mL of anhydrous THF under an argon atmosphere was cooled to 0 °C on an ice bath. Then 1.6 M *n*-BuLi in hexanes (1.8 mL, 2.92 mmol, 1.1 equiv.) was added dropwise. The reaction mixture was stirred for 20 min at 0 °C. Then, the corresponding ketone was (500 mg, 2.65 mmol, 1.0 equiv. in 4.0 mL of anhydrous THF) was added dropwise. The ice bath was removed, and the reaction mixture was stirred at room temperature overnight. The reaction was quenched with 10 mL saturated ammonium chloride solution and extracted with diethyl ether (3 x 8 mL). The collected organic phase was washed with brine and dried over $MgSO_4$. The mixture was concentrated *in vacuo*. Purification by flash column chromatography (pentane/EtOAc = 95/5) afforded a white solid (310 mg, 1.66 mmol 63%).

^{1}H NMR (300 MHz, $CDCl_3$): δ 7.47 – 7.35 (m, 5H), 7.25 (dd, *J* = 5.1, 1.2 Hz, 1H), 6.99 (dd, *J* = 5.1, 3.6 Hz, 1H), 6.92 (dd, *J* = 3.6, 1.2 Hz, 1H), 5.60 (d, *J* = 0.9 Hz, 1H), 5.26 (d, *J* = 0.9 Hz, 1H). **^{13}C NMR**: (75 MHz, $CDCl_3$): δ 144.8, 143.4, 141.0, 128.3 (2C), 128.2 (2C), 128.0, 127.3, 126.4, 125.0, 113.6.
These data are in agreement with those reported previously in the literature.[16]

5-methylene-10,11-dihydro-5*H*-dibenzo[*a*,*d*][7]annulene (1y)

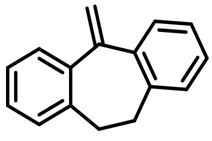

Wittig reaction was performed according to the previously reported procedure.[2] A suspension of methyltriphenylphosphonium bromide (960 mg, 2.69 mmol, 1.1 equiv.) in 20 mL of anhydrous THF under an argon atmosphere was cooled to 0 °C on an ice bath. Then 1.6 M *n*-BuLi in hexanes (1.7 mL, 2.69 mmol, 1.1 equiv.) was added dropwise. The reaction mixture was stirred for 20 min at 0 °C. Then, the corresponding ketone was (510 g, 2.45 mmol, 1.0 equiv. in 4.0 mL of anhydrous THF) was added dropwise. The ice bath was removed, and the reaction mixture was stirred at room temperature

overnight. The reaction was quenched with 10 mL saturated ammonium chloride solution and extracted with diethyl ether (3 x 8 mL). The collected organic phase was washed with brine and dried over $MgSO_4$. The mixture was concentrated *in vacuo*. Purification by flash column chromatography (pentane/EtOAc = 95/5) afforded a white solid (28 mg, 1.36 mmol, 55%).

^{1}H NMR (300 MHz, $CDCl_3$): δ 7.38 – 7.35 (m, 2H), 7.25 – 7.12 (m, 6H), 5.43 (s, 2H), 3.16 (s, 4H). **^{13}C NMR** (76 MHz, $CDCl_3$): δ 151.8, 141.2 (2C), 138.3 (2C), 128.9 (2C), 128.1 (2C), 127.7 (2C), 126.2 (2C), 117.5, 33.2 (2C).
These data are in agreement with those reported previously in the literature.[17]

Buta-1,3-diene-1,1-diyldibenzene (1z)

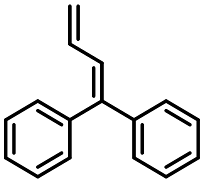

Wittig reaction was performed according to the previously reported procedure.[2] A suspension of allyltriphenylphosphonium bromide (1.49 g, 3.91 mmol, 1.1 equiv.) in 20 mL of anhydrous THF under an argon atmosphere was cooled to 0 °C on an ice bath. Then 1.6 M *n*-BuLi in hexanes (1.6 mL, 3.91 mmol, 1.1 equiv.) was added dropwise. The reaction mixture was stirred for 20 min at 0 °C. Then, the corresponding ketone was (650 mg, 3.56 mmol, 1.0 equiv. in 4.0 mL of anhydrous THF) was added dropwise. The ice bath was removed, and the reaction mixture was stirred at room temperature overnight. The reaction was quenched with 10 mL saturated ammonium chloride solution and extracted with diethyl ether (3 x 8 mL). The collected organic phase was washed with brine and dried over $MgSO_4$. The mixture was concentrated in vacuo. Purification by flash column chromatography (pentane/EtOAc = 99/1) afforded a colorless liquid (380 mg, 1.84 mmol, 52%).

^{1}H NMR (300 MHz, $CDCl_3$): δ 7.42 – 7.33 (m, 3H), 7.32 – 7.26 (m, 5H), 7.24 – 7.19 (m, 2H), 6.72 (d, *J* = 11.0 Hz, 1H), 6.45 (ddd, *J* = 16.8, 11.0, 10.1 Hz, 1H), 5.40 (ddd, *J* = 16.8, 1.9, 0.8 Hz, 1H), 5.13 (ddd, *J* = 10.1, 1.9, 0.8 Hz, 1H). **^{13}C NMR** (75 MHz, $CDCl_3$): δ 143.0, 141.8, 139.4, 134.7, 130.2, 128.3, 128.0, 127.9, 127.4, 127.3, 127.0, 118.4.
These data are in agreement with those reported previously in the literature.[18]

2-(1-phenylvinyl)aniline (1aa)

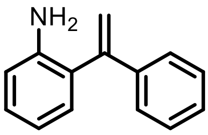

Wittig reaction was performed according to the previously reported procedure.[2] A suspension of methyltriphenylphosphonium bromide (2.36 g, 6.60 mmol, 3.3 equiv.) in 34 mL of anhydrous THF under an argon atmosphere was cooled to 0 °C on an ice bath. Then 1.6 M *n*-BuLi in hexanes (2.9 mL, 6.60 mmol, 3.3 equiv.) was added dropwise. The reaction mixture was stirred for 20 min at 0 °C. Then, the corresponding ketone was (395 mg, 2.00 mmol, 1.0 equiv. in 2.5 mL of anhydrous THF) was added dropwise. The ice bath was removed, and the reaction mixture was stirred at room temperature overnight. The reaction was quenched with 10 mL saturated ammonium chloride solution and extracted with diethyl ether (3 x 8 mL). The collected organic phase was washed with brine and dried over $MgSO_4$. The mixture was concentrated *in vacuo*. Purification by flash column chromatography (pentane/EtOAc = 92/8) afforded a yellow colored solid (210 mg, 1.08 mmol, 54%).

^{1}H NMR (300 MHz, $CDCl_3$): δ 7.43 – 7.27 (m, 5H), 7.20 – 7.10 (m, 2H), 6.80 (td, *J* = 7.5, 1.2 Hz, 1H), 6.70 (ddd, *J* = 7.9, 1.2, 0.5 Hz, 1H), 5.81 (d, *J* = 1.5 Hz, 1H), 5.37 (d, *J* = 1.4 Hz, 1H), 3.56 (s, 2H). **^{13}C NMR** (75 MHz, $CDCl_3$): δ 147.3, 143.9, 139.8, 130.7, 128.9, 128.4, 128.0, 127.2, 126.5,118.3, 116.1, 115.8.
These data are in agreement with those reported previously in the literature.[19]

2-(1-(4-chlorophenyl)vinyl)aniline (1ab)

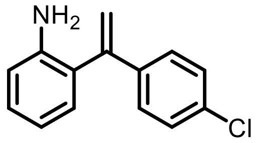

Wittig reaction was performed according to the previously reported procedure.[2] A suspension of methyltriphenylphosphonium bromide (1.18 g, 3.30 mmol, 3.3 equiv.) in 17 mL of anhydrous THF under an argon atmosphere was cooled to 0 °C on an ice bath. Then 1.6 M *n*-BuLi in hexanes (1.45 mL, 3.30 mmol, 3.3 equiv.) was added dropwise. The reaction mixture was stirred for 20 min at 0 °C. Then, the corresponding ketone was (232 mg, 1.00 mmol, 1.0 equiv. in 1.25 mL of anhydrous THF) was added dropwise. The ice bath was removed, and the reaction mixture was stirred at room temperature overnight. The reaction was quenched with 10 mL saturated ammonium chloride solution and extracted with diethyl ether (3 x 8 mL). The collected organic phase was washed with brine and dried over $MgSO_4$. The mixture was concentrated *in vacuo*. Purification by flash column chromatography (pentane/EtOAc = 92/8) afforded a yellow colored solid (97 mg, 0.42 mmol, 42%).

^{1}H NMR (300 MHz, $CDCl_3$): δ 7.33 – 7.27 (m, 4H), 7.17 (ddd, *J* = 8.0, 7.3, 1.6 Hz, 1H), 7.08 (ddd, *J* = 7.6, 1.6, 0.4 Hz, 1H), 6.79 (td, *J* = 7.4, 1.2 Hz, 1H), 6.70 (ddd, *J* = 8.0, 1.2, 0.4 Hz, 1H), 5.79 (d, *J* = 1.3 Hz, 1H), 5.37 (d, *J* = 1.3 Hz, 1H), 3.55 (s, 2H). **^{13}C NMR** (75 MHz, $CDCl_3$): δ 146.5, 143.9, 138.4, 133.9, 130.7, 129.3, 128.6, 127.9, 126.6, 118.3, 116.6, 115.8.
These data are in agreement with those reported previously in the literature.[20]

5-chloro-2-(1-phenylvinyl)aniline (1ac)

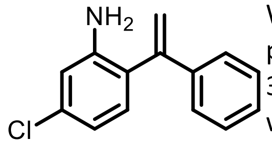

Wittig reaction was performed according to the previously reported procedure.[2] A suspension of methyltriphenylphosphonium bromide (1.18 g, 3.30 mmol, 3.3 equiv.) in 17 mL of anhydrous THF under an argon atmosphere was cooled to 0 °C on an ice bath. Then 1.6 M *n*-BuLi in hexanes (1.45 mL, 3.3 mmol, 3.3 equiv.) was added dropwise. The reaction mixture was stirred for 20 min at 0 °C. Then, the corresponding ketone was (232 mg, 1.00 mmol, 1.0 equiv. in 1.25 mL of anhydrous THF) was added dropwise. The ice bath was removed, and the reaction mixture was stirred at room temperature overnight. The reaction was quenched with 10 mL saturated ammonium chloride solution and extracted with diethyl ether (3 x 8 mL). The collected organic phase was washed with brine and dried over $MgSO_4$. The mixture was concentrated *in vacuo*. Purification by flash column chromatography (pentane/EtOAc = 92/8) afforded a yellow colored solid (120 mg, 0.52 mmol, 52%).

^{1}H NMR (300 MHz, $CDCl_3$): δ 7.39 – 7.29 (m, 5H), 7.03 (dd, *J* = 8.0, 0.3 Hz, 1H), 6.75 (dd, *J* = 8.1, 2.1 Hz, 1H), 6.69 (dd, *J* = 2.1, 0.3 Hz, 1H), 5.80 (d, *J* = 1.3 Hz, 1H), 5.35 (d, *J* = 1.4 Hz, 1H), 3.63 (s, 2H). **^{13}C NMR** (75 MHz, $CDCl_3$): δ 145.9, 143.6, 138.0, 133.7, 129.8, 129.2, 128.5, 127.6, 126.4, 118.1, 116.5, 115.6.
These data are in agreement with those reported previously in the literature.[21]

3-fluoro-4-vinyl-1,1'-biphenyl (1ad)

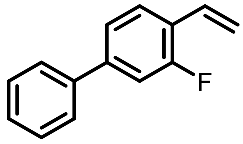

Wittig reaction was performed according to the previously reported procedure.[2] A suspension of methyltriphenylphosphonium bromide (1.07 g, 3.00 mmol, 1.2 equiv.) in 20 mL of anhydrous THF under an argon atmosphere was cooled to 0 °C on an ice bath. Then 1.6 M *n*-BuLi in hexanes (1.9 mL, 3.00 mmol, 1.2 equiv.) was added dropwise. The reaction mixture was stirred for 20 min at 0 °C. Then, the corresponding ketone was (500 mg, 2.50 mmol, 1.0 equiv. in 4.0 mL of anhydrous THF) was added dropwise. The ice bath was removed, and the reaction mixture was stirred at room temperature overnight. The reaction was quenched with 10 mL saturated ammonium chloride solution and extracted with diethyl ether (3 x 8 mL). The collected organic phase was washed with brine and dried over $MgSO_4$. The mixture was concentrated *in vacuo*. Purification by flash column chromatography (pentane/EtOAc = 95/5) afforded a white solid (380 mg, 1.92 mmol, 77%).

^{1}**H NMR** (300 MHz, $CDCl_3$): δ 7.59 – 7.54 (m, 2H), 7.48 – 7.35 (m, 4H), 7.27 – 7.20 (m, 2H), 6.72 (ddd, *J* = 17.4, 10.8, 0.7 Hz, 1H), 5.81 (dd, *J* = 17.4, 0.7 Hz, 1H), 5.34 (dt, *J* = 10.8, 0.7 Hz, 1H).
^{13}**C NMR** (75 MHz, $CDCl_3$): δ 160.0 (d, *J* = 250.2 Hz), 138.9 (d, *J* = 8.1 Hz), 135.6 (d, *J* = 1.4 Hz), 135.5 (d, *J* = 2.1 Hz), 130.7 (d, *J* = 4.0 Hz), 128.9 (d, *J* = 3.1 Hz, 2C), 128.5 (2C),128.3 (d, *J* = 13.7 Hz)127.7, 122.4 (d, *J* = 3.2 Hz), 115.2, 113.4 (d, *J* = 23.5 Hz). ^{19}**F NMR** (283 MHz, $CDCl_3$): δ -118.8.
These data are in agreement with those reported previously in the literature.[22]

Isopropyl 2-(4-(4-chlorobenzoyl)phenoxy)-2-methylpropanoate (1ae')

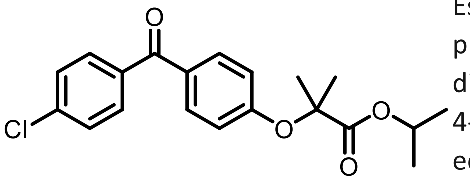

Esterification was done according to the previously reported procedure. [6] Fenofibric acid (2.62g, 8.20 mmol, 1.0 equiv.), dicyclohexylcarbodiimide (DCC, 1.85 g, 8.95 mmol, 1.12 equiv.), 4-(dimethylamino)pyridine (DMAP, 0.123 g, 1.0 mmol, 0.12 equiv.), and *t*-butanol (0.7 mL, 9.15 mmol, 1.15 equiv.) were stirred in 15 mL of dry CH_2Cl_2 until no further product formation was observed by thin-layer chromatography (approximately 16 h). The mixture was filtered, the precipitate was rinsed with CH_2Cl_2, and the combined filtrates were evaporated. The resulting solid was purified by flash column chromatography (pentane/EtOAc = 95/5) on silica gel to get a white solid (2.58 g, 7.15 mmol, 86%).

^{1} **H NMR** ($CDCl_3$, 300 MHz): δ 7.76 – 7.67 (m, 4H), 7.45 (d, *J* = 8.7 Hz, 2H), 6.86 (d, *J* = 9.0 Hz, 2H), 5.09 (hept, *J* = 6.3 Hz, 1H), 1.66 (s, 6H), 1.20 (d, *J* = 6.3 Hz, 6H). ^{13} **C NMR** ($CDCl_3$, 75 MHz): δ 194.5, 173.4, 159.7, 138.1, 137.0, 132.5, 131.7, 130.9, 128.6, 117.4, 79.5, 69.2, 25.9, 21.3.
These data are in agreement with those reported previously in the literature.[11]

Isopropyl 2-(4-(1-(4-chlorophenyl)vinyl)phenoxy)-2-methylpropanoate (1ae)

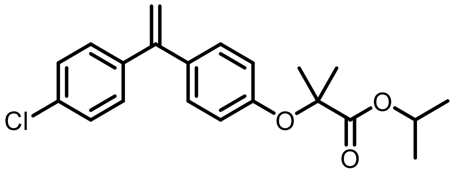

Wittig reaction was performed according to the previously reported procedure.[2] A suspension of methyltriphenylphosphonium bromide (1.4 g, 3.91 mmol, 1.1 equiv.) in 20 mL of anhydrous THF under an argon atmosphere was cooled to 0 °C on an ice bath. Then 1.6 M *n*-BuLi in hexanes (1.6 mL, 3.91 mmol, 1.1 equiv.) was added dropwise. The reaction mixture was stirred for 20 min at 0 °C. Then, the corresponding ketone (1ae') was (1.28 g, 3.56 mmol, 1.0 equiv. in 4.0 mL of anhydrous THF) was added dropwise. The ice bath was removed, and the reaction mixture was stirred at room temperature overnight. The reaction was quenched with 10 mL saturated ammonium chloride solution and extracted with diethyl ether (3 x 8 mL). The collected organic phase was washed with brine and dried over $MgSO_4$. The mixture was concentrated *in vacuo*. Purification by flash column chromatography (Pentane/EtOAc = 94/6) afforded a white solid (980 g, 2.73 mmol, 77%).

^{1}**H NMR** (300 MHz, $CDCl_3$): δ 7.32 – 7.22 (m, 4H), 7.21 – 7.15 (m, 2H), 6.82 – 6.77 (m, 2H), 5.40 (d, *J* = 1.2 Hz, 1H), 5.33 (d, *J* = 1.2 Hz, 1H), 5.09 (hept, *J* = 6.3 Hz, 1H), 1.61 (s, 6H), 1.23 (d, *J* = 6.3 Hz, 6H).
^{13}**C NMR** (75 MHz, $CDCl_3$): δ 173.6, 155.4, 148.3, 140.2, 134.4, 133.5, 129.6, 128.8, 128.3, 118.4, 113.6, 79.1, 68.9, 25.4, 21.5. I**R (neat)**: ν 3082, 3047, 2986, 2937, 1716, 1151, 1094, 829, 699 cm^{-1}.
HRMS (ESI) (*m/z*): $[M+Na]^+$ calculated for $[C_{21}H_{23}ClO_3Na]^+$: 381.1228, found: 381.1229.

Ethyl 2-(3-benzoylphenyl)propanoate (1af')

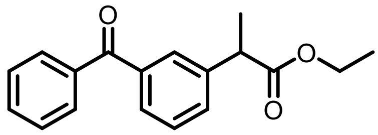

Esterification was done according to the previously reported procedure.[6] Ketoprofen (1.05 g, 4.10 mmol, 1.0 equiv), dicyclohexylcarbodiimide (DCC, 925 mg, 4.48 mmol, 1.12 equiv.), 4-(dimethylamino)pyridine (DMAP, 61 mg, 0.05 mmol, 0.12 equiv.), and ethanol (0.27 mL, 4.58 mmol, 1.15 equiv.) were stirred in 15 mL of dry CH_2Cl_2 until no further product formation was observed by thin-layer chromatography (approximately 16 h). The mixture was filtered, the precipitate was rinsed with CH_2Cl_2, and the combined filtrates were evaporated. The resulting solid was purified by flash column chromatography (pentane/EtOAc = 95/5) on silica gel to get a white solid (660 mg, 2.34 mmol, 57%).

^{1}H NMR (300 MHz, $CDCl_3$) δ 7.85 – 7.77 (m, 2H), 7.75 (tt, *J* = 1.8, 0.6 Hz, 1H), 7.68 (ddd, *J* = 7.6, 1.7, 1.3 Hz, 1H), 7.63 – 7.52 (m, 2H), 7.52 – 7.39 (m, 3H), 4.14 (qd, *J* = 7.1, 4.4 Hz, 2H), 3.78 (q, *J* = 7.2 Hz, 1H), 1.53 (d, *J* = 7.2 Hz, 3H), 1.22 (t, *J* = 7.1 Hz, 3H). **^{13}C NMR** (75 MHz, $CDCl_3$) δ 196.5, 174.1, 140.9, 137.8, 137.5, 132.5, 131.5, 130.1 (2C), 129.2, 128.9, 128.5, 128.3 (2C), 60.9, 45.4, 18.5, 14.1.

Ethyl 2-(3-(1-phenylvinyl)phenyl)propanoate (1af)

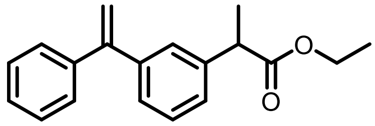

Wittig reaction was performed according to the previously reported procedure.[2] A suspension of methyltriphenylphosphonium bromide (1.32 g, 3.71 mmol, 1.1 equiv.) in 19 mL of anhydrous THF under an argon atmosphere was cooled to 0 °C on an ice bath. Then 1.6 M *n*-BuLi in hexanes (1.7 mL, 3.71 mmol, 1.1 equiv.) was added dropwise. The reaction mixture was stirred for 20 min at 0 °C. Then, the corresponding ketone was (951 mg, 3.34 mmol, 1.0 equiv. in 4.3 mL of anhydrous THF) was added dropwise. The ice bath was removed, and the reaction mixture was stirred at room temperature overnight. The reaction was quenched with 10 mL saturated ammonium chloride solution and extracted with diethyl ether (3 x 8 mL). The collected organic phase was washed with brine and dried over $MgSO_4$. The mixture was concentrated *in vacuo*. Purification by flash column chromatography (pentane/EtOAc = 97/3) afforded a colorless oil (660 mg, 2.35 mmol, 68%).

^{1}H NMR (300 MHz, $CDCl_3$) δ 7.38 – 7.31 (m, 4H), 7.31 – 7.26 (m, 3H), 7.25 – 7.19 (m, 1H), 5.48 – 5.45 (m, 2H), 4.21 – 4.04 (m, 2H), 3.70 (q, *J* = 7.2 Hz, 1H), 1.49 (d, *J* = 7.2 Hz, 3H), 1.20 (t, *J* = 7.1 Hz, 3H). **^{13}C NMR** (75 MHz, $CDCl_3$) δ 174.4, 149.8, 141.7, 141.3, 140.6, 128.4 (2C), 128.2 (2C), 128.1, 127.7, 127.5, 127.1, 126.7, 114.4, 60.7, 45.5, 18.6, 14.1. **IR (neat)**: ν 3043, 2982, 2939, 1723, 1244, 898, 699 cm^{-1}. **HRMS (ESI) (*m/z*)**: $[M+Na]^+$ calculated for $[C_{19}H_{20}O_2Na]^+$: 303.1355, found: 303.1357.

(2a*S*,5'*S*,6a*S*,6b*S*,8a*S*,8b*R*,9*S*,10*S*,11a*S*,12a*S*,12b*R*)-5',6a,8a,9-tetramethyldocosahydro-spiro[naphtho[2',1':4,5]indeno[2,1-*b*]furan-10,2'-pyran]-4-yl 4-vinylbenzoate (1ag)

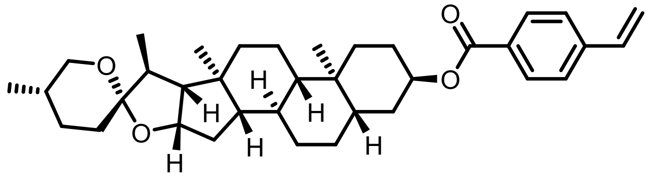

Esterification was done according to the previously reported procedure.[23] To a stirred solution of 4-vinyl benzoic acid (225 mg, 1.50 mmol, 1.0 equiv.) and dicyclohexylcarbodiimide (DCC, 375 mg, 1.80 mmol, 1.2 equiv.) in 5 mL CH_2Cl_2 were added 4-dimethylaminepyridin (DMAP, 220 mg, 1.80 mmol, 1.2 equiv.) and tigogenin (625 mg, 1.50 mmol, 1.0 equiv.). The reaction mixture was stirred at room temperature for 24 h. After filtration, the filtrate was concentrated *in vacuo* and the residue was purified by flash column chromatography (pentane/EtOAc = 5/1) to afford the product as a white solid in (570 mg, 1.04 mmol, 69%).

^{1}H NMR (300 MHz, $CDCl_3$): δ 7.98 (d, *J* = 8.4 Hz, 2H), 7.44 (d, *J* = 8.2 Hz, 2H), 6.74 (dd, *J* = 17.6, 10.9 Hz, 1H), 5.85 (d, *J* = 17.6 Hz, 1H), 5.37 (d, *J* = 10.9 Hz, 1H), 4.98 – 4.87 (m, 1H), 4.39 (q, *J* = 7.4 Hz, 1H), 4.03

(d, *J* = 7.8 Hz, 1H), 3.47 (dd, *J* = 11.2, 4.1 Hz, 2H), 3.37 (t, *J* = 11.0 Hz, 1H), 2.01 – 1.90 (m, 3H), 1.86 (t, *J* = 6.8 Hz, 1H), 1.76 (dd, *J* = 6.4, 2.3 Hz, 2H), 1.69 (d, *J* = 9.0 Hz, 4H), 1.54 – 1.47 (m, 2H), 1.33 – 1.23 (m, 6H), 1.17 – 1.05 (m, 6H), 0.96 (dd, *J* = 7.0, 3.2 Hz, 4H), 0.89 (s, 3H), 0.77 (t, *J* = 4.2 Hz, 6H).
^{13}C NMR (75 MHz, $CDCl_3$): δ 165.8, 141.7, 136.1, 129.8, 127.0, 126.2, 115.8, 109.2, 80.8, 74.2, 66.8, 62.2, 56.2, 54.2, 44.7, 41.6, 40.6, 40.0, 36.7, 35.6, 35.1, 34.1, 32.3, 31.4, 30.8, 30.3, 26.2, 21.0, 17.1, 16.5, 14.5, 12.3.

These data are in agreement with those reported previously in the literature.[23]

(8*R*,9*S*,13*S*,14*S*)-13-methyl-17-oxo-7,8,9,11,12,13,14,15,16,17-decahydro-6*H*-cyclopenta[*a*]phenanthren-3-yl 4-vinylbenzoate (1ah)

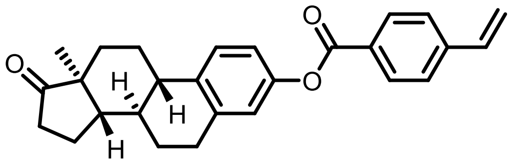

Esterification was done according to the previously reported procedure.[23] To a stirred solution of 4-vinyl benzoic acid (225 mg, 1.50 mmol, 1.0 equiv.) and DCC (375 mg, 1.80 mmol, 1.2 equiv.) in 5 mL were added DMAP (220 mg, 1.80 mmol, 1.2 equiv.) and estrone (405 mg, 1.50 mmol, 1.0 equiv.). The reaction mixture was stirred at room temperature for 24 h. After filtration, the filtrate was concentrated *in vacuo* and the residue was purified by flash column chromatography (pentane/EtOAc = 95/5 to 84/16) to afford the product as a white solid in (450 mg, 1.12 mmol, 75%).

^{1}H NMR (300 MHz, $CDCl_3$): δ 8.14 (d, *J* = 8.4 Hz, 2H), 7.52 (d, *J* = 8.1 Hz, 2H), 7.33 (m, 1H), 7.02 – 6.92 (m, 2H), 6.79 (dd, *J* = 17.6, 10.9 Hz, 1H), 5.91 (dd, *J* = 17.6, 0.7 Hz, 1H), 5.43 (dd, J = 10.9, 0.7 Hz, 1H), 2.94 (dd, *J* = 8.7, 4.1 Hz, 2H), 2.48 (tdd, *J* = 16.1, 8.7, 3.9 Hz, 2H), 2.31 – 2.28 (m, 1H), 2.21 – 1.95 (m, 4H), 1.68 – 1.58 (m, 2H), 1.57 – 1.45 (m, 4H), 0.93 (s, 3H). **^{13}C NMR** (75 MHz, $CDCl_3$): δ 165.2, 148.9, 142.5, 138.1, 137.4, 136.0, 130.5, 128.7, 126.5, 126.3, 121.7, 118.9, 116.9, 50.5, 48.0, 44.2, 38.0, 35.9, 31.6, 29.5, 26.4, 25.8, 21.6, 13.9.
These data are in agreement with those reported previously in the literature.[24]

***N*-(2-(1*H*-indol-3-yl)ethyl)-4-vinylbenzamide (1ai)**

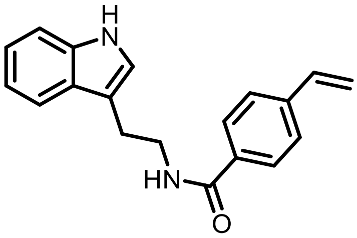

Coupling was performed according to the previously reported procedure.[25] To a mixture of tryptamine hydrochloride (201 mg, 1.00 mmol, 1.1 equiv.), 4-vinylbenzoic acid (135 mg, 0.91 mmol, 1.0 equiv.) and 2-chloro-1-methylpyridinium iodide (318 mg, 1.18 mmol, 1.3 equiv.) in 5 mL CH_2Cl_2 at 0 °C under argon, was added triethylamine (0.51 mL, 3.64 mmol, 4.0 equiv.) dropwise and the reaction mixture allowed to warm slowly to room temperature. Once the reaction was completed (by TLC) the organic phase was washed with 1.0 M aqueous HCl, followed by saturated aqueous Na_2CO_3 and dried over $MgSO_4$. The organic solvents were removed *in vacuo* and the desired product was purified by flash column chromatography on silica gel (pentane/EtOAc = 50/50) to afford a white solid (250 mg,0.86 mmol, 94%).

^{1}H NMR (300 MHz, $CDCl_3$): δ 8.22 (br, 1H), 7.67 – 7.61 (m, 3H), 7.41 – 7.37 (m, 3H), 7.25 – 7.19 (m, 1H), 7.16 – 7.11 (m, 1H), 7.06 (d, *J* = 2.3 Hz, 1H), 6.71 (dd, *J* = 17.7, 10.8 Hz, 1H), 6.25 (br, 1H), 5.81 (dd, *J* = 17.7, 0.8 Hz, 1H), 5.34 (dd, *J* = 10.8, 0.8 Hz, 1H), 3.80 (td, *J* = 6.7, 5.8 Hz, 2H), 3.10 (td, *J* = 6.7, 0.8 Hz, 2H). **^{13}C NMR** (75 MHz, $CDCl_3$): δ 167.1, 140.5, 136.4, 135.9 (2C), 133.7, 127.3, 127.1 (2C), 126.2 (2C), 122.3, 122.1, 119.5, 118.7, 115.8, 113.0, 111.3, 40.3, 25.3. **IR (neat)**: ν 3265, 2926, 1607, 1556, 1503, 1455, 1427, 1312, 861, 740, 688 cm^{-1}. **HRMS (ESI) (*m/z*)**: $[M+Na]^+$ calculated for $[C_{19}H_{18}N_2NaO]^+$: 313.1311, found: 313.1313.

N-(((1S,4aR,10aS)-7-isopropyl-1,4a-dimethyl-1,2,3,4,4a,9,10,10a-octahydrophenanthren-1-yl)methyl)-4-vinylbenzamide (1aj)

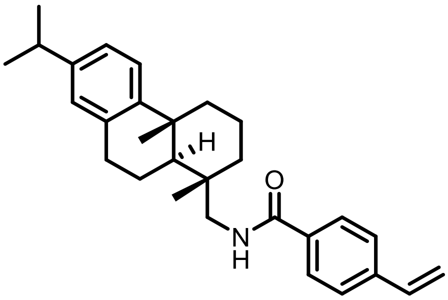

Coupling was performed according to the previously reported procedure.[25] To a mixture of dehydroabietylamine (317 mg, 1.00 mmol, 1.1 equiv.), 4-vinylbenzoic acid (135 mg, 0.91 mmol, 1.0 equiv.) and 2-chloro-1-methylpyridinium iodide (318 mg, 1.18 mmol, 1.3 equiv.) in 5 mL CH_2Cl_2 at 0 °C under argon, was added triethylamine (0.51 mL, 3.64 mmol, 4.0 equiv.) dropwise and the reaction mixture allowed to warm slowly to room temperature. Once the reaction was completed (by TLC) the organic phase was washed with 1.0 M aqueous HCl, followed by saturated aqueous Na_2CO_3 and dried over $MgSO_4$. The organic solvents were removed *in vacu*o and the desired product was purified by flash column chromatography on silica gel (pentane/EtOAc = 80/20) to afford a white solid (290 mg, 0.70 mmol, 77%).

^{1}H NMR (300 MHz, $CDCl_3$): δ 7.70 (d, *J* = 8.4 Hz, 2H), 7.44 (d, *J* = 8.4 Hz, 2H), 7.17 (d, *J* = 8.2 Hz, 1H), 6.99 (dd, *J* = 8.2, 2.0 Hz, 1H), 6.89 (d, *J* = 2.0 Hz, 1H), 6.73 (dd, *J* = 17.6, 10.9 Hz, 1H), 6.12 (t, *J* = 6.5 Hz, 1H), 5.82 (dd, *J* = 17.6, 0.8 Hz, 1H), 5.34 (dd, *J* = 10.9, 0.8 Hz, 1H), 3.39 (qd, *J* = 13.7, 6.5 Hz, 2H), 2.98 – 2.75 (m, 3H), 2.33 – 2.29 (m, 1H), 1.98 (ddt, *J* = 13.3, 7.1, 2.1 Hz, 1H), 1.88 – 1.62 (m, 3H), 1.56 – 1.47 (m, 2H), 1.38 (dddd, *J* = 17.5, 13.3, 8.7, 4.1 Hz, 2H), 1.24 (s, 3H), 1.23 (s, 3H), 1.21 (s, 3H), 1.02 (s, 3H). **^{13}C NMR**: (75 MHz, $CDCl_3$) δ 167.2, 147.0, 145.6, 140.5, 135.9, 134.7, 133.9, 127.1 (2C), 126.9, 126.3 (2C), 124.2, 123.9, 115.8, 50.3, 45.8, 38.3, 37.7, 37.6, 36.4, 33.4, 30.4, 25.4, 23.9, 23.9, 19.1, 18.8, 18.6. **IR (neat)**: ν 3315, 2926, 1638, 1541, 1500, 1298, 855, 752, 630 cm^{-1}.
HRMS (ESI) (*m/z*): $[M+Na]^+$ calculated for $[C_{29}H_{37}NNaO]^+$: 438.2767, found: 438.2768.

1-(trifluoromethyl)-4-(2-vinylcyclopropyl)benzene (1an)

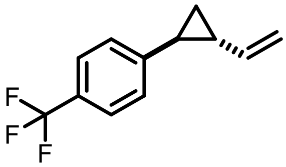

The literature procedure was followed.[26] An oven-dried round-bottom Schlenk flask was charged with methyltriphenylphosphonium bromide (1.32 g, 3.71 mmol, 1.2 equiv.) before it was evacuated and backfilled with argon for four times. It was suspended in anhydrous THF (0.1 M), cooled to 0 °C and *n*-BuLi (1.1 equiv., 1.6 M in hexane) was added dropwise and the reaction mixture stirred for 30 min at 0 °C. Then, a solution of the aldehyde (1.0 equiv. in 0.5 M in anhydrous THF) was added and the resulting mixture was stirred for 1 h at 0 °C followed by stirring at room temperature. The reaction was quenched by adding saturated aqueous ammonium chloride and organic phase was separated. The retained aqueous phase was extracted with CH_2Cl_2. The combined organic layers were dried over Na_2SO_4, filtered, and concentrated under reduced pressure. The crude vinylcyclopropane was purified by silica gel column chromatography (pentane, R_f = 0.5). The colourless oil was isolated (461 mg, 2.17 mmol, 41%) as diastereomeric mixture of *cis:trans*= 1:3.

^{1}H NMR (600 MHz, $CDCl_3$): δ 7.54 – 7.50 (m, 2H *cis* + 2H *trans*), 7.31 (d, *J* = 8.0 Hz, 2H, *cis*), 7.16 (d, *J* = 8.0 Hz, 2H, *trans*), 5.55 (dddd, *J* = 17.6, 9.0, 8.5, 1.8 Hz, 1H, *trans*), 5.17 –5.04 (m, 3H), 4.99 – 4.97 (m, 1H, *trans*), 4.91 (dt, *J* = 9.7, 1.6 Hz, 1H, *cis*), 2.38 (q, *J* = 8.5 Hz, 1H, *cis*), 2.00 – 1.92 (m, 1H *cis* + 1H *trans*), 1.74 (tt, *J* = 8.9, 5.0 Hz, 1H, *trans*), 1.35 – 1.31 (m, 1H, *cis*), 1.27 – 1.23 (m, 1H, *trans*), 1.22 – 1.17 (m, 1H, *trans*), 1.10 (q, *J* = 5.6 Hz, 1H, *cis*). **^{13}C NMR** (126 MHz, $CDCl_3$): δ 146.8, 143.3, 140.0, 137.3, 129.5, 128.3 (q, *J* = 39.3 Hz), 128.0 (q, *J* = 32.5 Hz), 126.0, 125.6 (d, *J* = 2.7 Hz), 125.4 (q, *J* = 4.4 Hz),

125.1 (q, J = 3.5 Hz), 123.4, 115.2, 113.4, 28.2, 25.2, 23.4, 23.2, 17.3, 12.0. **^{19}F NMR** (565 MHz, $CDCl_3$): δ – 62.3 (s, 3F, *trans*), – 62.3 (s, 3F, *cis*).

These data are in agreement with those reported previously in the literature.[26]

[(*E*)-3-(2-methylallyloxy)prop-1-enyl]-4-(trifluoromethyl)benzene (1ar)

Step 1:

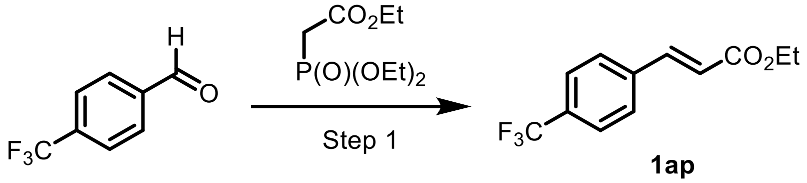

To a stirred suspension of NaH (55% in paraffin oil, 0.227 g, 5.20 mmol) in THF (4 mL) at room temperature was added a THF (10 mL) solution of ethyl 2-(diethoxyphosphoryl)acetate (1.08 g, 4.80 mmol). The mixture was stirred at room temperature for 30 min, and then a THF (4 mL) solution of 4-bromobenzaldehyde (0.74 g, 4.00 mmol) was added at 0 °C. The cold bath was removed, and the resulting mixture was stirred at room temperature for 2 h. The reaction was quenched by the addition of H_2O. The phases were separated, and the aqueous phase was extracted two additional times with Et_2O. The combined organic layers were washed with brine, dried over anhydrous $MgSO_4$, filtered, and concentrated. The residue was purified by a silica gel column chromatography (pentane/EtOAc = 97:3), which furnished ethyl ester (**1ap,** 0.84 g, 3.24 mmol, 81%) as a pale yellow oil.

^{1}H NMR (300 MHz, $CDCl_3$): δ 7.69 (d, J = 16.0 Hz, 1H), 7.63 (m, 4H), 6.50 (d, J = 16.0 Hz, 1H), 4.28 (q, J = 7.1 Hz, 2H), 1.34 (t, J = 7.1 Hz, 3H). **^{13}C NMR** (75 MHz, $CDCl_3$) δ 166.3, 142.6, 137.8, 131.7 (q, J = 32.6 Hz), 128.1, 125.8 (q, J = 3.8 Hz), 123.8 (q, J = 272.2 Hz), 119.8, 60.7, 14.2. **^{19}F NMR** (283 MHz, $CDCl_3$): δ –62.9.

These data are in agreement with those reported previously in the literature.[27]

Step 2:

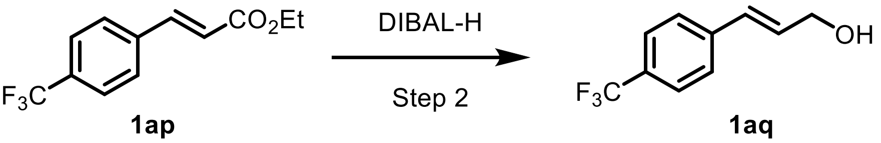

To a stirred solution of ester **1ap** (0.84 g, 3.24 mmol) in CH_2Cl_2 (6.3 mL) at –78 °C was added a 1.0 M solution of DIBAL in hexane (8.1 mL, 8.5 mmol). The resulting mixture was stirred at –78 °C for 2 h. The reaction was allowed to warm to 0 °C and quenched by the addition of 1 M aqueous HCl. The phases were separated, and the aqueous phase was extracted two additional times with CH_2Cl_2. The combined organic layers were washed with brine, dried over anhydrous $MgSO_4$, filtered, and concentrated. The crude allyl alcohol (**1aq**, 0.66 g) was used in the next step without further purification.

Step 3:

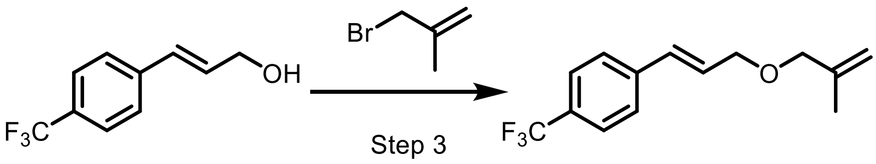

1aq **1ar**

To a stirred suspension of NaH (55% in paraffin oil, 0.175 g, 4.00 mmol) in THF (2 mL) at room temperature was added a THF (4 mL) solution of the above crude **1aq** (0.63 g). The mixture was stirred at room temperature for 30 min, and then a THF (4 mL) solution of methallyl bromide (0.5 g, 3.6 mmol) was added at 0 °C. The cold bath was removed, and the resulting mixture was stirred at room temperature overnight. The reaction was quenched by the addition of H_2O. The phases were separated, and the aqueous phase was extracted two additional times with Et_2O. The combined organic layers were washed with brine, dried over anhydrous $MgSO_4$, filtered, and concentrated. The residue was purified by a silica gel column chromatography (pentane/EtOAc = 9:1), which furnished allyl ether [**1ar** 0.625 g, 2.34 mmol, 69% (2 steps)] as a yellow oil.

^{1}H NMR ($CDCl_3$, 300 MHz) δ 7.57 (d, *J* = 8.3 Hz, 2H), 7.48 (d, *J* = 8.0 Hz, 2H), 6.66 (dt, *J* = 15.9, 1.6 Hz, 1H), 6.40 (dt, *J* = 16.0, 5.6 Hz, 1H), 5.01 (m, 1H), 4.94 (m, 1H), 4.15 (dd, *J* = 5.6, 1.6 Hz, 2H), 3.96 (s, 2H), 1.78 (s, 3H). **^{13}C NMR** ($CDCl_3$, 75 MHz) δ 142.2, 140.4, 132.5, 129.5 (q, *J* = 32.3 Hz), 129.2, 126.7, 125.6 (q, *J* = 3.8 Hz), 124.3 (q, *J* = 270.0 Hz), 112.5, 74.5, 70.2, 19.7. **^{19}F NMR** (283 MHz, $CDCl_3$): δ –62.9.

These data are in agreement with those reported previously in the literature.[28]

3. Products characterization data

3.1. Hydrocarboxylation product

2-(4-cyanophenyl)propanoic acid (2a)

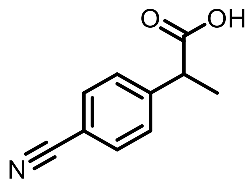

Prepared from 0.1 mmol (13.0 mg) of corresponding alkene and obtained as a yellowish-white solid in 95% (15.9 mg) isolated yield. **^{1}H NMR**: (400 MHz, $CDCl_3$) δ 7.63 (d, *J* = 8.6 Hz, 2H), 7.43 (d, *J* = 8.2 Hz, 2H), 3.80 (q, *J* = 7.2 Hz, 1H), 1.53 (d, *J* = 7.2 Hz, 3H). **^{13}C NMR**: (101 MHz, $CDCl_3$) δ 179.4, 145.1, 132.6 (2C), 128.7 (2C), 118.7, 111.6, 45.8, 18.1. **IR (neat)**: ν 3093, 2988, 2920, 1692, 1608, 1459 cm^{-1}. **HRMS (ESI) (*m/z*)**: $[M+Na]^+$ calculated for $[C_{10}H_9NO_2Na]^+$: 198.0525, found: 198.0526.

2-(4-(trifluoromethyl)phenyl)propanoic acid (2b)

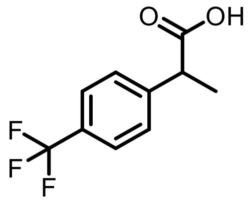

Prepared from 0.1 mmol (17.2 mg) of corresponding alkene and obtained as a white solid in 46% (10.0 mg) isolated yield. **^{1}H NMR** (300 MHz, $CDCl_3$): δ 7.60 (d, *J* = 7.8 Hz, 1H), 7.44 (d, *J* = 8.1 Hz, 2H), 3.81 (q, *J* = 7.2 Hz, 1H), 1.54 (d, *J* = 7.2 Hz, 3H). **^{13}C NMR** (75 MHz, $CDCl_3$): δ 180.2, 143.7, 129.9 (q, *J* = 32.5 Hz), 128.2 (2C), 125.8 (q, *J* = 3.8 Hz, 2C), 124.0 (q, *J* = 270.0 Hz), 45.4, 18.2. **^{19}F NMR** (283 MHz, $CDCl_3$): δ –63.0 (3F). **IR (neat)**: ν 2925, 1708, 1619, 1417, 1324, 1231, 1164, 1119, 1069, 863, 842 cm^{-1}. **HRMS (ESI) (*m/z*)**: $[M-H]^-$ calculated for $[C_{10}H_8F_3O_2]^-$: 217.0482, found: 217.0484.

2-(4-(methylsulfonyl)phenyl)propanoic acid (2c)

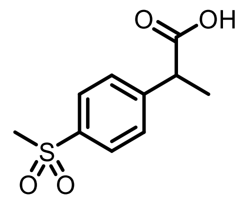

Prepared from 0.1 mmol (18.2 mg) of corresponding alkene and obtained as a brownish white solid in 66% (15.1 mg) isolated yield. **^{1}H NMR** (400 MHz, $CDCl_3$): δ 7.90 (d, *J* = 7.8 Hz, 2H), 7.52 (d, *J* = 8.0 Hz, 2H), 3.90 (br, 1H), 3.04 (s, 3H), 1.56 (d, *J* = 6.3 Hz, 3H). **^{13}C NMR** (101 MHz, $CDCl_3$): δ 178.8, 146.3, 139.8, 128.9 (2C), 127.9 (2C), 45.3, 44.6, 18.4. **IR (neat)**: ν 3063, 3010, 2927, 1700, 1296, 1141, 1083 cm^{-1}. **HRMS (ESI) (*m/z*)**: $[M+Na]^+$ calculated for $[C_{10}H_{12}O_4SNa]^+$: 349.1398, found: 349.1411.

2-(4-acetylphenyl)propanoic acid (2d)

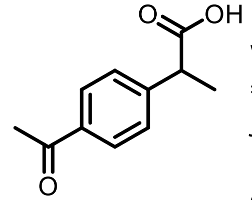

Prepared from 0.1 mmol (14.6 mg) of corresponding alkene and obtained as a white solid in 54% (10.8 mg) isolated yield. **^{1}H NMR** (500 MHz, $CDCl_3$): δ 7.92 (d, *J* = 8.3 Hz, 2H), 7.41 (d, *J* = 8.3 Hz, 2H), 3.81 (q, *J* = 7.2 Hz, 1H), 2.59 (s, 3H), 1.53 (d, *J* = 7.2 Hz, 3H). **^{13}C NMR** (125 MHz, $CDCl_3$): δ 197.9, 179.1, 145.3, 136.4, 128.9 (2C), 128.1 (2C), 45.5, 26.7, 18.2. **IR (neat)**: ν 3052, 2923, 2896, 1695, 1607, 1416, 1253, 927 cm^{-1}. **HRMS (ESI) (*m/z*)**: $[M+H]^+$ calculated for $[C_{11}H_{13}O_3]^+$: 193.0859, found: 193.0860.

2-(4-(methoxycarbonyl)phenyl)propanoic acid (2e)

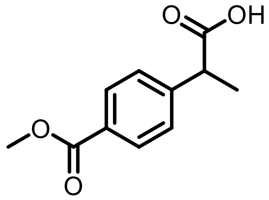

Prepared from 0.1 mmol (16.2 mg) of corresponding alkene and obtained as a white solid in 60% (12.5 mg) isolated yield. ^{1}**H NMR** (500 MHz, $CDCl_3$): δ 8.00 (d, *J* = 8.3 Hz, 2H), 7.39 (d, *J* = 8.3 Hz, 2H), 3.91 (s, 3H), 3.81 (q, *J* = 7.1 Hz, 1H), 1.53 (d, *J* = 7.2 Hz, 3H). ^{13}**C NMR** (126 MHz, $CDCl_3$): δ 179.4, 166.8, 144.8, 130.0 (2C), 129.3, 127.7 (2C), 52.1, 45.3, 18.0. **IR (neat)**: ν 2926, 1721, 1710, 1610, 1436, 1416, 1279, 1181, 1110, 1020, 798, 769 cm^{-1}. **HRMS (ESI) (*m/z*)**: $[M+Na]^+$ calculated for $[C_{11}H_{12}NaO_4]^+$: 231.0628, found: 231.0626.
These data are in agreement with those reported previously in the literature.[29]

2-(4-(*tert*-butoxycarbonyl)phenyl)propanoic acid (2f)

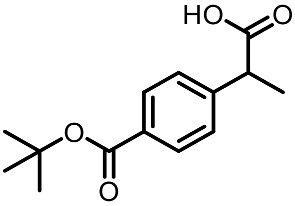

Prepared from 0.1 mmol (20.4 mg) of the corresponding alkene and obtained as a white solid in 64% (16.0 mg) isolated yield. ^{1}**H NMR** (300 MHz, $CDCl_3$): δ 7.95 (d, *J* = 8.5 Hz, 2H), 7.36 (d, *J* = 8.3 Hz, 2H), 3.79 (q, *J* = 7.1 Hz, 1H), 1.58 (s, 3H), 1.52 (d, *J* = 7.2 Hz, 3H). ^{13}**C NMR** (75 MHz, $CDCl_3$): δ 179.3, 165.6, 144.4, 131.4, 130.0 (2C), 127.7 (2C), 81.2, 45.4, 28.3 (3C), 18.2. **IR (neat)**: ν 3080, 2977, 2925, 1697, 1608, 1457, 1410, 1291, 949 cm^{-1}. **HRMS (ESI) (*m/z*)**: $[M+Na]^+$ calculated for $[C_{14}H_{18}O_4Na]^+$: 273.1098, found: 273.1098.

2-([1,1'-biphenyl]-4-yl)propanoic acid (2g)

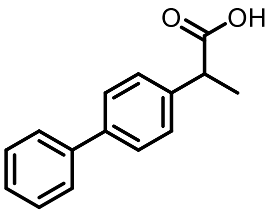

Prepared from 0.1 mmol (18.0 mg) of the corresponding alkene and obtained as a white solid in 42% (9.5 mg) isolated yield. ^{1}**H NMR** (600 MHz, $CDCl_3$): δ 7.58 – 7.56 (m, 4H), 7.44 – 7.40 (m, 4H), 7.36 – 7.33 (m, 1H), 3.81 (q, *J* = 7.2 Hz, 1H), 1.57 (d, *J* = 7.2 Hz, 3H).^{13}**C NMR** (151 MHz, $CDCl_3$): δ 178.5, 140.9, 140.6, 139.0, 128.9 (2C), 128.2 (2C), 127.6 (2C), 127.5, 127.2 (2C), 44.9, 19.0. **IR (neat)**: ν 2978, 2934, 1729, 1520, 1236, 1009, 759, 698 cm^{-1}. **HRMS (ESI) (*m/z*)**: $[M+Na]^+$ calculated for $[C_{15}H_{14}NaO_2]^+$: 249.0886, found: 249.0886.
These data are in agreement with those reported previously in the literature.[29]

2-methyl-2-(naphthalen-2-yl)propanoic acid (2h)

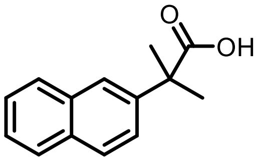

Prepared from 0.1 mmol (16.8 mg) of the corresponding alkene and obtained as a white solid in 28% (6.0 mg) isolated yield. ^{1}**H NMR** (300 MHz, $CDCl_3$): δ 7.84 – 7.79 (m, 4H), 7.54 – 7.43 (m, 3H), 1.71 (s, 6H). ^{13}**C NMR** (75 MHz, $CDCl_3$): δ 181.0, 141.2, 133.2, 132.3, 128.1, 128.1, 127.5, 126.2, 126.0, 124.5, 124.1, 46.4, 26.3 (2C). **IR (neat)**: ν 2923, 2853, 1696, 1413, 1369, 1293, 1133, 818, 752, 480 cm^{-1}. **HRMS (ESI) (*m/z*)**: $[M+Na]^+$ calculated for $[C_{14}H_{14}NaO_2]^+$: 237.0886, found: 237.0886.
These data are in agreement with those reported previously in the literature.[30]

2-(4-cyanophenyl)butanoic acid (2i)

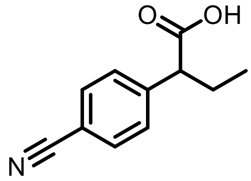

Prepared from 0.1 mmol (14.4 mg) of the corresponding alkene and obtained as a white solid in 84% (15.8 mg) isolated yield. ^{1}**H NMR** (600 MHz, $CDCl_3$): δ 7.63 (d, *J* = 8.6 Hz, 2H), 7.43 (d, *J* = 8.3 Hz, 2H), 3.54 (t, *J* = 7.6 Hz, 1H), 2.22 – 1.74 (m, 2H), 0.91 (t, *J* = 7.4 Hz, 3H). ^{13}**C NMR** (151 MHz, $CDCl_3$) δ 177.9, 143.7, 132.6 (2C), 129.1 (2C), 118.7, 111.7, 53.2, 26.5, 12.1. **IR (neat)**: ν 3286, 3091, 3025, 2924, 2863, 2222, 1688, 1415, 1293, 1086, 934 cm^{-1}. **HRMS (ESI) (*m/z*)**: $[M+Na]^+$ calculated for $[C_{11}H_{11}NO_2Na]^+$: 212.0682, found: 212.0683.

2-(2-cyanophenyl)propanoic acid (2j)

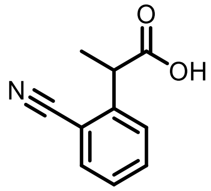

Prepared from 0.1 mmol (12.9 mg) of the corresponding alkene and obtained as a brown solid in 92% (16.1 mg) isolated yield. **^{1}H NMR** (300 MHz, $CDCl_3$): δ 7.65 (ddd, *J* = 7.7, 1.5, 0.6 Hz, 1H), 7.62 – 7.55 (m, 1H), 7.51 – 7.44 (m, 1H), 7.38 (td, *J* = 7.6, 1.3 Hz, 1H), 4.23 (q, *J* = 7.2 Hz, 1H), 1.57 (d, *J* = 7.2 Hz, 3H).**^{13}C NMR** (75 MHz, $CDCl_3$): δ 179.0, 143.5, 133.3, 133.2, 128.0, 127.9, 117.6, 112.8, 43.5, 18.1. **IR (neat)**: ν 3204, 3096, 2962, 2228, 1700, 1600, 1452 cm^{-1}. **HRMS (ESI) (*m/z*)**: $[M+Na]^+$ calculated for $[C_{10}H_9NO_2Na]^+$: 198.0525, found: 198.0526.

2-(2-chlorophenyl)propanoic acid (2k)

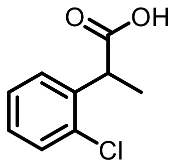

Prepared from 0.1 mmol (13.8 mg) of the corresponding alkene and obtained as an off-white solid in 51% (9.4 mg) isolated yield. **^{1}H NMR** (300 MHz, $CDCl_3$): δ 7.40 – 7.32 (m, 2H), 7.29 – 7.18 (m, 2H), 4.30 (d, *J* = 6.9 Hz, 1H), 1.53 (d, *J* = 7.2 Hz, 3H). **^{13}C NMR** (75 MHz, $CDCl_3$): δ 180.2, 137.8, 134.0, 129.8, 128.7, 128.6, 127.3, 42.1, 17.4. **IR (neat)**: ν 2983, 1703, 1476, 1443, 1413 1234, 1001, 750, 681 cm^{-1}. **HRMS (ESI) (*m/z*)**: $[M+Na]^+$ calculated for $[C_9H_9ClNaO_2]^+$: 207.0183, found: 207.0183.
These data are in agreement with those reported previously in the literature.[31]

2-(3-chlorophenyl)propanoic acid (2l)

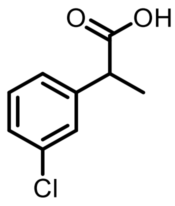

Prepared from 0.1 mmol (13.8 mg) of corresponding alkene and obtained as an off-white solid in 54% (9.6 mg) isolated yield (with 6.5% impurity of oxidized Hantzsch ester). **^{1}H NMR** (300 MHz, $CDCl_3$): δ 7.33 – 7.31 (m, 1H), 7.28 – 7.16 (m, 3H), 3.72 (q, *J* = 7.2 Hz, 1H), 1.51 (d, *J* = 7.2 Hz, 3H). **^{13}C NMR** (75 MHz, $CDCl_3$): δ 179.7, 141.8, 134.6, 130.1, 128.0, 127.8, 126.0, 45.2, 17.8.
These data are in agreement with those reported previously in the literature.[32]

2-(2-bromophenyl)propanoic acid (2m)

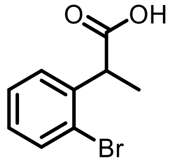

Prepared from 0.1 mmol (18.2 mg) of the corresponding alkene and obtained as a brownish-white solid in 70% (16.0 mg) isolated yield. **^{1}H NMR** (300 MHz, $CDCl_3$): δ 7.57 (ddd, *J* = 8.0 Hz, 1.3 Hz, 0.4 Hz, 1H), 7.37 – 7.27 (m, 2H), 7.16 – 7.10 (ddd, *J* = 8.0 Hz, 7.0 Hz, 2.0 Hz, 1H), 4.28 (q, *J* = 7.2 Hz, 1H), 1.52 (d, *J* = 7.2 Hz, 3H). **^{13}C NMR** (75 MHz, $CDCl_3$): δ 178.9, 140.6, 133.0, 128.5, 127.8, 127.8, 124.8, 44.6, 16.9. **IR (neat)**: ν 3046, 2998, 2922, 1439, 1232, 907 cm^{-1}. **HRMS (ESI) (*m/z*)**: $[M+Na]^+$ calculated for $[C_9H_9BrO_2Na]^+$: 250.9678, found: 250.9679.

2-(perfluorophenyl)propanoic acid (2n)

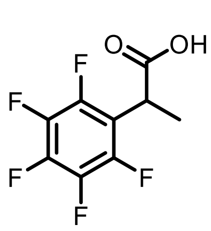

Prepared from 0.1 mmol (19.4 mg) of the corresponding alkene and obtained as a white solid in 61% (14.6 mg) isolated yield. **^{1}H NMR** (300 MHz, $CDCl_3$): δ 4.15 (q, *J* = 7.4 Hz, 1H), 1.57 (d, *J* = 7.4 Hz, 3H). **^{13}C NMR** (75 MHz, $CDCl_3$): δ 177.0, 144.9 (d, *J* = 247.5, 2C), 140.6 (d, *J* = 253.9 Hz), 137.6 (d, *J* = 252.4 Hz, 2C), 114.0 (t, *J* = 16.9 Hz), 34.6, 15.9. **^{19}F NMR:** -142.9 (2F), -156.0, -162.4 (2F). I**R (neat)**: ν 2948, 1716, 1657, 1522, 1503, 1461, 1413, 1301, 1240, 1152, 1135, 1080, 1033, 969, 927 cm^{-1}. **HRMS (ESI) (*m/z*)**: $[M-H+2Na]^+$ calculated for $[C_9H_4F_5Na_2O_2]^+$: 284.9921, found: 284.9922.
These data are in agreement with those reported previously in the literature.[33]

2,2-diphenylpropanoic acid (2p)

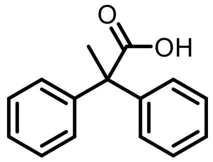

Prepared from 0.1 mmol (18.0 mg) of the corresponding alkene and obtained as a white solid in 90% (20.4 mg) isolated yield. **^{1}H NMR** (300 MHz, $CDCl_3$): δ 7.36 – 7.24 (m, 10H), 1.95 (s, 3H). **^{13}C NMR** (75 MHz, $CDCl_3$): δ 180.8, 143.8 (2C), 128.1 (4C), 128.1 (4C), 127.0 (2C), 56.4, 26.8. **IR (neat)**: ν 2987, 1697, 1494, 1445, 1272, 1029, 925, 761, 727, 697, 657 cm^{-1}. **HRMS (ESI) (*m/z*)**: $[M+Na]^+$ calculated for $[C_{15}H_{14}NaO_2]^+$: 249.0886, found: 249.0887.
These data are in agreement with those reported previously in the literature.[34]

2-(4-methoxyphenyl)-2-phenylpropanoic acid (2q)

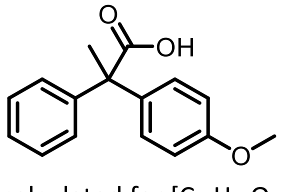

Prepared from 0.1 mmol (21.1 mg) of the corresponding alkene and obtained as a white solid in 57% (14.5 mg) isolated yield. **^{1}H NMR** (300 MHz, $CDCl_3$): δ 7.29–7.25 (m, 5H), 7.23–7.19 (m, 2H), 6.87-6.82 (m, 2H), 3.80 (s, 3H), 1.91 (s, 3H). **^{13}C NMR** (75 MHz, $CDCl_3$): δ 180.6, 158.6, 144.5, 135.7, 129.4 (2C), 128.3 (2C), 128.1 (2C), 127.1, 113.6 (2C), 55.8, 55.4, 27.1. **HRMS (ESI) (*m/z*)**: $[M+Na]^+$ calculated for $[C_{16}H_{16}O_3Na]^+$: 279.0992, found: 279.0992.

2-(4-fluorophenyl)-2-phenylpropanoic acid (2r)

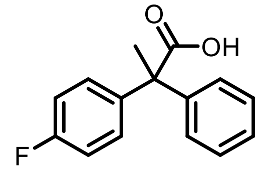

Prepared from 0.1 mmol (19.8 mg) of the corresponding alkene and obtained as a white solid in 54% (13.1 mg) isolated yield. **^{1}H NMR** (500 MHz, $CDCl_3$): δ 7.34 – 7.23 (m, 7H), 7.00 (t, *J* = 8.7 Hz, 2H), 1.93 (s, 3H). **^{13}C NMR** (126 MHz, $CDCl_3$): δ 179.5, 161.8 (d, *J* = 246.5 Hz), 143.7, 139.4 (d, *J* = 3.4 Hz), 129.8 (d, *J* = 7.9 Hz, 2C), 128.3 (2C), 127.8 (2C), 127.2, 114.9 (d, *J* = 21.4 Hz, 2C), 55.8, 27.0. **^{19}F NMR** (283 MHz, $CDCl_3$): δ -116.1. **IR (neat)**: ν 2926, 1698, 1602, 1509, 1464, 1447, 1265, 1230, 1165, 1016, 830, 814, 763, 698 cm^{-1}. **HRMS (ESI) (*m/z*)**: $[M+Na]^+$ calculated for $[C_{15}H_{13}FNaO_2]^+$: 267.0792, found: 267.0793.
These data are in agreement with those reported previously in the literature.[35]

2-phenyl-2-(*o*-tolyl) propanoic acid (2s)

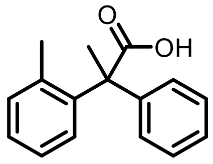

Prepared from 0.1 mmol (19.4 mg) of the corresponding alkene and obtained as a white solid in 75% (18.0 mg) isolated yield. **^{1}H NMR**: (300 MHz, $CDCl_3$) δ 7.42 (dd, *J* = 8.1, 1.7 Hz, 2H), 7.34 – 7.27 (m, 3H), 7.24 – 7.10 (m, 4H), 2.08 (s, 3H), 1.99 (s, 3H). **^{13}C NMR**: ^{13}C NMR (75 MHz, $CDCl_3$) δ 180.5, 142.4, 142.2, 137.0, 132.1, 128.2 (2C), 128.1 (2C), 127.8, 127.2, 127.0, 125.9, 55.7, 26.7, 21.2. I**R (neat)**: ν 2927, 1696, 1492, 1461, 1445, 1267, 1218, 1032, 756, 729, 698, cm^{-1}. **HRMS (ESI) (*m/z*)**: $[M+Na]^+$ calculated for $[C_{16}H_{16}NaO_2]^+$: 263.1043, found: 263.1043.

2-([1,1'-biphenyl]-4-yl)-2-phenylpropanoic acid (2t)

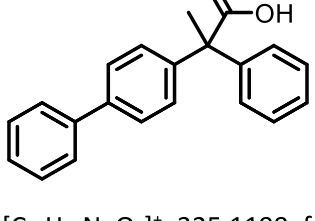

Prepared from 0.1 mmol (25.6 mg) of corresponding alkene and obtained as a white solid in 89% (26.9 mg) isolated yield. **^{1}H NMR** (300 MHz, $CDCl_3$): δ 7.54 (dd, *J* = 13.1, 7.6 Hz, 4H), 7.41 (t, *J* = 7.5 Hz, 2H), 7.36 – 7.24 (m, 8H), 1.96 (s, 3H). **^{13}C NMR** (75 MHz, $CDCl_3$): δ 181.5, 143.9, 142.9, 140.7, 140.0, 128.9 (2C), 128.7 (2C), 128.3 (2C), 128.2 (2C), 127.5, 127.2, 127.2 (2C), 127.0 (2C), 56.4, 27. I**R (neat)**: ν 3028, 1696, 1486, 1446, 1399, 1272, 1217, 1007, 834, 753, 733, 694, 648 cm^{-1}. **HRMS (ESI) (*m/z*)**: $[M+Na]^+$ calculated for $[C_{21}H_{18}NaO_2]^+$: 325.1199, found: 325.1200.

These data are in agreement with those reported previously in the literature.[36]

2,2-bis(4-fluorophenyl)propanoic acid (2u)

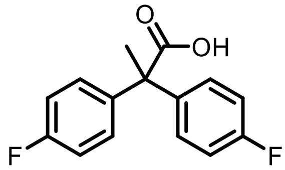

Prepared from 0.1 mmol (21.6 mg) of the corresponding alkene and obtained as a white solid in 91% (23.9 mg) isolated yield. **^{1}H NMR** (600 MHz, $CDCl_3$): δ 7.23 (m, 4H), 7.01 (m, 4H), 1.91 (s, 3H). **^{13}C NMR** (151 MHz, $CDCl_3$): δ 180.8 162.8 (d, *J* = 246.2 Hz, 2C), 139.5 (d, *J* = 3.4 Hz, 2C), 129.8 (d, *J* = 8.0 Hz, 4C), 115.2 (d, *J* = 21.5 Hz, 4C), 55.5, 27.3. **^{19}F NMR** (283 MHz, $CDCl_3$): δ -115.7 (2F). **IR (neat)**: ν 2924, 1697, 1602, 1506, 1273, 1225, 1164, 1015, 828, 738, 636, 572 cm^{-1}. **HRMS (ESI) (*m/z*)**: $[M+Na]^+$ calculated for $[C_{15}H_{12}F_2NaO_2]^+$: 285.0698, found: 285.0698.

These data are in agreement with those reported previously in the literature.[37]

2,2-bis(4-chlorophenyl)propanoic acid (2v)

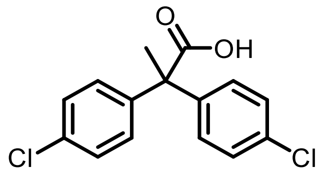

Prepared from 0.1 mmol (24.9 mg) of the corresponding alkene and obtained as a brownish-white solid in 85% (25.0 mg) isolated yield. **^{1}H NMR** (500 MHz, $CDCl_3$): δ 7.25 (m, 4H), 7.16 (m, 4H), 1.87 (s, 3H). **^{13}C NMR** (126 MHz, $CDCl_3$): δ 180.3, 142.1 (2C), 133.4 (2C), 129.5 (2C), 128.6 (2C), 56.0, 26.5. **IR (neat)**: ν 3060, 3020, 2926, 1692, 1489, 1279, 757 cm^{-1}.

These data are in agreement with those reported previously in the literature.[38]

2,2-diphenylbutanoic acid (2w)

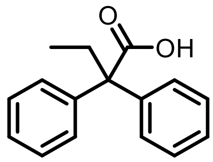

Prepared from 0.1 mmol (19.4 mg) of the corresponding alkene and obtained as a white solid in 75% (18.0 mg) isolated yield. **^{1}H NMR** (600 MHz, $CDCl_3$): δ 7.33 – 7.29 (m, 8H), 7.27 – 7.26 (m, 1H), 7.25 – 7.23 (m, 1H) 2.43 (q, *J* = 7.3 Hz, 2H), 0.77 (t, *J* = 7.3 Hz, 3H). **^{13}C NMR** (151 MHz, $CDCl_3$): δ 179.2, 142.6 (2C), 129.3 (4C), 128.0 (4C), 127.0 (2C), 60.9, 31.0, 10.0. **IR (neat)**: ν 3057, 3022, 2926, 2869, 1692, 1444, 1247, 1116, 914 cm^{-1}. **HRMS (ESI) (*m/z*)**: $[M+Na]^+$ calculated for $[C_{16}H_{16}O_2Na]^+$: 263.1043, found: 263.1043.

2-phenyl-2-(thiophen-2-yl)propanoic acid (2x)

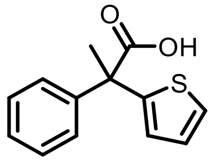

Prepared from 0.1 mmol (18.6 mg) of the corresponding alkene and obtained as a white solid in 47% (10.9 mg) isolated yield. **^{1}H NMR** (300 MHz, $CDCl_3$): δ 7.36 – 7.25 (m, 6H), 7.01 – 6.97 (m, 2H), 2.03 (s, 3H). **^{13}C NMR** (75 MHz, $CDCl_3$): δ 179.4, 146.7, 144.0, 128.3 (2C), 127.4, 126.9 (3C), 126.4, 125.3, 53.9, 28.0. **IR (neat)**: ν 2986, 2634, 1698,1494, 1446, 1402, 1271, 1237, 1029, 931, 761, 694 cm^{-1}. **HRMS (ESI) (*m/z*)**: $[M+Na]^+$ calculated for $[C_{13}H_{12}NaO_2S]^+$: 255.0450, found: 255.0451.

5-methyl-10,11-dihydro-5*H*-dibenzo[*a*,*d*][7]annulene-5-carboxylic acid (2y)

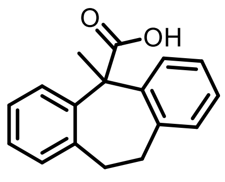

Prepared from 0.1 mmol (20.6 mg) of the corresponding alkene and obtained as a white solid in 65% (16.4 mg) isolated yield. **^{1}H NMR**: (600 MHz, $CDCl_3$) δ 7.40 – 7.38 (m, 2H), 7.19 – 7.15 (m, 4H), 7.13 – 7.11 (m, 2H), 3.38 (q, *J* = 8.7 Hz, 2H), 2.99 (q, *J* = 8.7 Hz, 2H), 2.20 (s, 3H). **^{13}C NMR**: (151 MHz, $CDCl_3$) δ 181.8, 140.5 (2C), 139.1 (2C), 130.7 (2C), 127.6 (2C), 126.2 (2C), 125.6 (2C), 53.4, 32.8 (2C), 25.9. **IR (neat)**: ν 2925, 2855, 1692, 1463, 1259, 1230, 1093, 1039, 750 cm^{-1}. **HRMS (ESI) (*m/z*)**: $[M+Na]^+$ calculated for $[C_{17}H_{16}NaO_2]^+$: 275.1043, found: 275.1043.

2-methyl-4,4-diphenylbut-3-enoic acid (2z major) and α-Methyl-α-[(1*E*)-3-phenyl-1-propen-1-yl]benzeneacetic acid (2z minor)

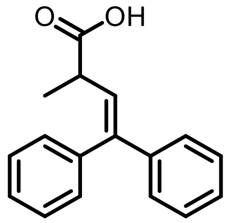

2z
(major)

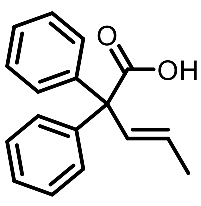

2z
(minor)

Prepared from 0.1 mmol (20.6 mg) of the corresponding alkene and obtained as a yellowish-white solid in 30% (7.6 mg) isolated yield as mixture of regioisomers in 3:1 ratio. **^{1}H NMR** (600 MHz, $CDCl_3$) (major isomer): δ 7.30 – 7.20 (m, 10H), 6.11 (d, *J* = 10.3 Hz, 1H), 3.34 (t, *J* = 8.6 Hz, 1H), 1.31 (d, *J* = 7.0 Hz, 3H). **^{13}C NMR** (151 MHz, $CDCl_3$) (major isomer): δ 180.2, 143.8, 141.9, 139.4, 129.9, 128.6, 128.3, 128.1, 127.5, 127.3, 40.3, 18.5. **^{1}H NMR** (600 MHz, $CDCl_3$) (minor isomer): δ 7.43 – 7.33 (m, 10H), 6.43 (d, *J* = 15.7 Hz, 1H), 5.01 (dt, *J* = 15.6, 6.5 Hz, 1H), 1.78 (dd, *J* = 6.5, 1.6 Hz, 3H). **^{13}C NMR** (150 MHz, $CDCl_3$) (minor isomer, selected peaks): δ 141.6, 134.0, 129.7, 129.2, 18.3. **IR (neat)**: ν 3025, 2966, 2925, 2832, 1700, 1448, 1253, 1071, 932 cm^{-1}. **HRMS (ESI) (*m/z*)**: $[M+Na]^+$ calculated for $[C_{17}H_{16}O_2Na]^+$: 275.1043, found: 275.1043.

3-methyl-3-phenylindolin-2-one (2aa)

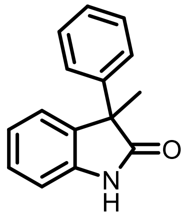

Prepared from 0.1 mmol (19.5 mg) of corresponding alkene and obtained as a white solid in 57% (12.9 mg) isolated yield, includes 10% impurity of the oxidized Hantzsch ester. **^{1}H NMR** (400 MHz, $CDCl_3$): δ 8.74 (s, 1H), 7.34 – 7.28 (m, 4H), 7.27 – 7.20 (m, 2H), 7.15 – 7.12 (m, 1H), 7.05 (td, *J* = 4.5 Hz, 1.0 Hz, 1H), 6.97 (dt, *J* = 7.8 Hz, 0.8 Hz, 1H), 1.83 (s, 3H). **^{13}C NMR** (101 MHz, $CDCl_3$): δ 182.1, 140.7, 140.5, 135.7, 128.7 (2C), 128.2, 127.5, 126.8 (2C), 124.6, 122.9, 52.8, 23.6. **IR (neat):** ν 3180, 3072, 2975, 2927, 1702, 1606, 1597, 1457, 1372, 1286, 1214 cm^{-1}. **HRMS (ESI) (*m/z*)**: $[M+Na]^+$ calculated for $[C_{15}H_{13}NONa]^+$: 246.0889, found: 246.0891.

3-(4-chlorophenyl)-3-methylindolin-2-one (2ab)

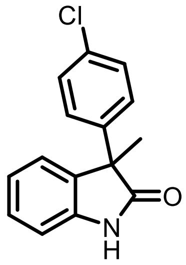

Prepared from 0.1 mmol (23.0 mg) of corresponding alkene and obtained as a yellowish-white solid in 63% (16.4 mg) isolated yield; includes 3% impurity of the oxidized Hantzsch ester. **^{1}H NMR** (600 MHz, $CDCl_3$): δ 8.90 (s, 1H), 7.29 – 7.23 (m, 5H), 7.14–7.04 (dtd, *J* = 15.9 Hz, 7.5 Hz, 1.3 Hz, 2H), 6.97 (dd, *J* = 7.8 Hz, 0.8 Hz, 1H), 1.79 (s, 3H). **^{13}C NMR** (151 MHz, $CDCl_3$) δ 181.8, 140.5, 139.2, 135.1, 133.5, 128.9 (2C), 128.5, 128.3 (2C), 124.5, 123.1, 110.5, 52.4, 23.7. **IR (neat):** ν 3183, 3080, 2978, 2925, 1699, 1610, 1473, 1386, 1291, 1215, 745 cm^{-1}. **HRMS (ESI) (*m/z*)**: $[M+Na]^+$ calculated for $[C_{15}H_{12}ClNONa]^+$: 280.0500, found: 280.0500.

6-Chloro-3-methyl-3-phenylindolin-2-one (2ac)

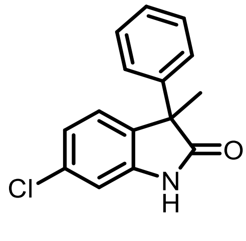

Prepared from 0.1 mmol (23.0 mg) of corresponding alkene and obtained as a yellowish-white solid in 60% (15.6 mg) isolated yield; includes 25% impurity of the oxidized Hantzsch ester. **^{1}H NMR** (600 MHz, $CDCl_3$): δ 9.19 (s, 1H), 7.35 – 7.23 (m, 5H), 7.05 – 7.01 (m, 2H), 6.99 – 6.96 (m, 1H), 1.80 (s, 3H). **^{13}C NMR** (151 MHz, $CDCl_3$): δ 182.4, 141.7, 140.1, 134.1, 133.9, 128.9 (2C), 127.7, 126.7 (2C), 125.4, 122.9, 111.0, 52.6, 25.1. **IR (neat):** ν 3168, 3047, 2985, 2928, 1711, 1601, 1446,

1371, 1285, 1216, 773 cm^{-1}. **HRMS (ESI) (*m/z*)**: $[M+Na]^+$ calculated for $[C_{15}H_{12}ClNONa]^+$: 280.0500, found: 280.0500.

2-(3-fluoro-[1,1'-biphenyl]-4-yl)propanoic acid (2ad)

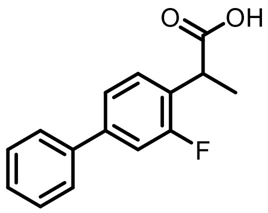

Prepared from 0.2 mmol (39,6 mg) of the corresponding alkene and obtained as a white solid in 92% (45.0 mg) isolated yield. This reaction was also conducted on a 1 mmol scale using 198 mg of the alkene to afford a white solid in 65% (159 mg) isolated yield**^{1}H NMR** (300 MHz, $CDCl_3$): δ 7.56 – 7.51 (m, 2H), 7.47 – 7.34 (m, 4H), 7.20 – 7.14 (m, 2H), 3.80 (q, *J* = 7.1 Hz, 1H), 1.57 (d, *J* = 7.1 Hz, 3H). **^{13}C NMR** (76 MHz, $CDCl_3$): δ 180.0, 159.7 (d, *J* = 248.5 Hz), 140.9 (d, *J* = 7.6 Hz), 135.4 (d, *J* = 1.3 Hz), 130.9 (d, *J* = 4.0 Hz), 128.9 (d, *J* = 3.0 Hz, 2C), 128.4 (2C), 128.2 (d, *J* = 13.6 Hz), 127.7, 123.7 (d, *J* = 3.4 Hz), 115.4 (d, *J* = 23.8 Hz), 44.8, 18.0. **^{19}F NMR** (283 MHz, $CDCl_3$): δ -117.8. **IR (neat)**: ν 2934, 1695, 1414, 1214, 924, 872, 763, 695, 574 cm^{-1}. **HRMS (ESI) (*m/z*)**: $[M+Na]^+$ calculated for $[C_{15}H_{13}FNaO_2]^+$: 267.0792, found: 267.0792.
These data are in agreement with those reported previously in the literature.[39]

2-(4-chlorophenyl)-2-(4-((1-isopropoxy-2-methyl-1-oxopropan-2-yl)oxy)phenyl)propanoic acid (2ae)

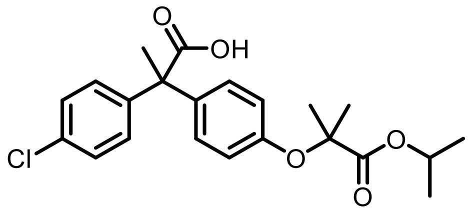

Prepared from 0.1 mmol (35.8 mg) of the corresponding alkene and obtained as a brown solid in 87% (35.4 mg) isolated yield. **^{1}H NMR** (500 MHz, $CDCl_3$): δ 7.22 (d, *J* = 7.1 Hz, 2H), 7.11–7.04 (m, 4H), 6.80-6.74 (m, 2H), 5.06 (hept, *J* = 6.2 Hz, 1H), 1.58 (s, 6H), 1.83 (s, 3H), 1.20 (d, *J* = 6.3 Hz, 6H). **^{13}C NMR** (125 MHz, $CDCl_3$): δ 180.1, 173.4, 154.5, 142.7, 136.1, 132.7, 129.3 (2C), 128.5 (2C), 128.0 (2C), 118.1 (2C), 78.9, 68.8, 53.5, 26.8, 25.2 (2C), 21.4 (2C). **IR (neat)**: ν 2985, 1743, 1700, 1281 cm^{-1}. **HRMS (ESI) (*m/z*)**: $[M+Na]^+$ calculated for $[C_{22}H_{25}ClO_5Na]^+$: 427.1283, found: 427.1284.

2-(3-(1-ethoxy-1-oxopropan-2-yl)phenyl)-2-phenylpropanoic acid (2af)

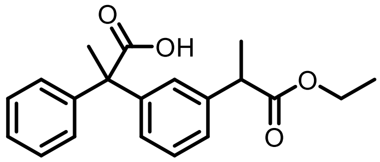

Prepared from 0.1 mmol (28.0 mg) of the corresponding alkene and obtained as a brown solid in 81% (26.7 mg) isolated yield as a mixture of diastereomers (*d.r.* = 1.1:1). Data is reported for the diastereomeric mixture. **^{1}H NMR** (500 MHz, $CDCl_3$): δ 7.36–7.13 (m, 9H), 4.08 (q, *J* = 7.1 Hz, 1H), 3.67 (q, *J* = 7.2 Hz, 1H), 1.94 (s, 3H), 1.46 (d, 7.2 Hz, 3H), 1.16 (t, 7.1 Hz, 3H). **^{13}C NMR** (125 MHz, $CDCl_3$): δ 180.9, 174.6, 144.2, 144.0, 140.6, 128.4, 128.2 (2C), 128.0 (2C), 127.7 (two peaks from different diastereomers), 127.1, 127.0, 126.0 (two peaks from different diastereomers), 60.9, 56.5, 46.1, 27.0, 18.6 (two peaks from different diastereomers), 14.2. **IR (neat)**: ν 3064, 2982, 2935, 1730, 1598, 1452, 1270, 1171, 914 cm^{-1}. **HRMS (ESI) (*m/z*)**: $[M+Na]^+$ calculated for $[C_{20}H_{22}O_4Na]^+$: 349.1410, found: 349.1411.

(2*R*)-2-(4-((((2*aS*,5'*S*,6*aS*,6*bS*,8*aS*,8*bR*,9*S*,10*S*,11*aS*,12*aS*,12b*R*)-5',6*a*,8*a*,9-tetramethyldocosahydrospiro[naphtho[2',1':4,5]indeno[2,1-*b*]furan-10,2'-pyran]-4-yl)oxy)carbonyl)phenyl)propanoic acid (2ag)

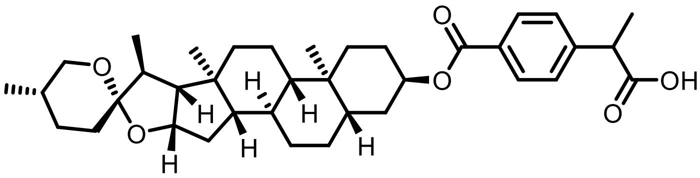

Prepared from 0.1 mmol (54.4 mg) of the corresponding alkene and obtained as a light brown coloured solid in 36% (21.3 mg) isolated yield as a diastereomeric mixture (*d.r.* = 1.1:1). Data is reported for the diastereomeric mixture. **^{1}H NMR** (500 MHz, $CDCl_3$): δ 7.98 (d, *J* = 7.6 Hz, 2H), 7.37 (d, *J* = 7.8 Hz, 2H), 4.96–4.89 (m, 1H), 4.37 (q, *J* = 7.5 Hz, 1H), 3.79 (br, 1H), 3.48 (ddd, , *J* = 10.9, 4.5, 2.3 Hz, 1H), 3.37 (t, *J* = 11.0 Hz, 1H), 1.99–1.91 (m, 2H), 1.86 (t, *J* = 6.9 Hz, 1H), 1.78-1.74 (m, 2H), 1.69–1.60 (m, 8H), 1.54–1.48 (m, 5H), 1.35-1.05 (m, 12H), 0.96 (d, *J* = 7.0 Hz, 3H), 0.88 (s, 3H), 0.79 (d, *J* = 6.4 Hz, 3H), 0.76 (s, 3H). **^{13}C NMR** (125 MHz, $CDCl_3$): δ 178.5, 166.0, 144.9, 130.2, 130.1 (2C), 127.7 (2C), 109.5 (two peaks from different diastereomers), 81.0, 74.5, 67.0, 62.3, 56.4, 54.4, 44.8, 41.8, 40.7, 40.62, 36.9, 35.8, 35.2, 34.3, 32.3, 31.9, 31.5, 30.4, 29.8, 28.9, 28.7, 27.7, 21.2, 18.2, 17.3, 16.56, 14.6, 12.5. **IR (neat):** ν 3104, 2963, 2929, 2858, 1745, 1713, 1271, 1164, 1056, 976 cm^{-1}. **HRMS (ESI) (*m/z*)**: $[M+Na]^+$ calculated for $[C_{37}H_{52}O_6Na]^+$: 615.3656, found: 615.3657.

2-(4-((((8*R*,9*S*,13*S*,14*S*)-13-methyl-17-oxo-7,8,9,11,12,13,14,15,16,17-decahydro-6*H*-cyclopenta[*a*]phenanthrene-3-yl)oxy)carbonyl)phenyl)-propanoic acid (2ah)

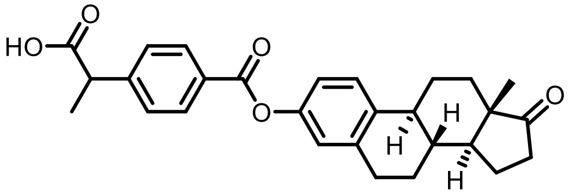

Prepared from 0.1 mmol (40.0 mg) of the corresponding alkene and obtained as a white solid in 54% (23.7 mg) isolated yield. **^{1}H NMR** (600 MHz, $CDCl_3$): δ 8.15 (d, *J* = 8.3 Hz, 2H), 7.47 (d, *J* = 8.2 Hz, 2H), 7.33 (dd, *J* = 8.6, 1.1 Hz, 2H), 6.97 (dd, *J* = 8.5, 2.6 Hz, 1H), 6.93 (dd, *J* = 2.5, 1.2 Hz, 1H), 3.85 (q, *J* = 7.1 Hz, 1H), 2.95–2.91 (m, 2H), 2.52 (ddd, *J* = 19.0, 8.8, 1.0 Hz, 1H), 2.46–2.28 (m, 2H), 2.07-1.88 (m, 4H), 1.65–1.59 (m, 2H), 1.57 (d, *J* = 7.1 Hz, 3H), 1.54–1.43 (m, 4H), 0.92 (s, 3H). **^{13}C NMR** (151 MHz, $CDCl_3$): δ 221.1, 178.4, 165.3, 148.9, 146.0, 138.2, 137.6, 130.7 (2C), 128.9, 128.1 (2C), 126.6, 121.8, 119.0, 50.6, 48.11, 45.6, 44.3, 38.2, 36.0, 31.7, 29.6, 26.5, 25.9, 21.7, 18.3, 14.0. **IR (neat):** 3271 (br, m), 2926, 2854, 1729, 1706, 1671, 1451, 1261, 1215, 1065, 859 cm^{-1}. **HRMS (ESI) (*m/z*)**: $[M+Na]^+$ calculated for $[C_{28}H_{30}O_5Na]^+$: 469.1985, found: 469.1988.

In contrast to **2af**, **2ag** and **2aj**, we did not observe any clear distinct peaks in the ^{1}H and ^{13}C NMR spectra to deduce the diastereomeric ratio. However, we note that this is also not the case for a similar compound that has been reported with a *d.r.* of 1:1 (*Angew. Chem. Int. Ed.* **2018**, *57*, 17220 – 17224, compound **3t** SI Pages S38 and S39).

Given the lack of stereoinduction in all other examples of we have presented, we believe that we also likely have a *d.r.* of approximately 1:1. Attempts to conclusively obtain the d.r. by separation of the diastereomers by GC or HPLC were also largely unsuccessful, although suggestive that there are two diastereomers present in an approximately equal amount.

2-(4-((2-(1*H*-indol-3-yl)ethyl)carbamoyl)phenyl)propanoic acid (2ai)

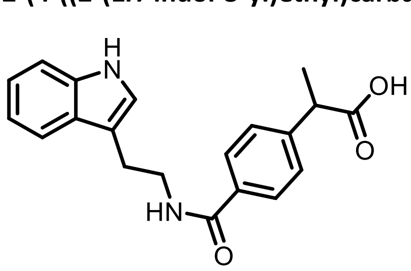

Prepared from 0.1 mmol (29.0 mg) of the corresponding alkene and obtained as a white solid in 45% (15.1 mg) isolated yield. **^{1}H NMR** (600 MHz, MeOD): δ 10.20 (s, NH), 8.46 (t, *J* = 5.8 Hz, NH), 7.72 (d, *J* = 8.1 Hz, 2H), 7.59 (d, *J* = 7.9 Hz, 1H), 7.38 (d, *J* = 8.1 Hz, 2H), 7.32 (d, *J* = 8.1 Hz, 1H), 7.08 – 7.05 (m, 2H), 6.99 – 6.96 (m, 1H), 3.75 (q, *J* = 6.7 Hz, 1H), 3.65 (dd, *J* = 13.7, 7.0 Hz, 2H), 3.05 (t, *J* = 7.4 Hz, 2H), 1.45 (d, *J* = 7.1 Hz, 3H). . **^{13}C NMR** (151 MHz, MeOD): δ 178.0, 170.0, 146.3, 138.2, 134.4, 128.7, 128.6 (2C), 128.4 (2C), 123.5, 122.2, 119.5, 119.2, 113.3, 112.2, 46.7, 42.1, 26.2, 18.8. **IR (neat)**: ν 3434, 2925, 2851, 1704, 1611, 1502, 1456, 1316, 1234, 740 cm^{-1}. **HRMS (ESI) (*m/z*)**: [M+Na]$^+$ calculated for $[C_{20}H_{20}N_2NaO_3]^+$: 359.1366, found: 359,1367.

2-(4-((((1*S*,4*aR*,10*aS*)-7-isopropyl-1,4a-dimethyl-1,2,3,4,4*a*,9,10,10*a*-octahydrophenanthren-1-yl)methyl)carbamoyl)phenyl)propanoic acid (2aj)

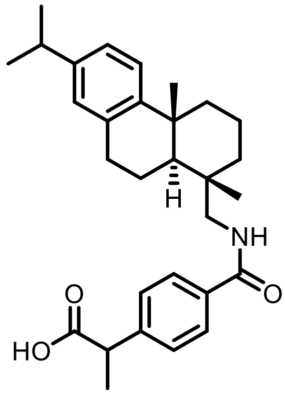

Prepared from 0.1 mmol (41.6 mg) of the corresponding alkene and obtained as a white solid in 91% (42.0 mg) isolated yield as a mixture of diastereomers (*d.r.* = 1.1:1). **^{1}H NMR** (500 MHz, $CDCl_3$): δ 7.66 (d, *J* = 8.0 Hz, 2H), 7.33 (d, *J* = 8.0 Hz, 2H), 7.16 (d, *J* = 8.2 Hz, 1H), 6.99 (dd, *J* = 8.2, 2.0 Hz, 1H), 6.88 (d, *J* = 2.0 Hz, 1H), 6.14 (t, *J* = 6.4 Hz, NH), 3.76 (d, *J* = 7.9 Hz, 1H), 3.37 (qdd, *J* = 13.7, 6.5, 2.5 Hz, 2H), 2.92 (dd, *J* = 17.5, 6.1 Hz, 1H), 2.86 – 2.78 (m, 2H), 2.30 (d, *J* = 12.8, 1H), 1.99 – 1.94 (m, 1H), 1.84 – 1.65 (m, 3H), 1.55 – 1.43 (m, 2H), 1.49 (d, *J* = 6.9 Hz, 3H), 1.42 – 1.25 (m, 3H), 1.23 (s, 3H), 1.22 (s, 3H), 1.21 (s, 3H), 1.00 (s, 3H). **^{13}C NMR** (126 MHz, $CDCl_3$): δ 178.3, 167.8, 147.0, 145.6, 143.6 (two peaks from different diastereomers), 134.7, 133.7 (two peaks from different diastereomers), 127.9 (2C), 127.2 (2C), 126.9, 124.2, 123.9, 50.4, 45.9, 45.2, 38.3, 37.6, 37.5, 36.4, 33.4, 30.4, 25.4, 23.9, 23.9, 19.1, 18.7, 18.6, 18.1. **IR (neat)**: ν 2928, 1709, 1612, 1544, 1500, 1304, 1216, 1175, 753 cm^{-1}. **HRMS (ESI) (*m/z*)**: [M+Na]$^+$ calculated for $[C_{30}H_{39}NNaO_3]^+$: 484.2822 found: 484.2822.

3.2. One-pot hydrocarboxylation-amidation reaction

***N*-benzyl-2-(perfluorophenyl)propenamide (2o)**

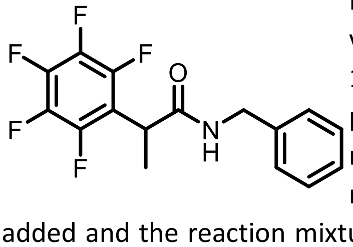

In the first step, 0.1 mmol (28.3 mg) of 1,2,3,4,5-pentafluoro-6-vinylbenzene was reacted with CO_2 in DMF under standard conditions for 18 h. In the second step, the vial was removed from the photo reactor, placed under Ar and cooled to at 0 °C. *N*-benzylamine (24.9 mg, 0.11 mmol, 1.1 equiv.), 2-chloro-1-methylpyridinium iodide (33.2 mg, 0.13 mmol, 1.3 equiv.) and triethylamine (40.5 mg, 0.40 mmol, 4 equiv.) were added and the reaction mixture allowed to warm slowly to room temperature for 4 h. The reaction mixture was diluted with EtOAc and the organic phase was washed with aqueous HCl (1.0 M), followed by sat. aqueous Na_2CO_3, 10% aqueous LiCl and sat. aqueous NaCl, then dried over $MgSO_4$. The organic solvents were removed *in vacuo* and the resulting crude product was purified by flash column chromatography on silica gel (pentane/EtOAc = 80/20) to afford a white solid in 61% (20.1 mg) isolated yield. **^{1}H NMR** (500 MHz, $CDCl_3$): δ 7.35 – 7.32 (m, 2H), 7.30 – 7.27 (m, 1H), 7.25 – 7.23 (m, 2H), 5.88 (t, *J* = 5.6 Hz, NH), 4.45 (dq, *J* = 10.7, 5.6 Hz, 2H), 4.03 (q, *J* = 7.3 Hz, 1H), 1.58 (d, *J* = 7.3 Hz, 3H). **^{13}C NMR** (151 MHz, $CDCl_3$): δ 170.2, 145.1 (dddt, *J* = 247.4, 12.3, 8.2, 3.8 Hz, 2C), 140.4 (d, *J* = 253.9 Hz),

137.8, 137.7 (d, *J* = 253.1 Hz, 2C), 128.8 (2C), 127.7 (2C), 127.7, 114.9 (td, *J* = 16.9, 4.2 Hz) 44.0, 36.0, 16.0 (t, *J* = 2.4 Hz). **^{19}F NMR:** δ -142.1 – -142.2 (m, 2F), -155.7 – -155.9 (m, 1F), -161.9 – -162.1 (m, 2F). **IR (neat)**: ν 3297, 1650, 1520, 1497, 1455, 966, 917, 730, 697 cm^{-1}. **HRMS (ESI) (*m/z*)**: $[M+Na]^+$ calculated for $[C_{16}H_{12}F_5NNaO]^+$: 352.0731, found: 352.0733.

4. Unsuccessful substrates

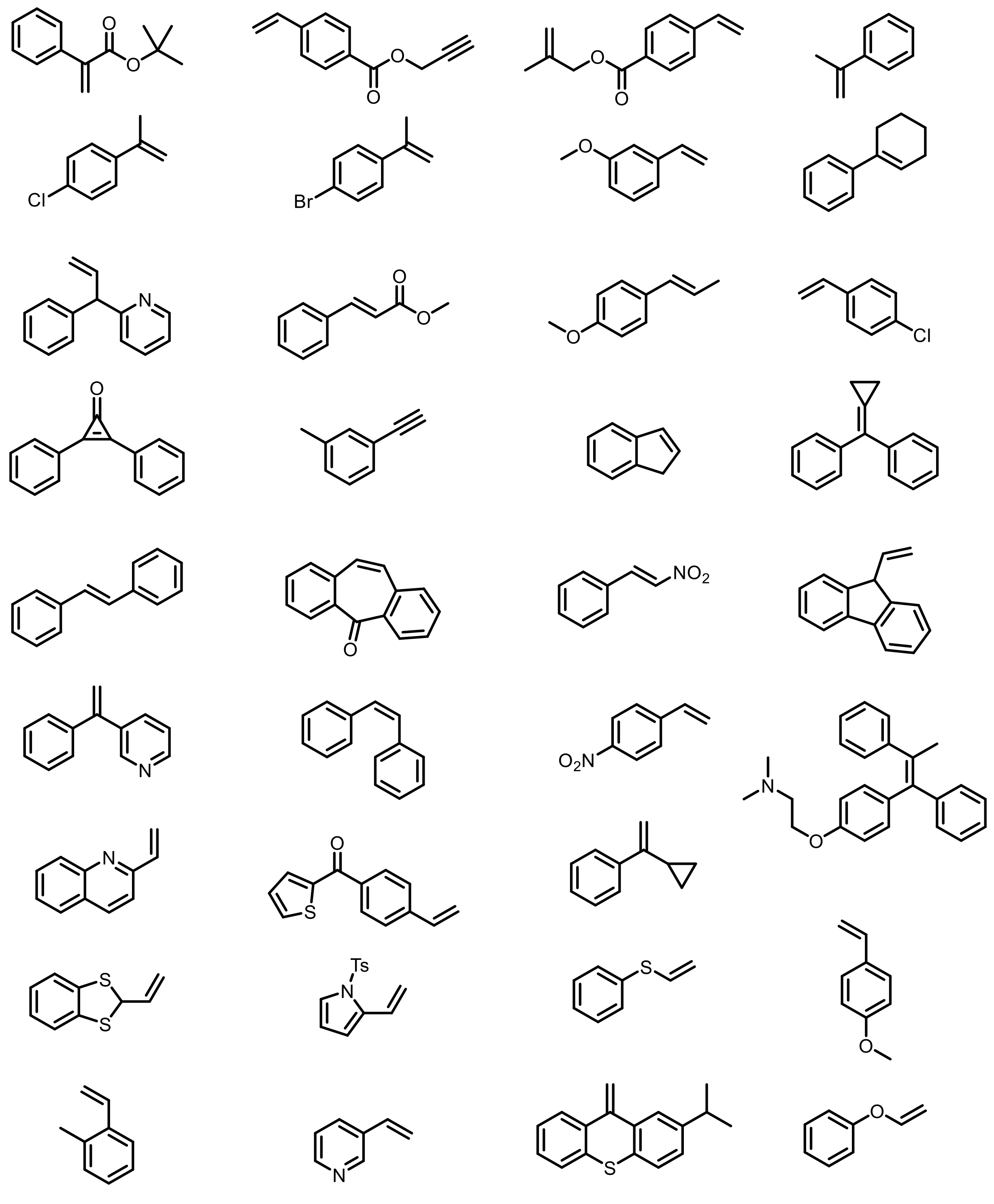

5. Mechanistic experiments

5.1. Investigation into stereoselectivity

The enantiomeric ratio was determined by chiral high-performance liquid chromatography (HPLC) using the racemic product was prepared according to the procedure described in section 3 as reference material.

(±)-2-(2-bromophenyl)propanoic acid (2ar)

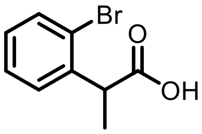

The synthesis of (±)-2-(2-bromophenyl)propanoic acid followed a stepwise approach based on previously reported procedures. [4] [40] [41] To a solution of ester (850 mg, 3.50 mmol, 1.0 equiv.) in absolute EtOH was added NaOH (5.0 equiv.) in deionised water 5 mL. The mixture was refluxed for 1 h. After cooling and concentrated under reduced pressure, the remaining solution was acidified to pH 2 by adding 1 M HCl. On acidification the solution becomes cloudy and precipitate is formed. The residue was filtered under vacuum and washed with cold deionised water. The solid obtained was recrystallised from EtOH/water affording light-brown powder solid (721 mg, 3.14 mmol, 90%). The characterisation data for isolated product matches the provided in (**2m**).

Chiral high-performance liquid chromatography (HPLC) analysis:

The chiral HPLC conditions were as follows DAICEL Chiralpak IC-3 column, dimensions 150 x 4.6 mm, particle size 5 µm, mobile phase 99:1 *n*-Hexane/Isopropanol (*i*PrOH) at a flowrate of 1.0 mL/min

The *rac*-2-(2-bromophenyl)propanoic acid reference shows the retention time (t_r) at 8.455 min and the second peak at t_r = 10.549 min. The enantiomeric ratio (*e.r.*) 53:47.

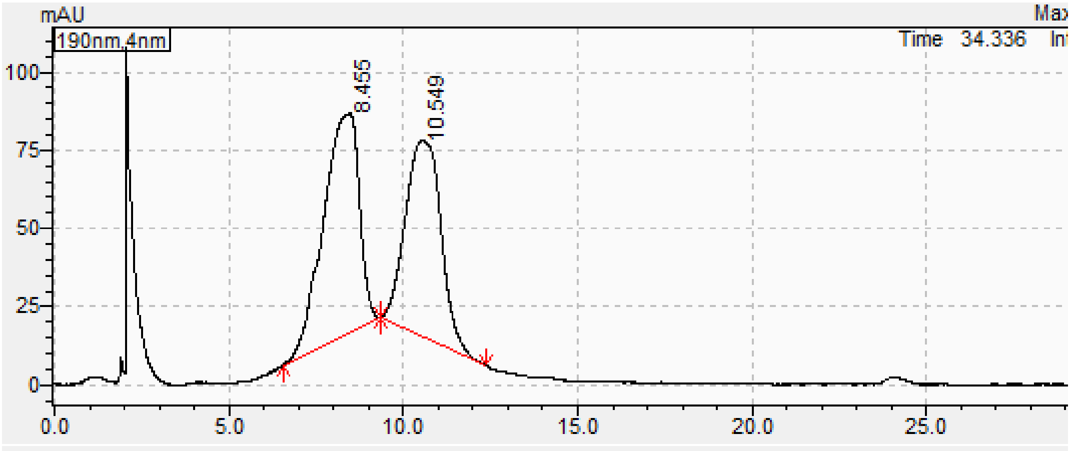

Figure S1: Racemic 2-(2-bromophenyl)propanoic acid

On comparing the product obtained with hydrocarboxylation approach, the two peaks are observed at t_r = 8.242 min and the second peak at t_r = 10.344 min. The *e.r.* 52:48. From this result we can conclude the product obtained is racemic.

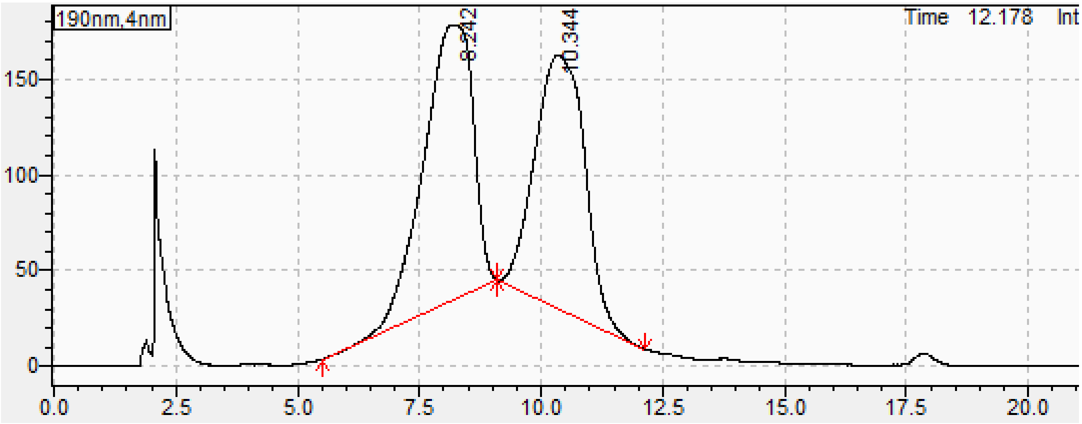

Figure S2: 2-(2-bromophenyl)propanoic acid via hydrocarboxylation approach.

5.2. Deuterium experiments

Diethyl 2,6-dimethyl-1,4-dihydropyridine-3,5-dicarboxylate-1-*d* (HED)

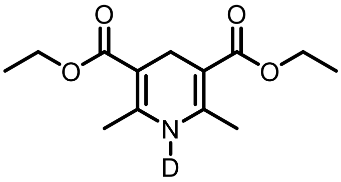

The Hantzsch ester (126.6 mg, 0.5 mmol) previously prepared was suspended in 1.5 mL of CD_3OD and the resulting mixture was stirred overnight under argon atmosphere. The solvent was then evaporated to afford the product as a yellow solid (67.4 mg, 0.27 mmol, 53%).

^{1}H NMR (300 MHz, $CDCl_3$): δ 4.17 (q, *J* = 7.1 Hz, 4H), 3.26 (s, 2H), 2.19 (s, 6H), 1.28 (t, *J* = 7.1 Hz, 6H). This data is in agreement with those reported in the literature.[42]

Diethyl 2,6-dimethyl-1,4-dihydropyridine-3,5-dicarboxylate-4,4-*d₂* (*C4-D*-HEH)

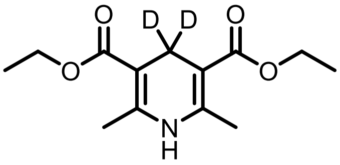

A mixture of paraformaldehyde-d_2 (162 mg, 5.0 mmol, 1.0 equiv.), ethyl acetoacetate (2.60 g, 20.0 mmol, 4.0 equiv.) and ammonium acetate (771 mg,10.0 mmol, 2.0 equiv.) in 6 mL of distilled water was vigorously stirred at refluxing temperature for 17 h. Then, the reaction mixture was allowed to cool down to room temperature. The crude reaction mixture was extracted three times with CH_2Cl_2 and the organic phase was washed with brine. The organic layer was then dried over $MgSO_4$, filtered, and concentrated under reduced pressure. The desired product was obtained on crystallization with ethanol. Isolated as a bright yellow solid (1.17 g, 4.58 mmol, 92%).

^{1}H NMR (300 MHz, $CDCl_3$): δ 5.09 (s, 1H), 4.17 (d, *J* = 7.1 Hz, 4H), 2.19 (s, 6H), 1.28 (d, *J* = 7.1 Hz, 6H).
^{13}C NMR (75 MHz, $CDCl_3$): δ 168.2, 144.9, 99.6, 59.8, 19.4, 14.6.
These data are in agreement with those reported in the literature.[1]

Diethyl 2,6-dimethyl-1,4-dihydropyridine-3,5-dicarboxylate-1,4,4-*d₃* (*C4-D*-HED)

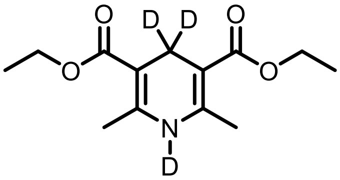

The *C4-D*-HEH (587 mg, 2.3 mmol) previously prepared was suspended in 6 mL CD_3OD and the resulting mixture was stirred overnight under argon atmosphere. The solvent was then evaporated to afford the product as a yellow solid (524 mg, 2.0 mmol, 90%).

^{1}H NMR (600 MHz, $CDCl_3$): δ 5.09 (s, 0.55H), 4.17 (q, *J* = 7.1 Hz, 4H), 2.19 (s, 6H), 1.28 (t, *J* = 7.1 Hz, 6H).

These data are in agreement with those reported in the literature.[1]

2,2-diphenylpropanoic-3-d acid (C4-D-HEH)

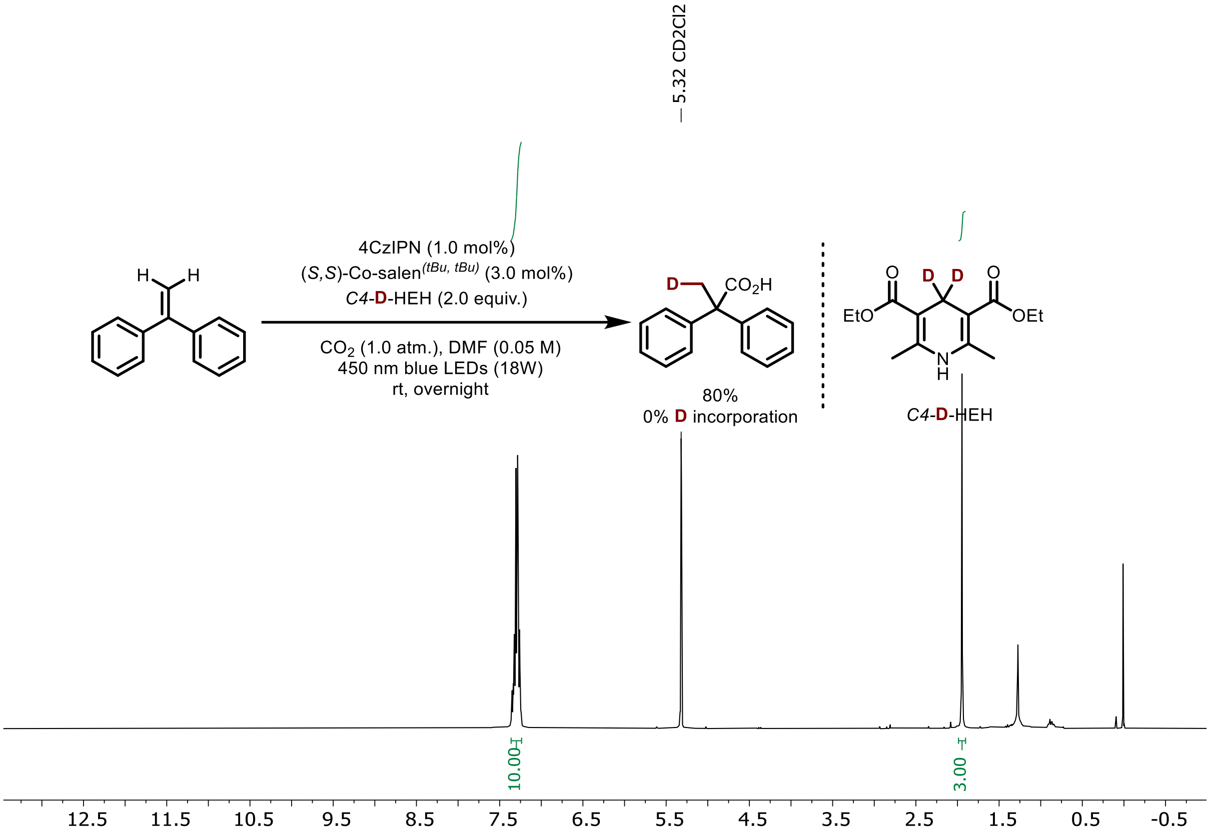

2,2-diphenylpropanoic-3-*d* acid (*C4-D*-HE, with D_2O)

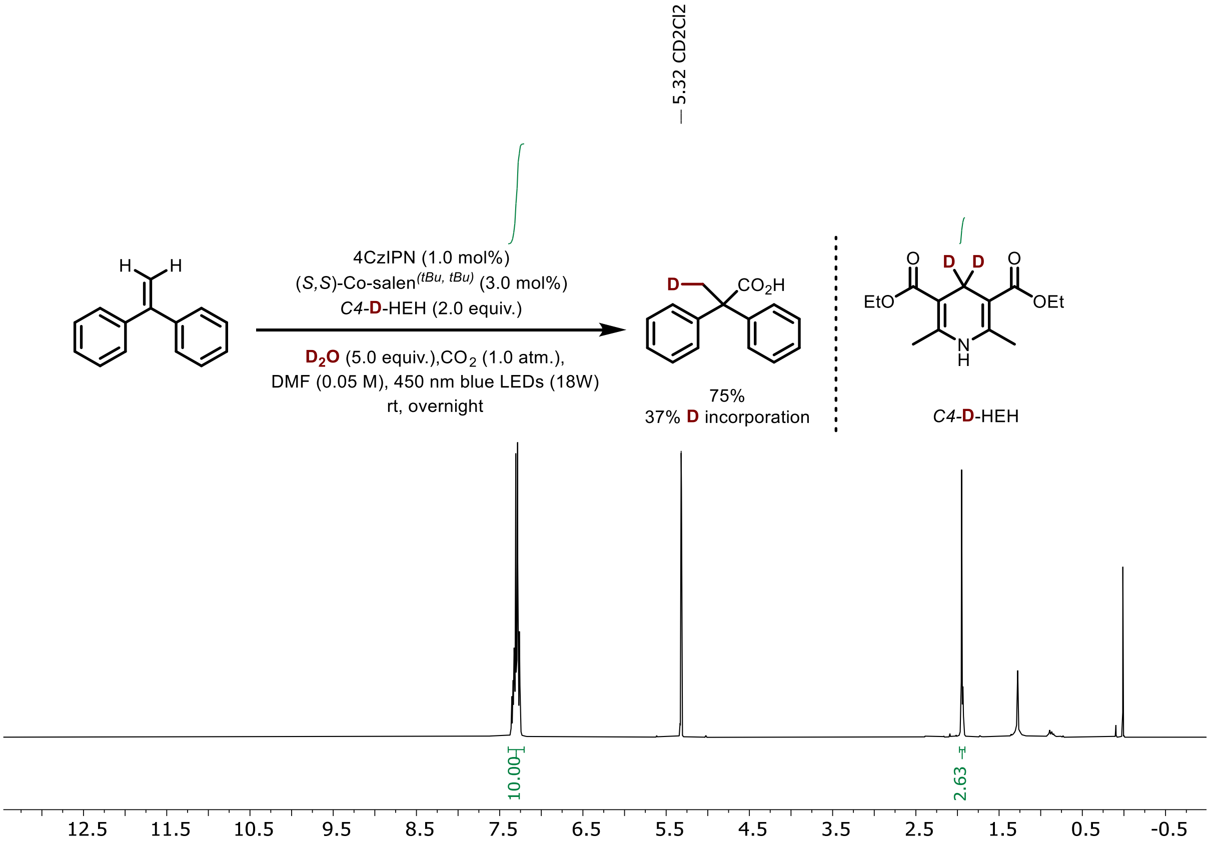

2,2-diphenylpropanoic-3-*d* acid (HEH with D_2O)

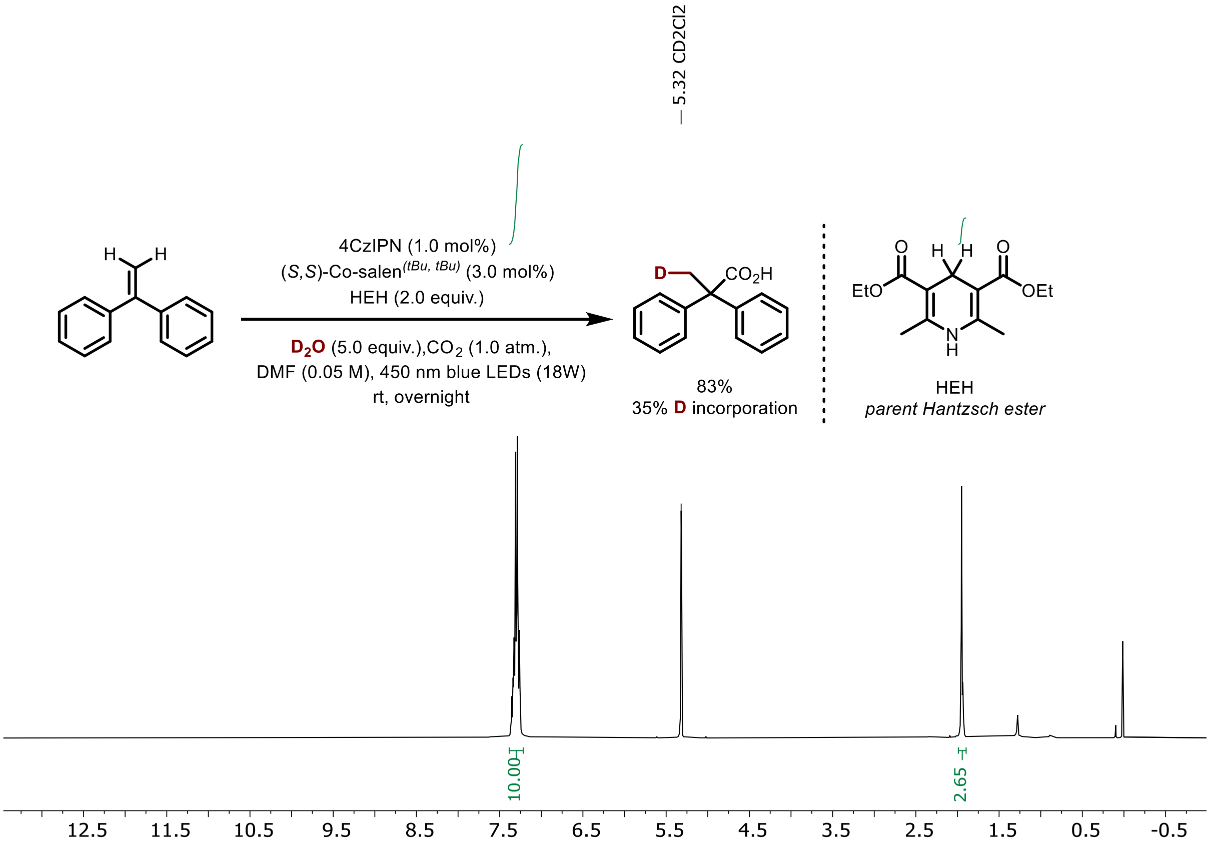

2,2-diphenylpropanoic-3-*d* acid (*N-D*-HEHH)

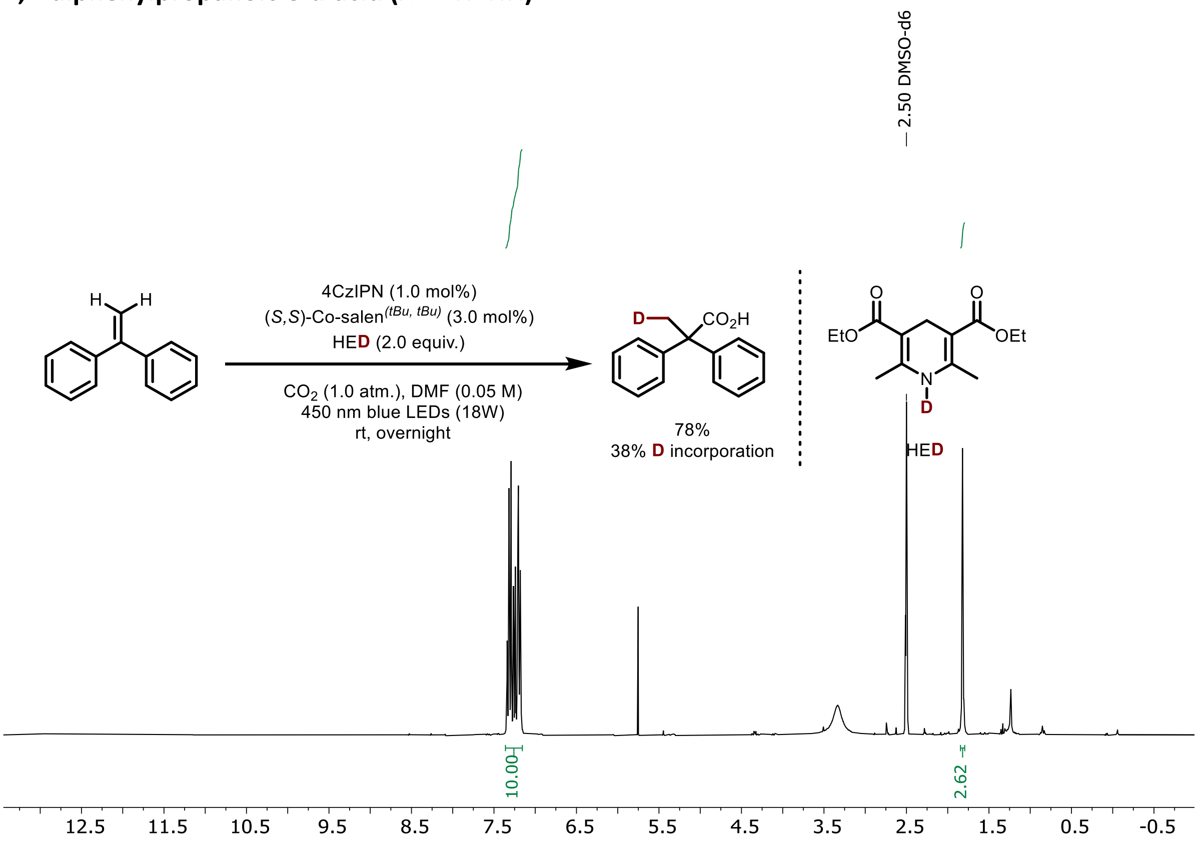

2,2-diphenylpropanoic-3-*d* acid (HED with D_2O)

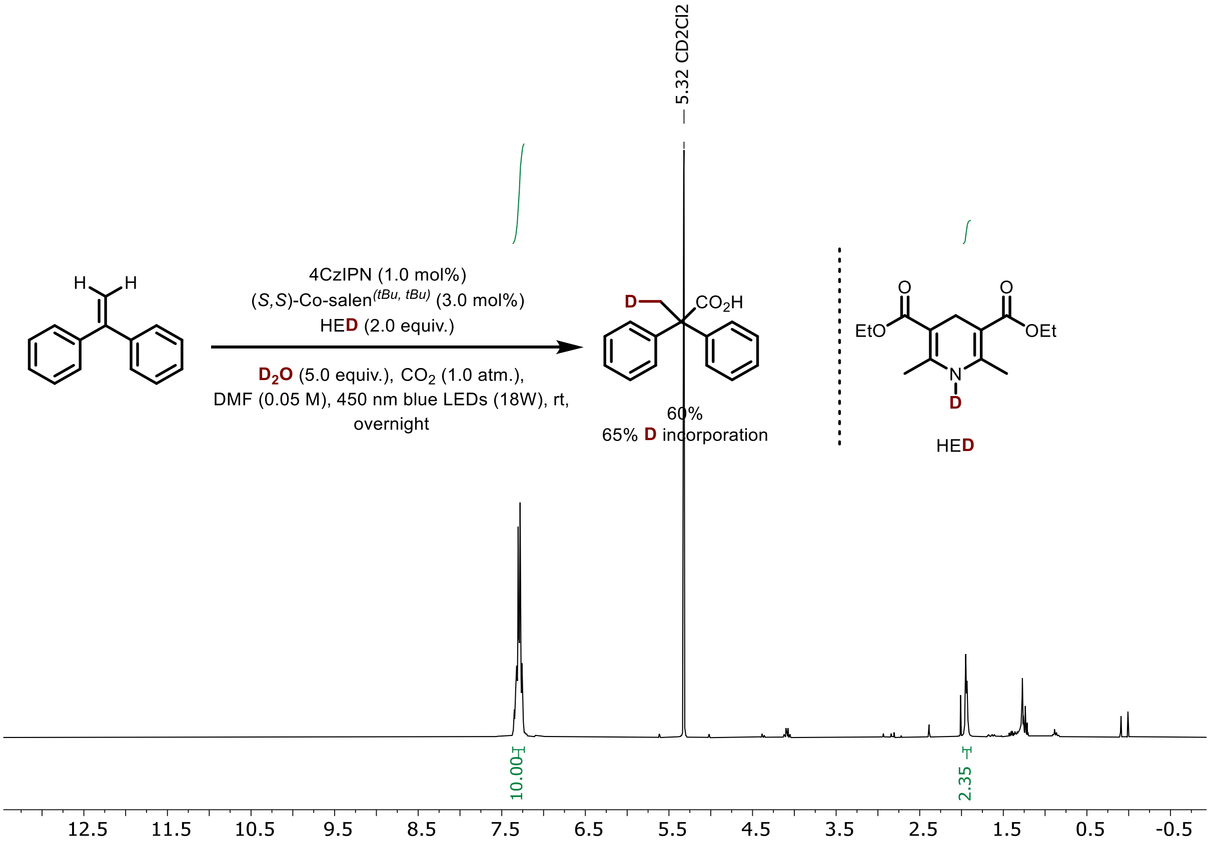

4-(1-phenylethyl-1,2-*d*₂)-1,1'-biphenyl (HEH with D_2O)

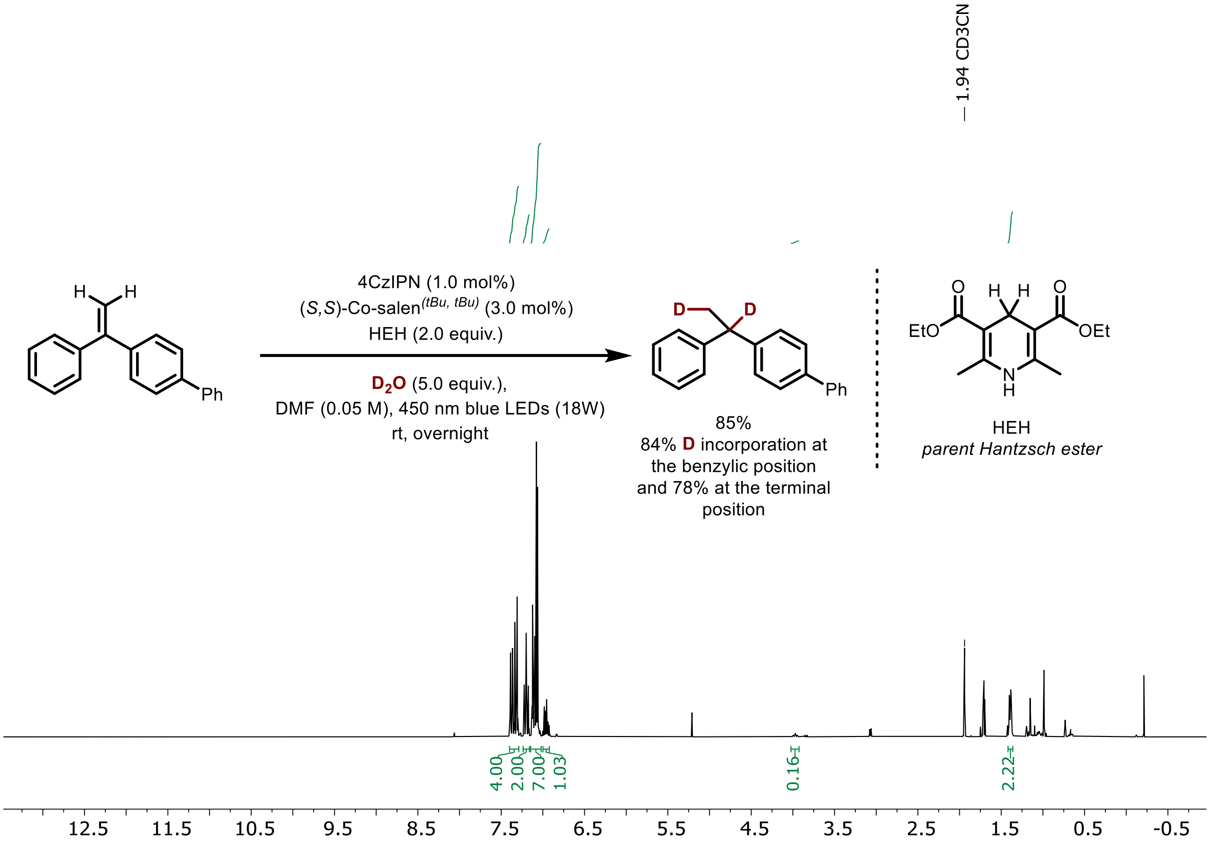

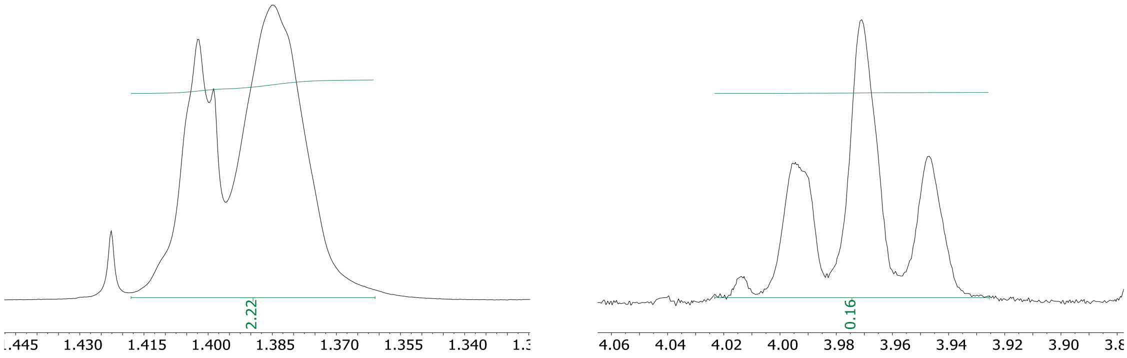

4-(1-phenylethyl)-1,1'-biphenyl (HEH with DMF-*d*$_7$)

N,*N*-dimethylformamide (DMF) is hence not the source of protons. Benzylic anions generated from reductive radical polar crossover can act as superbasic carbanions and are known to pick up protons from solvents like DMF and MeCN.[48] However, in the presence of a good proton source like Hantzsch ester, DMF is not deprotonated as observed in our studies here.

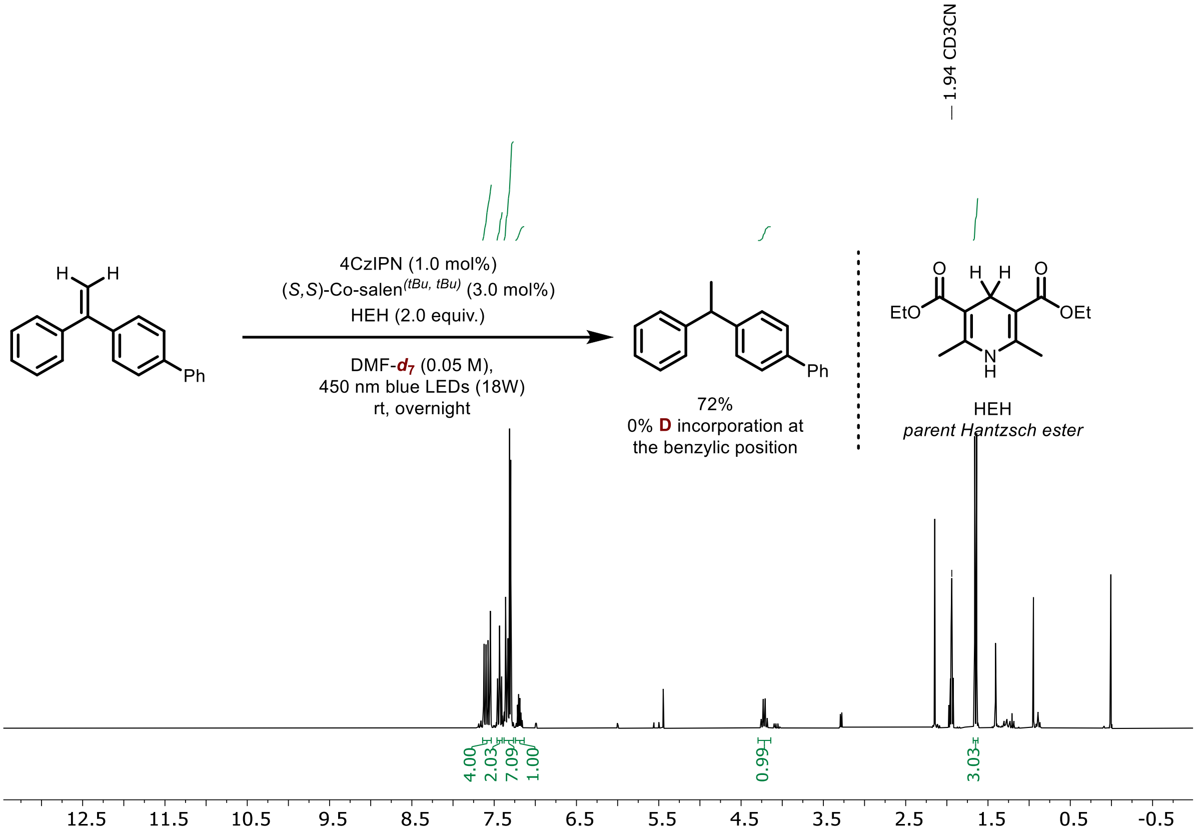

2,2-diphenylpropanoic-3-*d* acid (*C4-D*-HED)

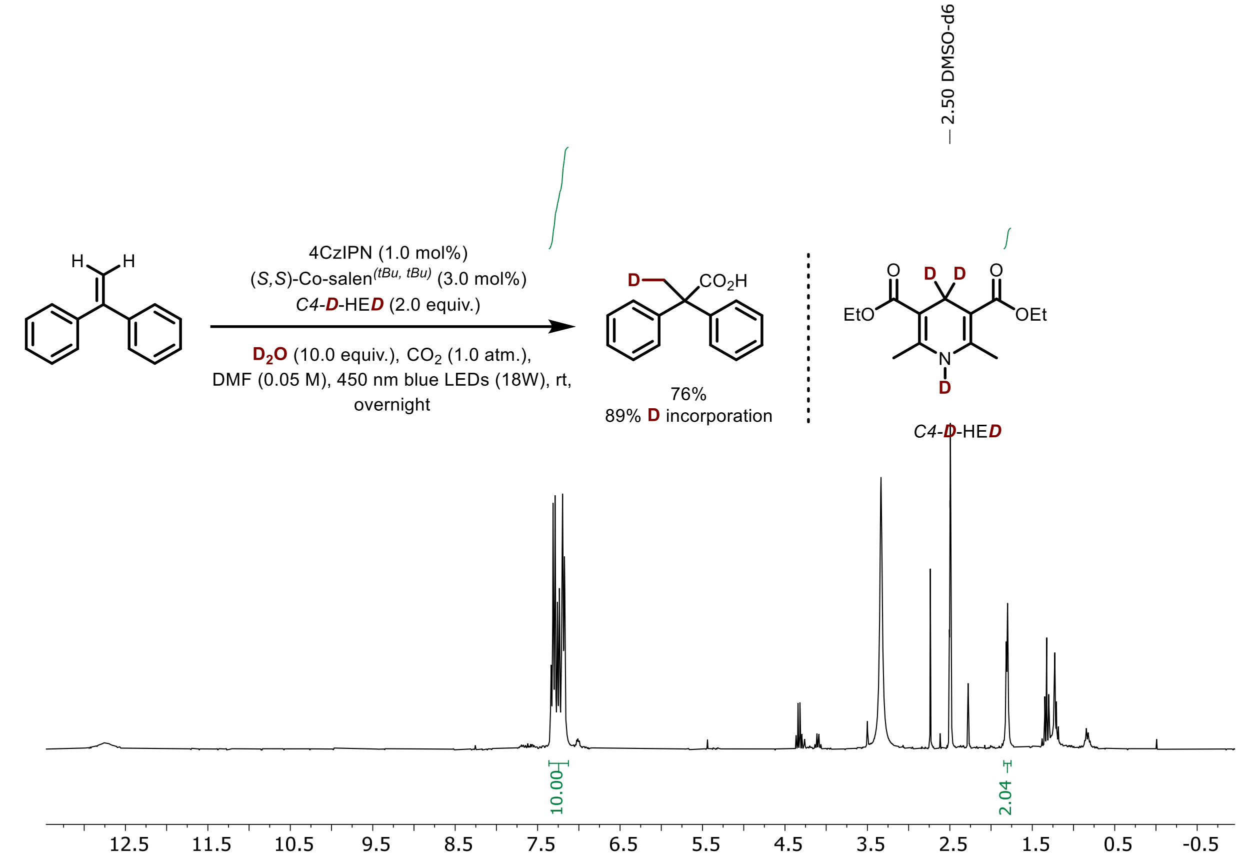

4-(1-phenylethyl-1,2-*d*$_2$)-1,1'-biphenyl (*C4-D*-HED)

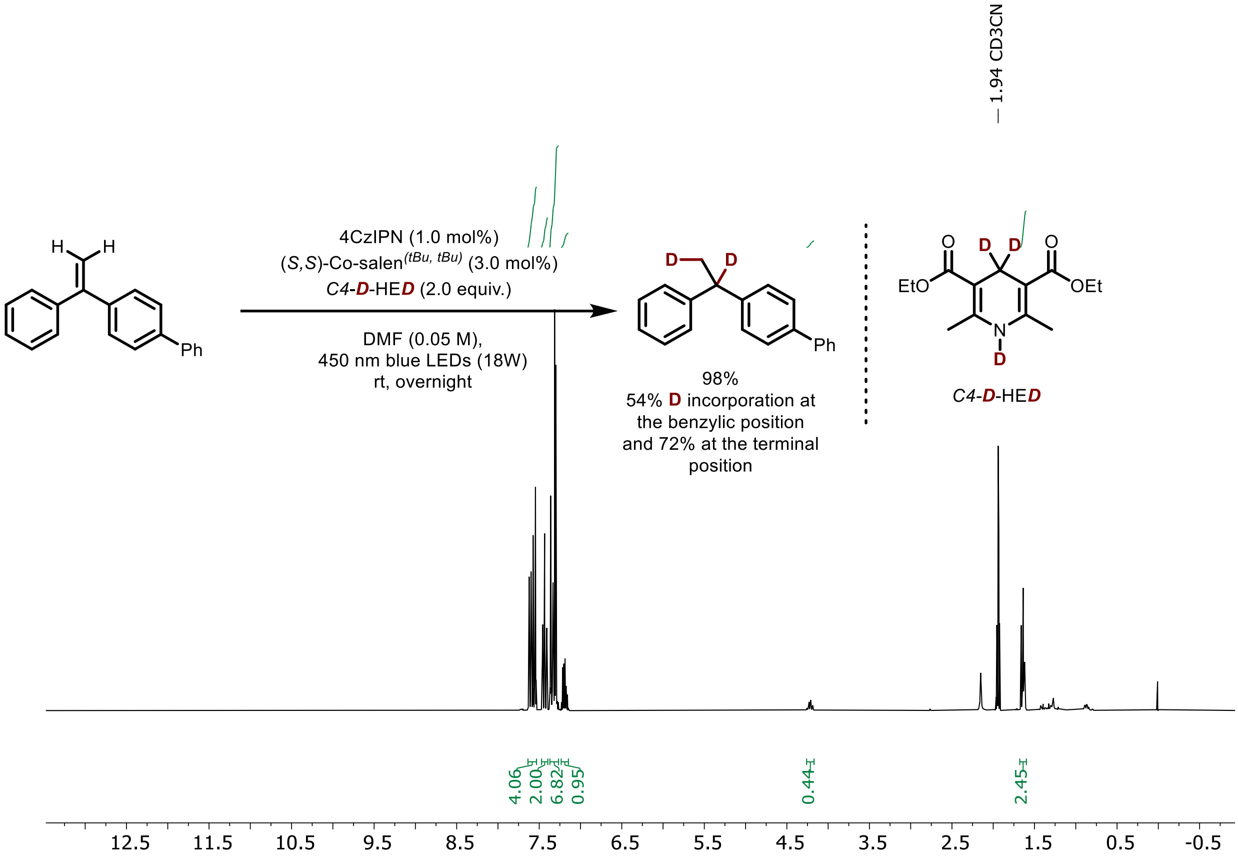

Reversibility of MHAT

Model reaction run with 2.0 equiv. *C4-D*-HED and 5.0 equiv. of D_2O for 0.5 h and we observed no notable deuterium incorporation in the starting material

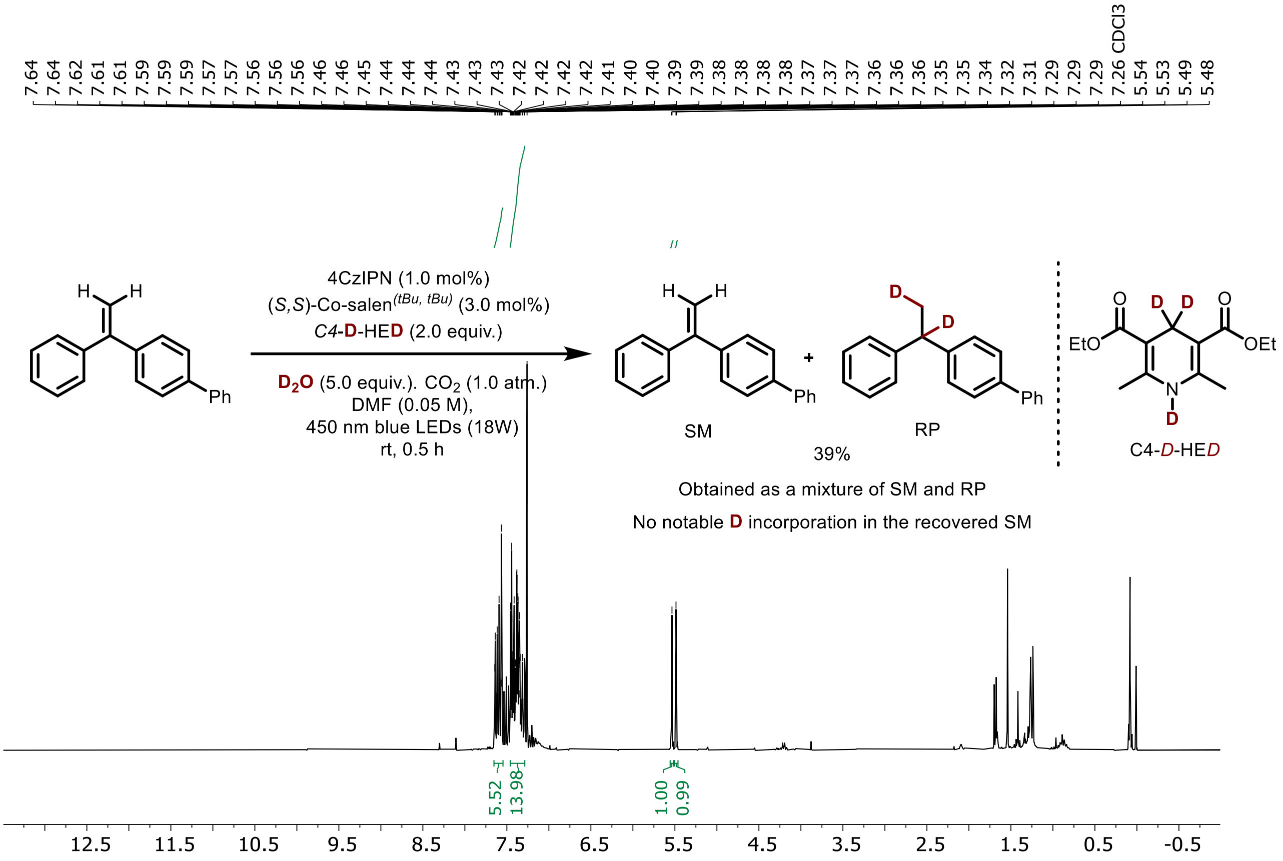

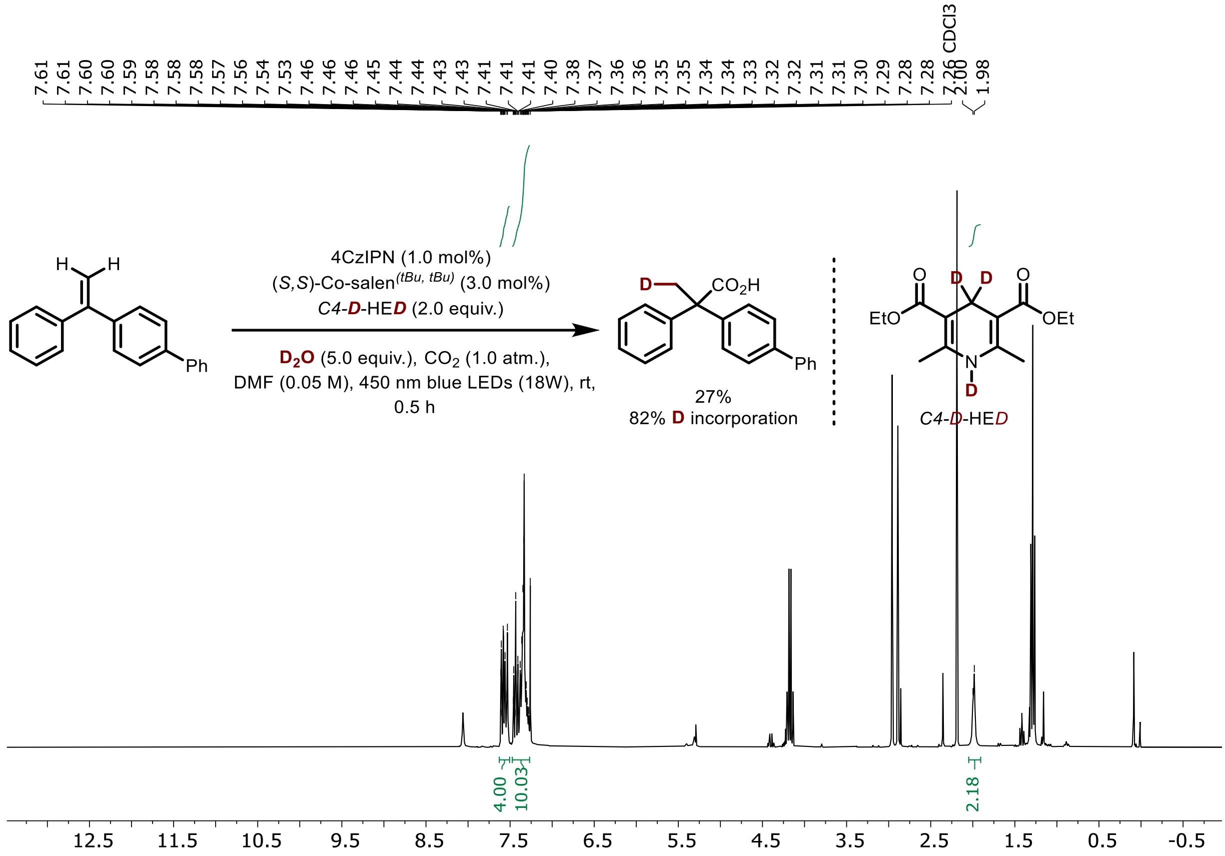

5.3. Photosubstituted photocatalyst studies

2,3,4,6-Tetra(9*H*-carbazol-9-yl)-5-methylbenzonitrile (4CzMeBN) (2ao)

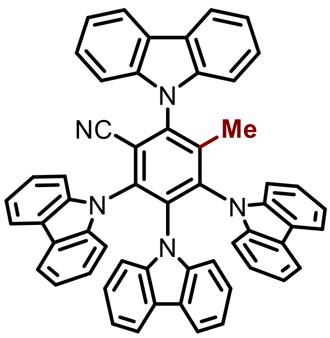

10 mg (0.012 mmol, 1.0 equiv.) of 1,2,3,5-Tetrakis(carbazol-9-yl)-4,6-dicyanobenzen (4CzIPN) and 30.0 mg (0.12 mmol, 10.0 equiv.) Hantzsch ester were loaded in an oven-dried 4 mL vial equipped with a stirring bar and put under an argon atmosphere. Then, 1 mL (0.012 M) of dry DMF was added, and the mixture was irradiated with 450 nm blue LEDs overnight. Then the reaction mixture was diluted with distilled water and extracted with EtOAc and the mixture obtained after drying over anhydrous $MgSO_4$ and concentrating under reduced pressure was purified using silica column chromatography (pentane/ EtOAc = 8/2) to afford 4CzMeBN as a greenish white solid.

^{1}H NMR (600 MHz, $CDCl_3$) δ 8.23 (d, *J* = 7.6 Hz, 2H), 7.73 – 7.66 (m, 6H), 7.52 (d, *J* = 8.1 Hz, 2H), 7.44 (ddd, *J* = 7.9, 7.3, 0.9 Hz, 2H), 7.31 (d, *J* = 7.8 Hz, 2H), 7.25 (t, *J* = 1.1 Hz, 2H), 7.11 – 7.03 (m, 10H), 6.94 (d, *J* = 8.2 Hz, 2H), 6.77 (ddd, *J* = 7.9, 7.3, 0.9 Hz, 2H), 6.64 (ddd, *J* = 8.3, 7.2, 1.2 Hz, 2H), 1.77 (s, 3H). **^{13}C NMR** (151 MHz, $CDCl_3$) δ 142.3, 141.7, 140.8, 140.2, 139.2, 139.1, 138.7, 138.1, 136.2, 127.0, 125.7, 125.6, 124.4, 124.3, 124.2, 123.7, 121.5, 121.4, 121.1, 120.9, 120.4, 120.4, 120.3, 119.5, 117.1, 112.81, 110.2, 110.1, 110.1, 109.3, 15.2 **HRMS** (**ESI**) **(*m/z*):** $[M+Na]^+$ calculated for $C_{56}H_{36}N_5Na$: 800.27976; Found: 800.27852.

[**Note:** The 4CzMeBN photocatalyst was obtained as a mixture with oxidized Hantzsch ester (ox-HEH) in the ratio (4CzMeBN: ox-HEH = 1: 7.7) because its polarity on silica gel column is very similar to that

of the ox-HEH. **^{1}H NMR** (600 MHz, $CDCl_3$): δ 8.67 (s, 1H), 4.39 (q, J = 7.2 Hz, 4H), 2.84 (s, 6H), 1.41 (t, J = 7.1 Hz, 6H). **^{13}C NMR** (151 MHz, $CDCl_3$): δ 166.1, 162.4, 141.0, 123.2, 61.5, 25.1, 14.4].

In order to investigate the effect of CO_2, the reaction was replicated but under an atmosphere of CO_2. The same result isobserved, indicating CO_2 has no role in this reaction.

The methyl substituted photocatalyst is known to form via a photo-substitution reaction from methyl radicals generated from DMSO under similar photocatalytic conditions.[47] To probe if DMF may play a similar role, we carried out the above reaction with deuterated DMF (DMF-d_7).

2,3,4,6-tetra(9*H*-carbazol-9-yl)-5-(methyl-*d3*)benzonitrile (4CzMe-*d*$_3$-BN) (2ao-*d*$_3$)

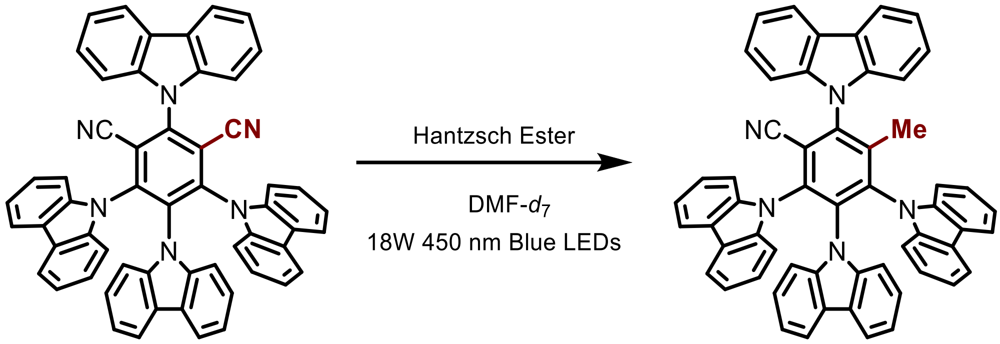

10 mg (0.012 mmol, 1.0 equiv.) of 1,2,3,5-Tetrakis(carbazol-9-yl)-4,6-dicyanobenzen (4CzIPN) and 30.0 mg (0.12 mmol, 10.0 equiv.) Hantzsch ester were loaded in an oven-dried 4 mL vial equipped with a stirring bar and put under an argon atmosphere. Then, 1 mL (0.012 M) of dry deuterated DMF (DMF-*d*$_7$) was added, and the mixture was irradiated with 450 nm blue LEDs overnight. Then the reaction mixture was diluted with distilled water and extracted with EtOAc and the mixture obtained after drying over anhydrous $MgSO_4$ and concentrating under reduced pressure was purified using silica column chromatography (Pentane/EtOAc = 8/2) to afford 4CzMeBN as a greenish white solid.

No deuterium incorporation was observed.

Next we carried out the reaction with *C4-D*-HE*D* Hantzsch ester in DMF-*d*$_7$.

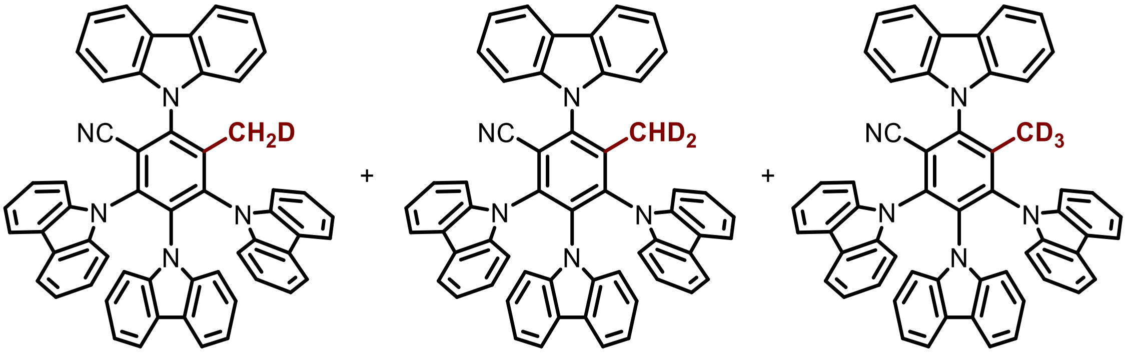

A complex mixture of all three were obtained along with non-deuterated 4CzMeBN as well

10 mg (0.012 mmol, 1.0 equiv.) of 1,2,3,5-Tetrakis(carbazol-9-yl)-4,6-dicyanobenzene (4CzIPN) and 30.0 mg (0.12 mmol, 10.0 equiv.) *C4-D*-HE*D* Hantzsch ester were loaded in an oven-dried 4 mL vial equipped with a stirring bar and put under an argon atmosphere. Then, 1 mL (0.012 M) of dry deuterated DMF (DMF-*d*$_7$) was added, and the mixture was irradiated with 450 nm blue LEDs at room temperature overnight. Then the reaction mixture was diluted with distilled water and extracted with ethyl acetate and the mixture obtained after drying over anhydrous $MgSO_4$ and concentrating under reduced pressure was purified using silica column chromatography (Pentane: EtOAc = 8:2) to afford 4CzMeBN as a greenish white solid.

From analysis of the proton NMR we observed ~60% deuterium incorporation. The mass spectra shows a mixture of three compounds. Mass number 800 refers to the non-deuterated 4CzMeBN whilst 801, 802 and 803 refer to the mono, di and tri deuterated versions, respectively.

^{1}H NMR (300 MHz, $CDCl_3$) δ 8.24 (d, *J* = 7.8 Hz, 2H), 7.70 (m, 6H), 7.52 (d, *J* = 8.1 Hz, 2H), 7.45 (t, *J* = 7.6 Hz, 2H), 7.31 (d, *J* = 7.7 Hz, 2H), 7.25 (t, *J* = 1.1 Hz, 2H), 7.08 (m, 10H), 6.94 (d, *J* = 8.2 Hz, 2H), 6.78 (t, *J*

= 7.4, 2H), 6.65 (m, 2H), 1.77 (s, 3H). **^{13}C NMR** (75 MHz, $CDCl_3$) δ 142.5, 142.0, 141.0, 140.5, 139.4, 139.3, 139.0, 138.3, 136.4, 127.2, 125.9, 125.8, 124.7, 124.5, 124.4, 124.0, 123.9, 121.7, 121.6, 121.4, 121.1, 120.6, 120.6, 120.5, 119.7, 117.4, 113.0, 110.4, 110.3, 110.3, 109.5, 15.4. **HRMS** (**ESI**) ***(m/z)***: $[M+Na]^+$ calculated for $[C_{56}H_{36}N_5Na^+]$: 800.2787, found: 800.2785.

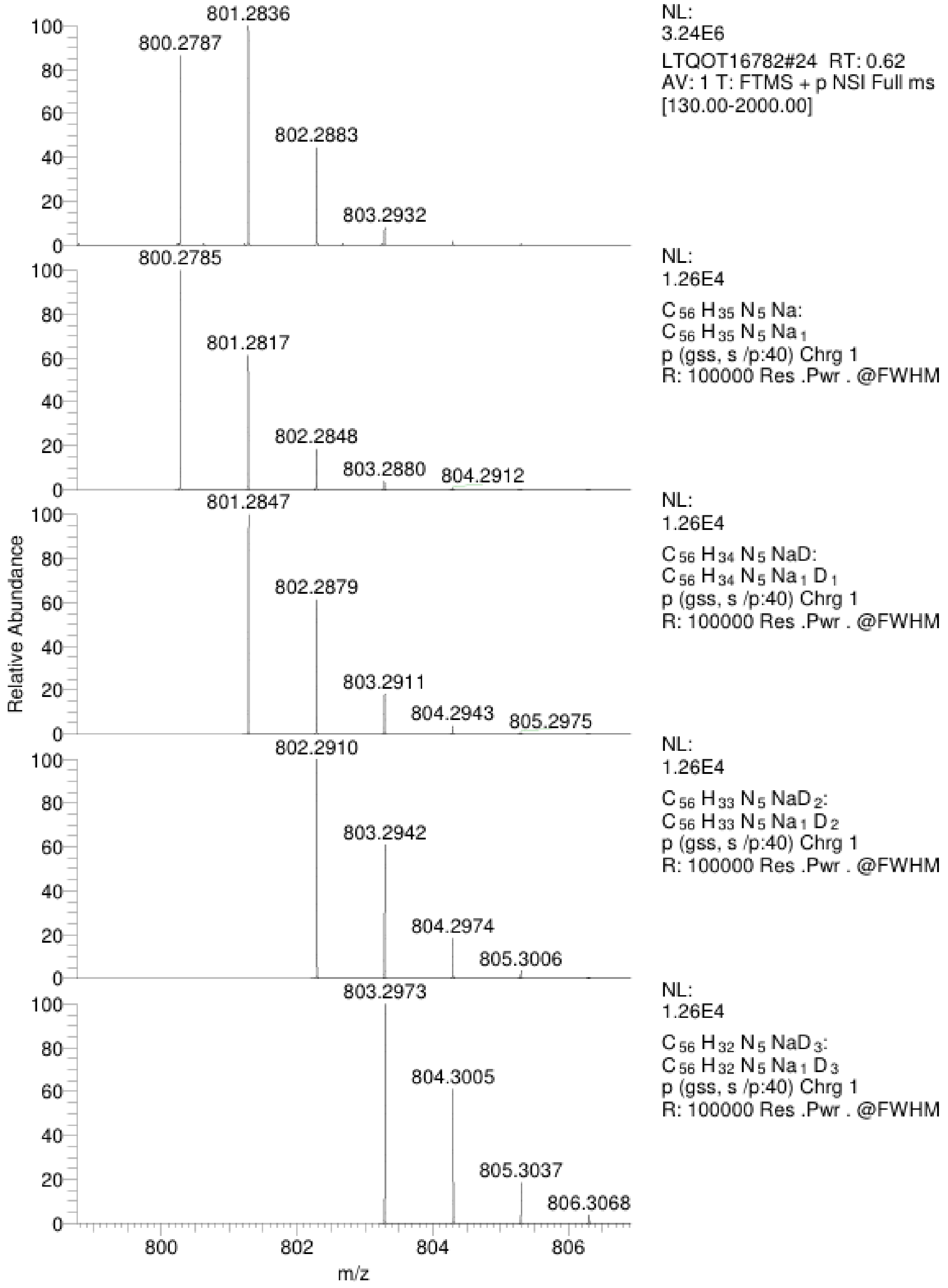

5.4. UV-Vis experiments

UV-Vis spectra were measured at room temperature in air-saturated *N*,*N*-dimethylformamide (DMF) solvent.

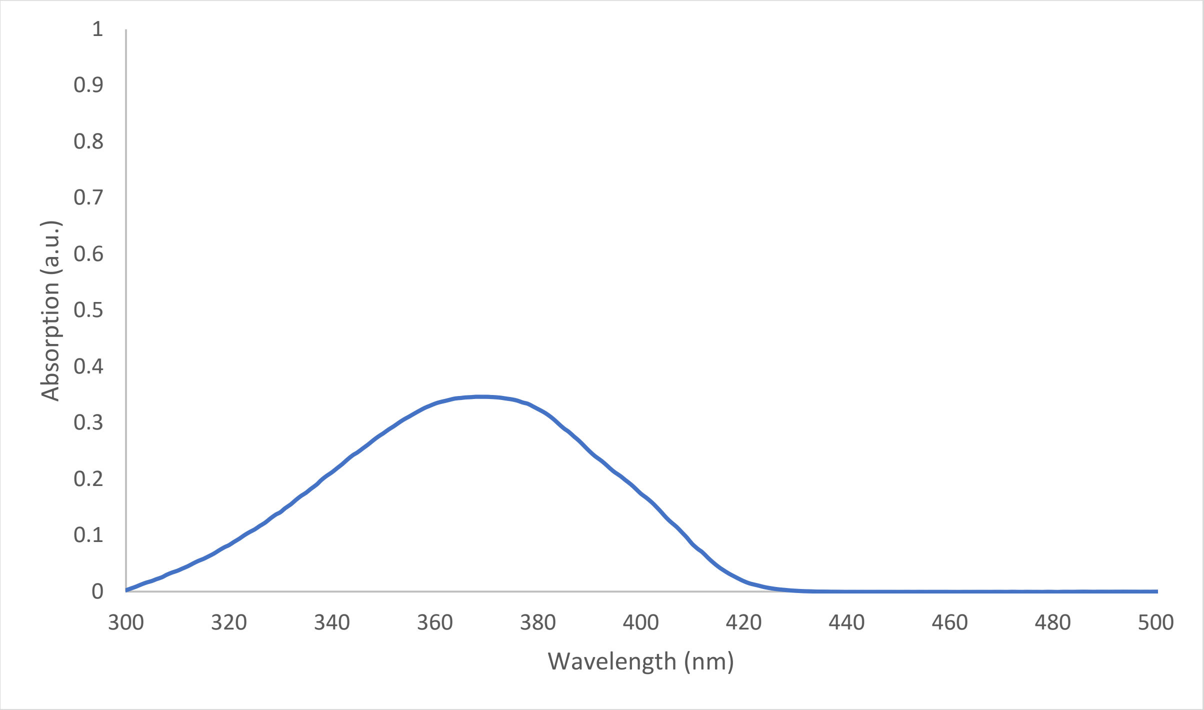

Figure S3: Hantzsch ester (HEH) (65 μM) in DMF solvent.

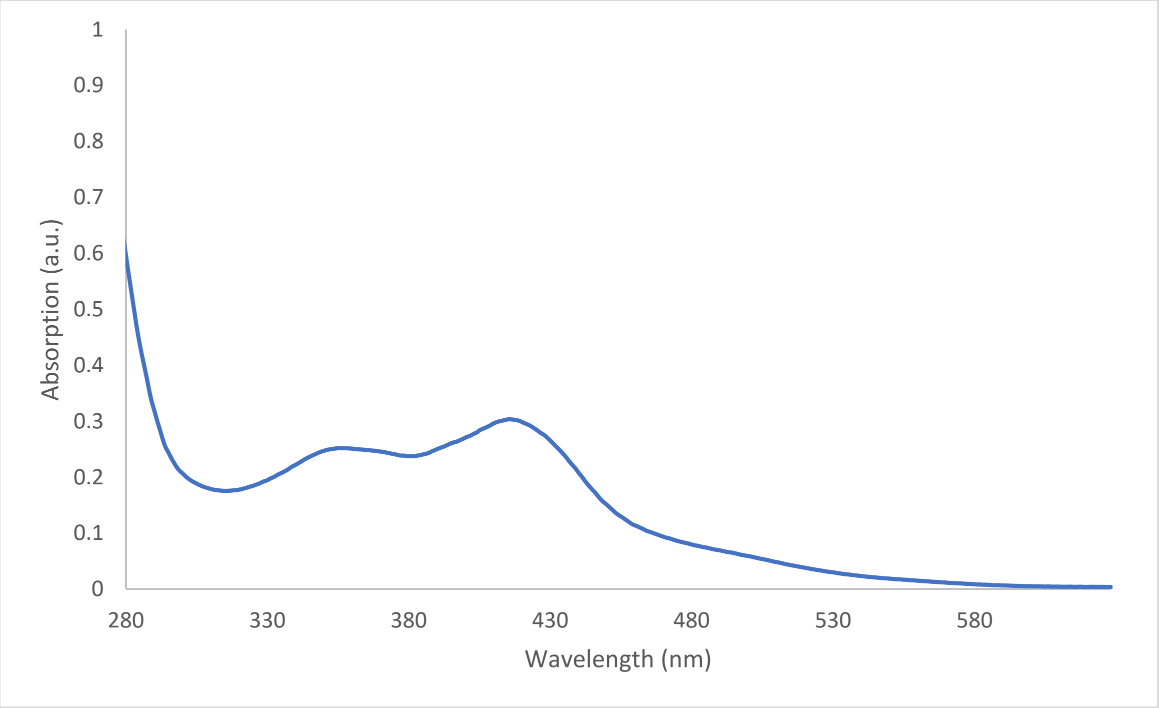

Figure S4: UV-Vis absorption of Co-salen$^{(tBu\text{-}tBu)}$ (36 μM) in DMF solvent.

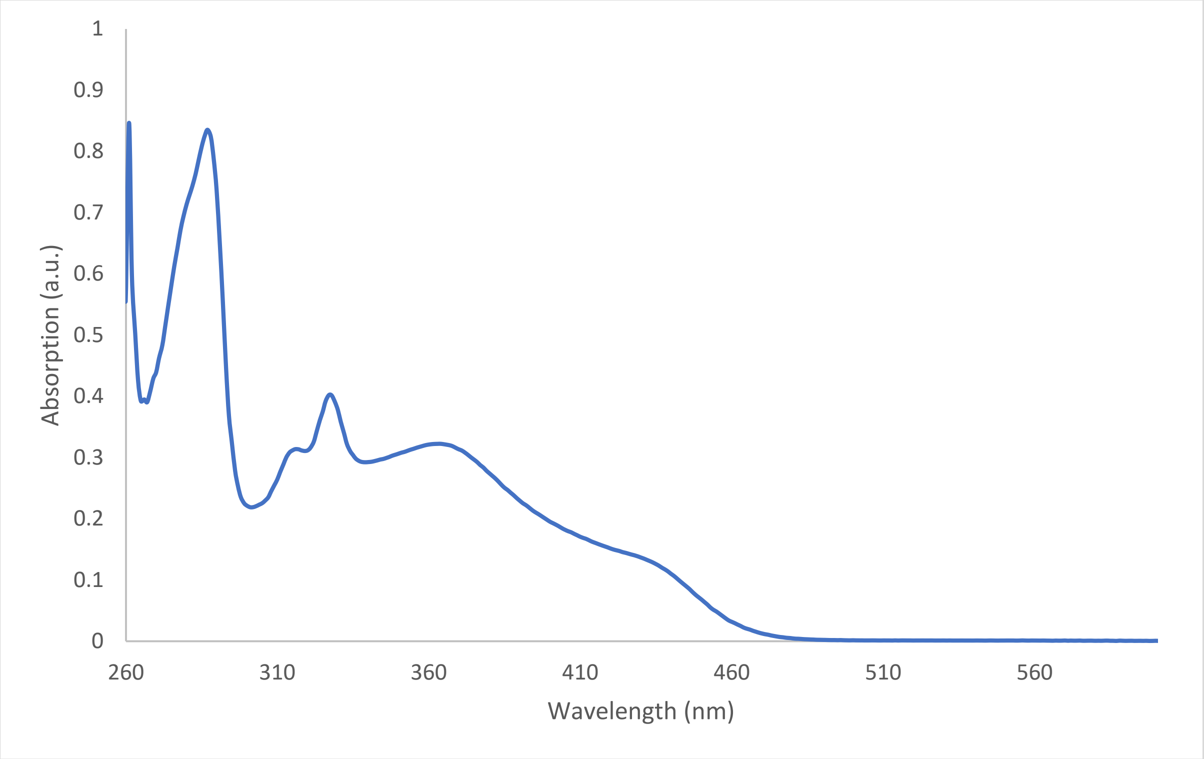

Figure S5: UV-Vis absorption of 1,2,3,5-Tetrakis(carbazol-9-yl)-4,6-dicyanobenzen (4CzIPN) (47 μM) in DMF solvent.

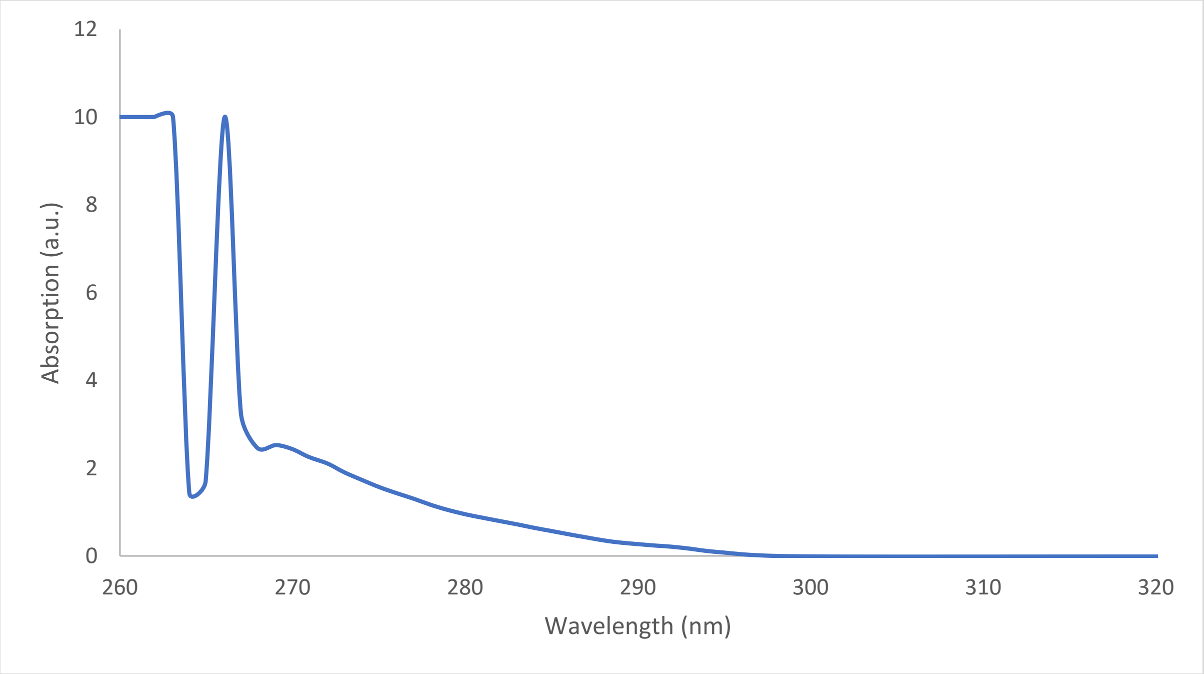

Figure S6: UV-Vis absorption of ethene-1,1-diyldibenzene (166 μM) in DMF solvent

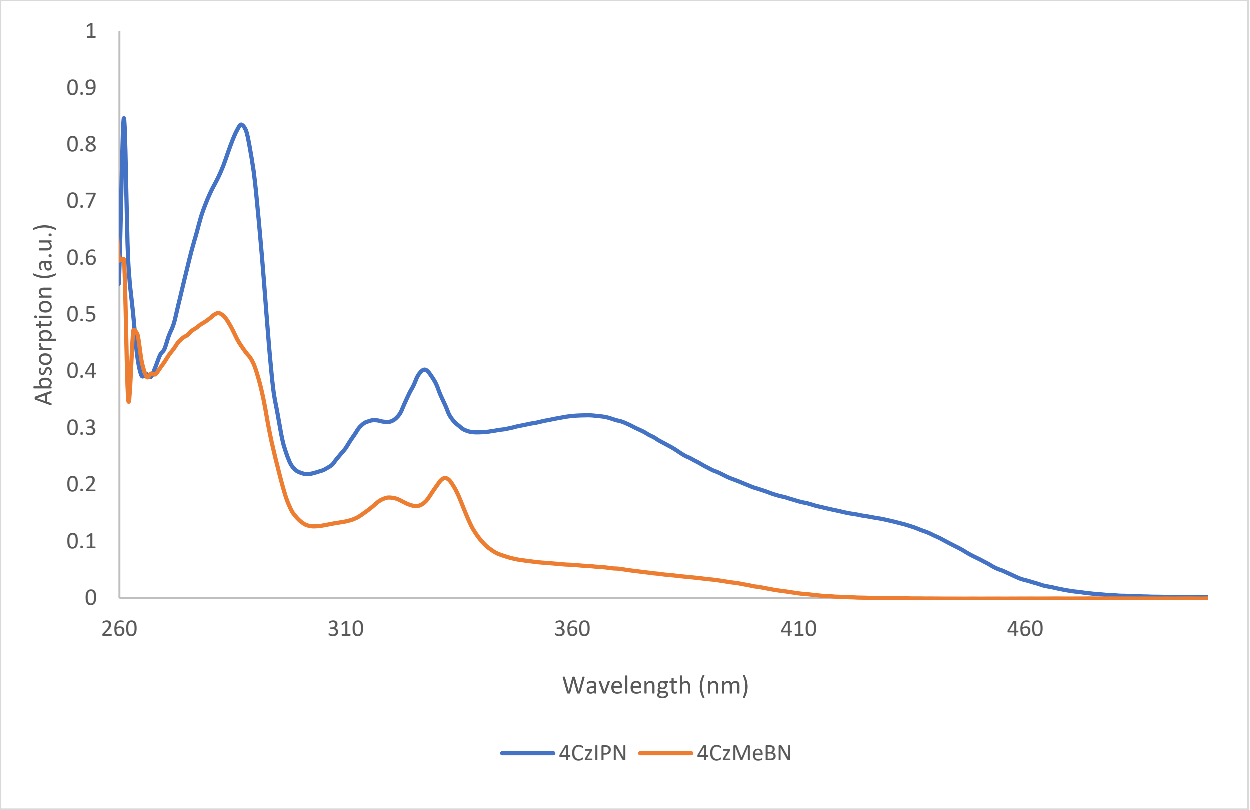

Figure S7: Superposition of UV-vis experiment of 1,2,3,5-Tetrakis(carbazol-9-yl)-4,6-dicyanobenzen (4CzIPN) (47 μM) and 2,3,4,6-Tetra(9H-carbazol-9-yl)-5-methylbenzonitrile (4CzMeBN) (100 μM) in DMF.

5.5. Fluorescence quenching studies

5.5.1. 1,2,3,5-Tetrakis(carbazol-9-yl)-4,6-dicyanobenzene (4CzIPN)

Quenching of 4CzIPN with Co-salen

To a standard solution of 1,2,3,5-Tetrakis(carbazol-9-yl)-4,6-dicyanobenzene (4CzIPN) (0.1 mM in degassed DMF) different amounts of standard solution of Co-salen (1 mM in degassed DMF) were added to afford the final concentrations reported in the table below then the fluorescence of each entry was measured, (quencher concentration; [Co] = 0.00 mM, 0.01 mM, 0.02 mM, 0.03 mM, 0.04 mM, 0.05 mM, 0.06 mM, 0.07 mM, 0.08 mM, 0.09 mM, 0.1 mM)

Excitation wavelength: 340 nm; Excitation slit: 5 nm; Emission slit: 5 nm

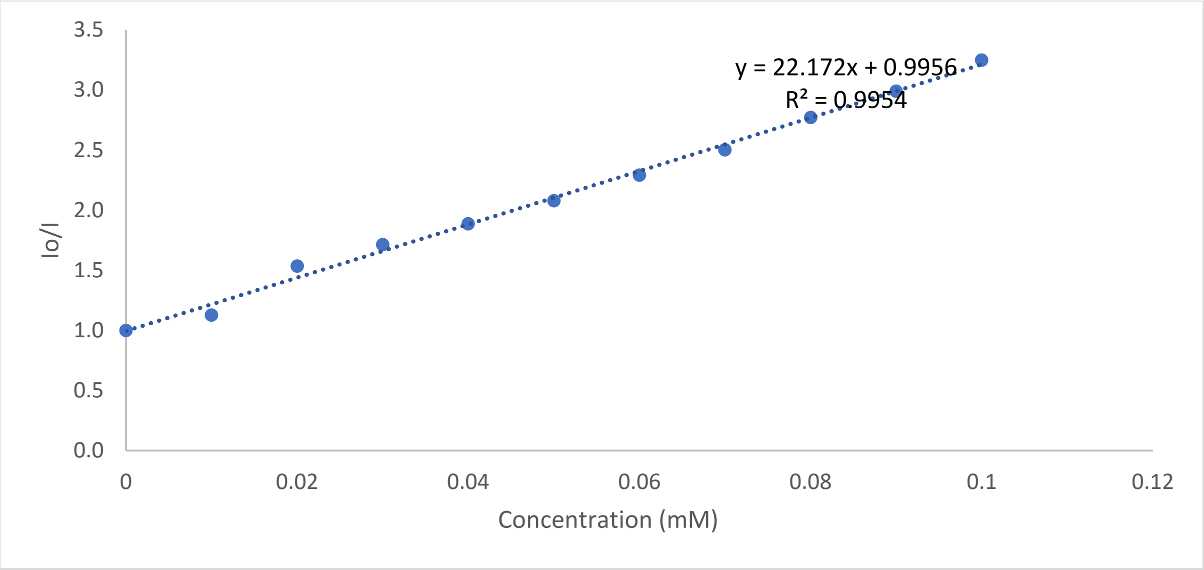

Figure S8: Stern-Volmer for photocatalyst (4CzIPN) quenching with Co-salen$^{(tBu\text{-}tBu)}$

The Co-salen complex has absorption in the visible light region that does overlap insignificantly with the emission region of 4CzIPN at 471 nm, therefore the inner-filter effect of Co-salen, the quenching ratio I_o/I has not been applied to correct the Stern-Volmer plot using the following formula:

$$\left(\frac{I_o}{I}\right) correction = \left(\frac{\text{Abs}(4CzIPN)}{\text{Abs }(4CzIPN)+\text{Abs}(Co)}\right)\left(\frac{1\text{-}10^{-(\text{Abs}(4CzIPN)+\text{Abs}(Co))}}{1\text{-}10^{-\text{Abs}(4CzIPN)}}\right) \qquad \text{(Eq.1)}$$

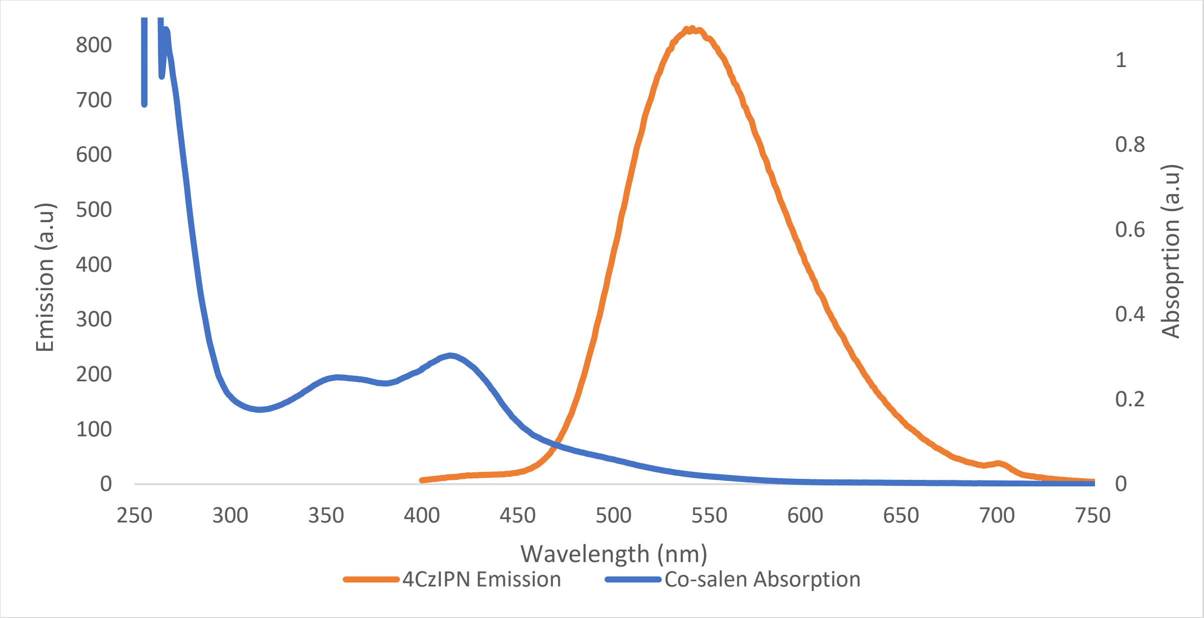

Figure S9: UV-Vis absorption of Co-salen$^{(tBu\text{-}tBu)}$ and fluorescence emission of 4CzIPN in DMF.

Quenching of 4CzIPN with Hantzsch ester

To a standard solution of 1,2,3,5-Tetrakis(carbazol-9-yl)-4,6-dicyanobenzene (4CzIPN) (0.1 mM in degassed DMF) different amounts of standard solution of Hantzsch ester (1 mM in degassed DMF) were added to afford the final concentrations reported in the table below then the fluorescence of each entry was measured (quencher concentration; [HEH] = 0.00 mM, 0.01 mM, 0.02 mM, 0.03 mM, 0.04 mM, 0.05 mM, 0.06 mM, 0.07 mM, 0.08 mM, 0.09 mM, 0.1 mM)

Excitation wavelength: 340 nm; Excitation slit: 5 nm; Emission slit: 5 nm

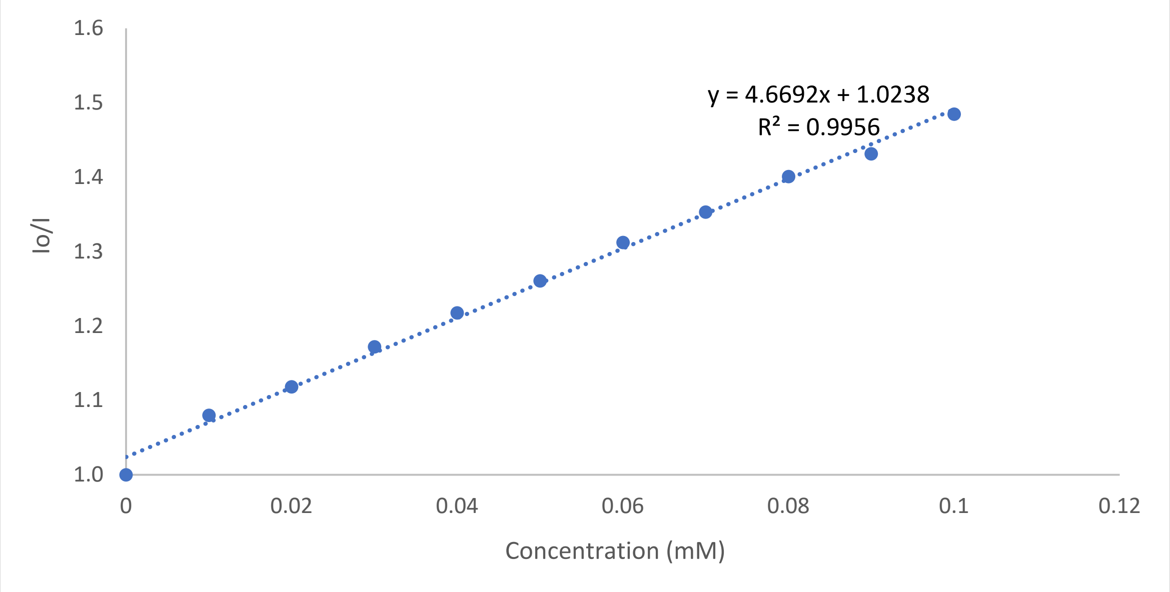

Figure S10: Stern-Volmer for photocatalyst (4CzIPN) quenching with Hantzsch ester (HEH)

Quenching of 4CzIPN with ethene-1,1-diyldibenzene

To a standard solution of 1,2,3,5-Tetrakis(carbazol-9-yl)-4,6-dicyanobenzene (4CzIPN) (0.1 mM in degassed DMF) different amounts of standard solution of model substrate (1 mM in degassed DMF) were added to afford the final concentrations reported in the table below then the fluorescence of each entry was measured, (quencher concentration; [SM] = 0.00 mM, 0.01 mM, 0.02 mM, 0.03 mM, 0.04 mM, 0.05 mM, 0.06 mM, 0.07 mM, 0.08 mM, 0.09 mM, 0.1 mM)

Excitation wavelength: 340 nm; Excitation slit: 5 nm; Emission slit: 5 nm

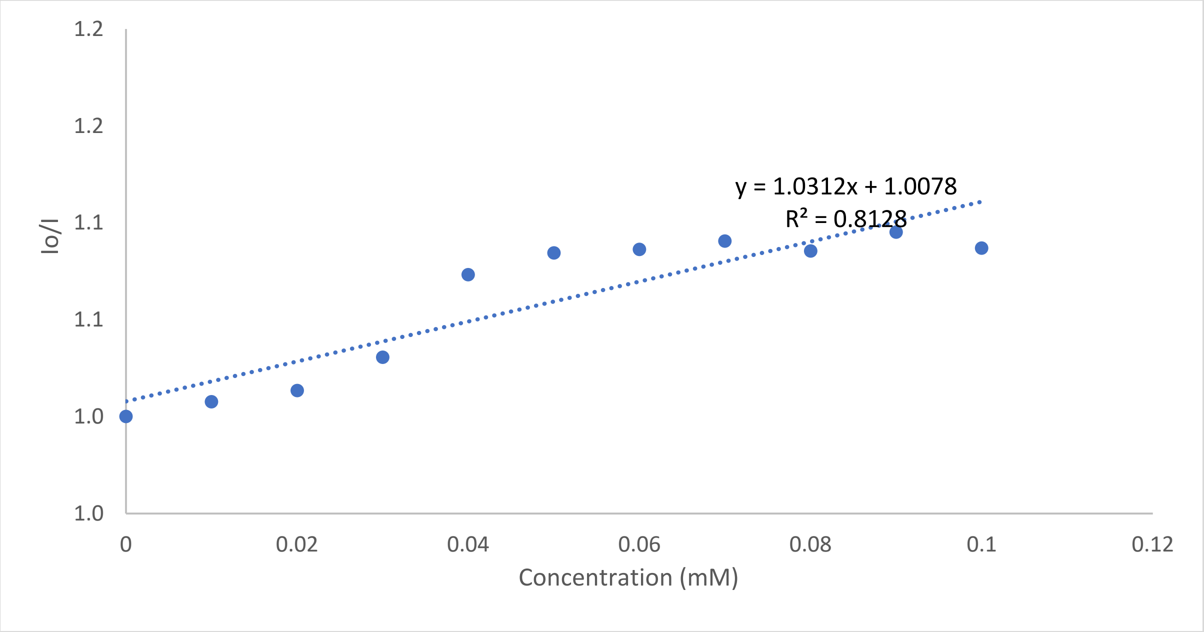

Figure S11: Stern-Volmer for photocatalyst (4CzIPN) quenching experiment with Ethene-1,1-diyldibenzene.

From the quenching experiment it is observed that the model substrate does not have significant quenching effect on 4CzIPN due to the last entries maintaining the same emission ratio.

Combination of quenching experiments for 4CzIPN

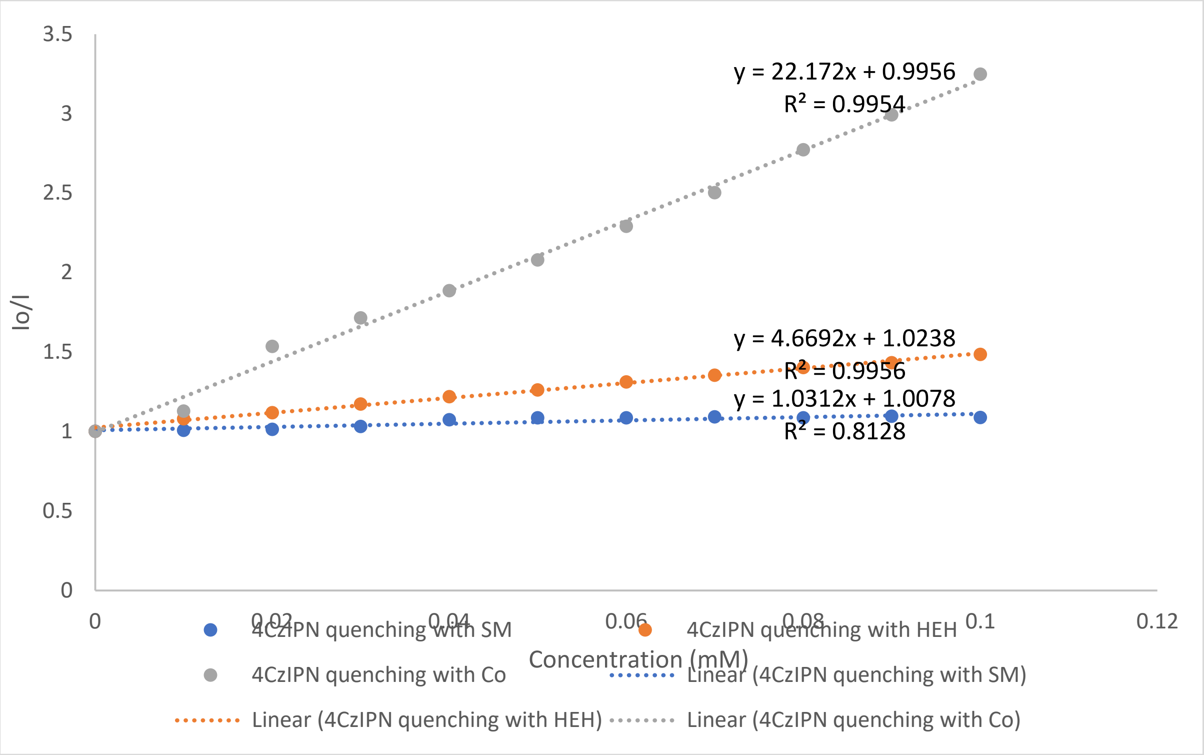

Figure S12: Stern-Volmer for photocatalyst (4CzIPN) quenching experiment with Ethene-1,1-diyldibenzene (SM), with Hantzsch ester (HEH) and Co-salen$^{(tBu\text{-}tBu)}$ (Co).

5.5.2. 2,3,4,6-Tetra(9*H*-carbazol-9-yl)-5-methylbenzonitrile (4CzMeBN)

Quenching of 4CzMeBN with Co-salen

To a standard solution of 5.4.2. 2,3,4,6-Tetra(9*H*-carbazol-9-yl)-5-methylbenzonitrile (4CzMeBN) (0.1 mM in degassed DMF) different amounts of standard solution of Co-salen (1 mM in degassed DMF) were added to afford the final concentrations reported in the table below then the fluorescence of each entry was measured, (quencher concentration; [Co] = 0.00 mM, 0.01 mM, 0.02 mM, 0.03 mM, 0.04 mM, 0.05 mM, 0.06 mM, 0.07 mM, 0.08 mM, 0.09 mM, 0.1 mM)

Excitation wavelength: 340 nm; Excitation slit: 5 nm; Emission slit: 5 nm

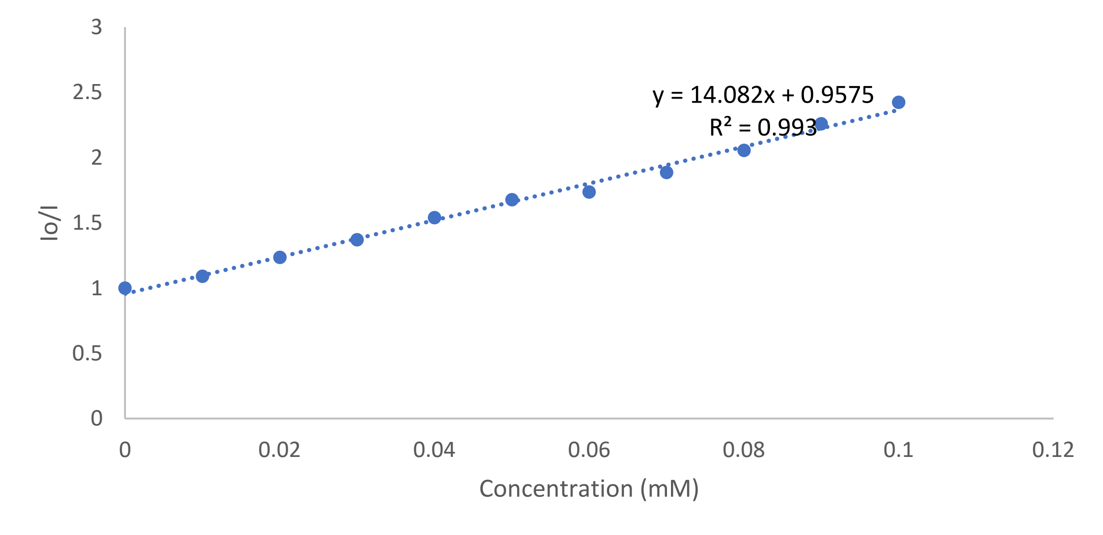

Figure S13: Stern-Volmer for photocatalyst (4CzMeBN) quenching with Co-salen$^{(t\text{Bu-}t\text{Bu})}$ prior correction.

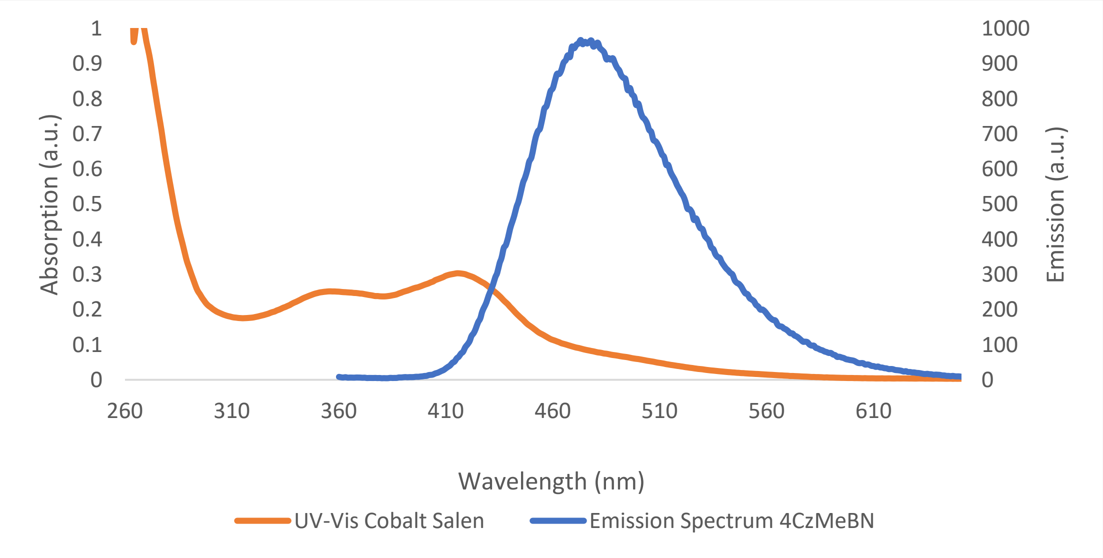

Figure S14:UV-vis absorption spectrum of Co-salen$^{(t\text{Bu-}t\text{Bu})}$ shows overlap with emission spectrum of photocatalyst (4CzMeBN). Significant overlap indicates the requirement of inner-filter effect.

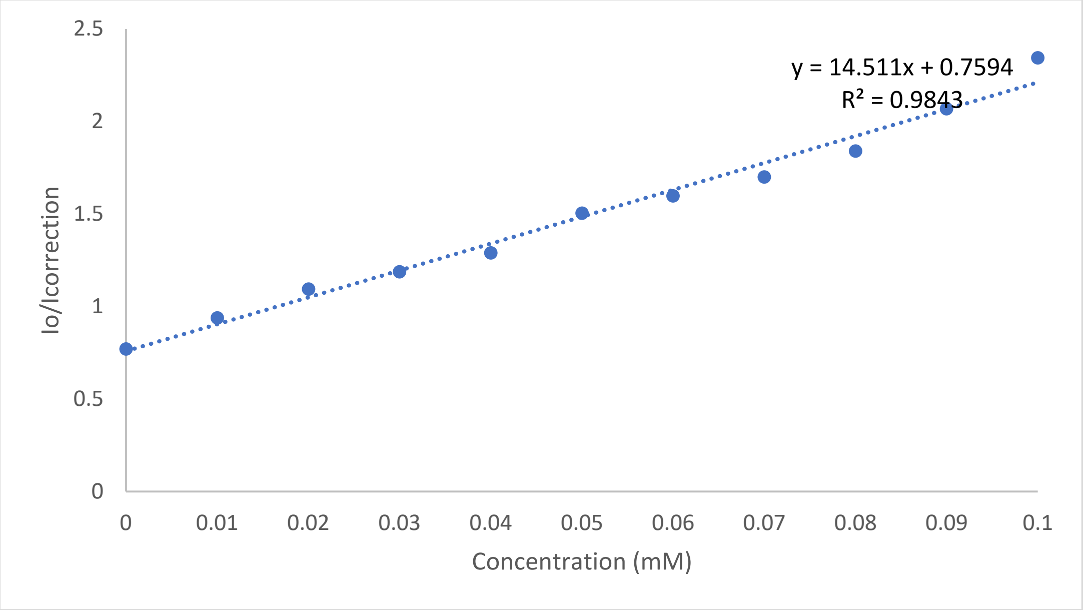

Figure S15: Stern-Volmer for photocatalyst (4CzMeBN) quenching with Co-salen$^{(tBu\text{-}tBu)}$ after correction.

Quenching of 4CzMeBN with Hantzsch ester

To a standard solution of 5.4.2. 2,3,4,6-Tetra(9*H*-carbazol-9-yl)-5-methylbenzonitrile (4CzMeBN) (0.1 mM in degassed DMF) different amounts of standard solution of HEH (1 mM in degassed DMF) were added to afford the final concentrations reported in the table below then the fluorescence of each entry was measured, (quencher concentration; [HEH] = 0.00 mM, 0.01 mM, 0.02 mM, 0.03 mM, 0.04 mM, 0.05 mM, 0.06 mM, 0.07 mM, 0.08 mM, 0.09 mM, 0.1 mM)

Excitation wavelength: 340 nm; Excitation slit: 5 nm; Emission slit: 5 nm

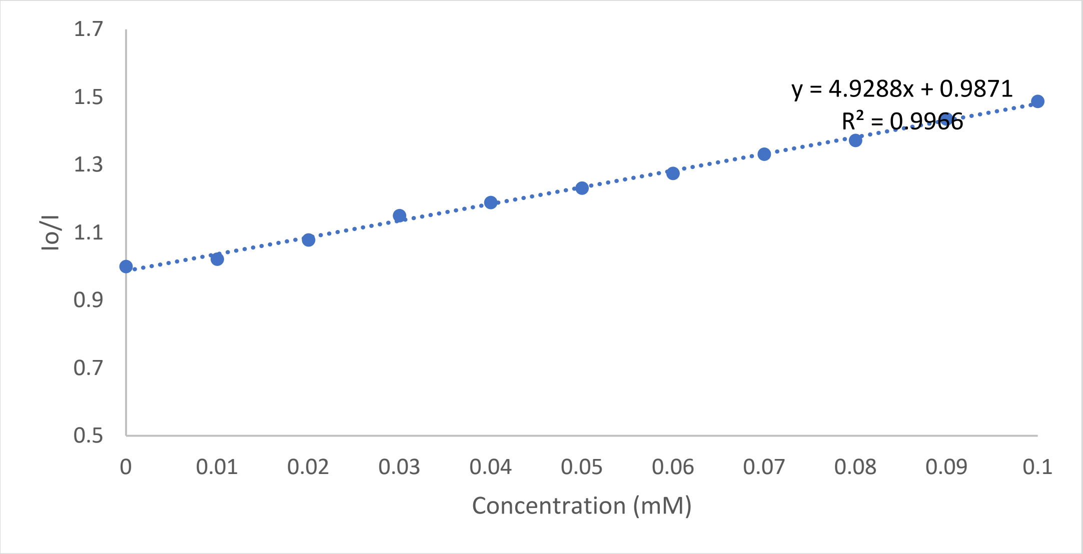

Figure S16: Stern-Volmer for photocatalyst (4CzMeBN) quenching with Hantzsch ester.

Quenching of 4CzMeBN with ethene-1,1-diyldibenzene

To a standard solution of 5.4.2. 2,3,4,6-Tetra(9*H*-carbazol-9-yl)-5-methylbenzonitrile (4CzMeBN) (0.1 mM in degassed DMF) different amounts of standard solution of HEH (1 mM in degassed DMF) were added to afford the final concentrations reported in the table below then the fluorescence of each entry was measured, (quencher concentration; [HEH] = 0.00 mM, 0.01 mM, 0.02 mM, 0.03 mM, 0.04 mM, 0.05 mM, 0.06 mM, 0.07 mM, 0.08 mM, 0.09 mM, 0.1 mM)

Excitation wavelength: 340 nm; Excitation slit: 5 nm; Emission slit: 5 nm

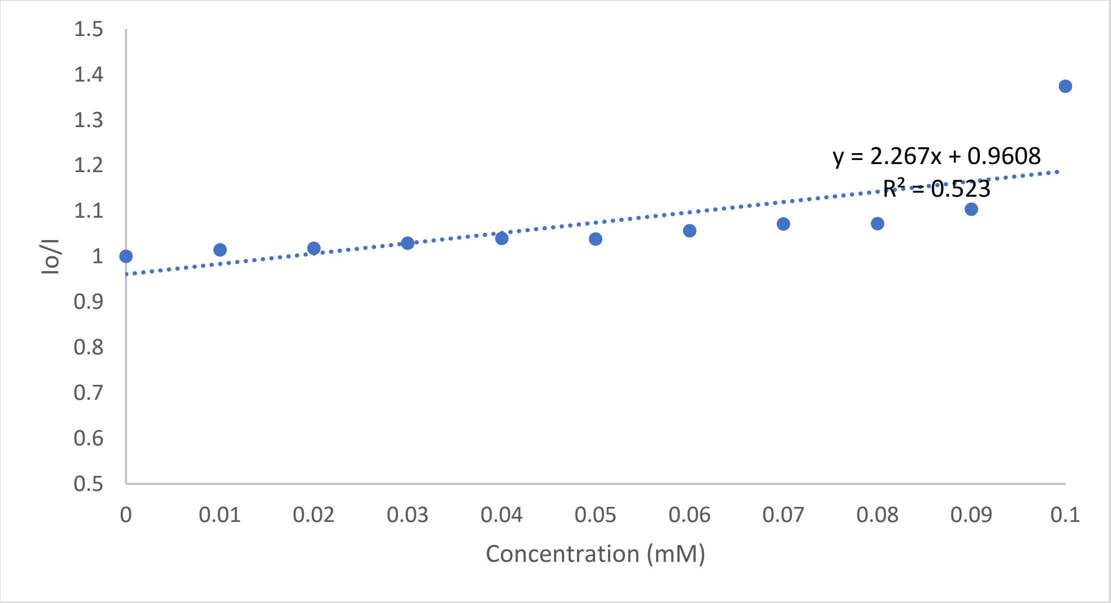

Figure S17: Stern-Volmer for photocatalyst (4CzMeBN) quenching with ethene-1,1-diyldibenzene.

Combination of quenching experiments for 4CzMeBN

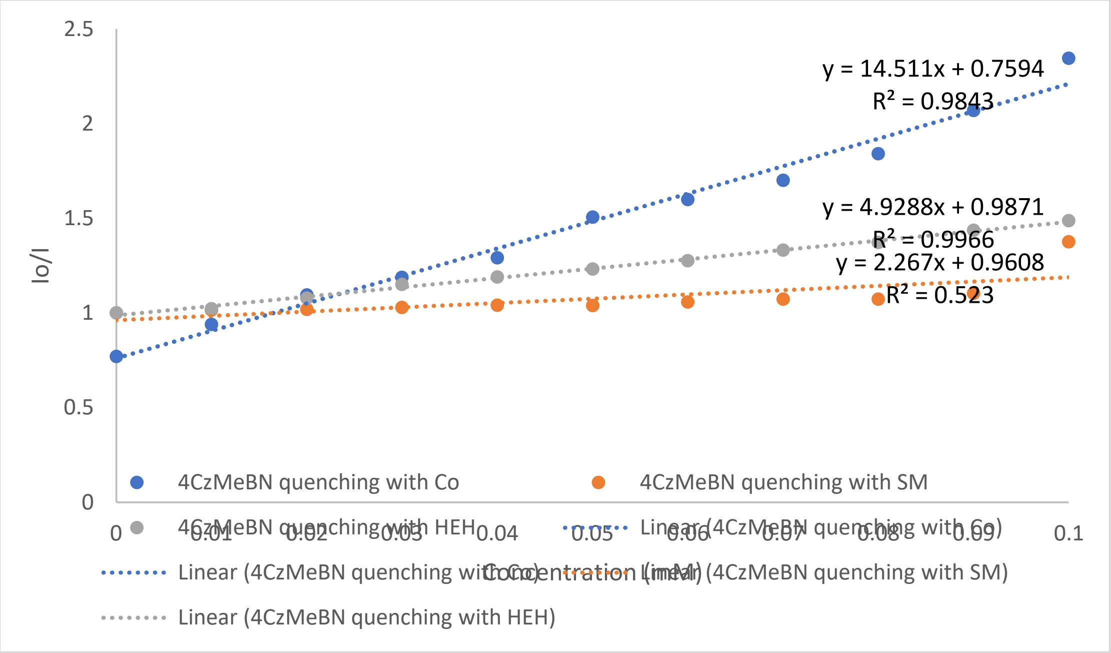

Figure S18: Stern-Volmer for photocatalyst (4CzMeBN) quenching experiment with ethene-1,1-diyldibenzene (SM), with Hantzsch ester (HEH) and Co-salen$^{(tBu-tBu)}$ (Co).

5.6. Cyclic voltammetry experiment and redox potentials

Relevant redox potentials

Table S1: Summary of relevant redox potentials of involved chemical species

	E_{red} (V vs. SCE)	E_{ox} (V vs. SCE)	*E_{red} (V vs. SCE)	*E_{ox} (V vs. SCE)	Reference
4CzIPN	-1.21	+1.52	+1.38 ^{t}; + 1.43 ^{s}	-1.07^{t}; -1.12^{s}	[43]
Hantzsch ester	+1.00	+0.79	-2.28	-	[44]
Co-salen$^{(tBu\text{-}tBu)}$	-1.60	-	-	-	[45]
Ethene-1,1-diyldibenzene	+ 1.54	-	-	-	[46]

^{t}Data for triplet excited state. ^{S}Data for singlet excited state.

Cyclic voltammetry (CV) of 2,3,4,6-Tetra(9*H*-carbazol-9-yl)-5-methylbenzonitrile (4CzMeBN)

Ossila Potentiostat electrochemical measurement device was used to perform cyclic voltammetry.

Anhydrous *N*,*N*-dimethylformamide (DMF) was degassed using freeze pump thaw technique in three cycles. Reference electrode : non-aqueous Ag/Ag^{+}. A solution of 10 mM of silver nitrite in degassed DMF was added into the reference electrode tube with the help of a syringe and needle until the tube is approximately 2/3 full. Working electrode: Platinum disc -2 mm diameter. Counter electrode: Platinum wire – 0.5 mm diameter. The concentration of 2,3,4,6-Tetra(9*H*-carbazol-9-yl)-5-methylbenzonitrile (4CzMeBN): 1 mM in DMF. The concentration of internal standard (ferrocene): 1 mM in DMF. A 0.1 M solution of tetrabutylammoniumtetrafluoroborate (TBATFB) in degassed DMF as supporting electrolyte.

Experimental parameters:

Start Potential (V): -0.1; Potential Vertex 1 (V): 1.3; Potential Vertex 2 (V): -3.2; Cycles: 2; Scan Rate (mV/s): 100

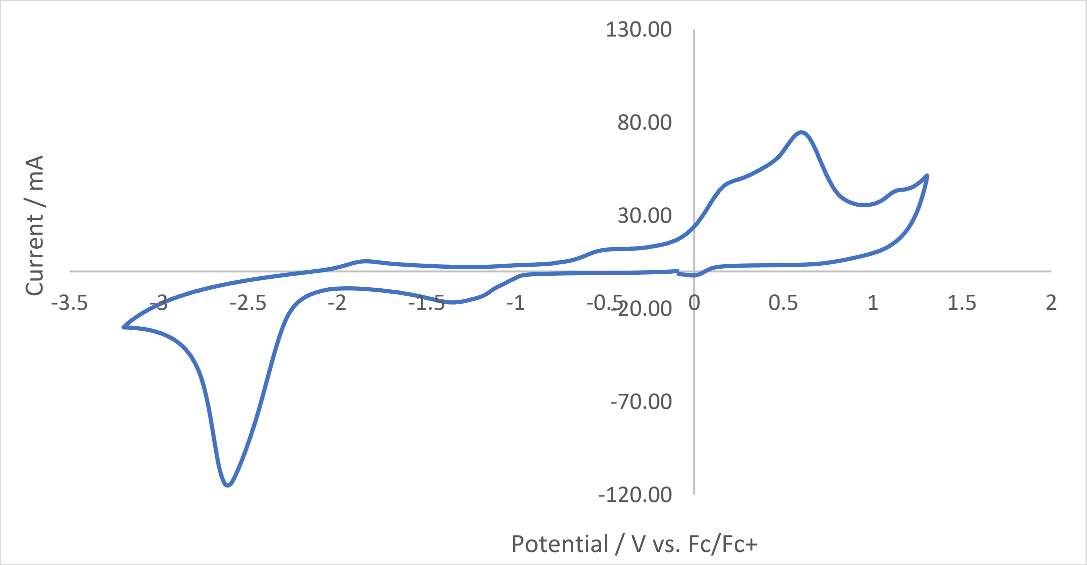

Figure S18: Reductive half of cyclic voltammogram of 2,3,4,6-Tetra(9*H*-carbazol-9-yl)-5-methylbenzonitrile (4CzMeBN) with ferrocene as internal standard. No reversible oxidation of 4CzMeBN in the stability window of DMF. Possible irreversible reduction below -3.0 V. However, no reversible oxidation in DMF.

Reversible reduction at $E_{1/2}$ = -2.1 V vs. *Fc/Fc*$^+$ in *N,N*-dimethylformamide (DMF) which is equivalent to – 1.65 V vs. SCE.

The $^*E_{red}$ (V vs. SCE) was calculated using the following formula:

$$E^{*}red = Ered + E_{0\text{-}0} \qquad \text{(Eq.2)}$$

where $E_{0\text{-}0}$ (eV) values were calculated by the wavelength at the intercept of emission and adsorption and the associated energy. [43]

$$E_{0\text{-}0} = \frac{1240}{\lambda} \qquad \text{(Eq.3)}$$

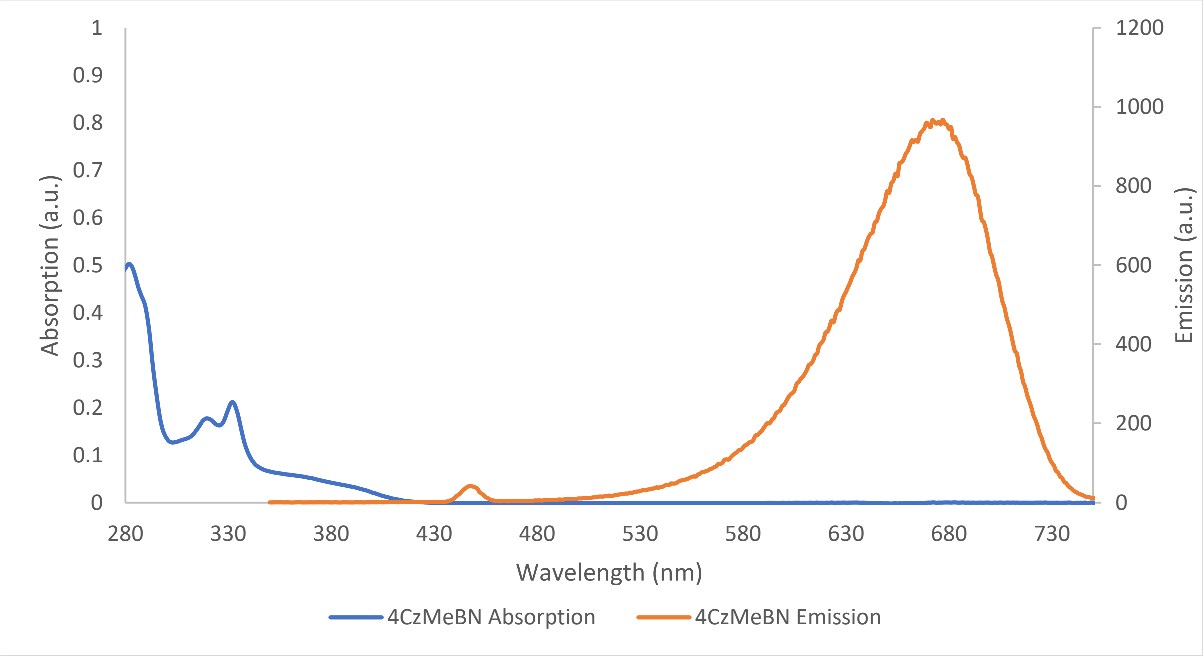

Figure S19: UV-Vis adsorption and fluorescence emission of photosubstituted photocatalyst (4CzMeBN) in DMF.

The experimental analysis of 4CzMeBN in *N,N*-dimethylformamide (DMF) provides $E_{0\text{-}0}$ is 3.08 eV. In the DMF the $^*E_{red}$ is approximately + 1.43 V vs. *SCE*. Possibly irreversible reduction of 4CzMeBN below –3.0 V vs. Fc/Fc$^+$ in DMF which is equivalent to –2.55 V vs. *SCE*.

5.7. Radical ring opening

2-(4-(trifluoromethyl)phenyl)hex-4-enoic acid **(2an)**

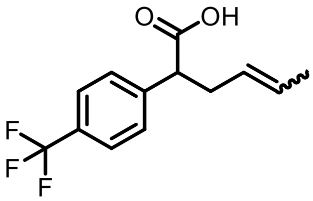

Prepared from 0.1 mmol (21.2 mg) of vinylcyclopropane, the product is isolated as a light-brown coloured oil (12.7 mg, 0.05 mmol, 63 %,) as a mixture of *cis*:*trans* = 42:58 stereoisomers and traces of impurities (H grease, pump oil, silicon grease) that eluted with the product. The NMR yield is 94 % using dibromomethane as an internal standard.

^{1}H NMR (600 MHz, $CDCl_3$): δ 7.59 – 7.57 (m, 2H), 7.47 – 7.42 (m, 2H), 5.54 – 5.47 (m, 1H), 5.34 – 5.26 (m, 1H), 3.68 (m, 1H), 2.87 – 2.81 (m, 1H), 2.79 – 2.73 (m, 1H), 2.58 – 2.52 (m, 1H), 2.49 – 2.43 (m,1H), 1.60 (d, *J* = 5.4 Hz, 3H), 1.56 (d, *J* = 6.3 Hz, 3H). **^{13}C NMR** (151 MHz, $CDCl_3$): δ 178.3, 130.2 (q, *J* = 32.5 Hz), 128.7 (m), 127.1, 125.7 (q, *J* = 3.5 Hz), 124.7 (q, *J* = 272.3 Hz) 52.2, 36.5, 31.3, 18.3, 13.2. **^{19}F NMR** (565 MHz, $CDCl_3$): δ - 62.7 (s, 3F), - 62.7 (s, 3F) **HRMS (EI)**: **(*m/z*)** [M–H$^+$]: calculated for $[C_{13}H_{12}O_2F_3]^+$: 257.0789, found 257.0794. **IR (neat)** ν = 2961, 1713, 1413, 1324, 1259, 1083, 1023 cm^{-1}

Note: 2D NMR HSQC, HMBC, COSY was used to assign ^{13}C NMR for the isolated product.

6. Spectra

Starting materials

Isopropyl 2-(4-(1-(4-chlorophenyl)vinyl)phenoxy)-2-methylpropanoate (1ae)

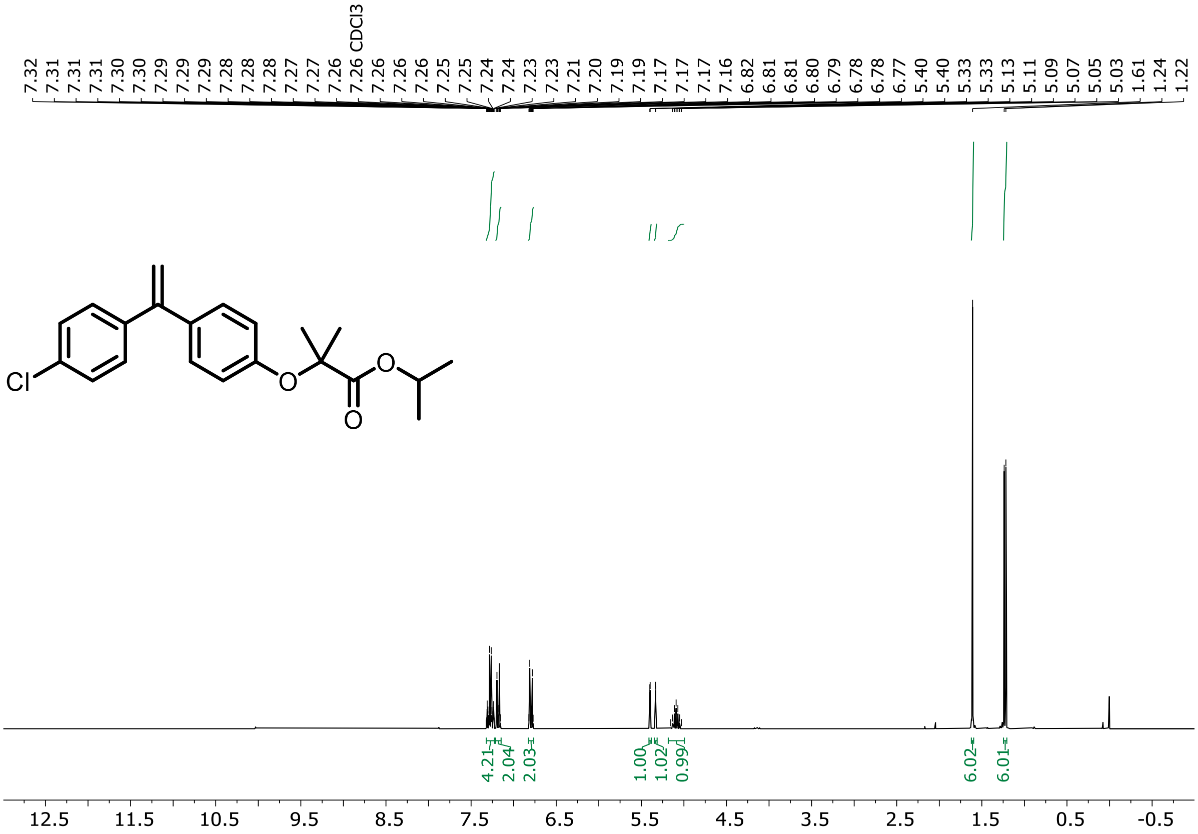

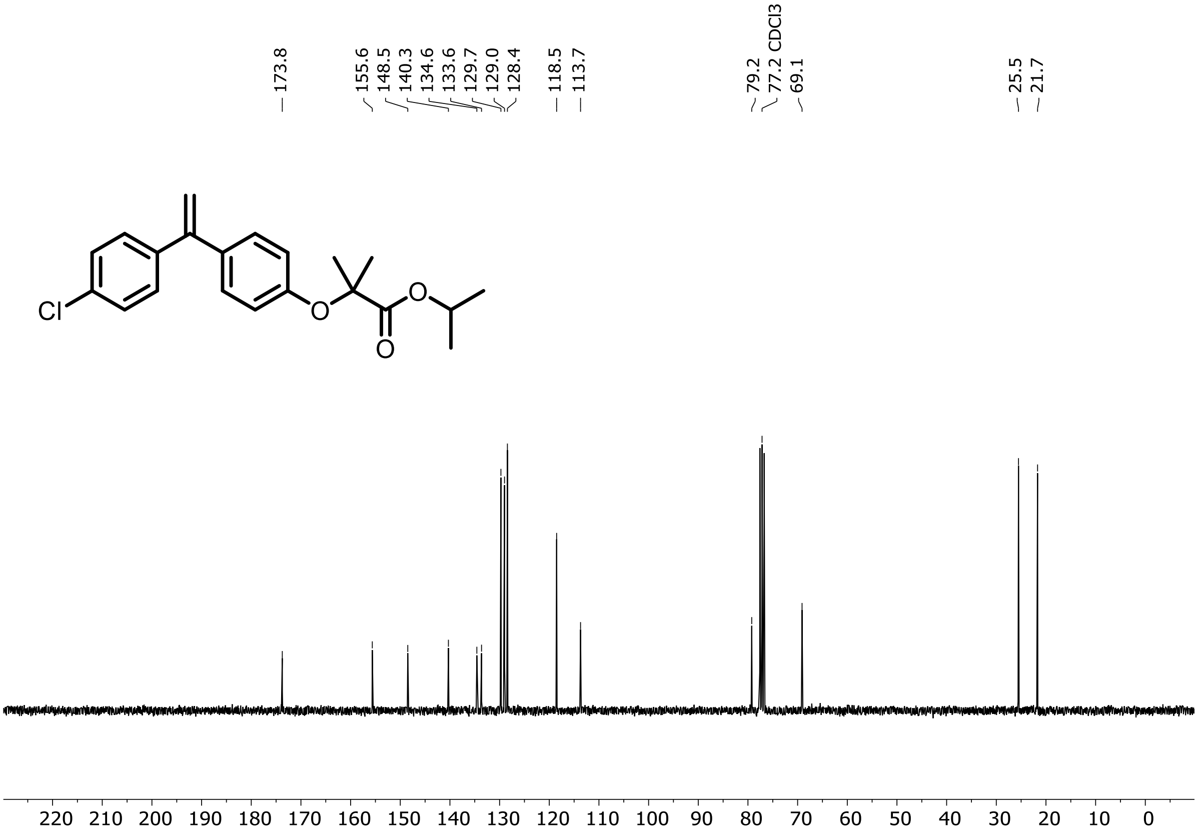

Ethyl 2-(3-benzoylphenyl)propanoate (1af’)

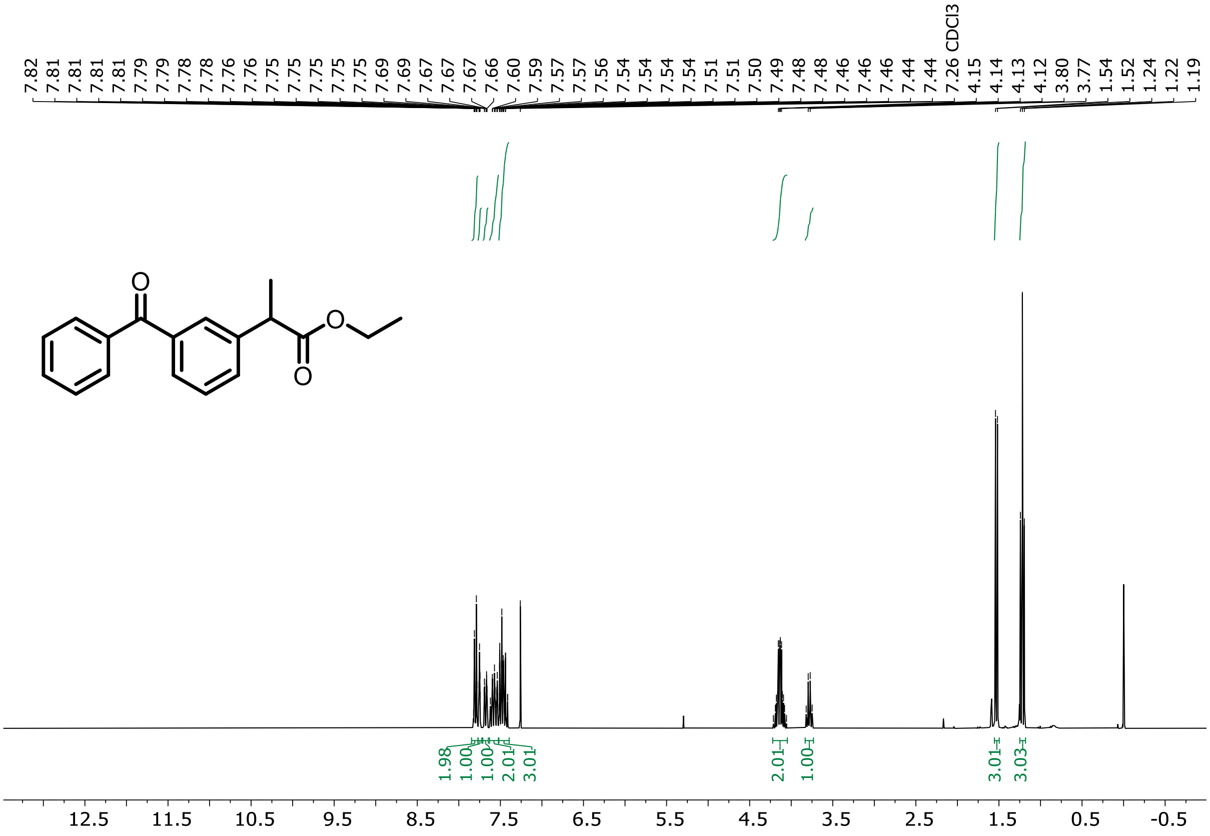

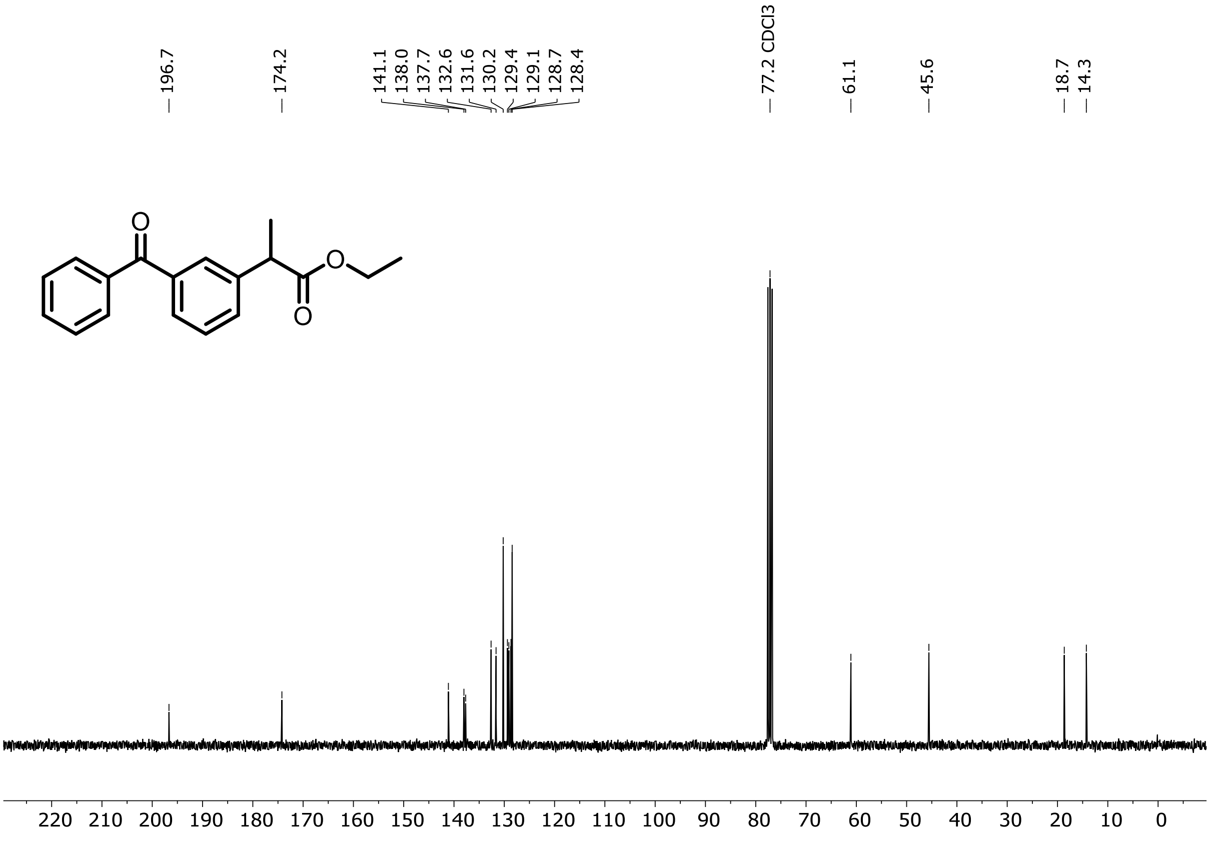

Ethyl 2-(3-(1-phenylvinyl)phenyl)propanoate (1af)

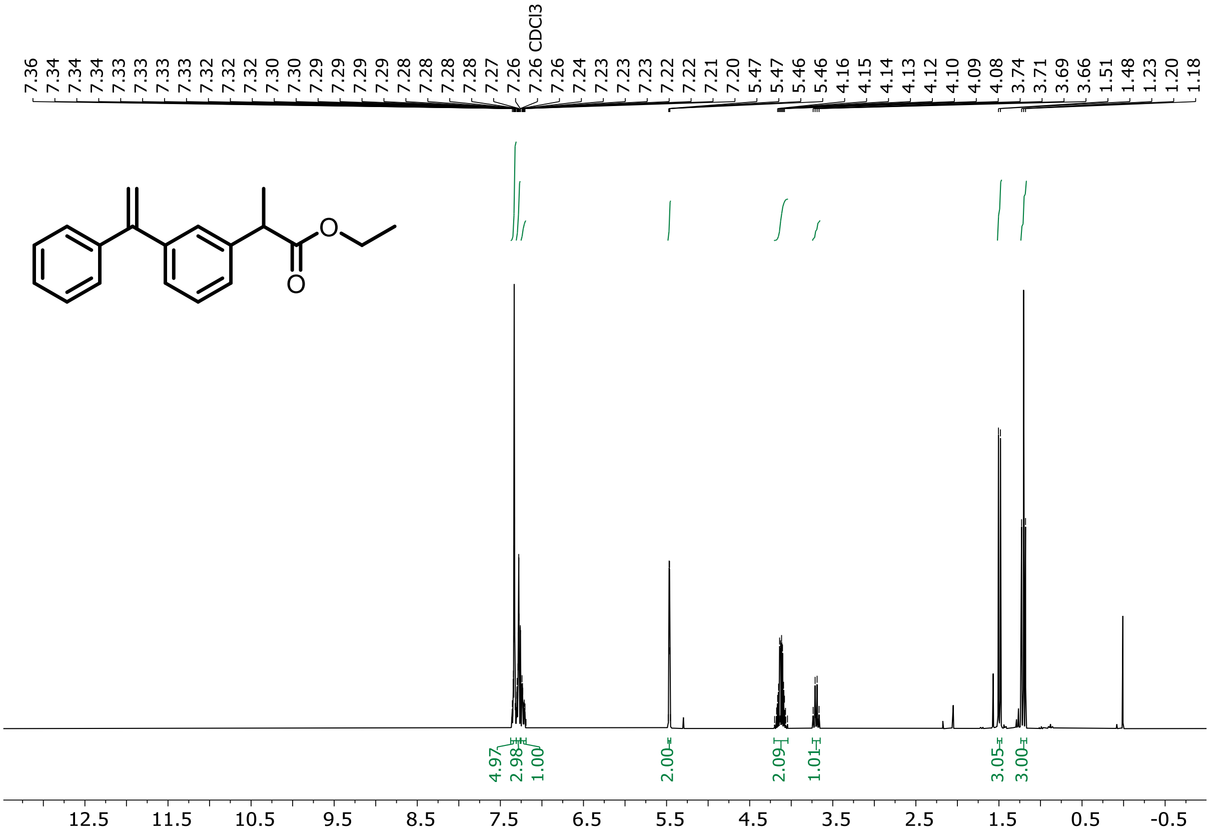

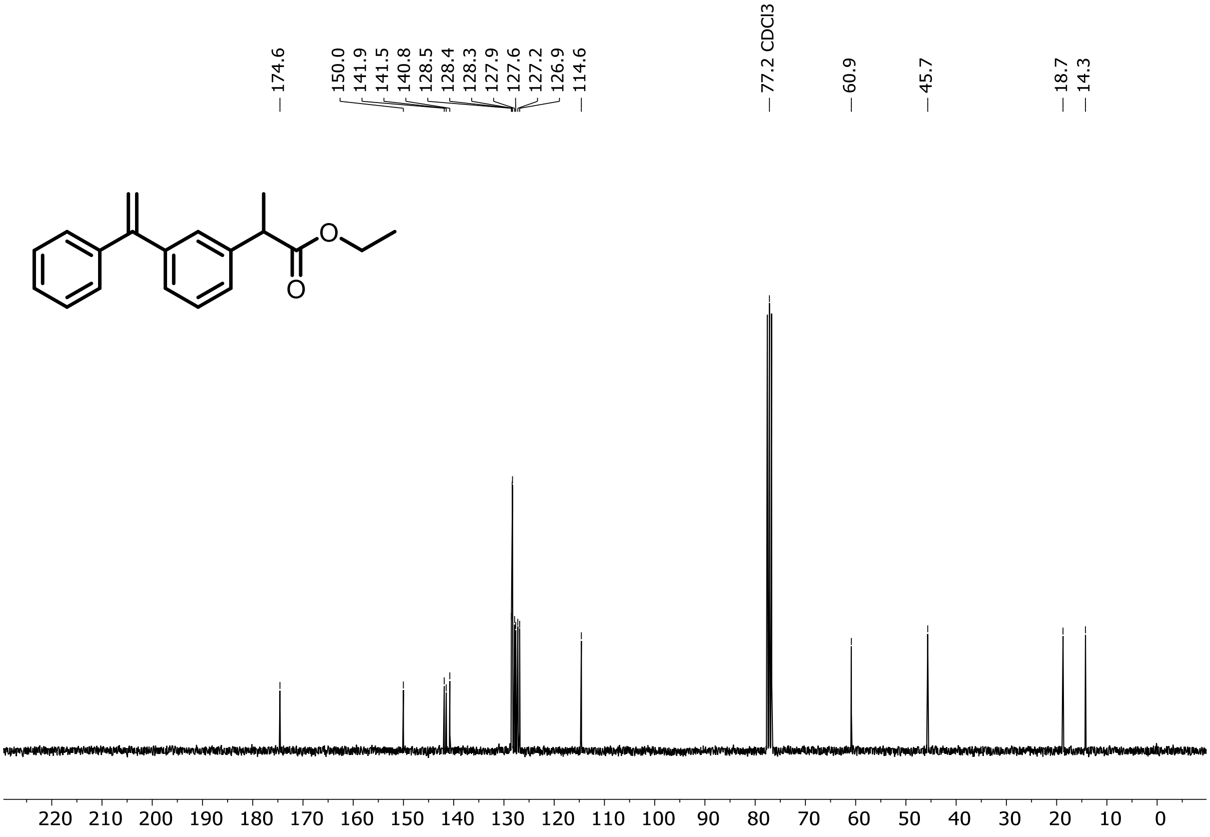

1-(trifluoromethyl)-4-(2-vinylcyclopropyl)benzene (**1an**)

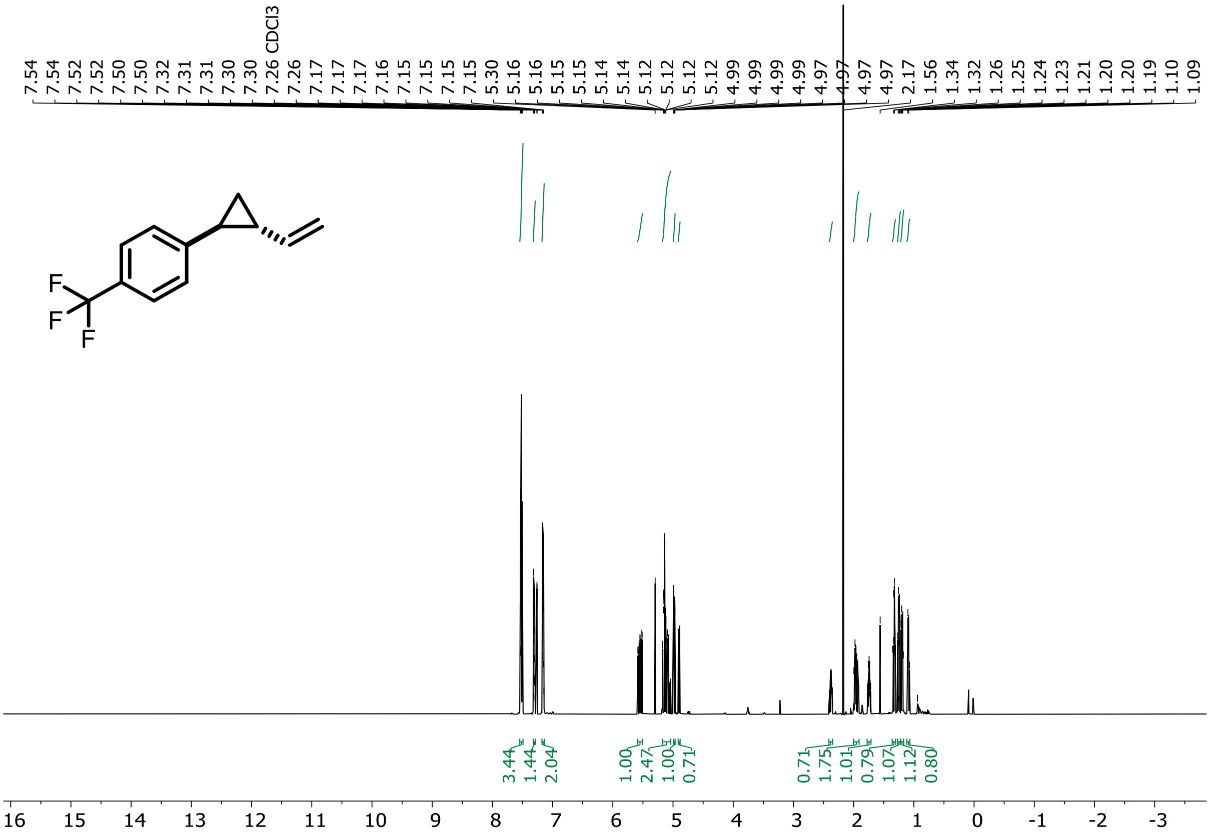

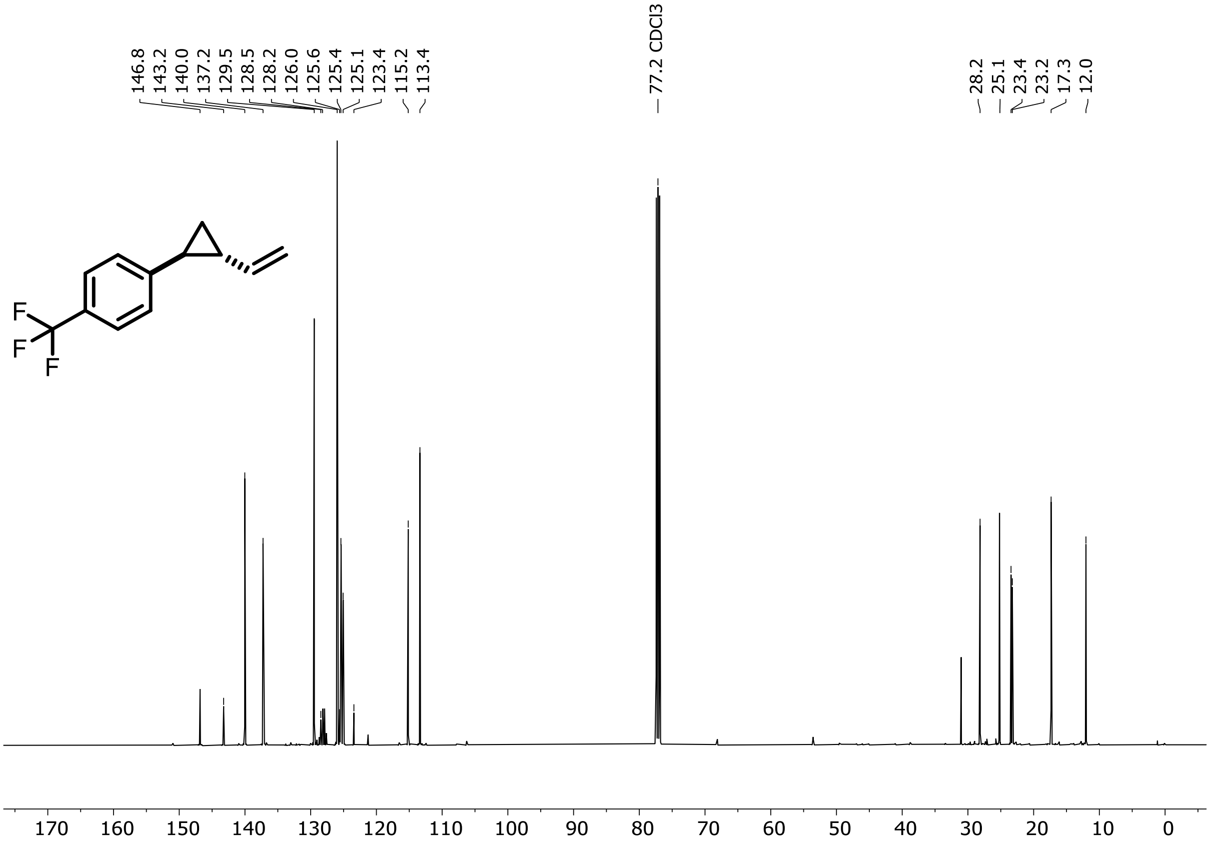

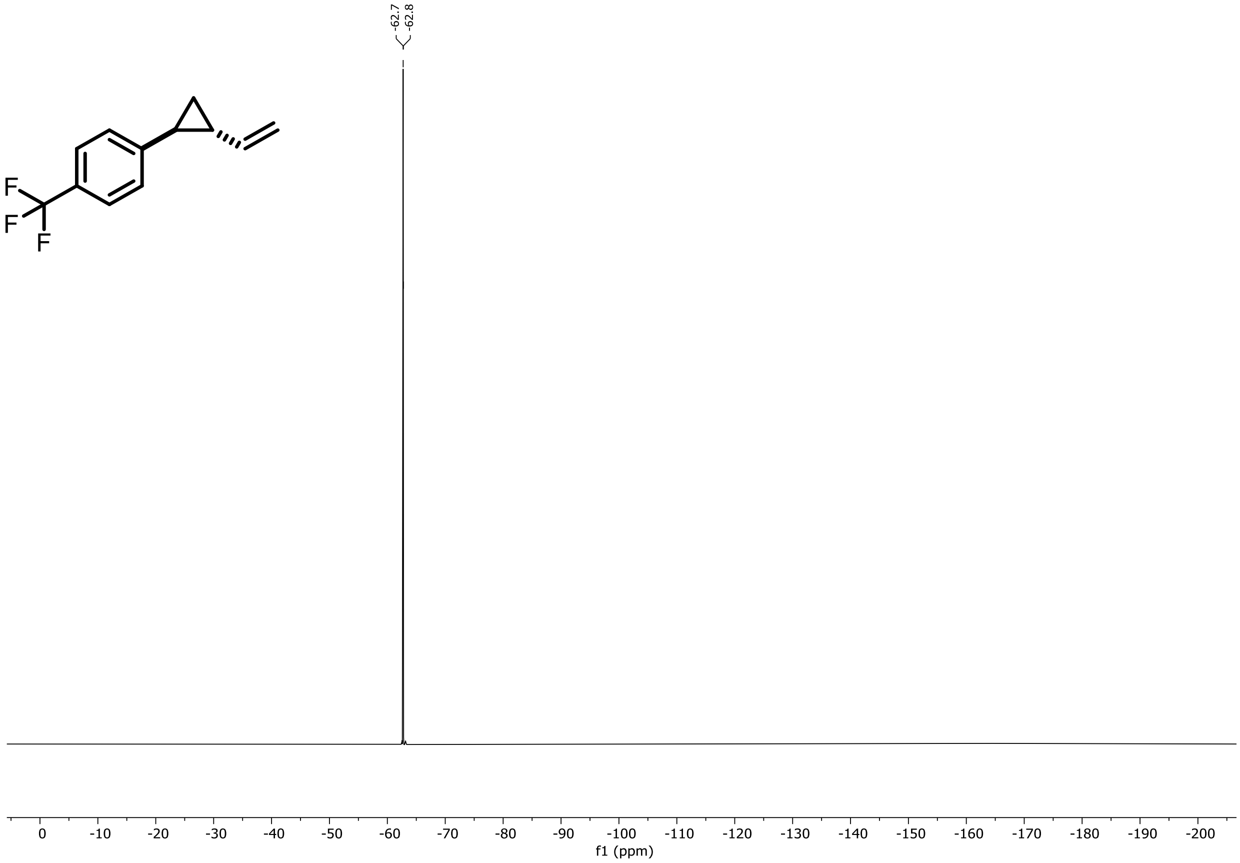
-62.7
-62.8
F
F
F
0 -10 -20 -30 -40 -50 -60 -70 -80 -90 -100 -110 -120 -130 -140 -150 -160 -170 -180 -190 -200
f1 (ppm)

Substrate scope

2-(4-cyanophenyl)propanoic acid (2a)

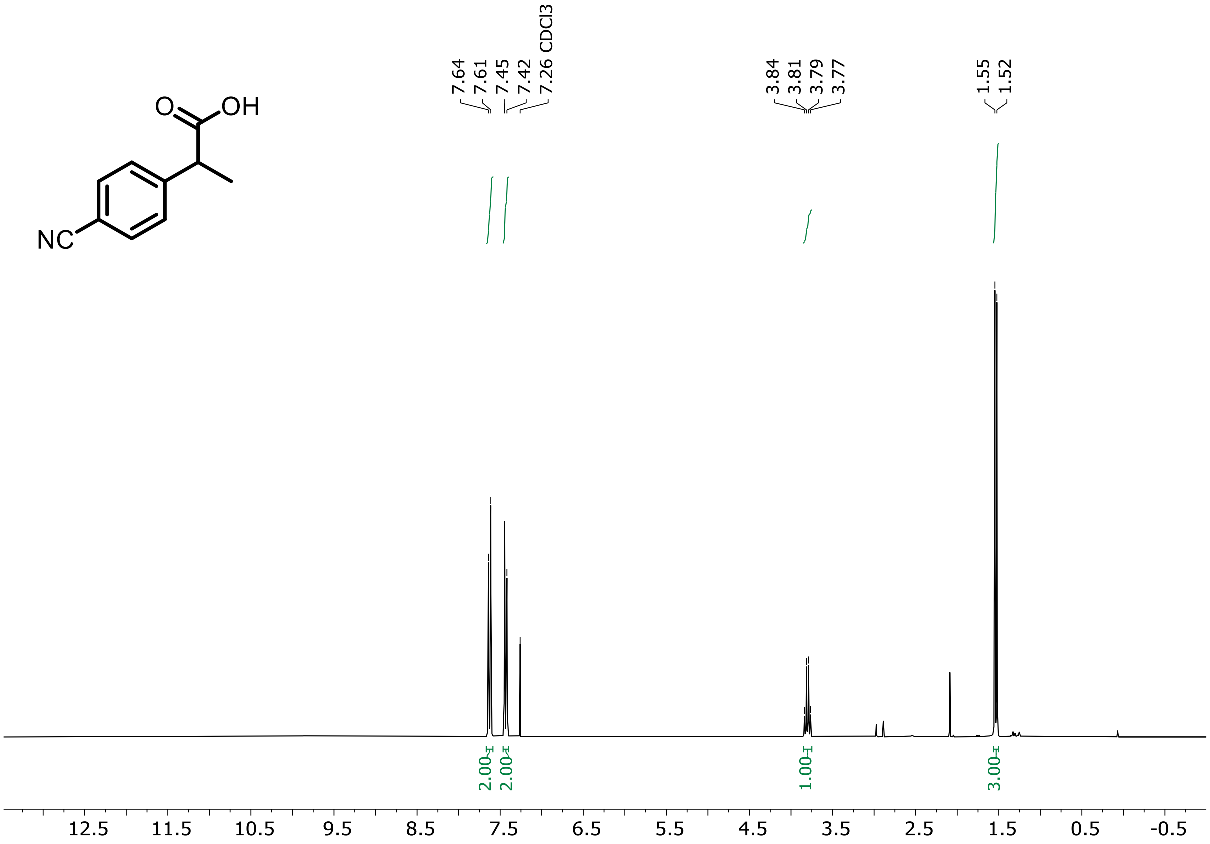

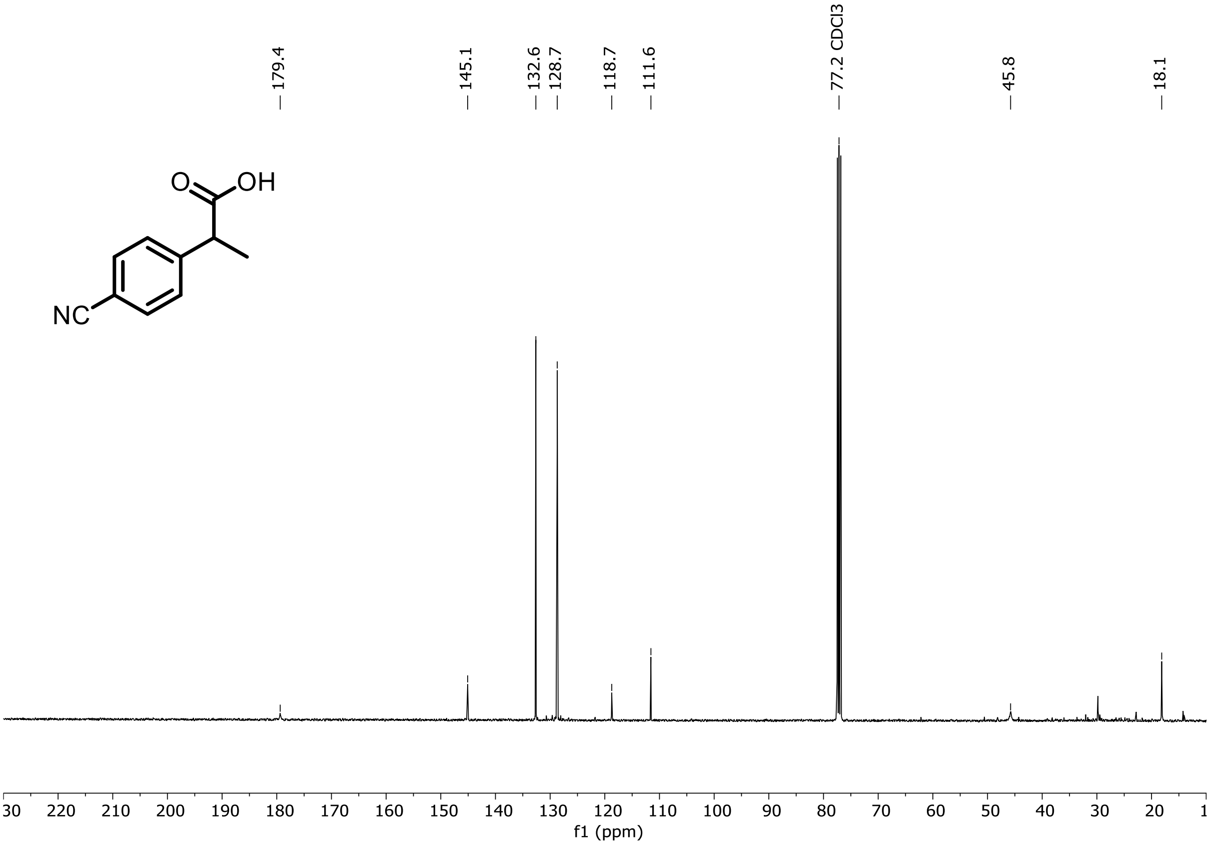

2-(4-(trifluoromethyl)phenyl)propanoic acid (2b)

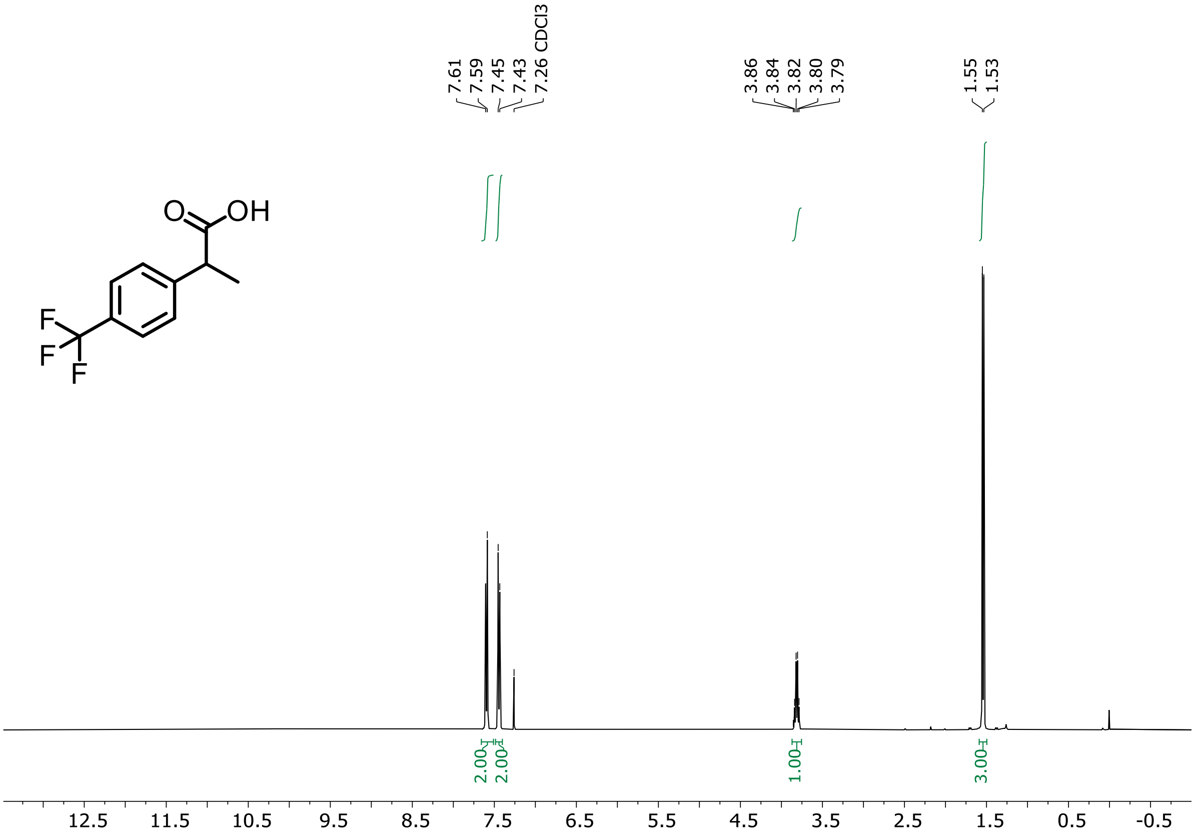

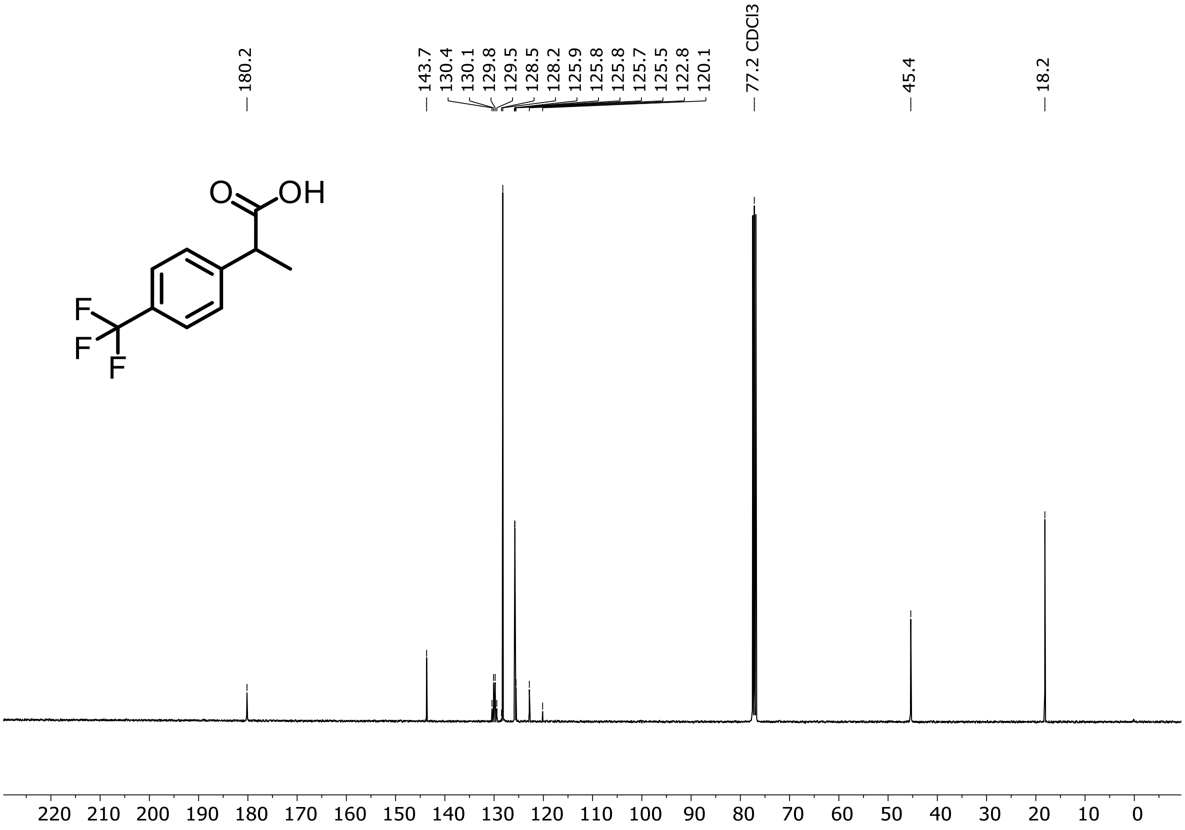

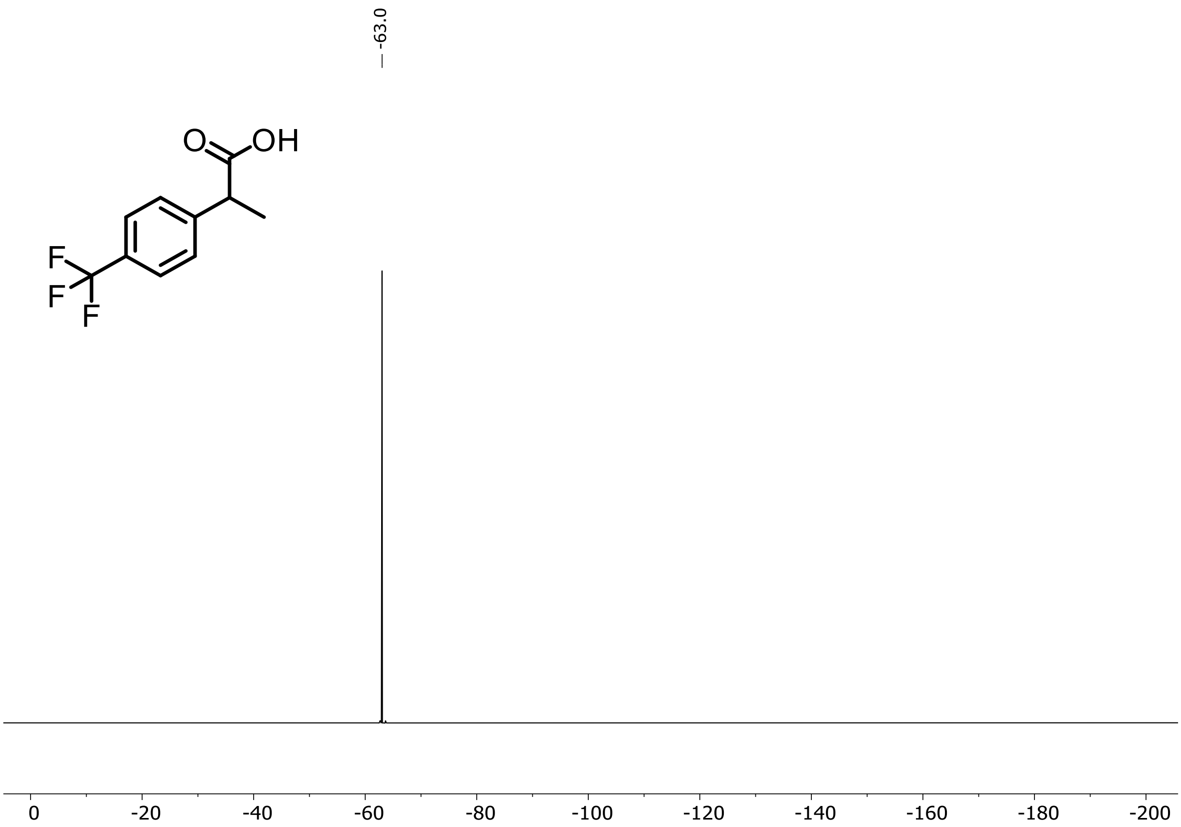
-63.0
O
OH
F
F
F
0
-20
-40
-60
-80
-100
-120
-140
-160
-180
-200

2-(4-(methylsulfonyl)phenyl)propanoic acid (2c)

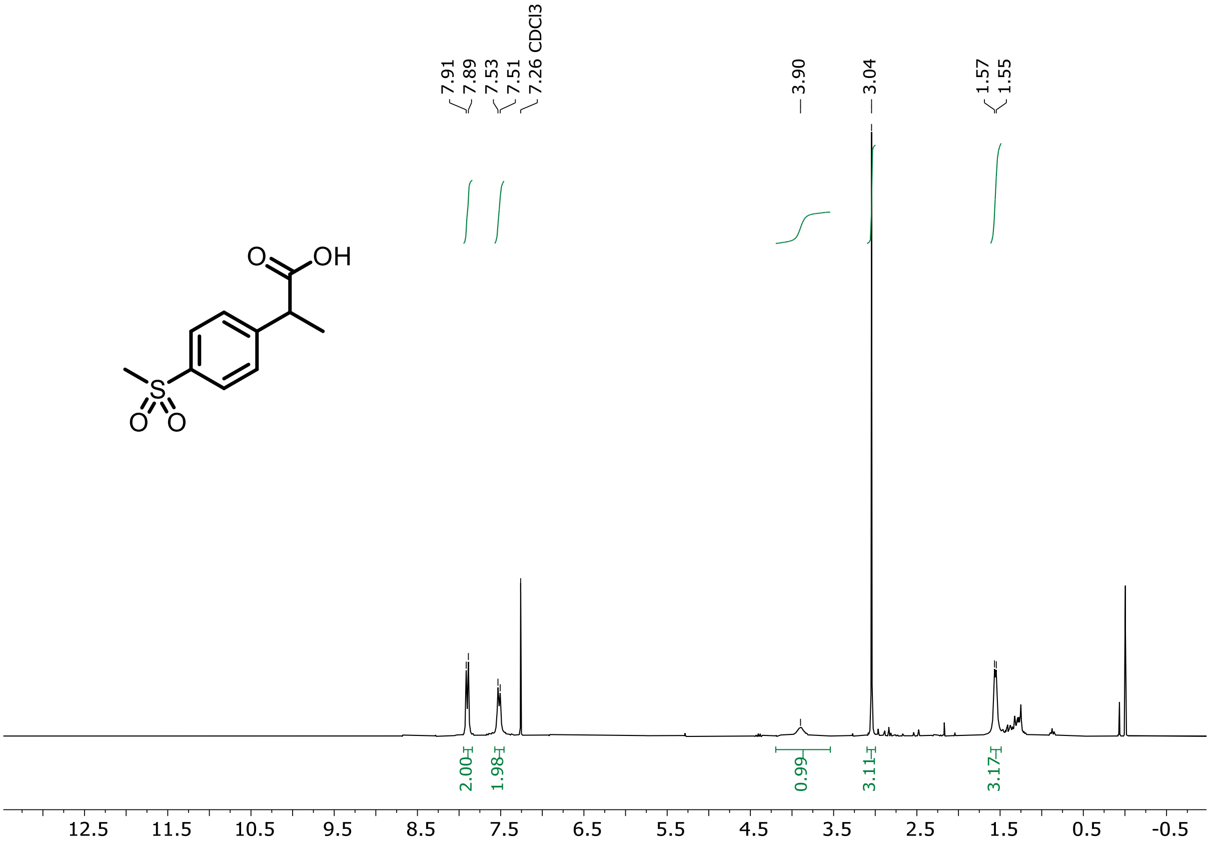

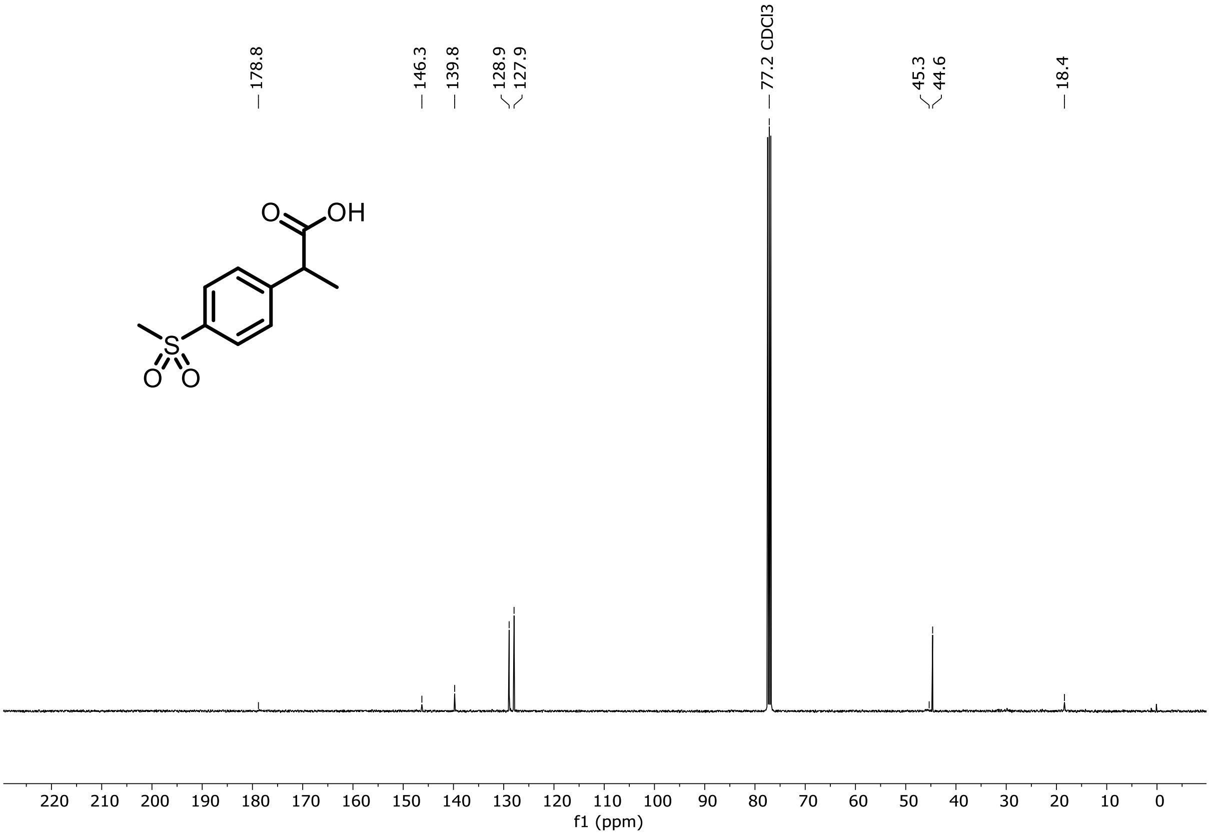

2-(4-acetylphenyl)propanoic acid (2d)

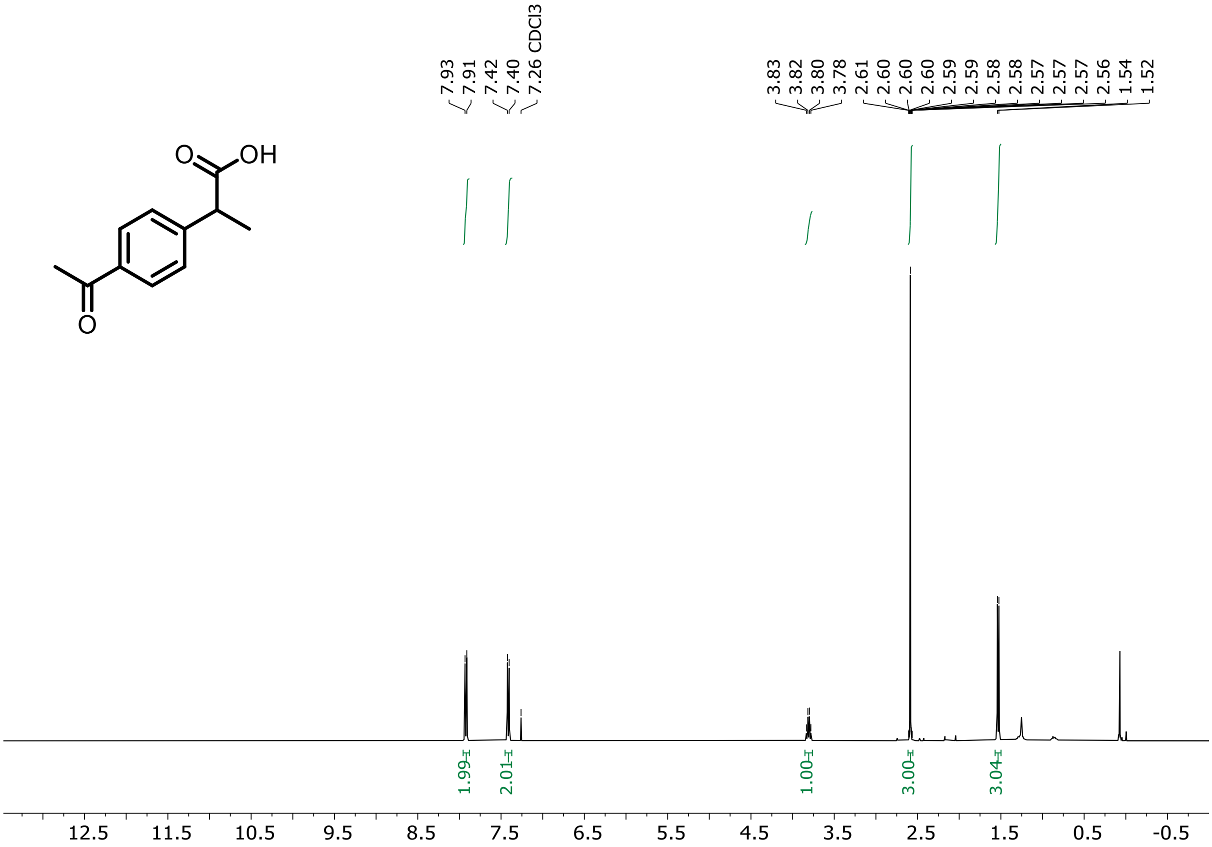

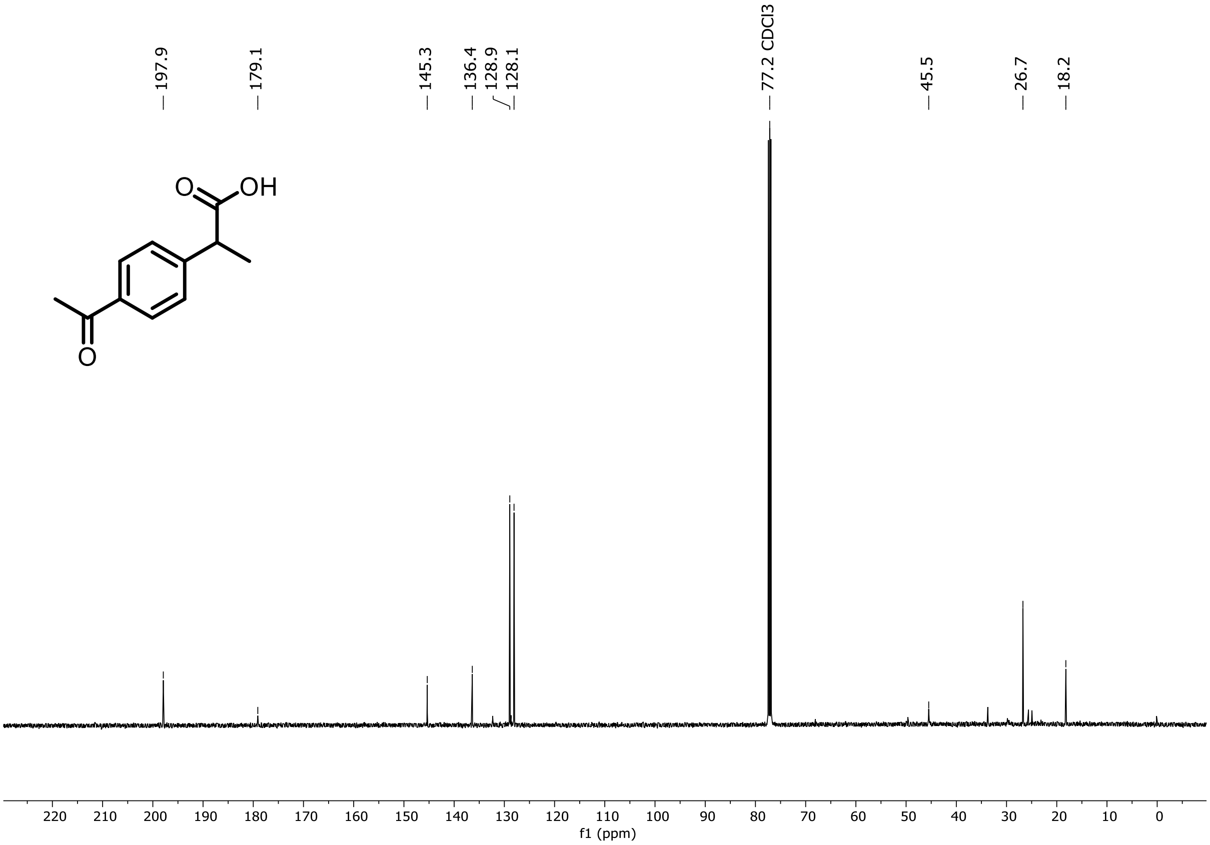

2-(4-(methoxycarbonyl)phenyl)propanoic acid (2e)

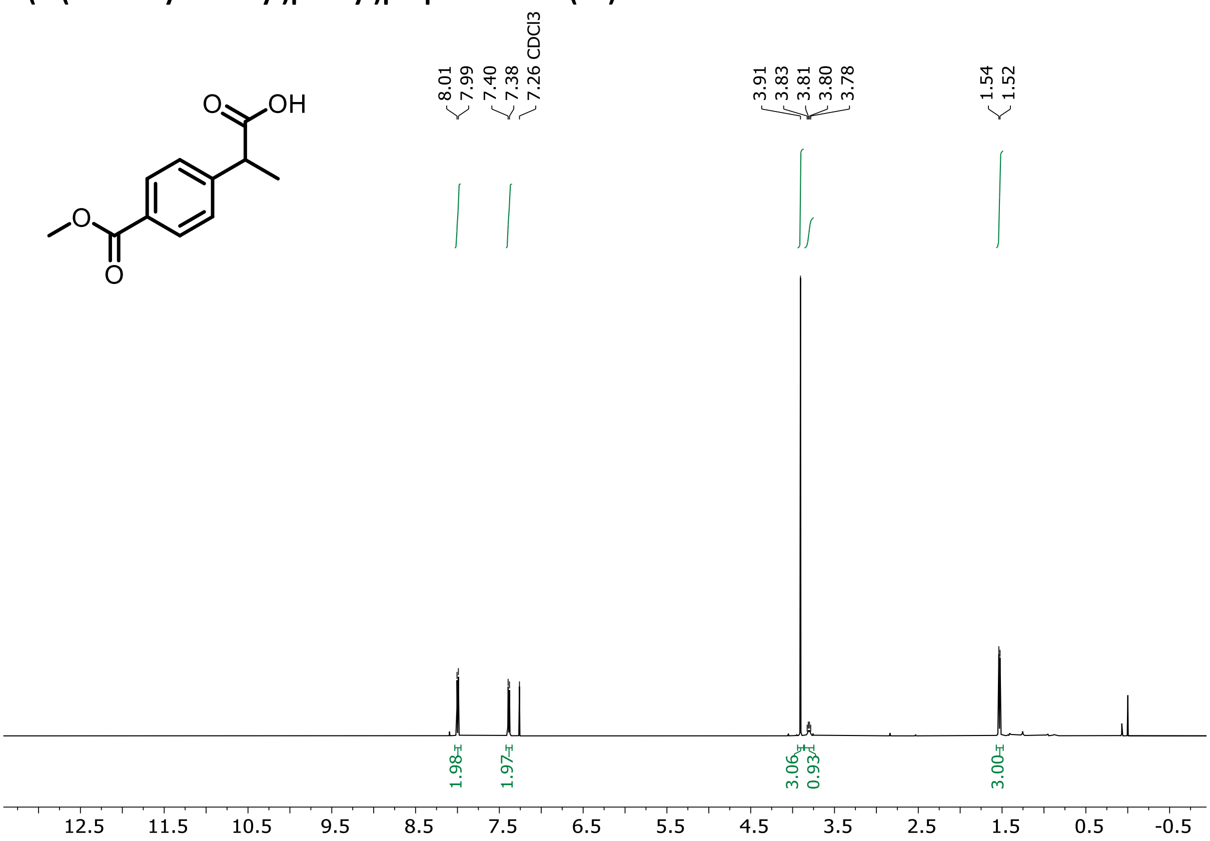

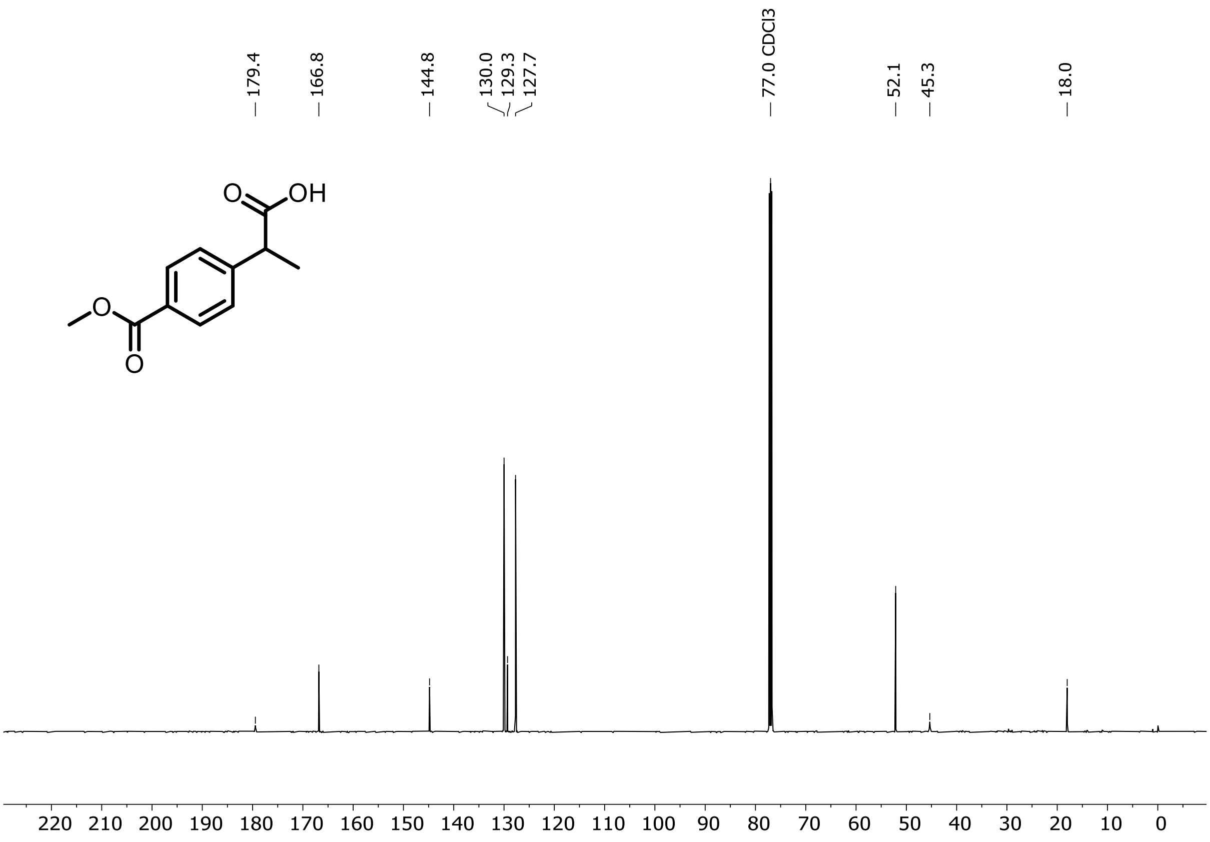

2-(4-(tert-butoxycarbonyl)phenyl)propanoic acid (2f)

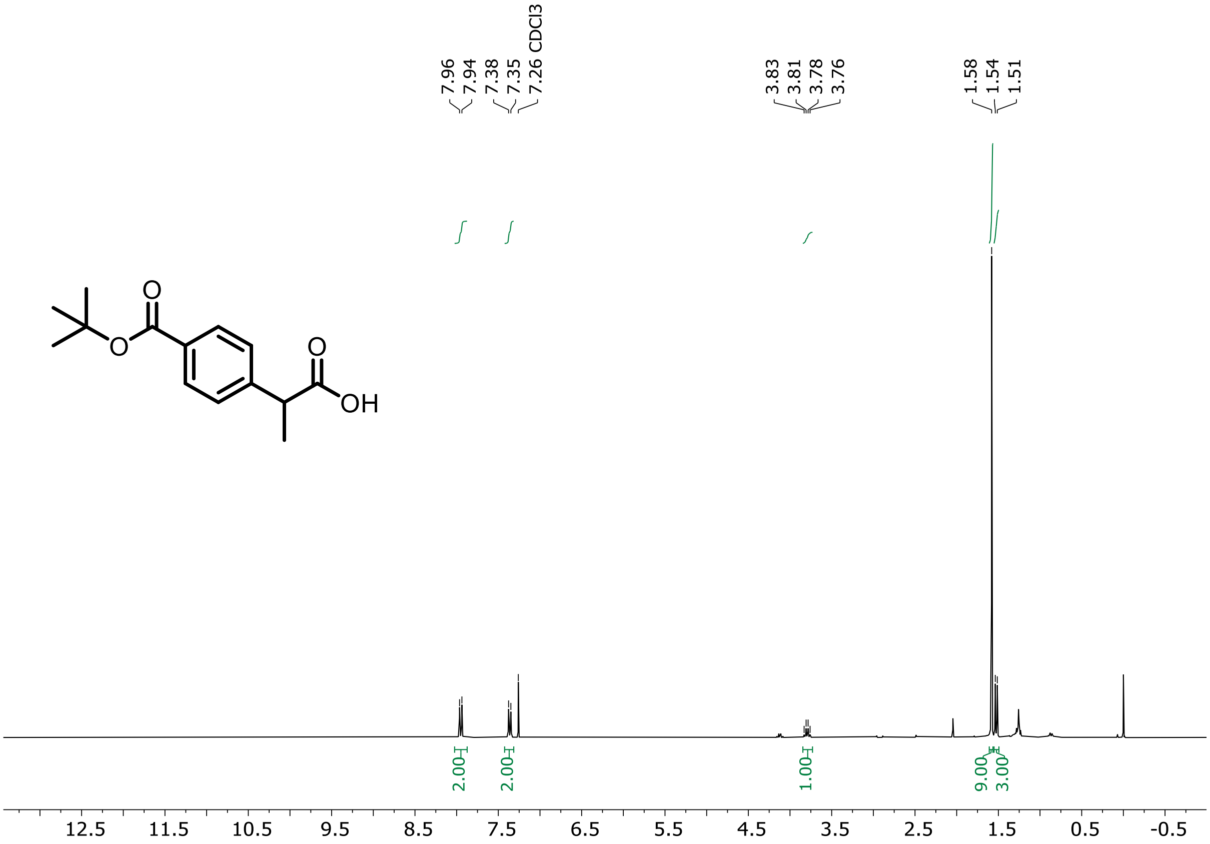

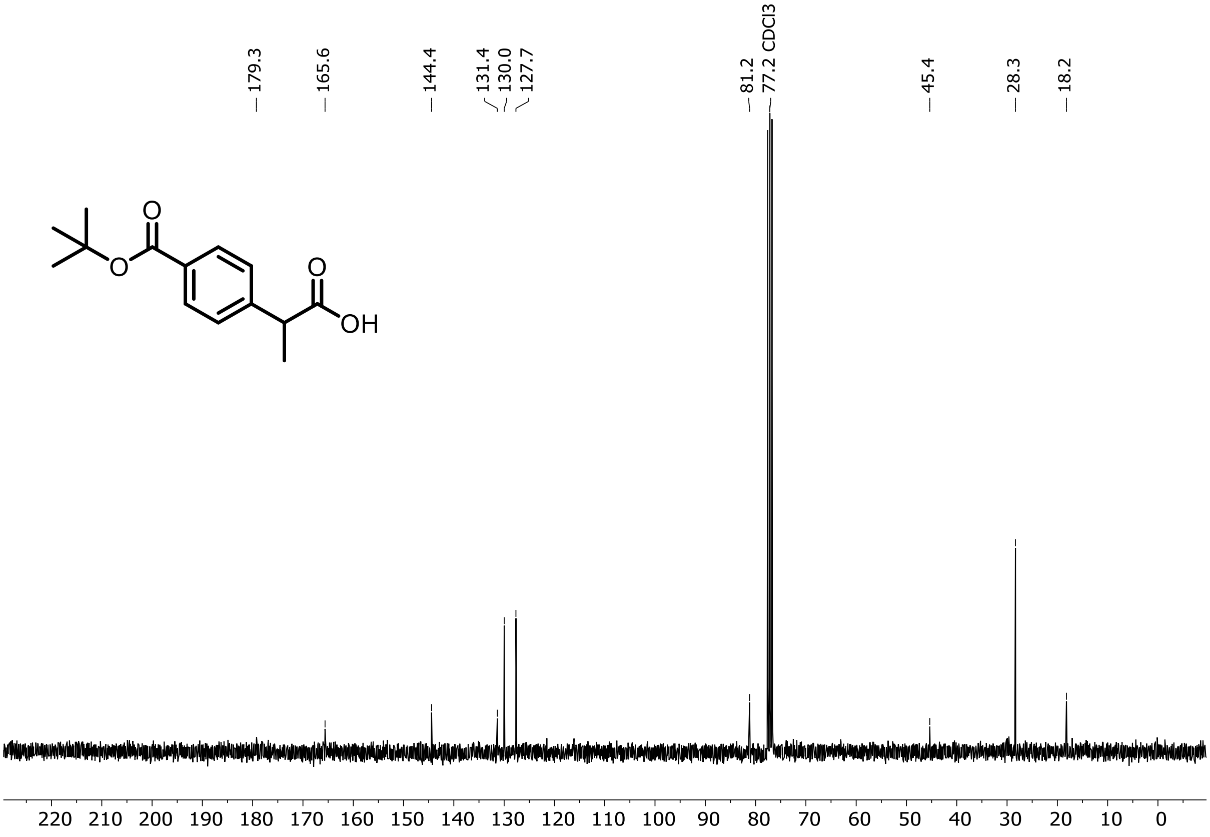

2-([1,1'-biphenyl]-4-yl)propanoic acid (2g)

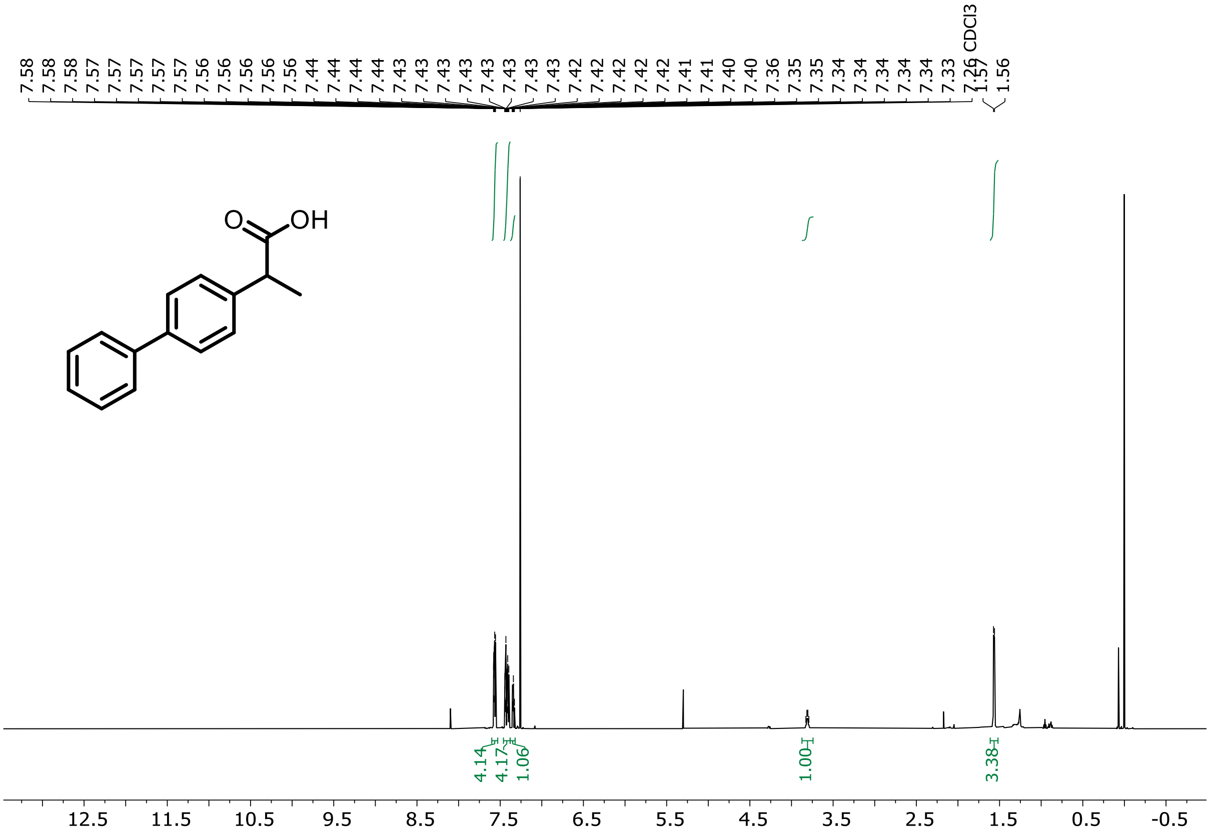

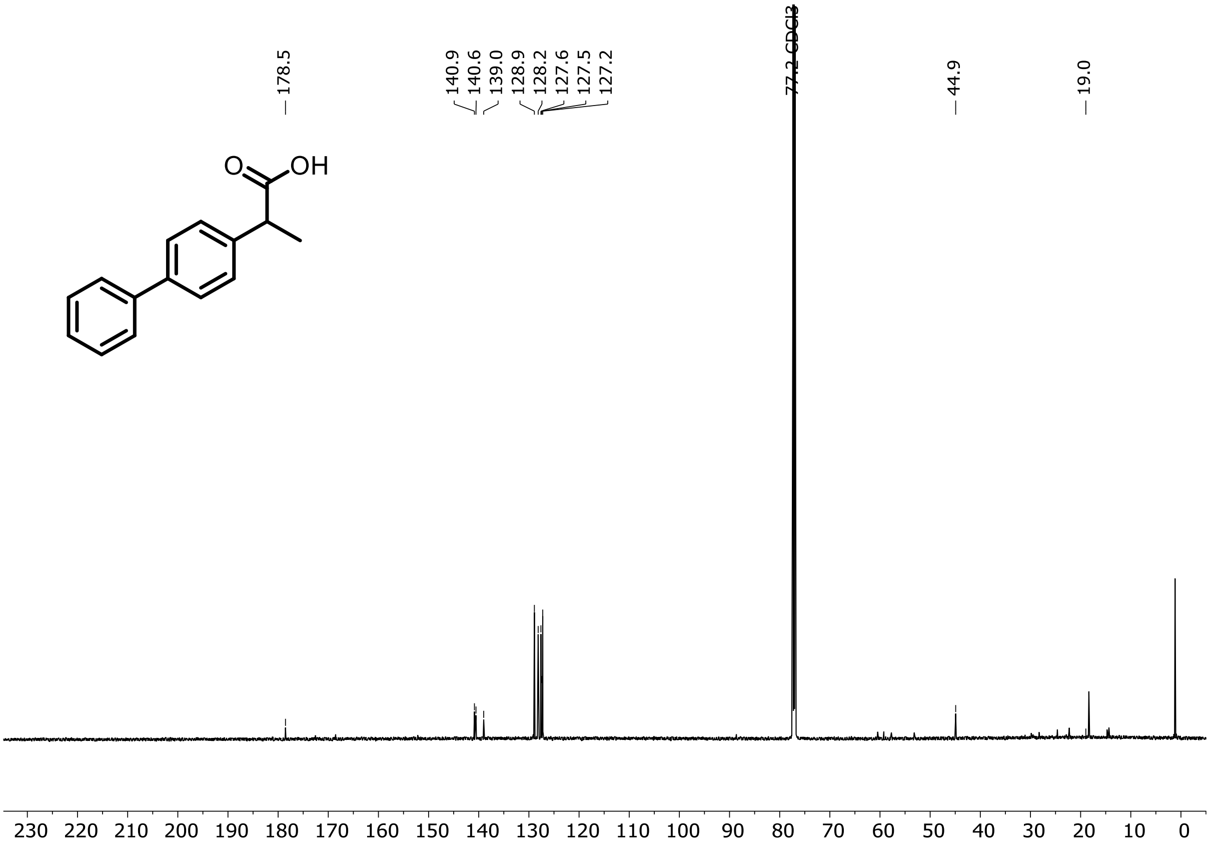

2-methyl-2-(naphthalen-2-yl)propanoic acid (2h)

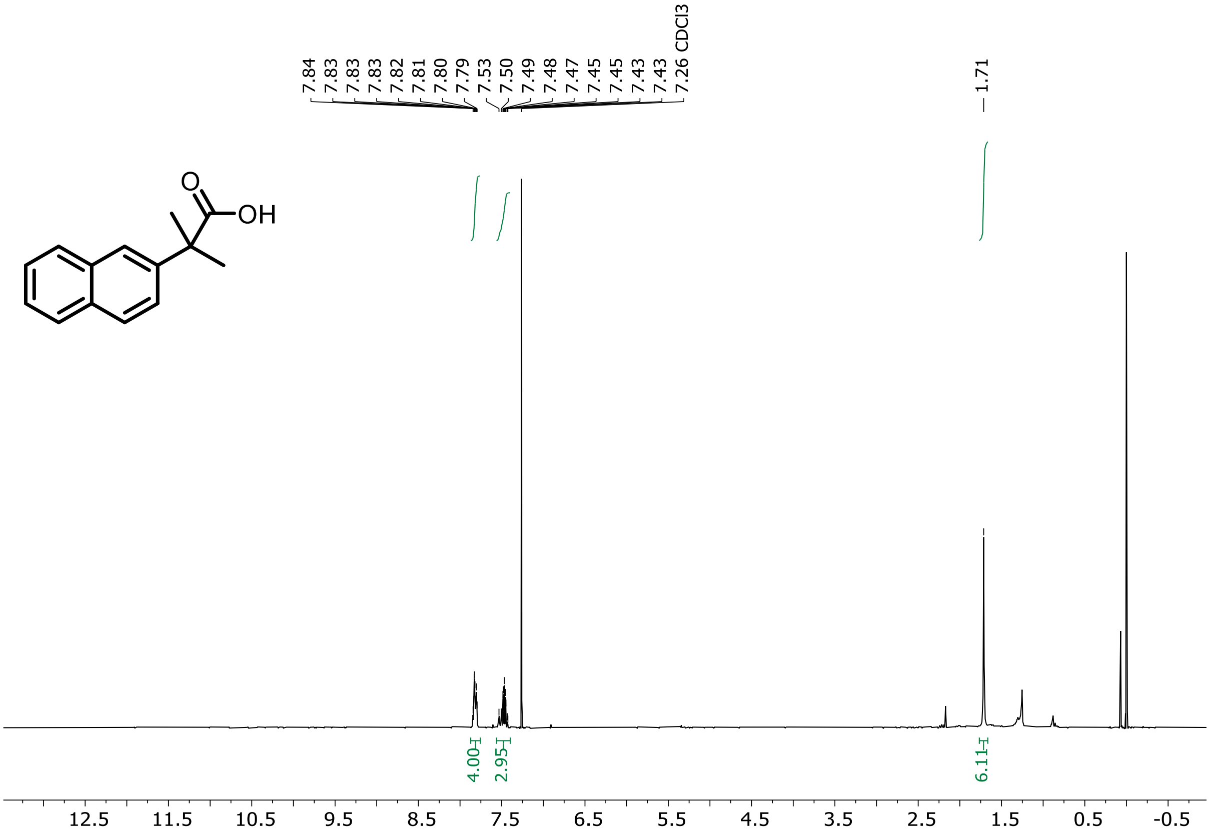

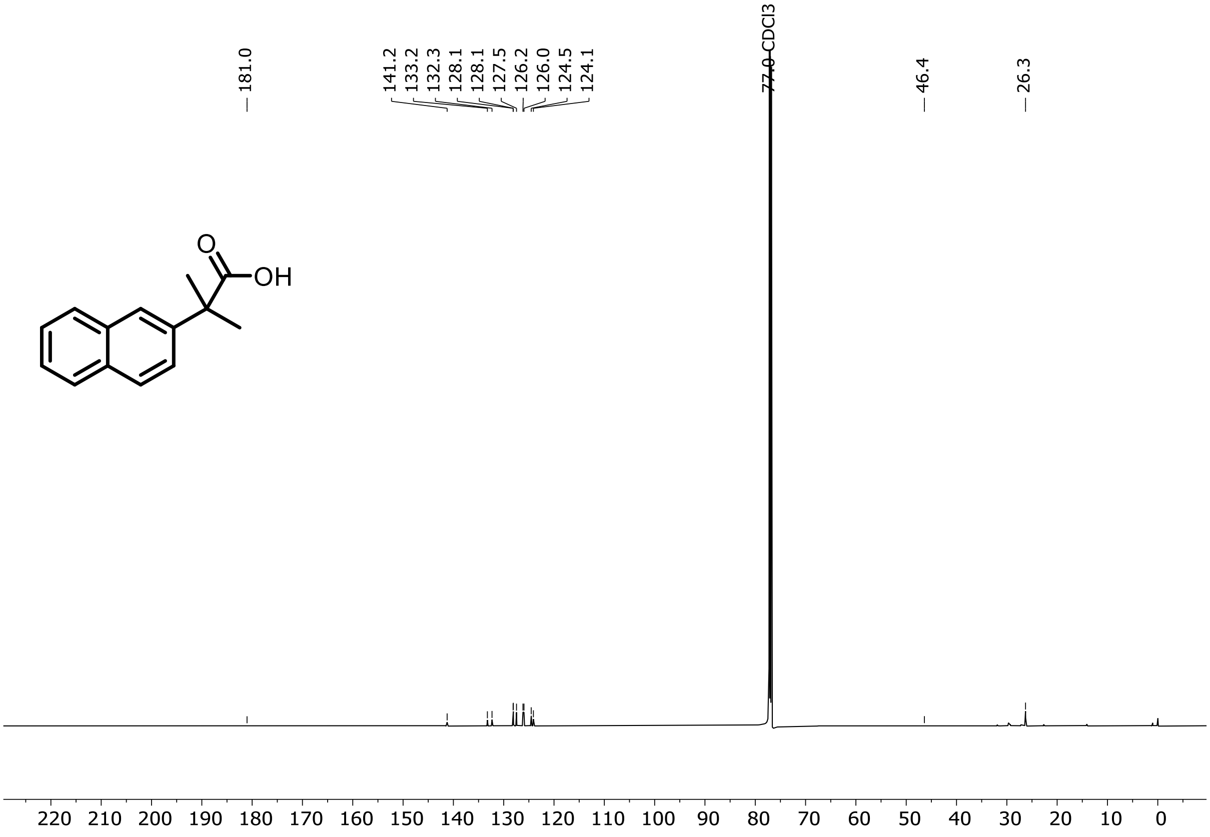

2-(4-cyanophenyl)butanoic acid (2i)

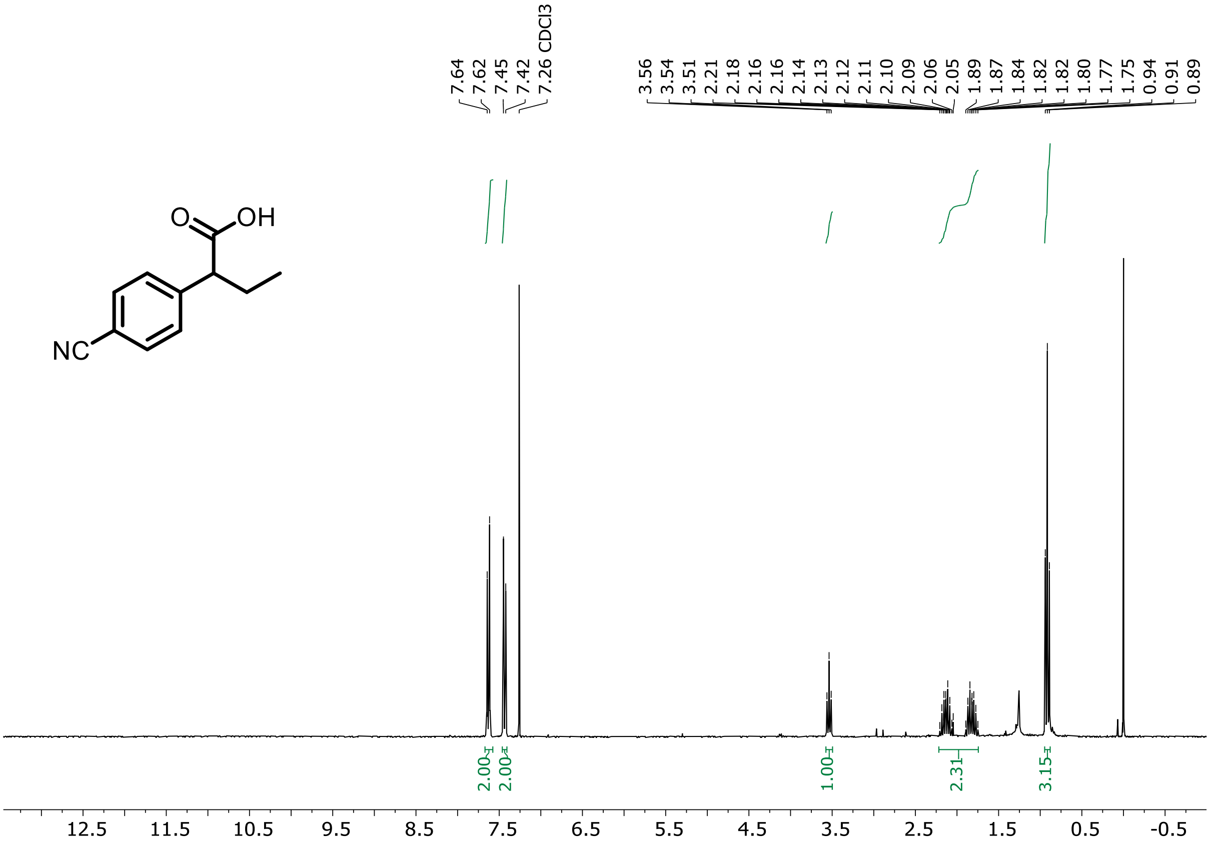

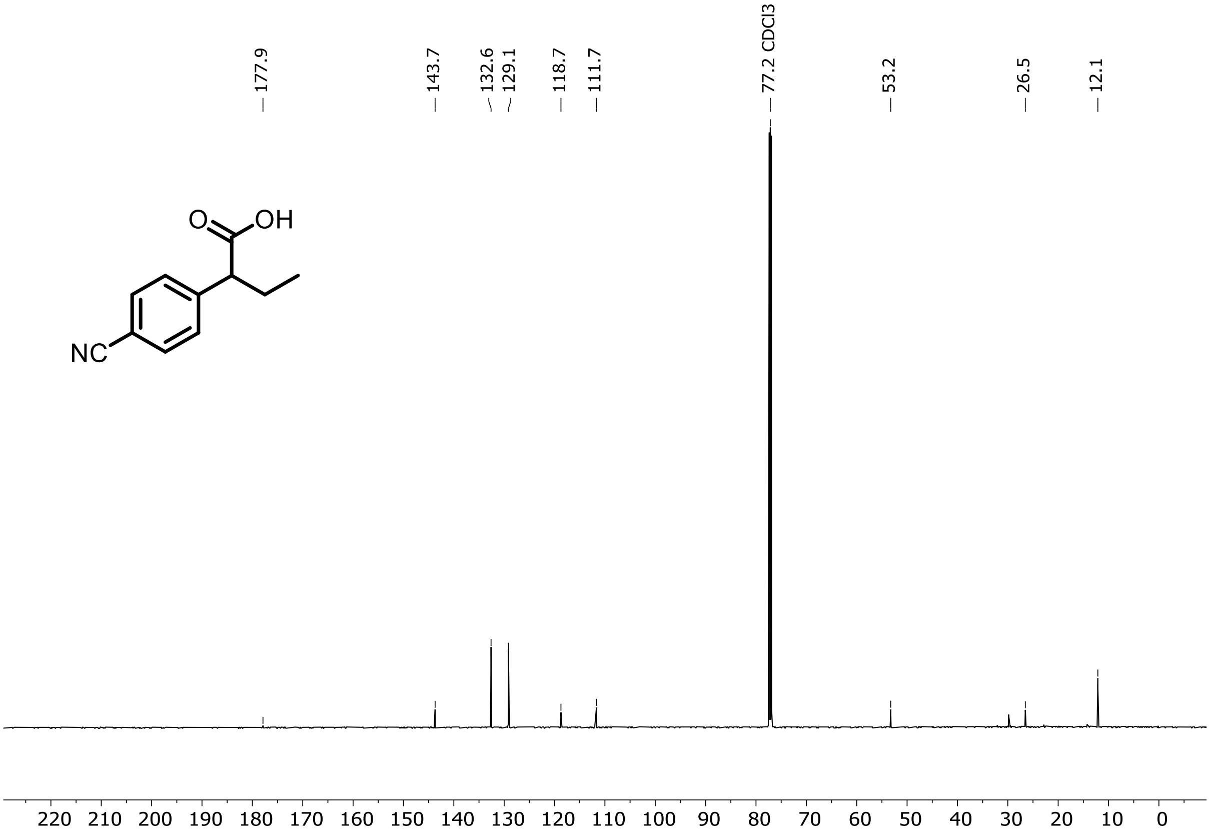

2-(2-cyanophenyl)propanoic acid (2j)

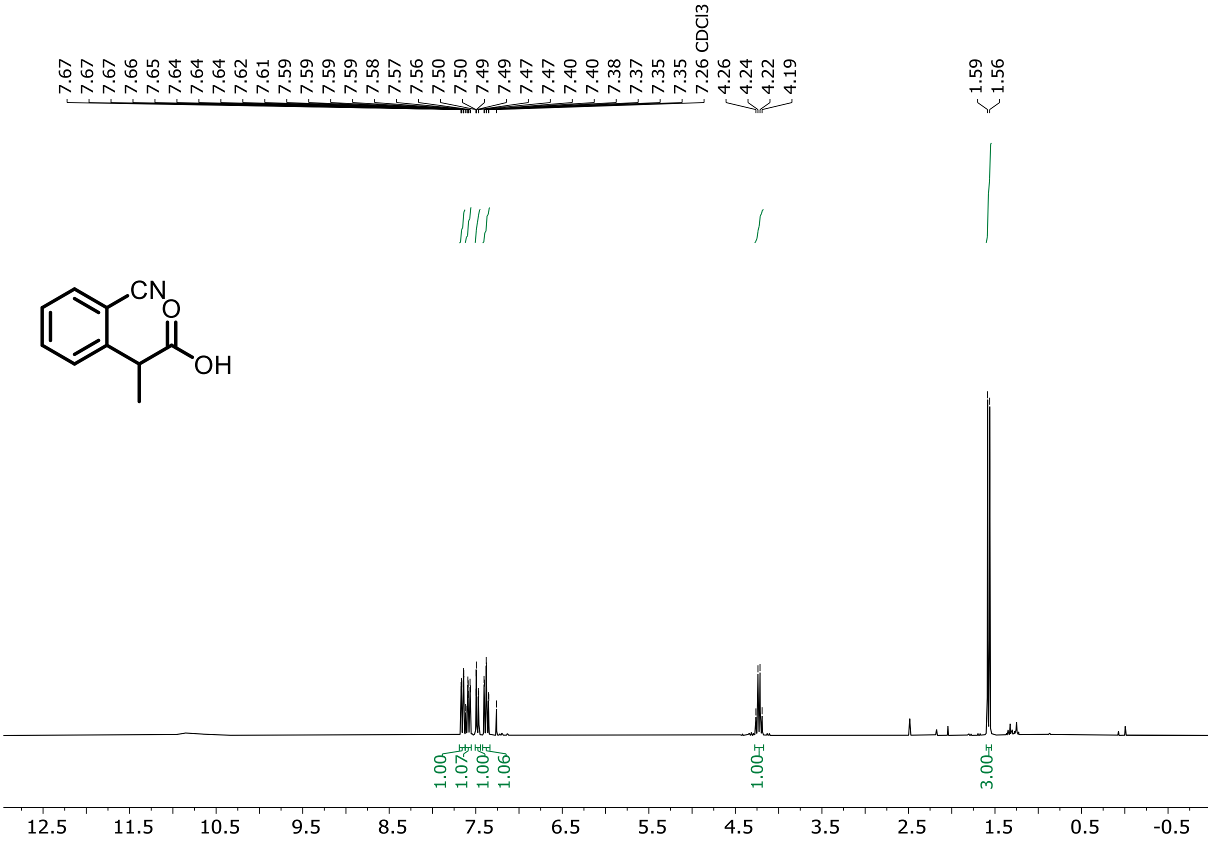

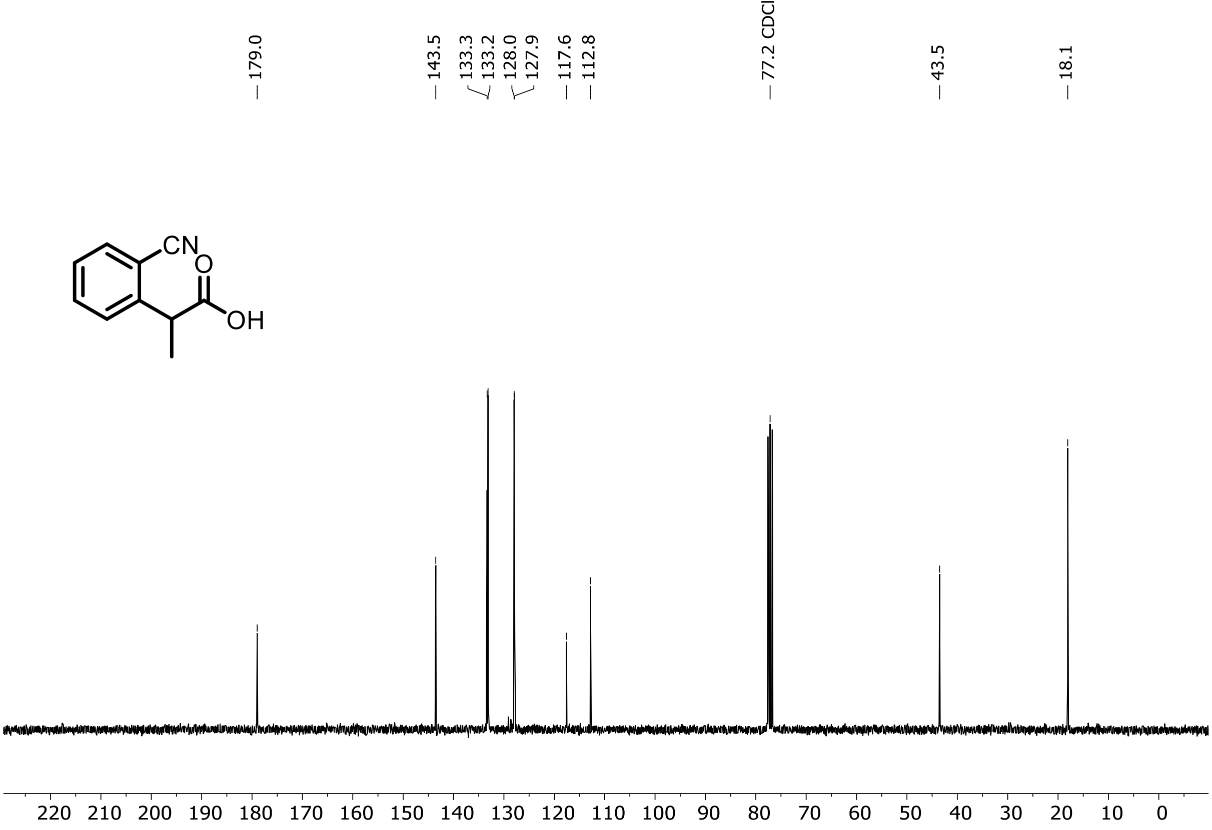

2-(2-chlorophenyl)propanoic acid (2k)

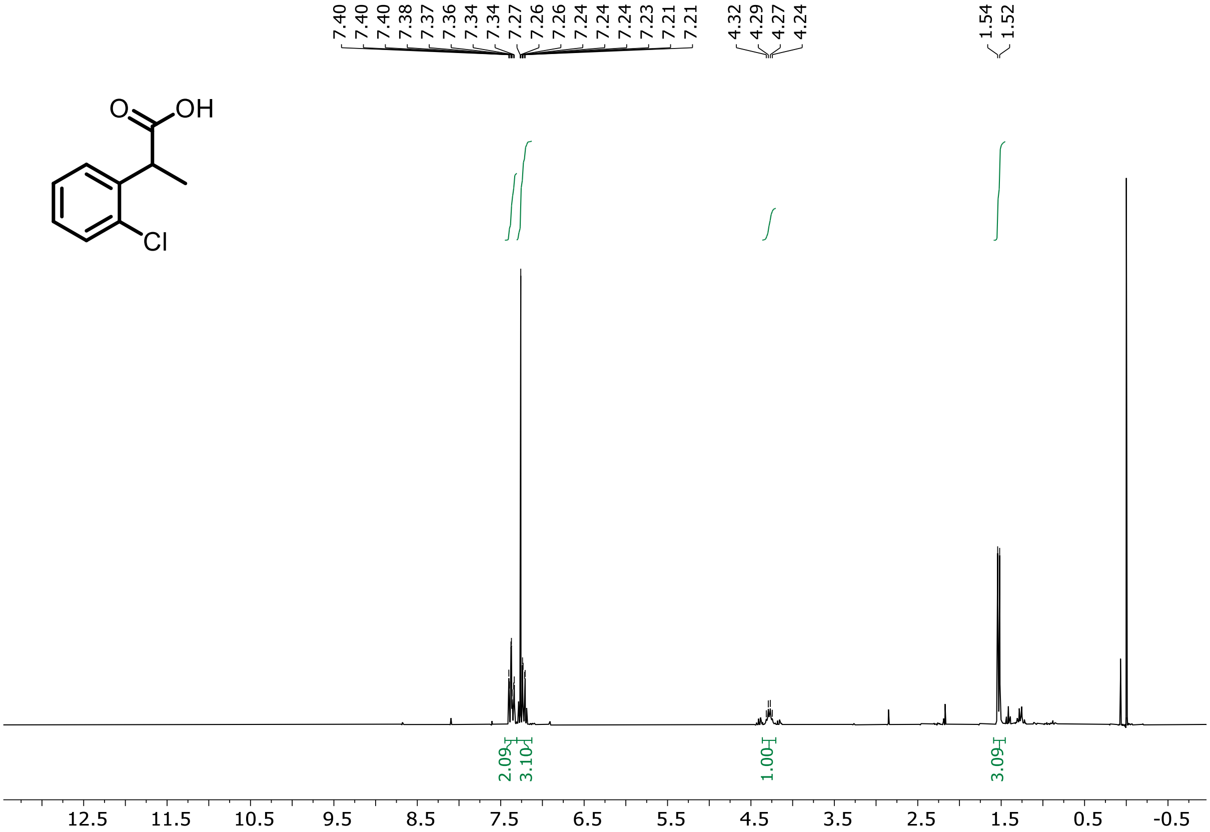

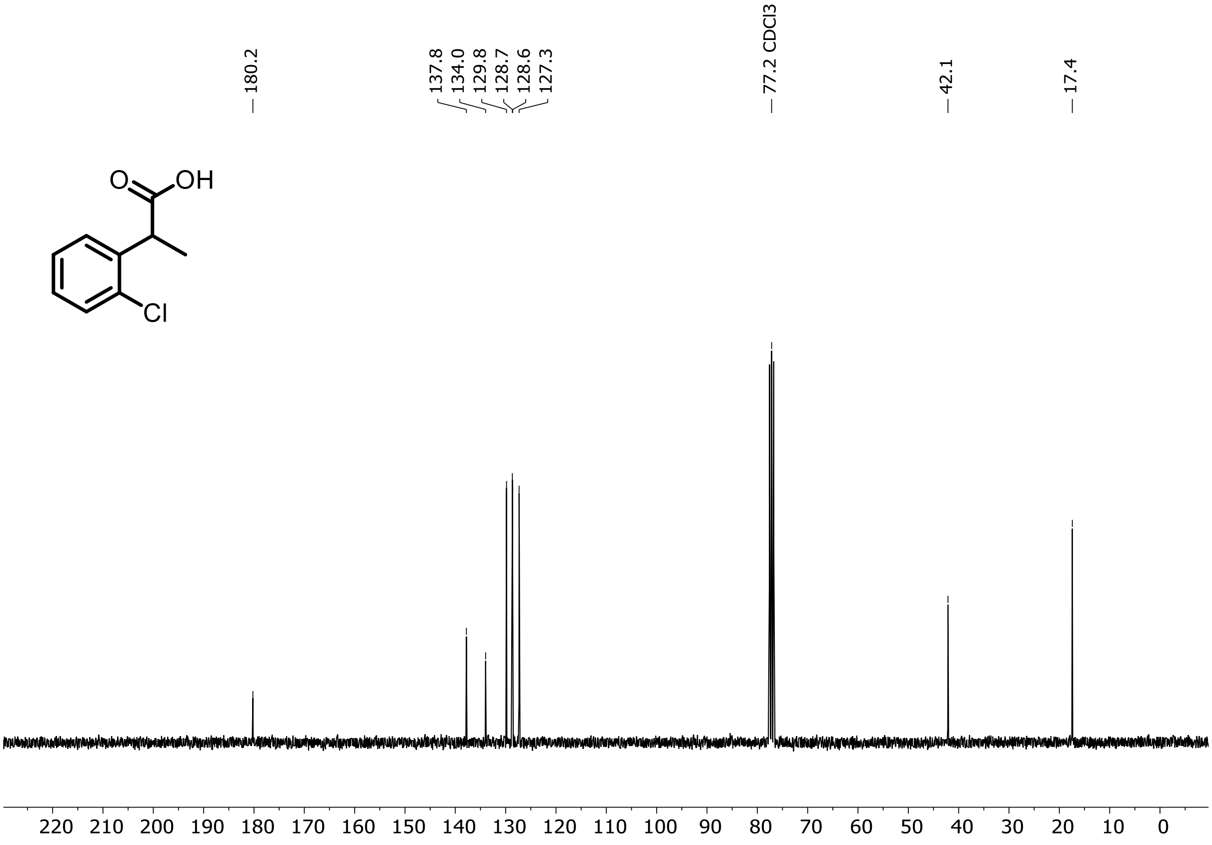

2-(3-chlorophenyl)propanoic acid (2l)

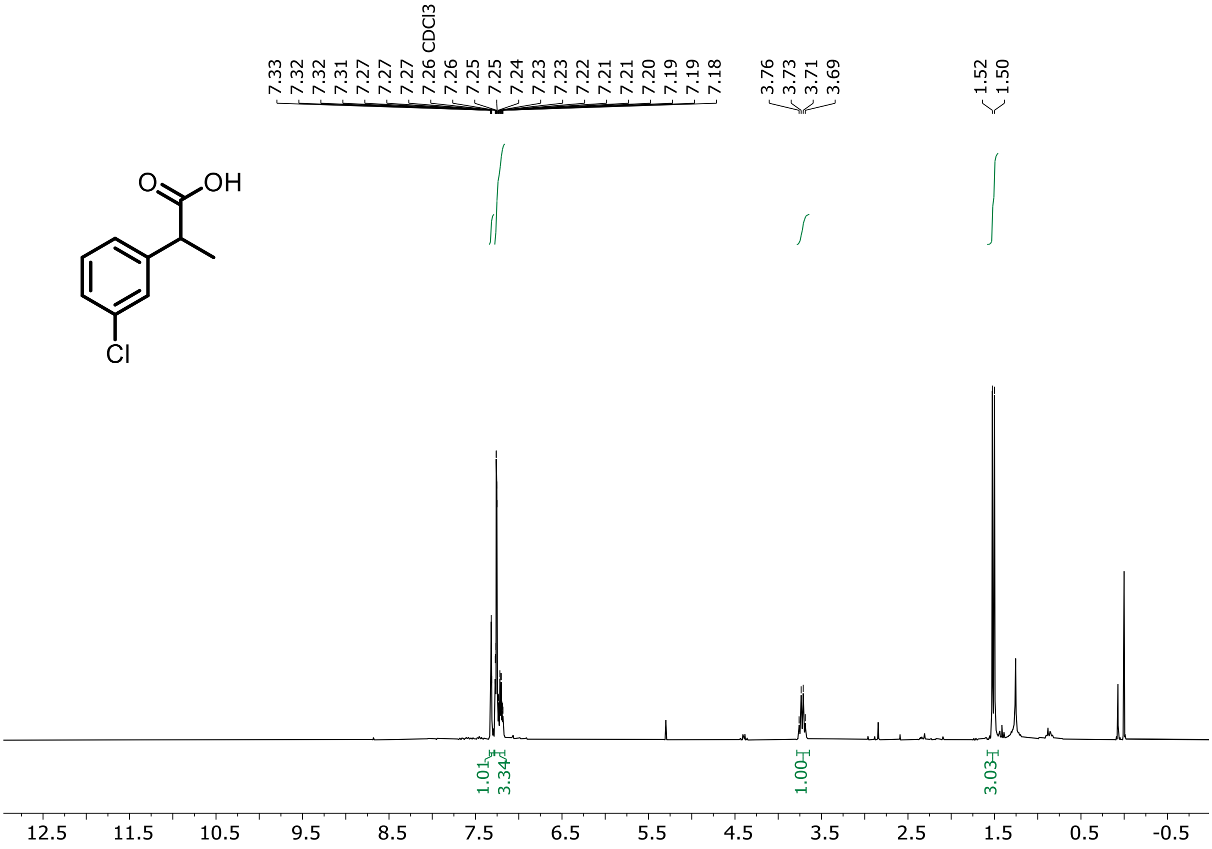

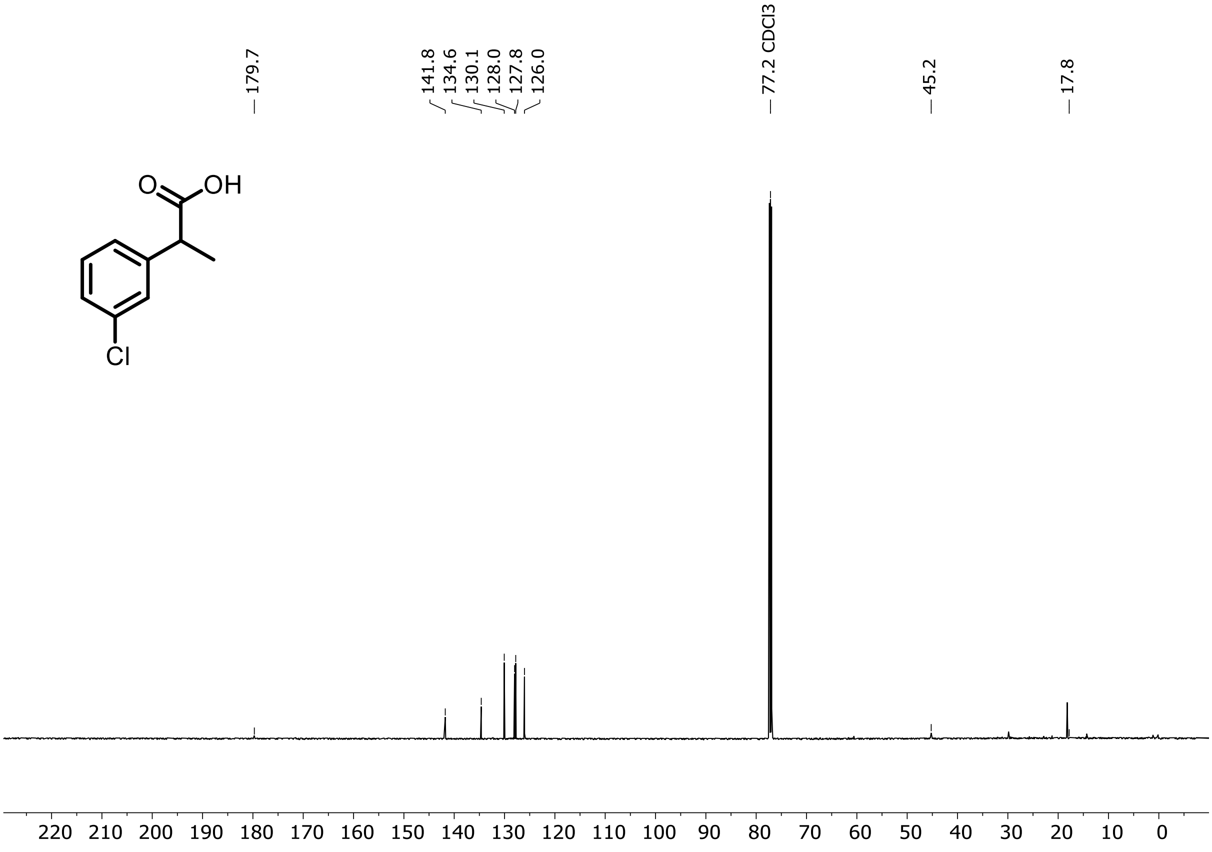

2-(2-bromophenyl)propanoic acid (2m)

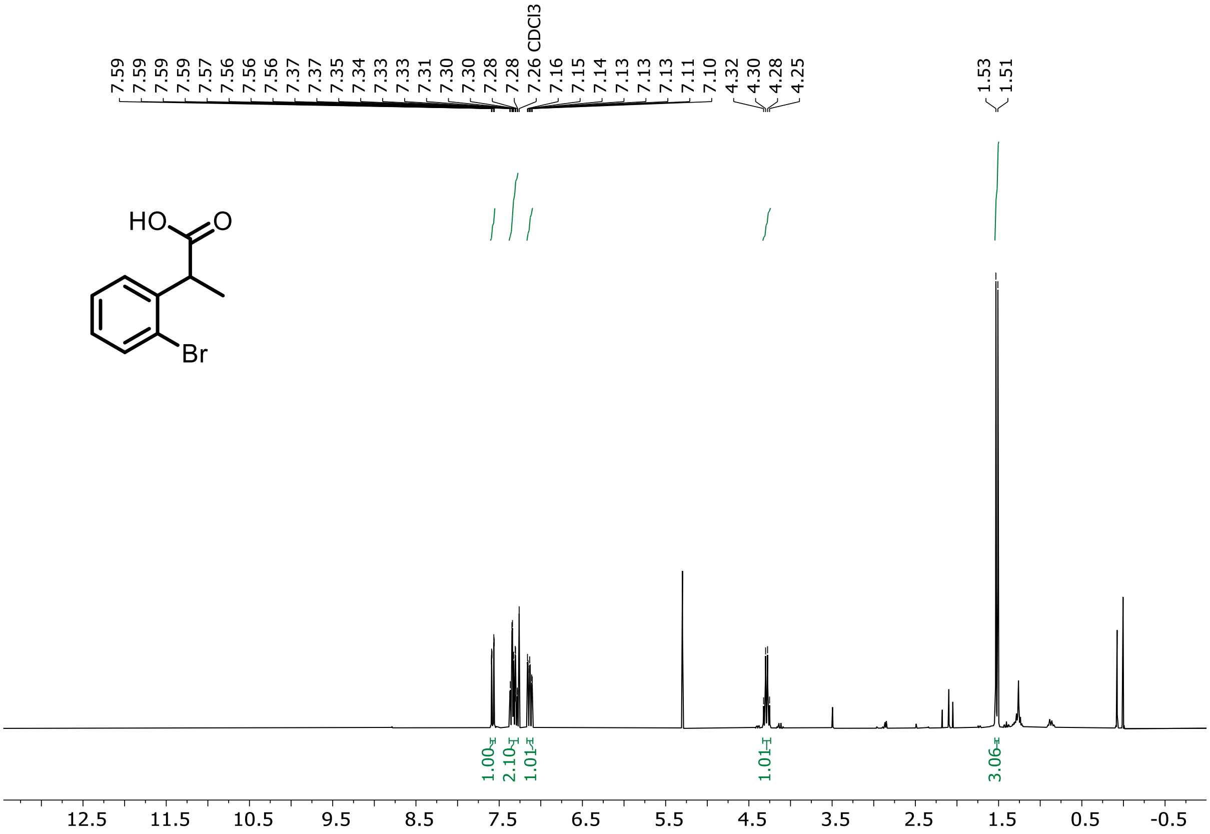

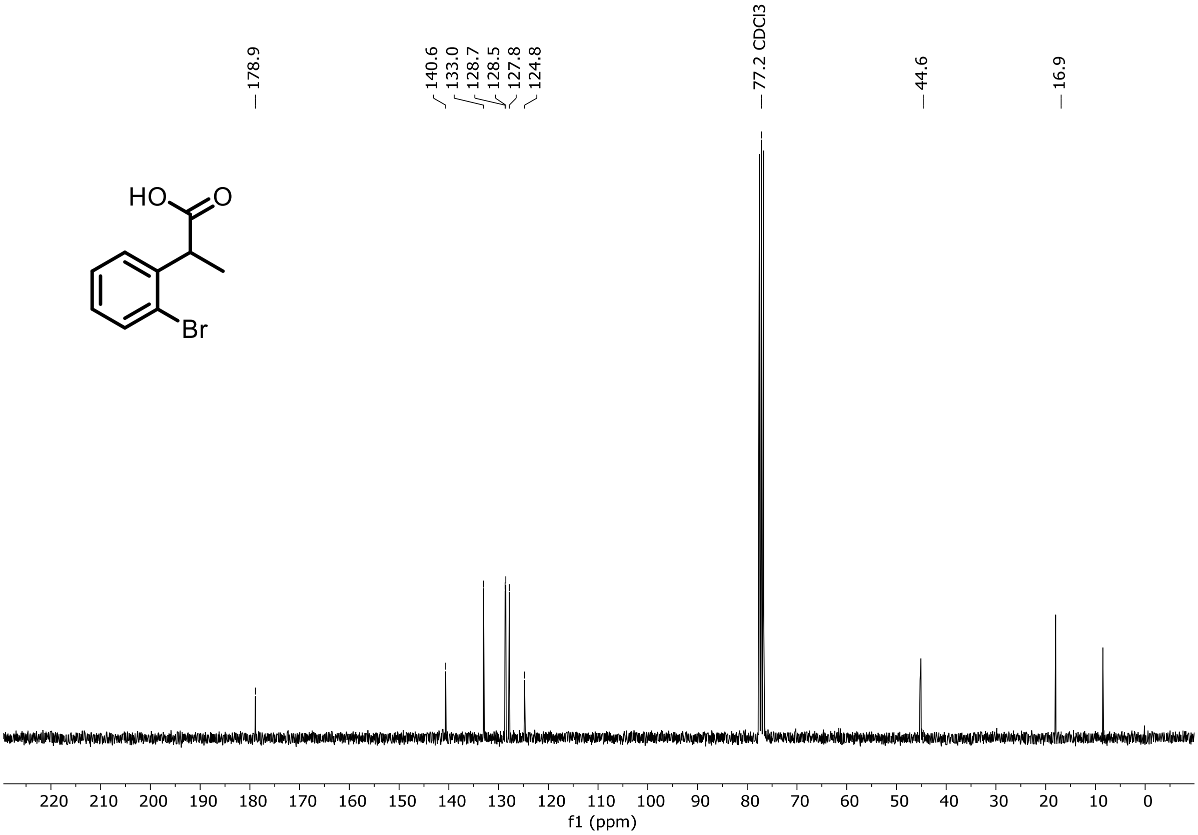

2-(perfluorophenyl)propanoic acid (2n)

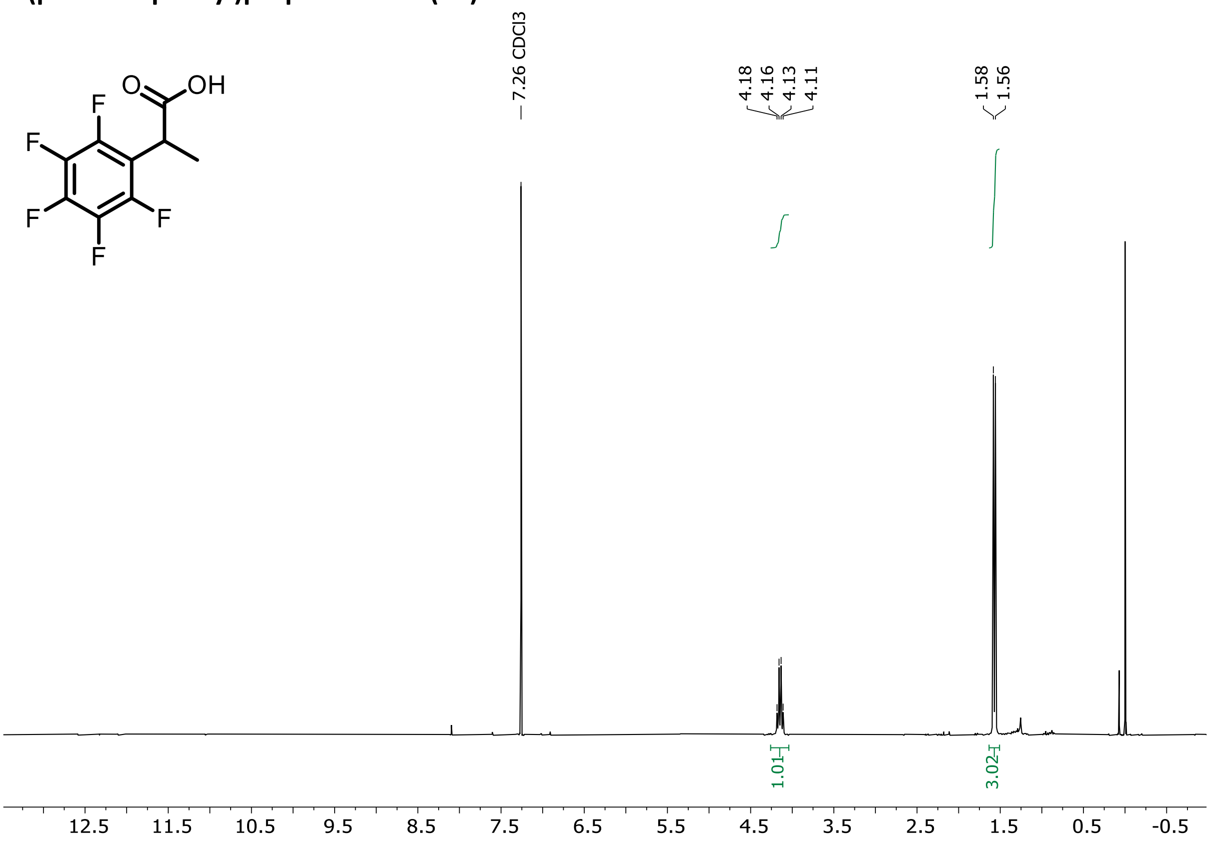

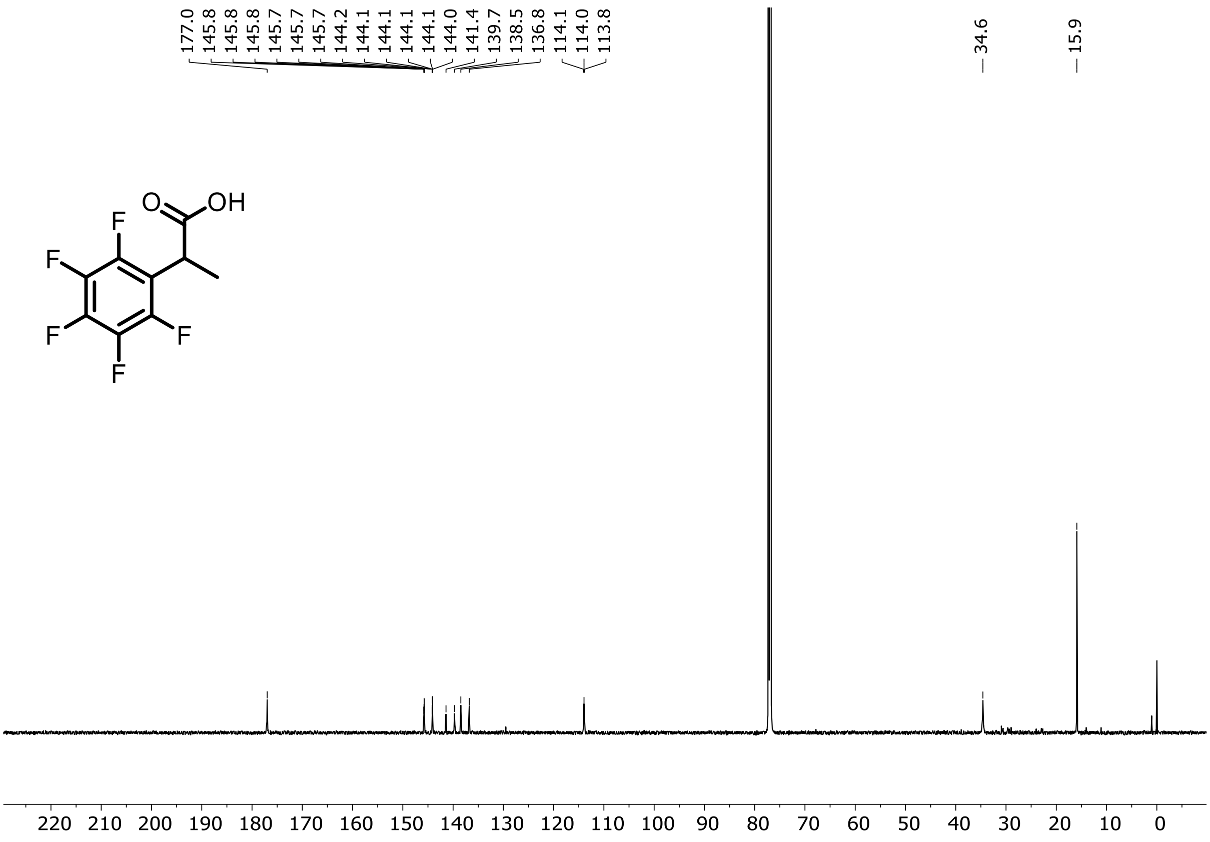

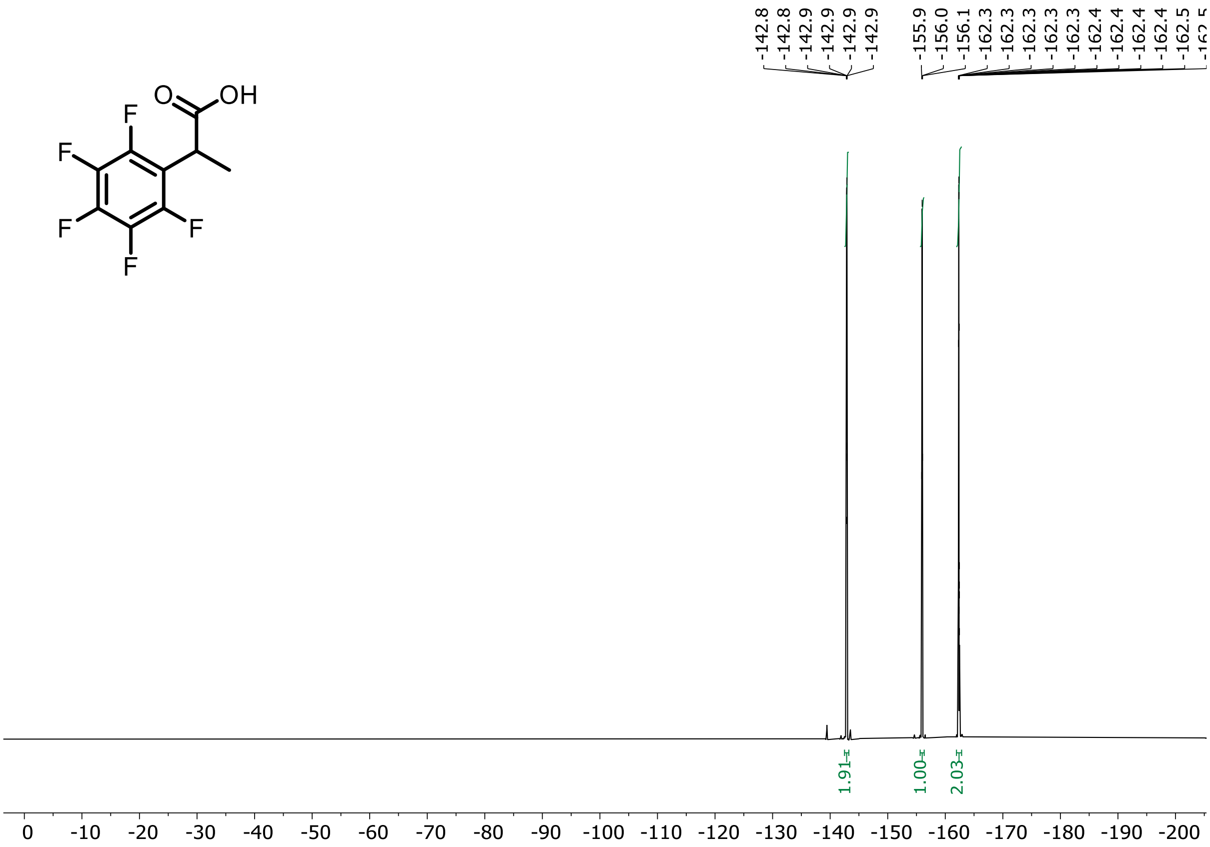

_N_-benzyl-2-(perfluorophenyl)propenamide (2o)

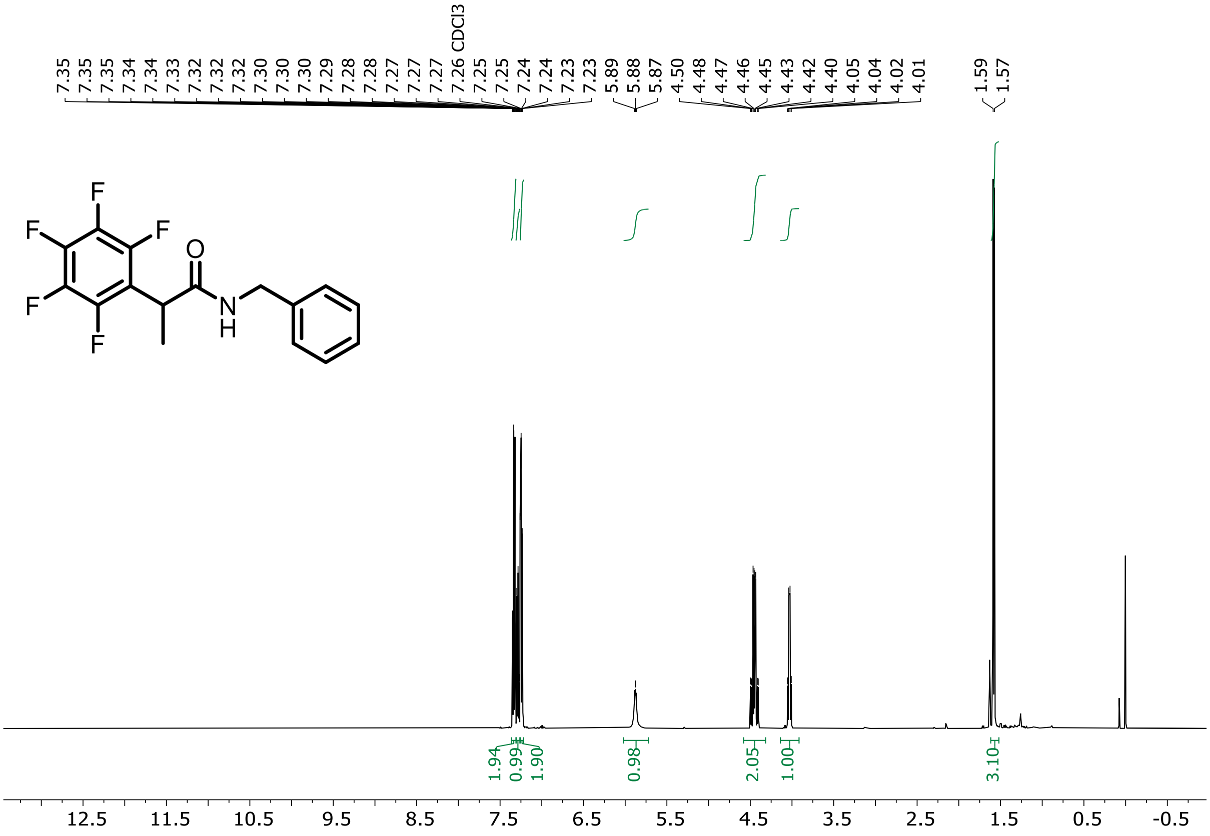

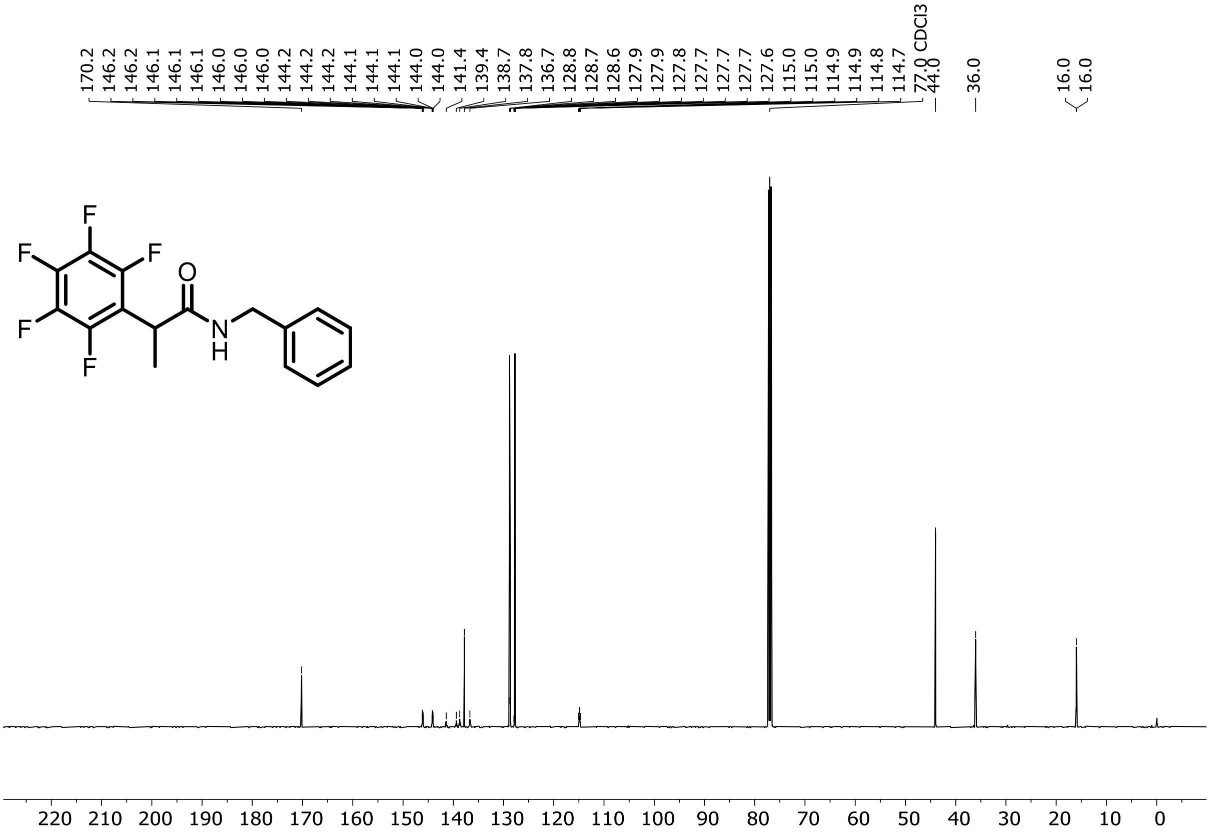
170.2
146.2
146.2
146.1
146.1
146.1
146.0
146.0
144.2
144.2
144.2
144.1
144.1
144.1
144.0
144.0
141.4
139.4
138.7
137.8
136.7
128.8
128.7
128.6
127.9
127.9
127.8
127.7
127.7
127.7
127.6
115.0
115.0
114.9
114.9
114.8
114.7
77.0 CDCl3
44.0
36.0
16.0
16.0
F
F
F
F
F
O
N
H
220 210 200 190 180 170 160 150 140 130 120 110 100 90 80 70 60 50 40 30 20 10 0

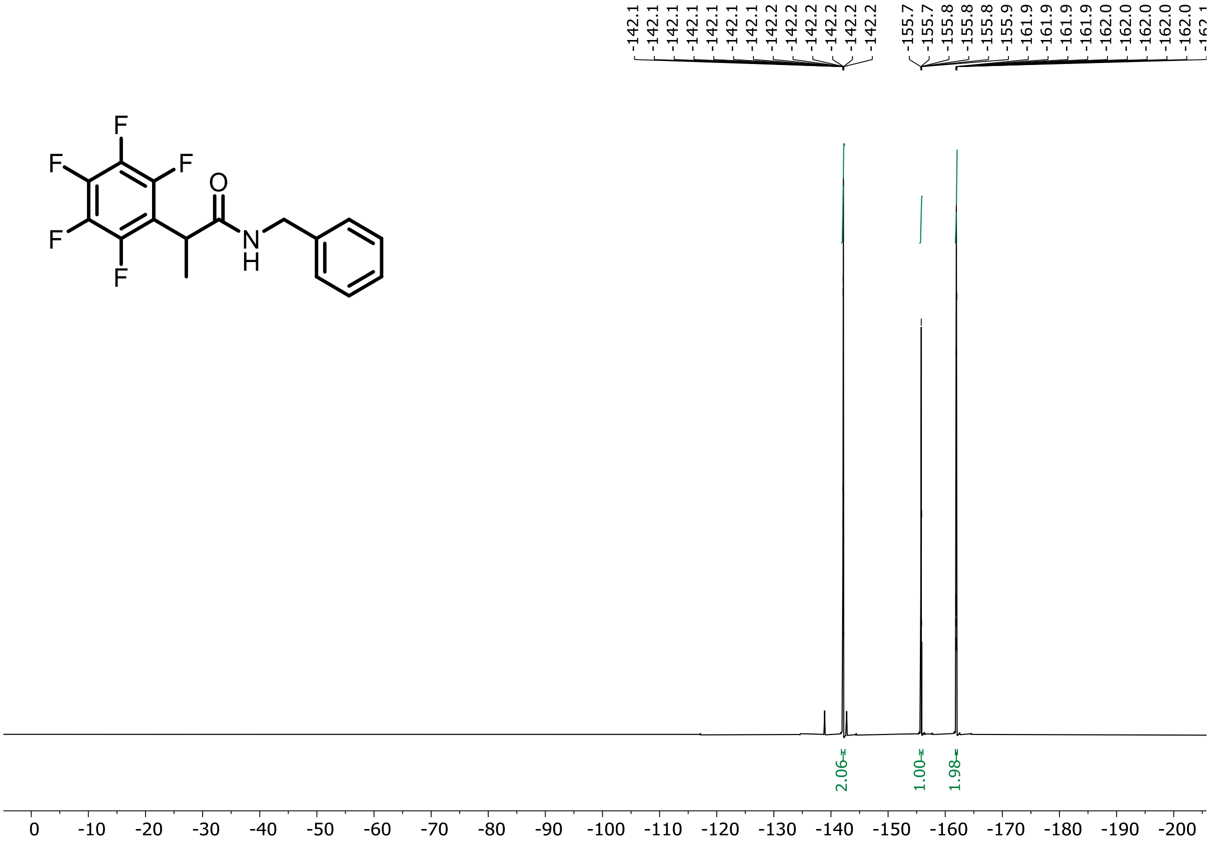
-142.1
-142.1
-142.1
-142.1
-142.1
-142.1
-142.2
-142.2
-142.2
-142.2
-142.2
-142.2
-155.7
-155.7
-155.8
-155.8
-155.8
-155.9
-161.9
-161.9
-161.9
-161.9
-162.0
-162.0
-162.0
-162.0
F
F
F
F
F
O
N
H
2.06
1.00
1.98
0 -10 -20 -30 -40 -50 -60 -70 -80 -90 -100 -110 -120 -130 -140 -150 -160 -170 -180 -190 -200

2,2-diphenylpropanoic acid (2p)

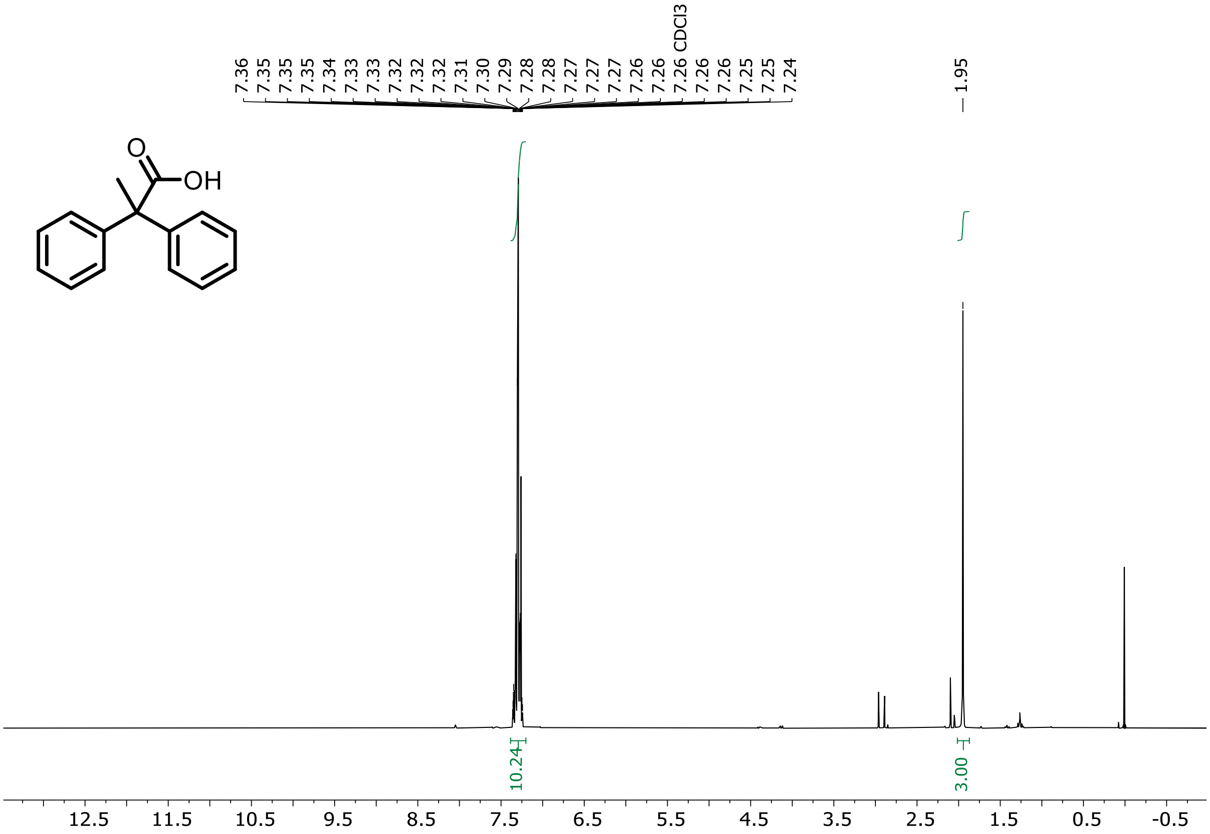

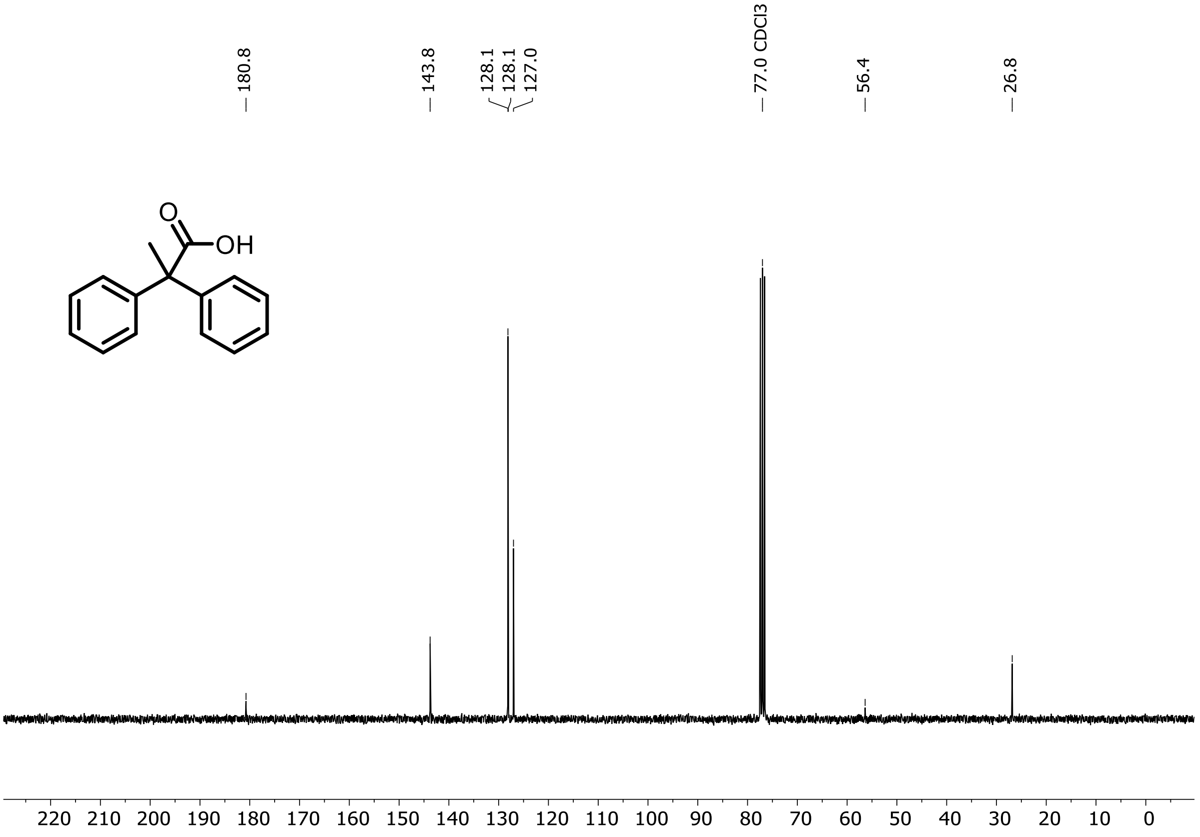

2-(4-methoxyphenyl)-2-phenylpropanoic acid (2q)

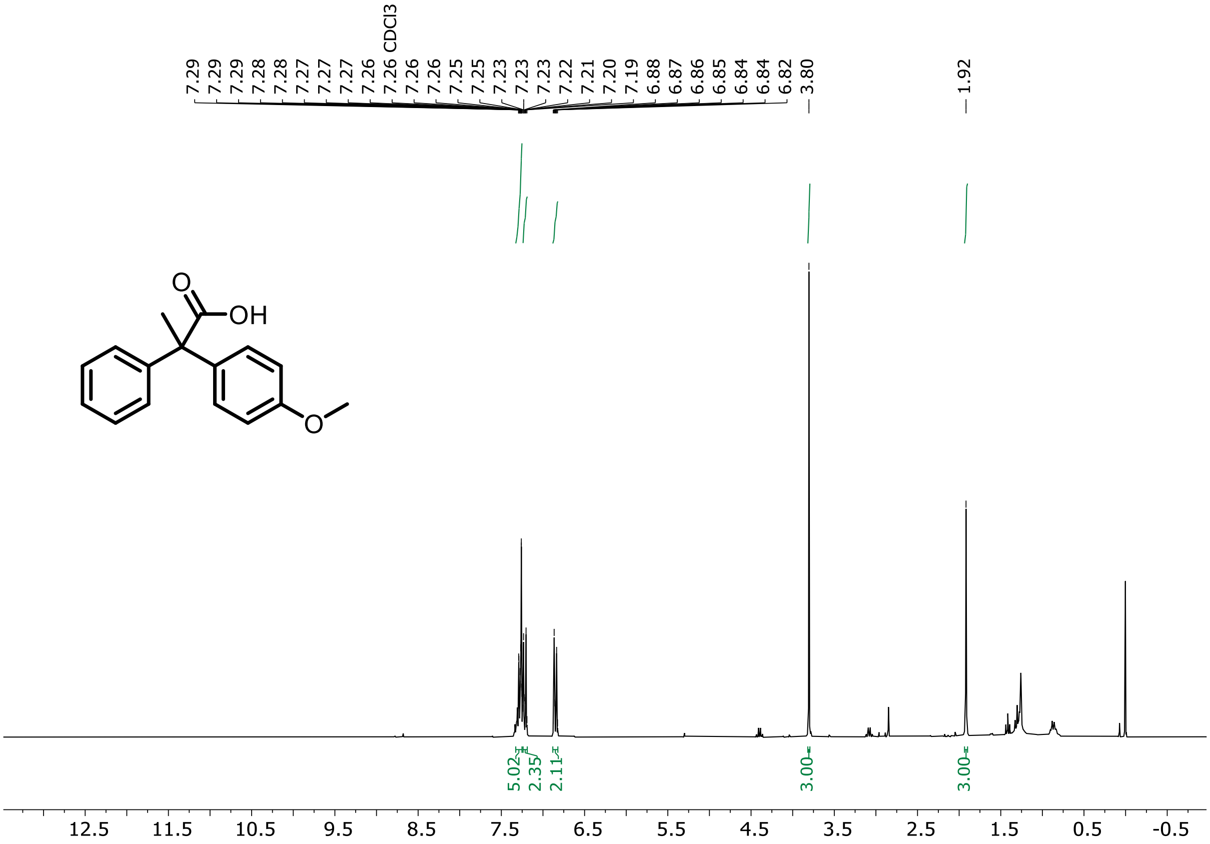

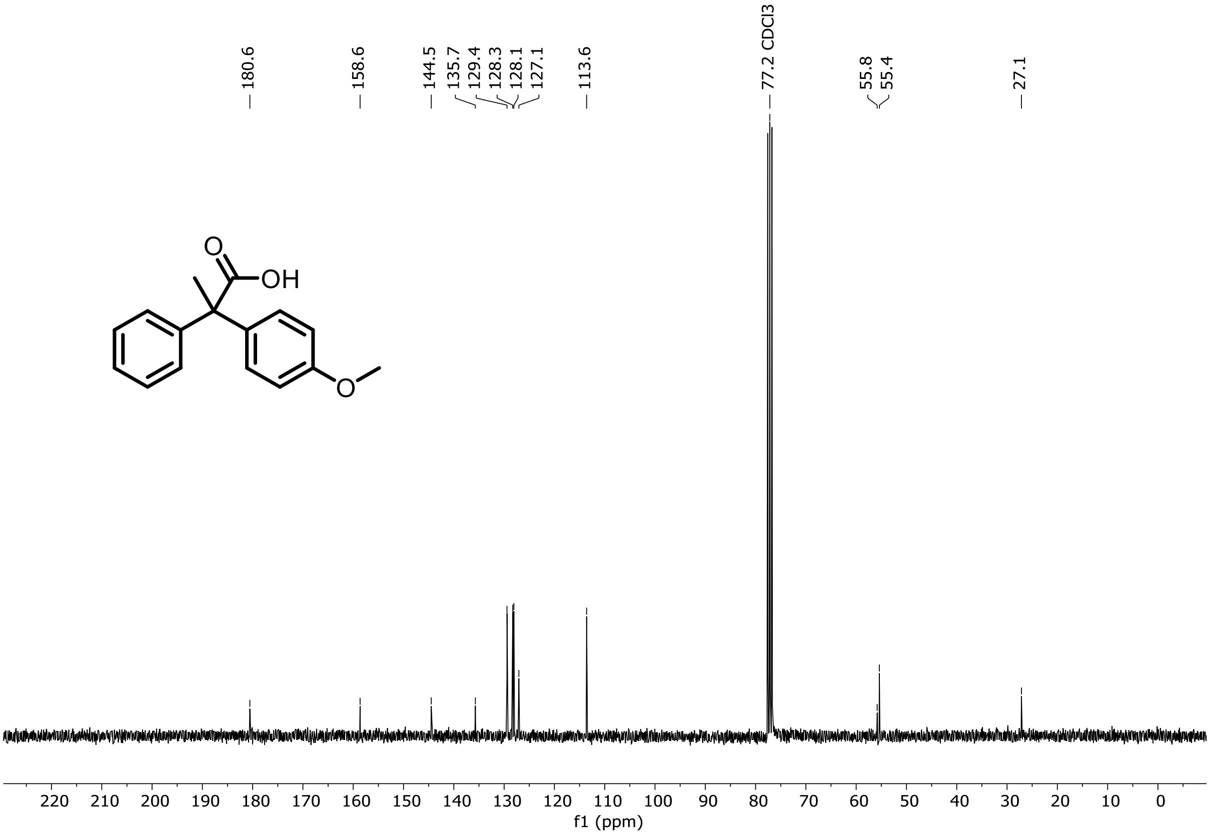

2-(4-fluorophenyl)-2-phenylpropanoic acid (2r)

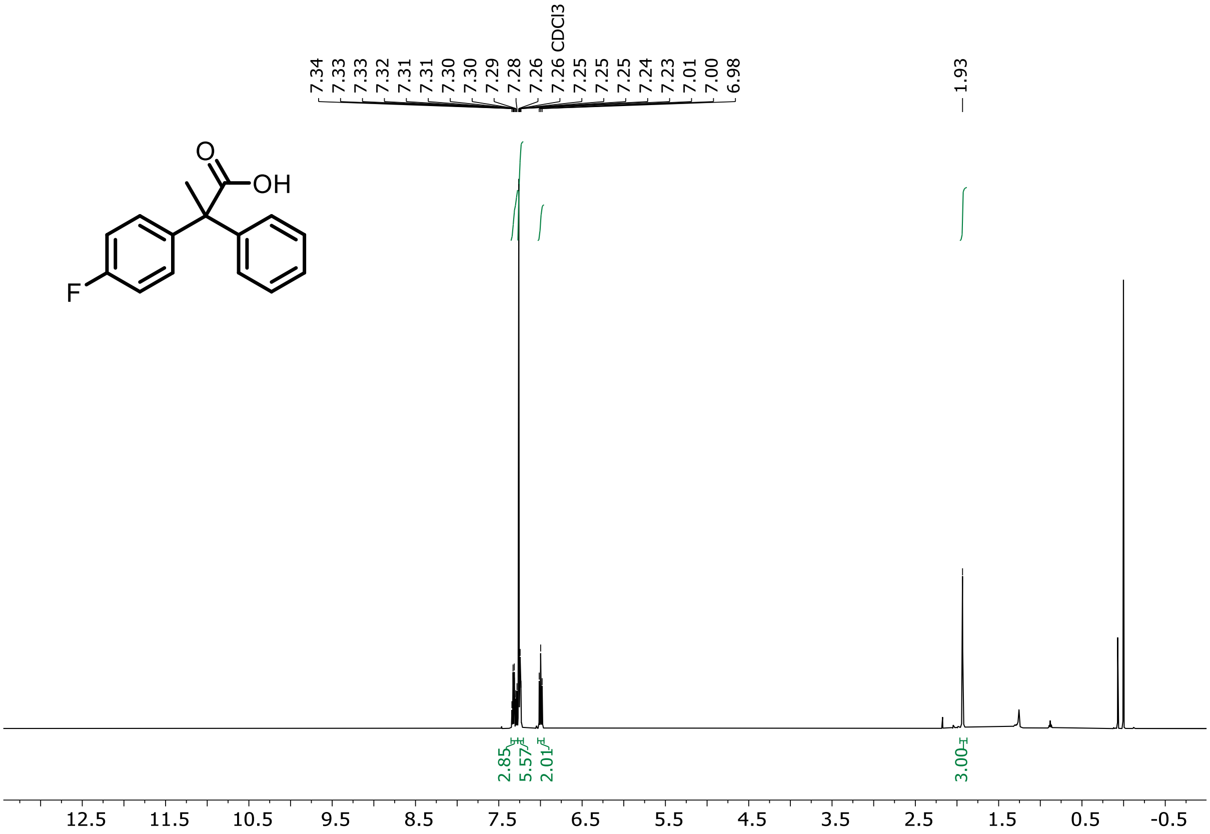

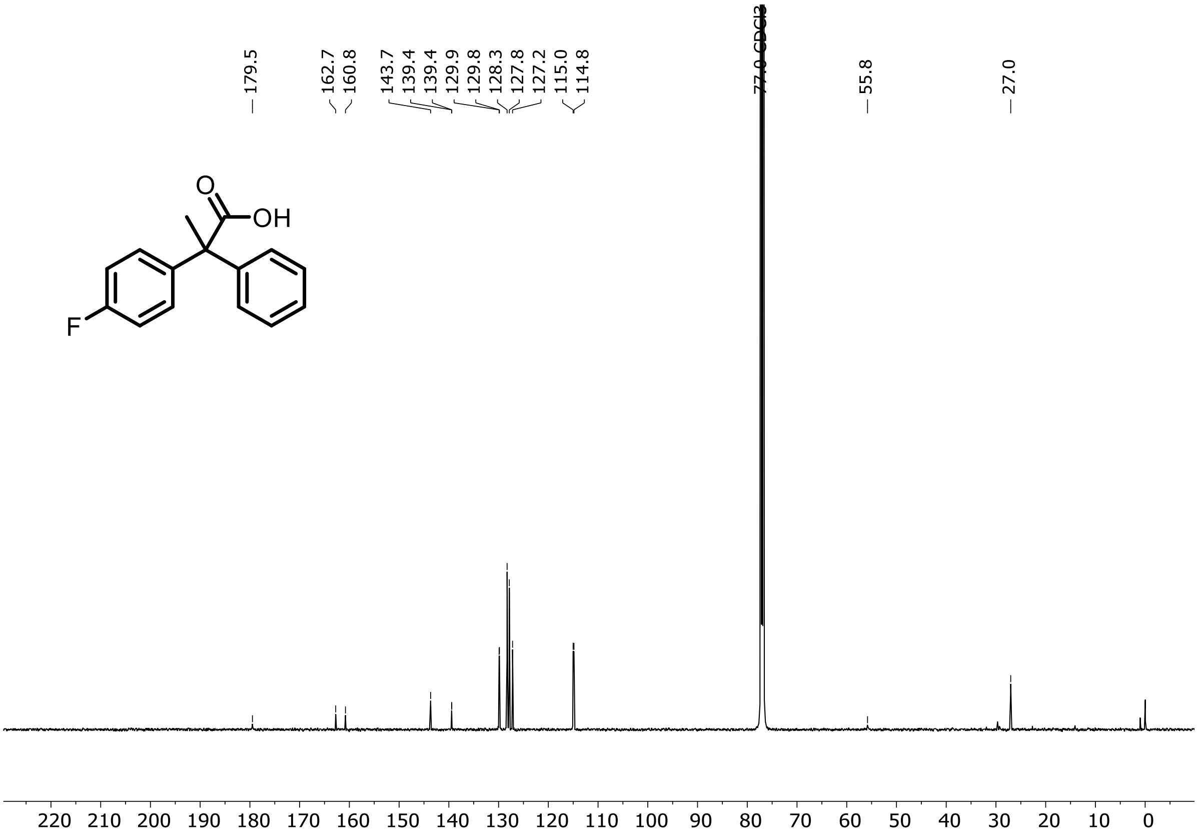

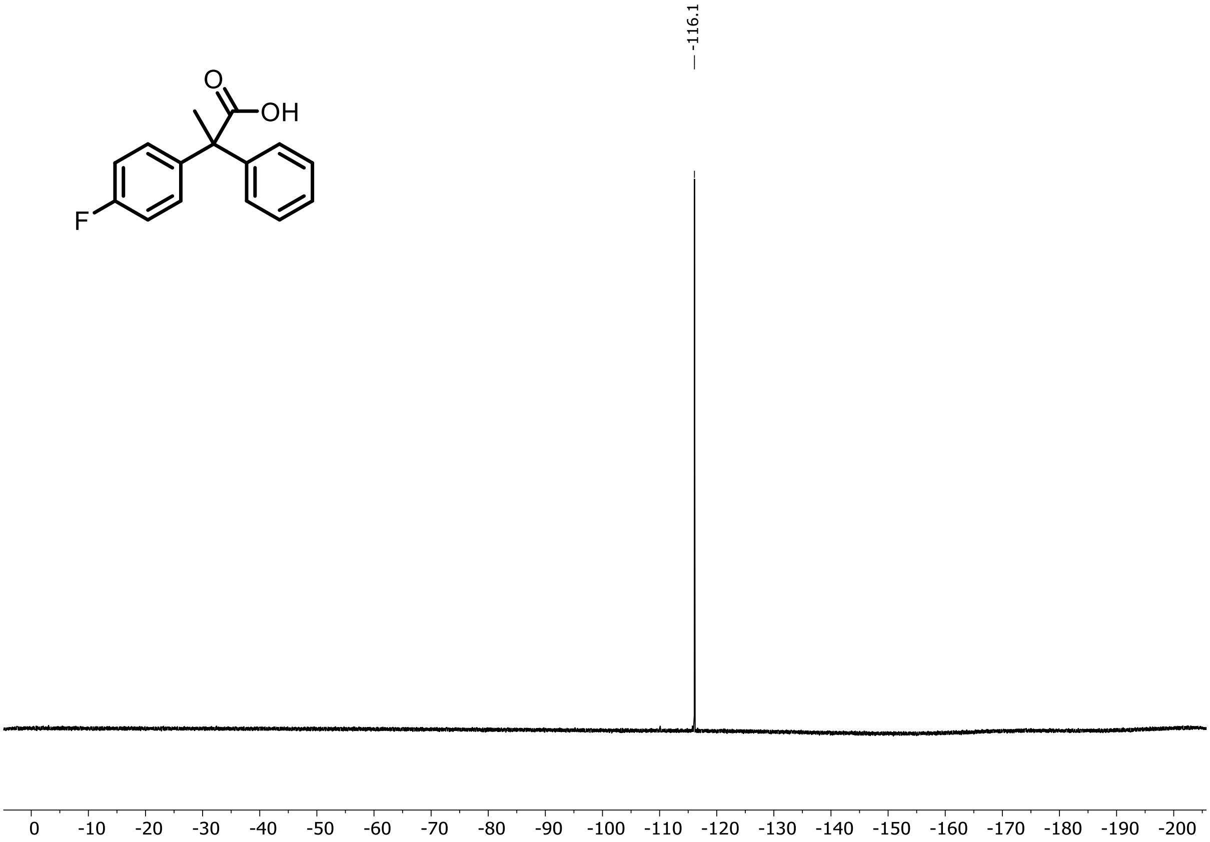

2-phenyl-2-(o-tolyl)propanoic acid (2s)

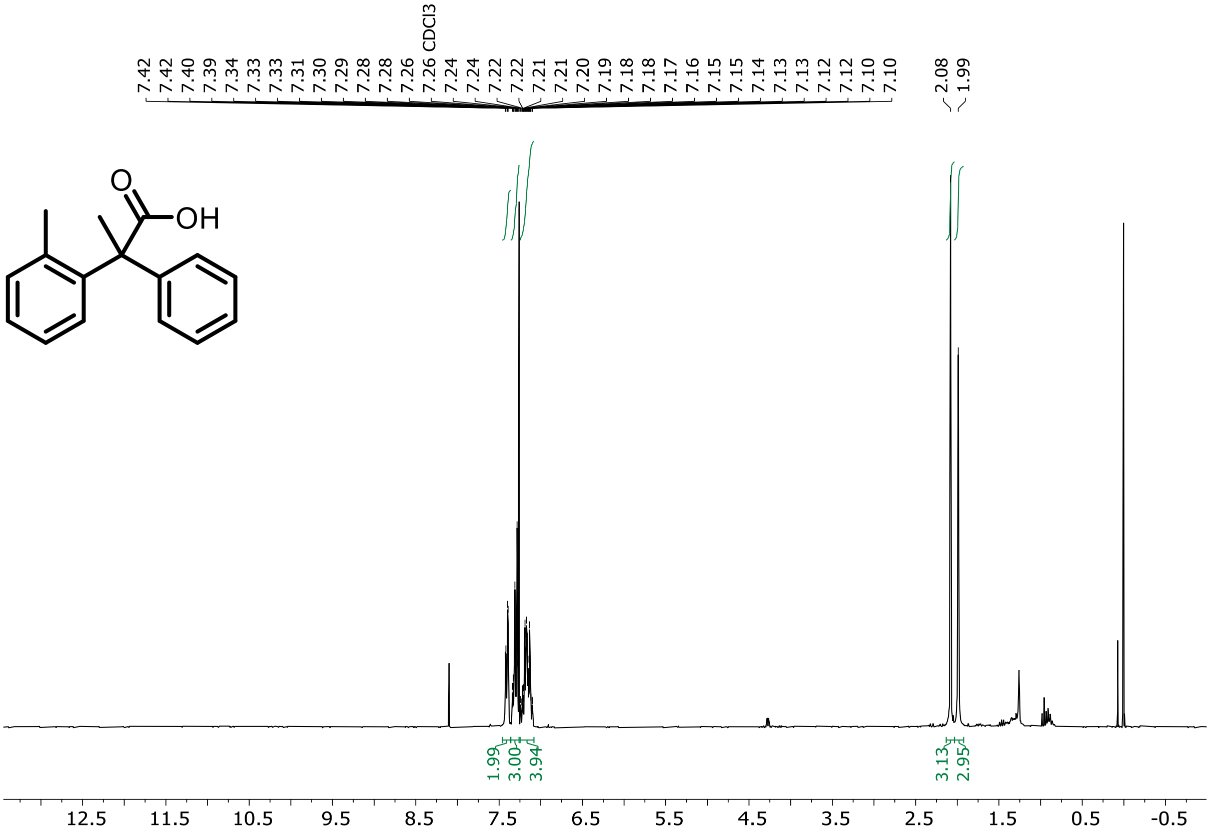

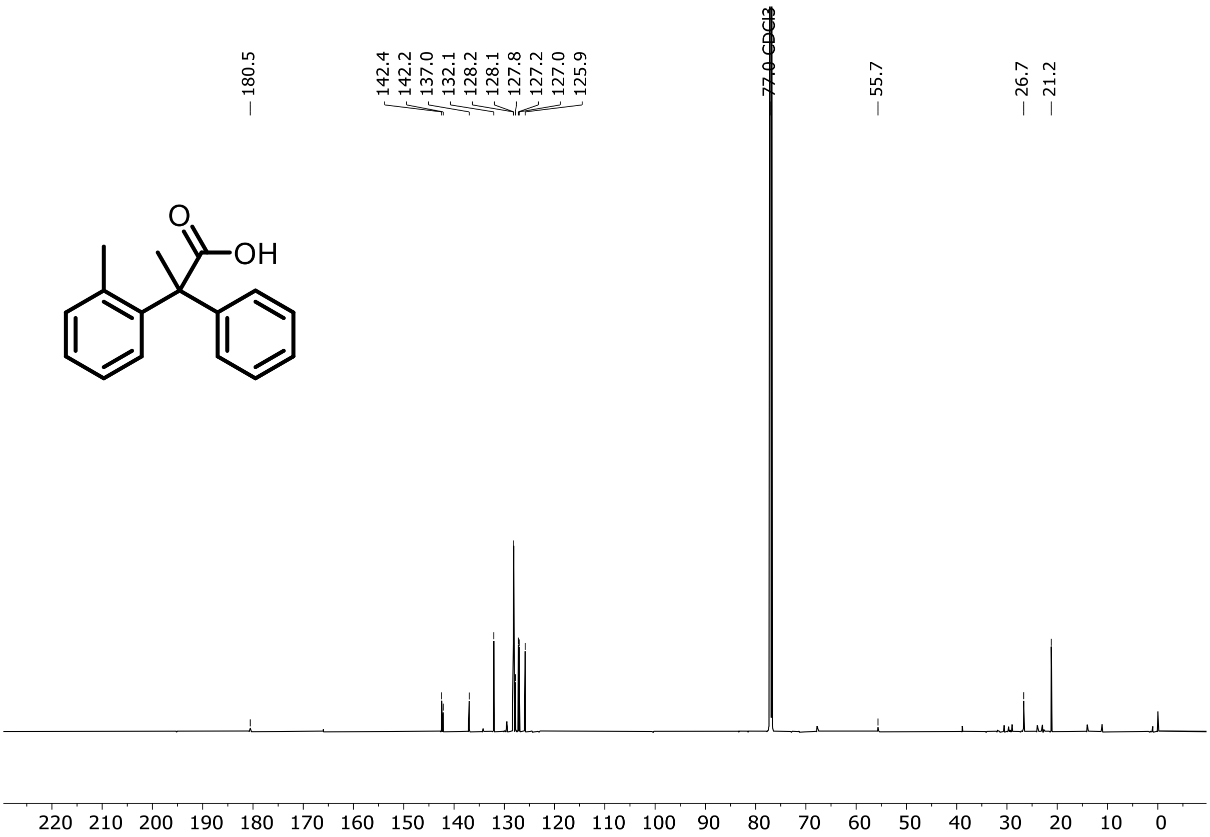

2-([1,1'-biphenyl]-4-yl)-2-phenylacetic acid (2t)

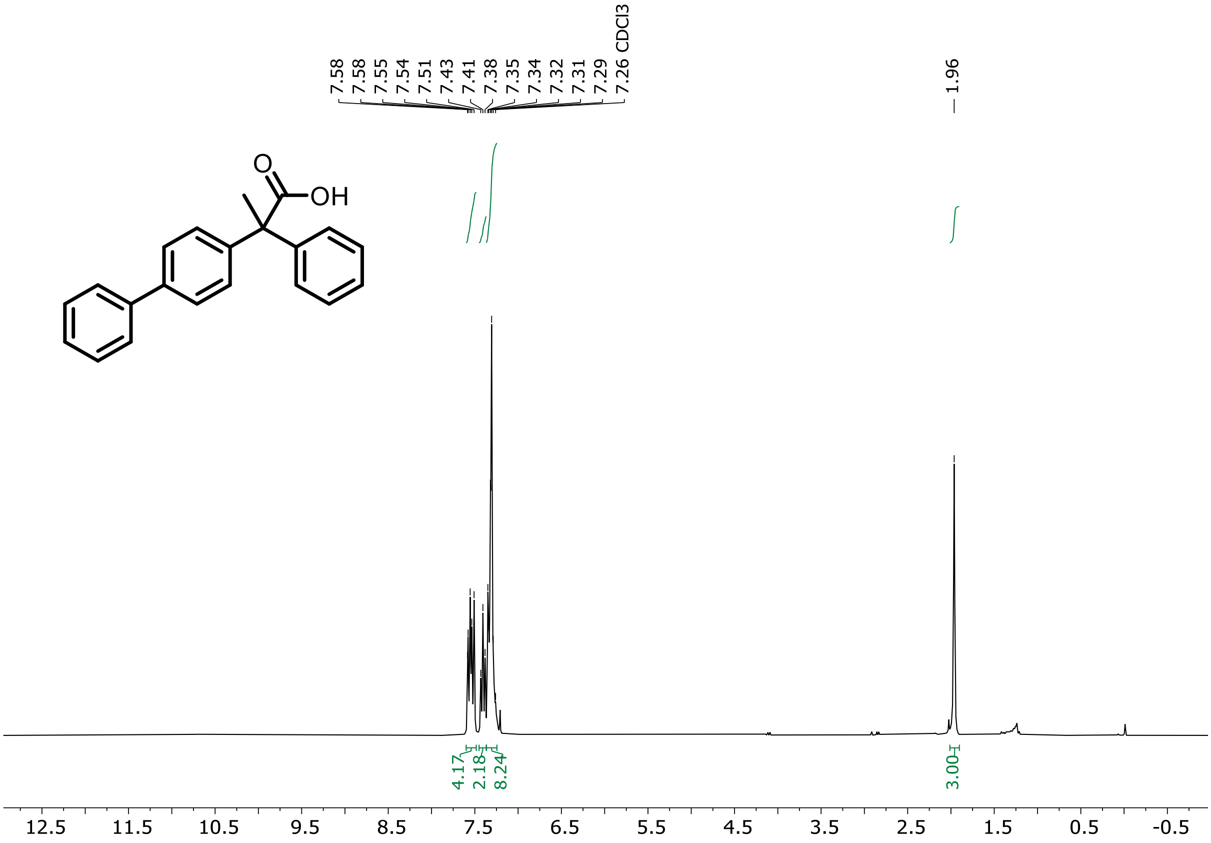

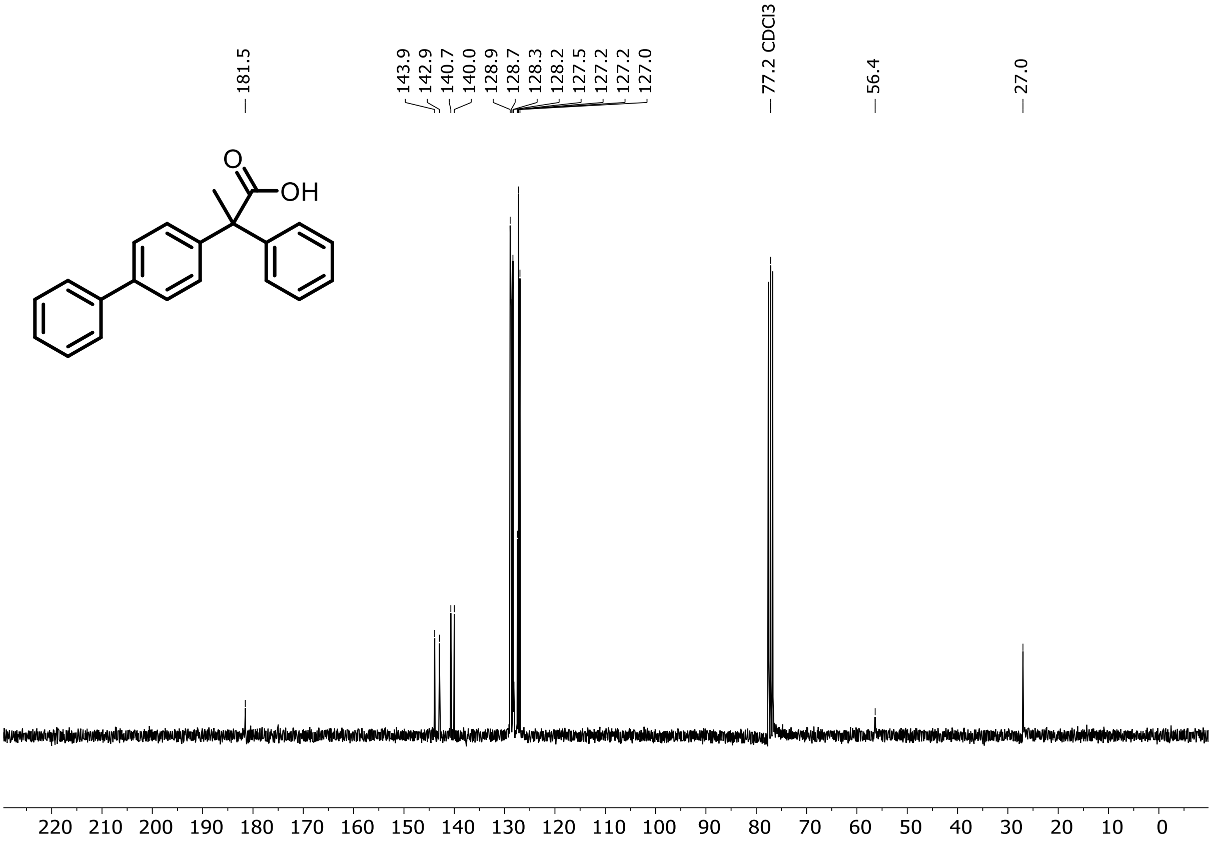

2,2-bis(4-fluorophenyl)propanoic acid (2u)

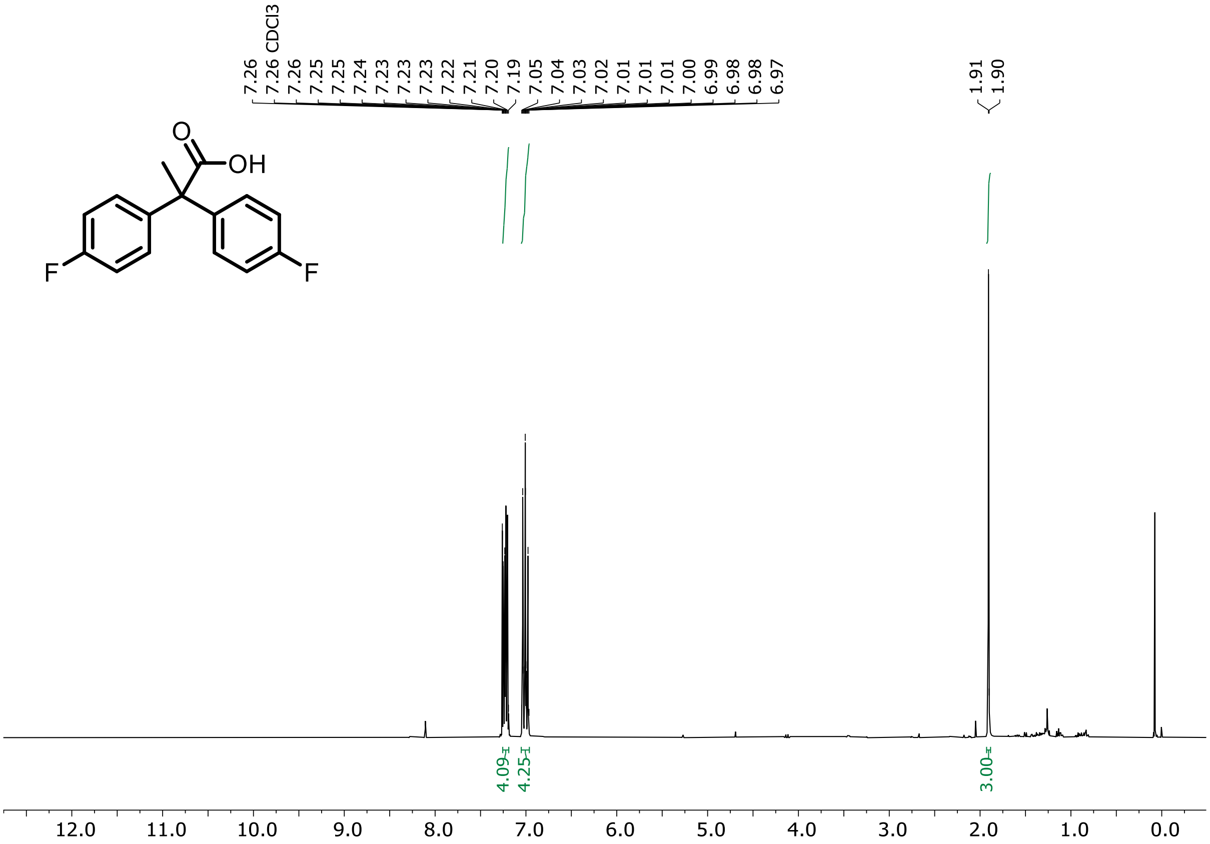

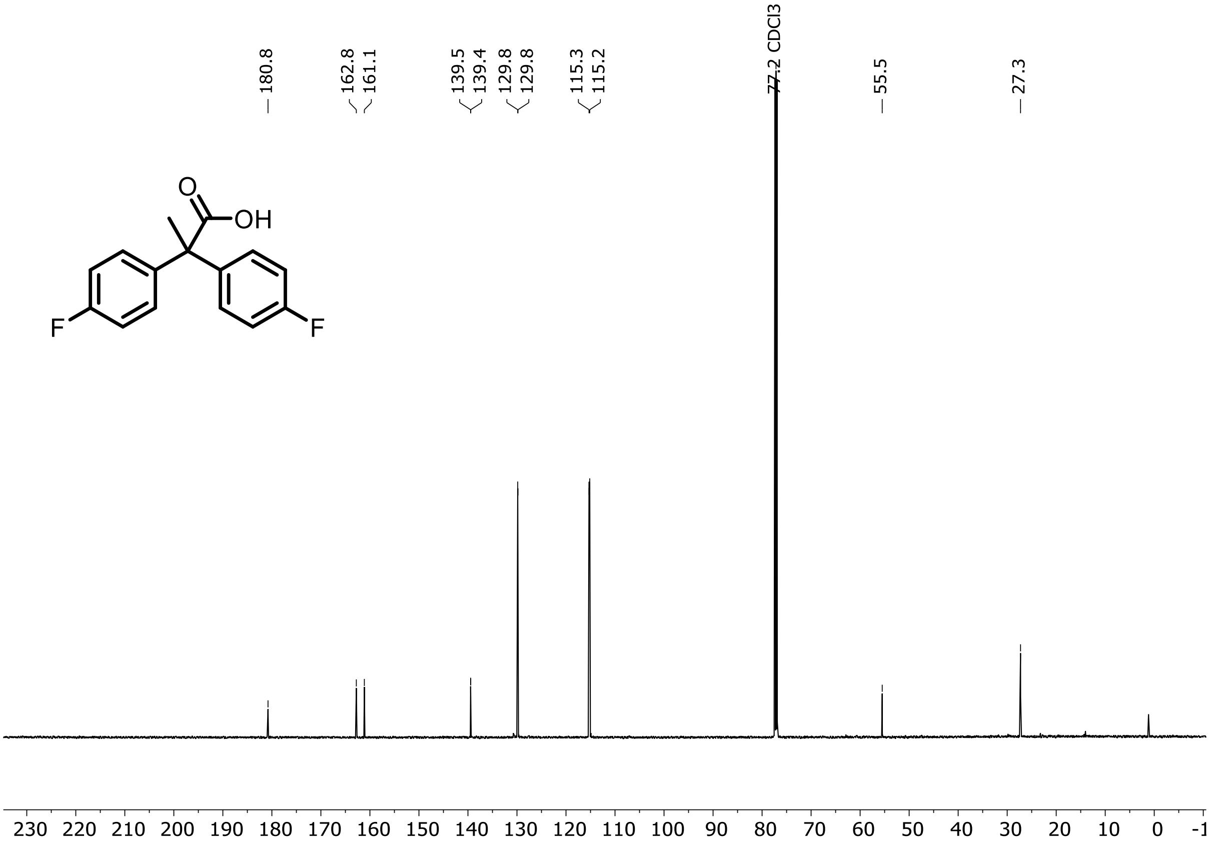

180.8
162.8
161.1
139.5
139.4
129.8
129.8
115.3
115.2
77.2 CDCl3
55.5
27.3
O
OH
F
F
230 220 210 200 190 180 170 160 150 140 130 120 110 100 90 80 70 60 50 40 30 20 10 0

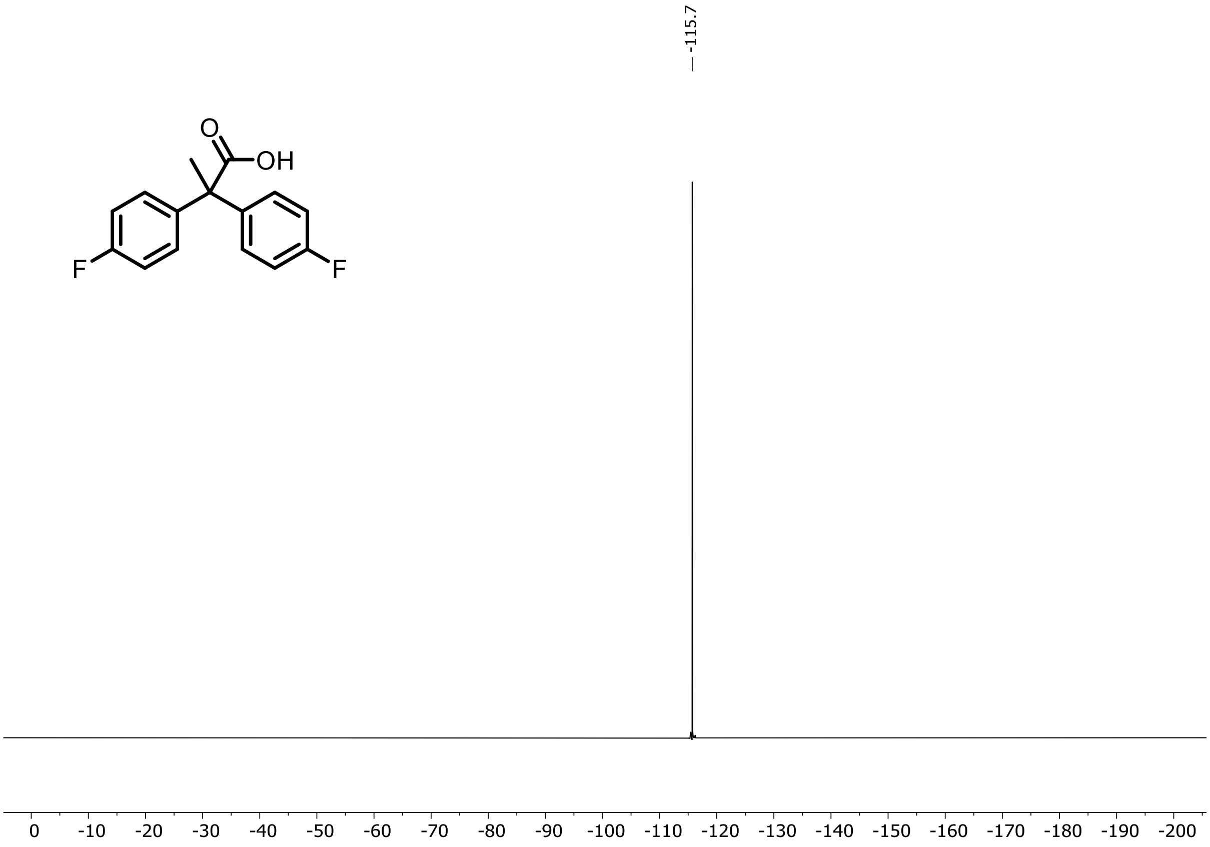

-115.7
O
OH
F
F
0 -10 -20 -30 -40 -50 -60 -70 -80 -90 -100 -110 -120 -130 -140 -150 -160 -170 -180 -190 -200

2,2-bis(4-chlorophenyl)propanoic acid (2v)

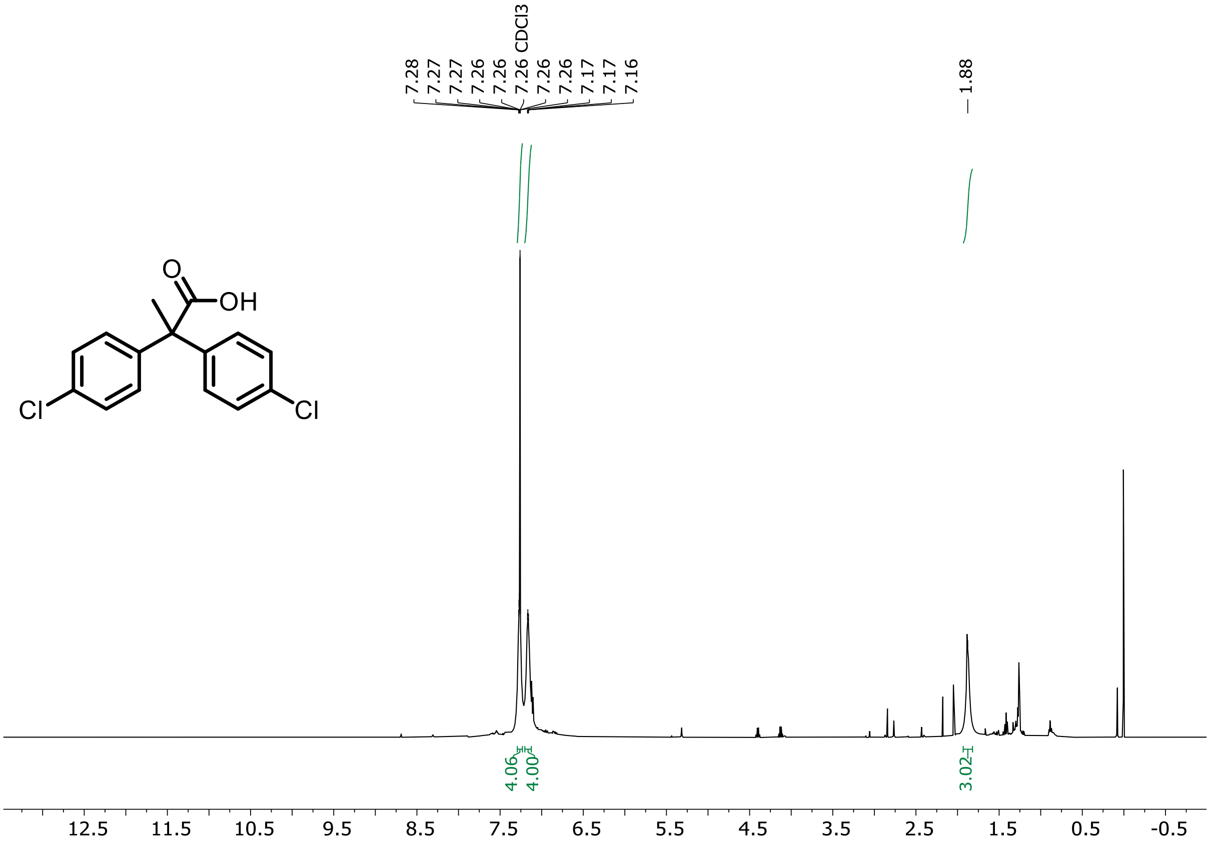

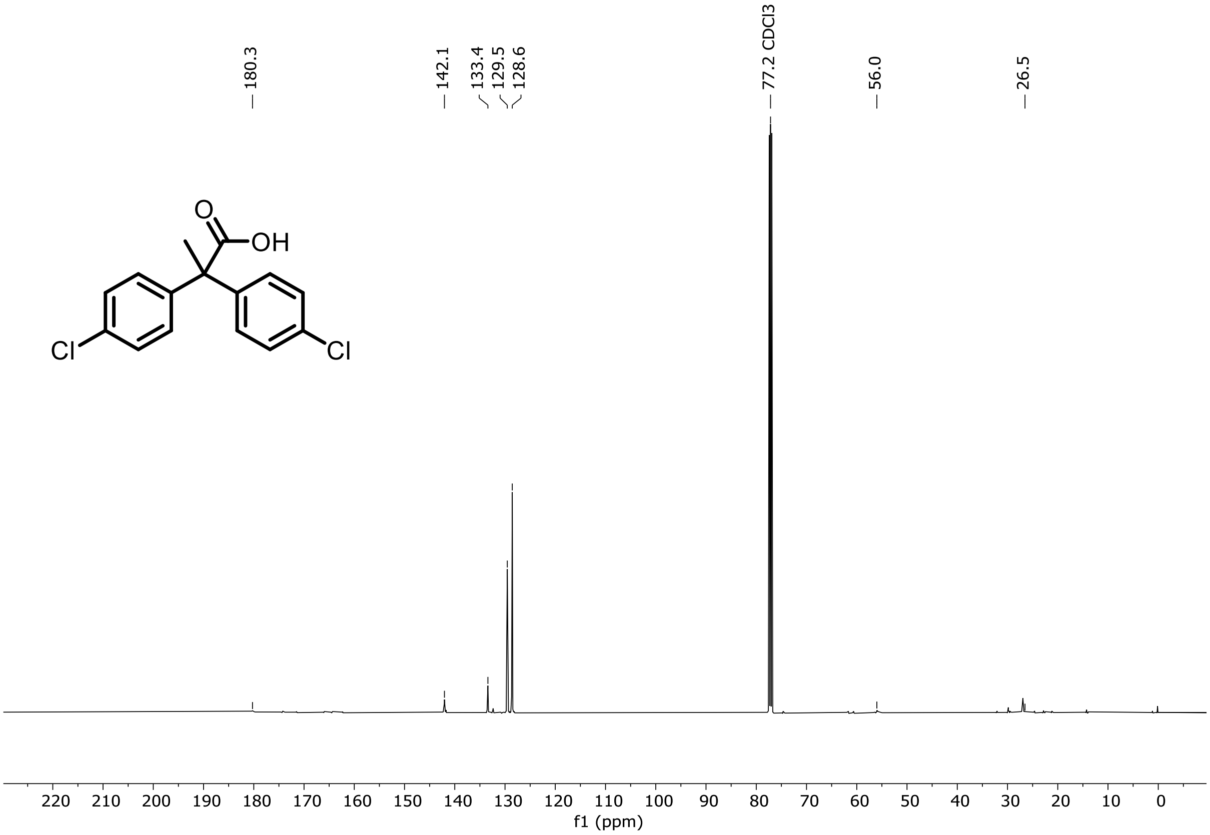

2,2-diphenylbutanoic acid (2w)

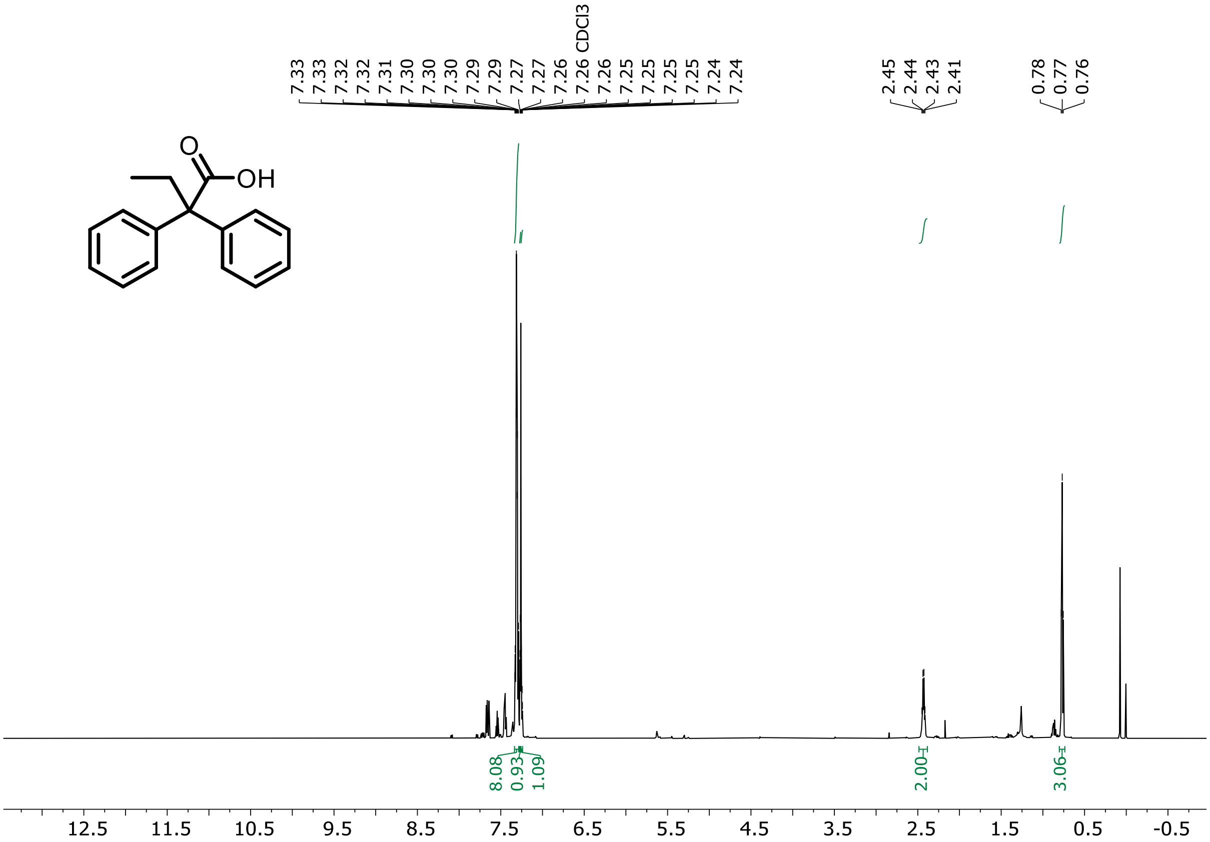

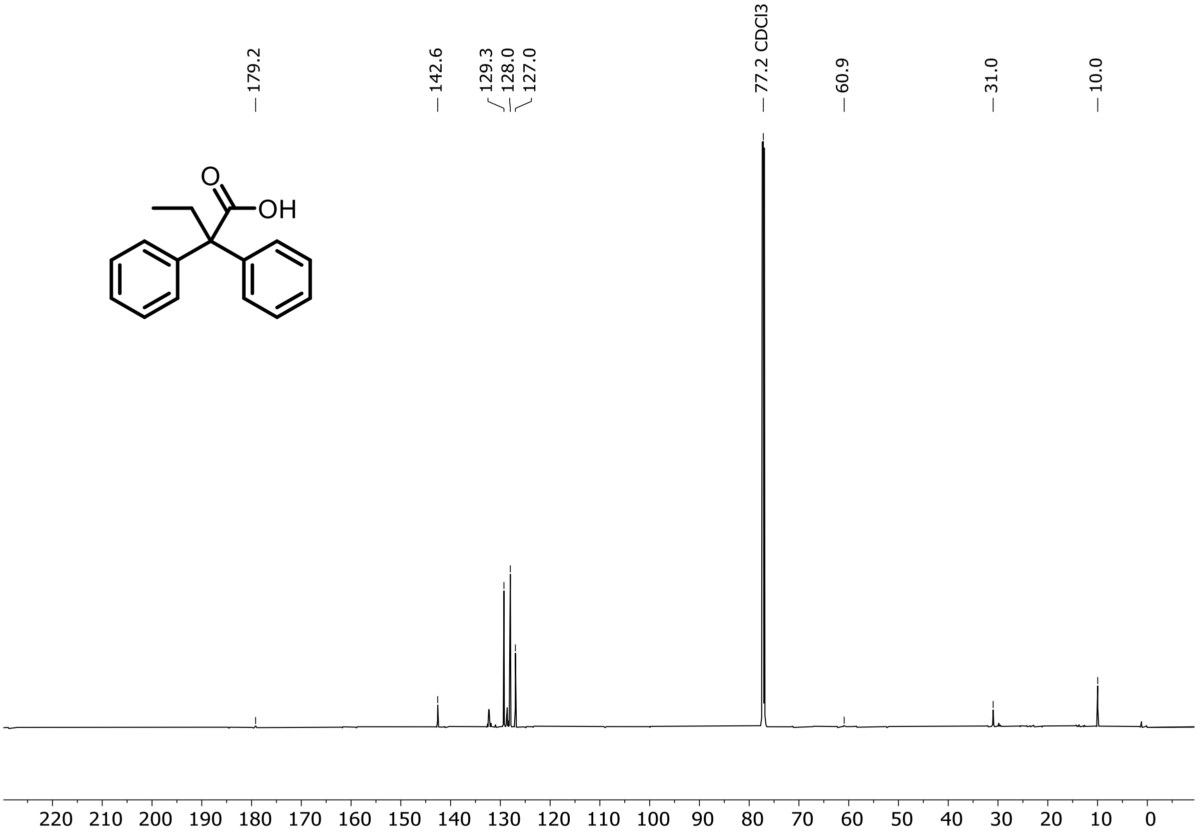

2-phenyl-2-(thiophen-2-yl)propanoic acid (2x)

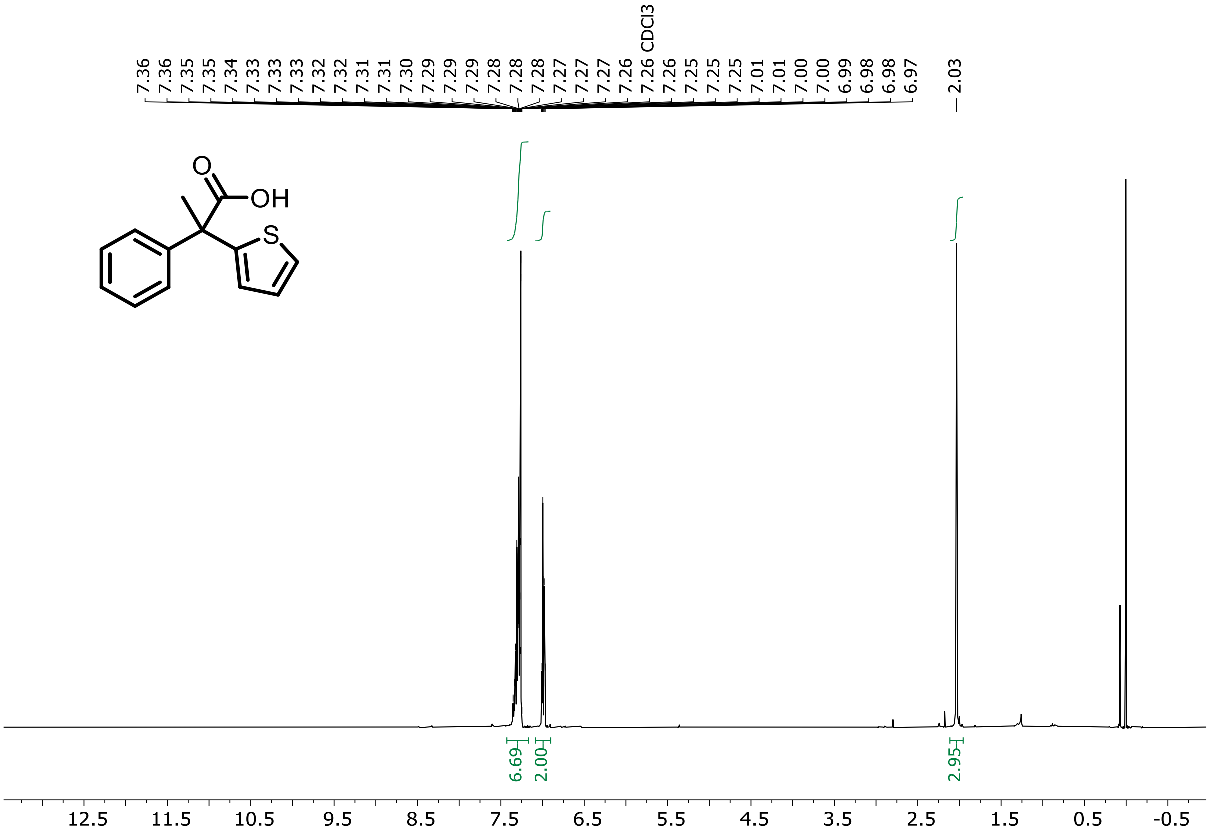

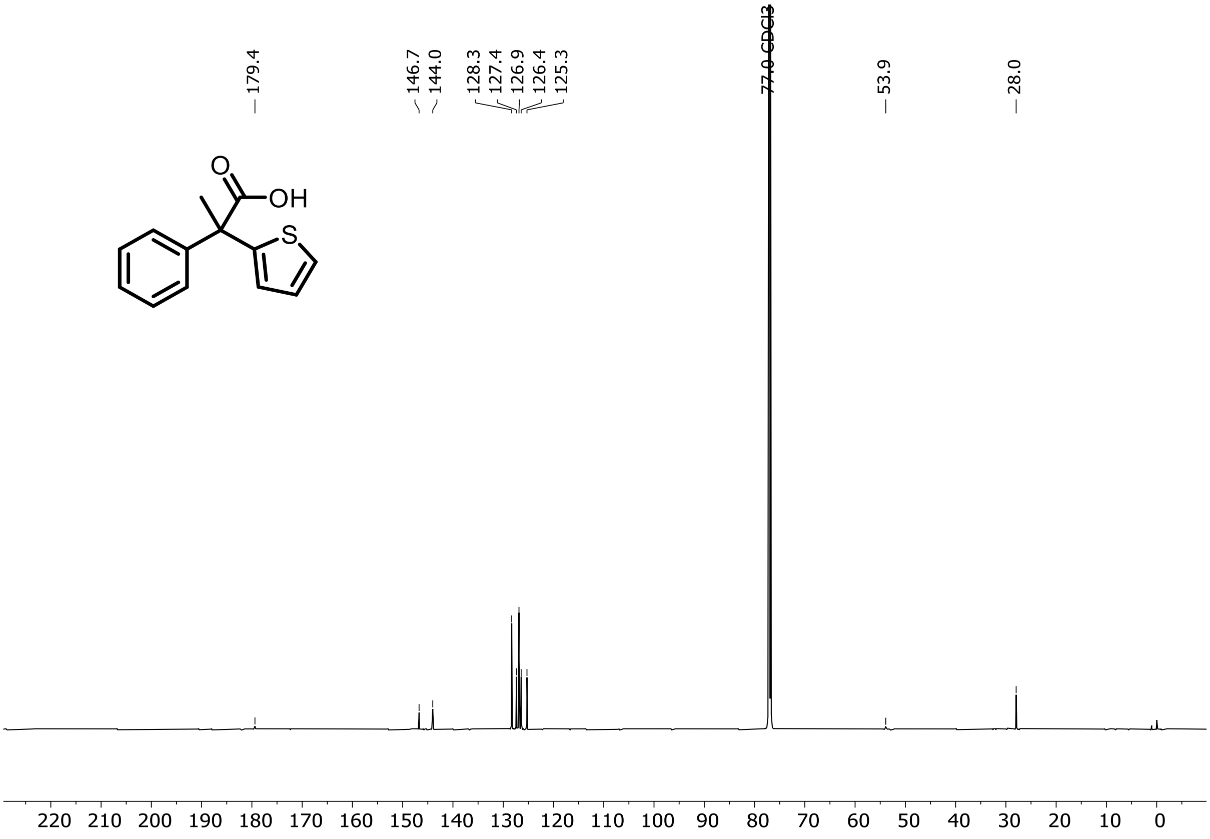

5-methyl-10,11-dihydro-5H-dibenzo[*a*,*d*][7]annulene-5-carboxylic acid (2y)

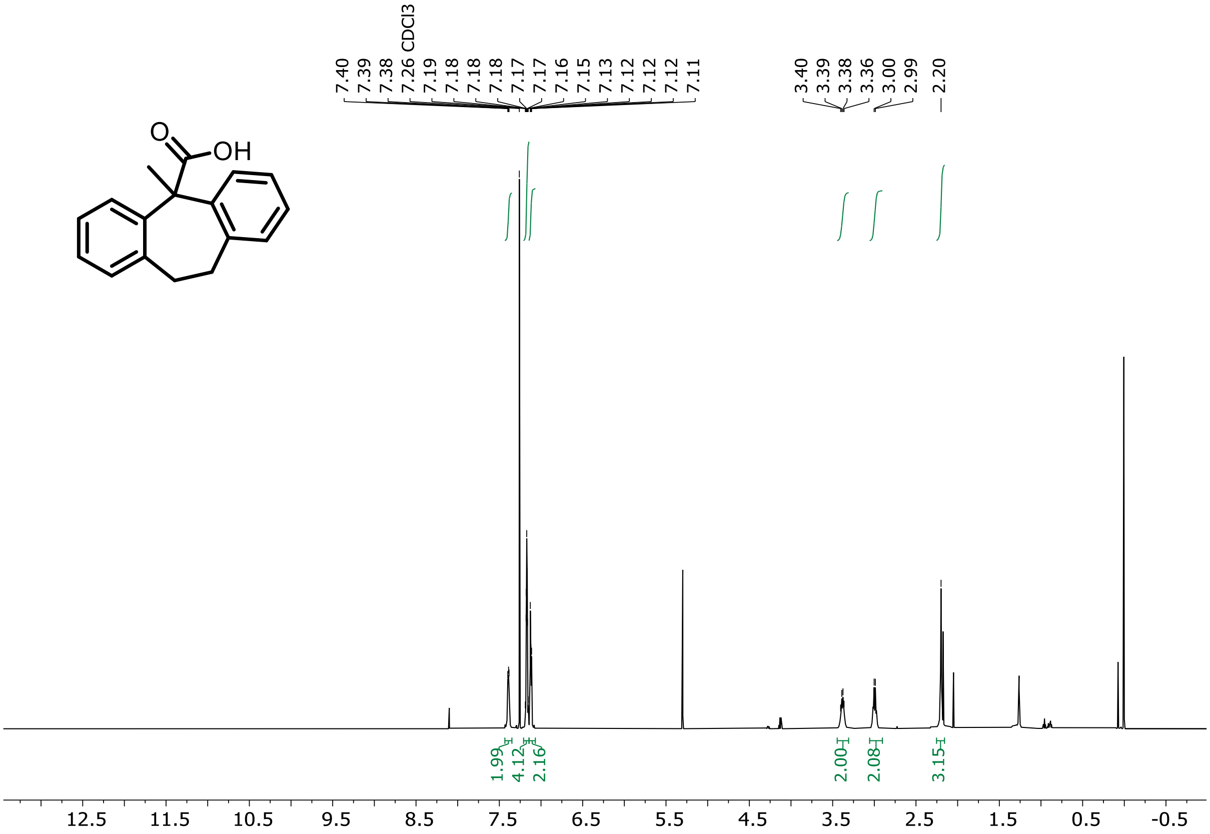

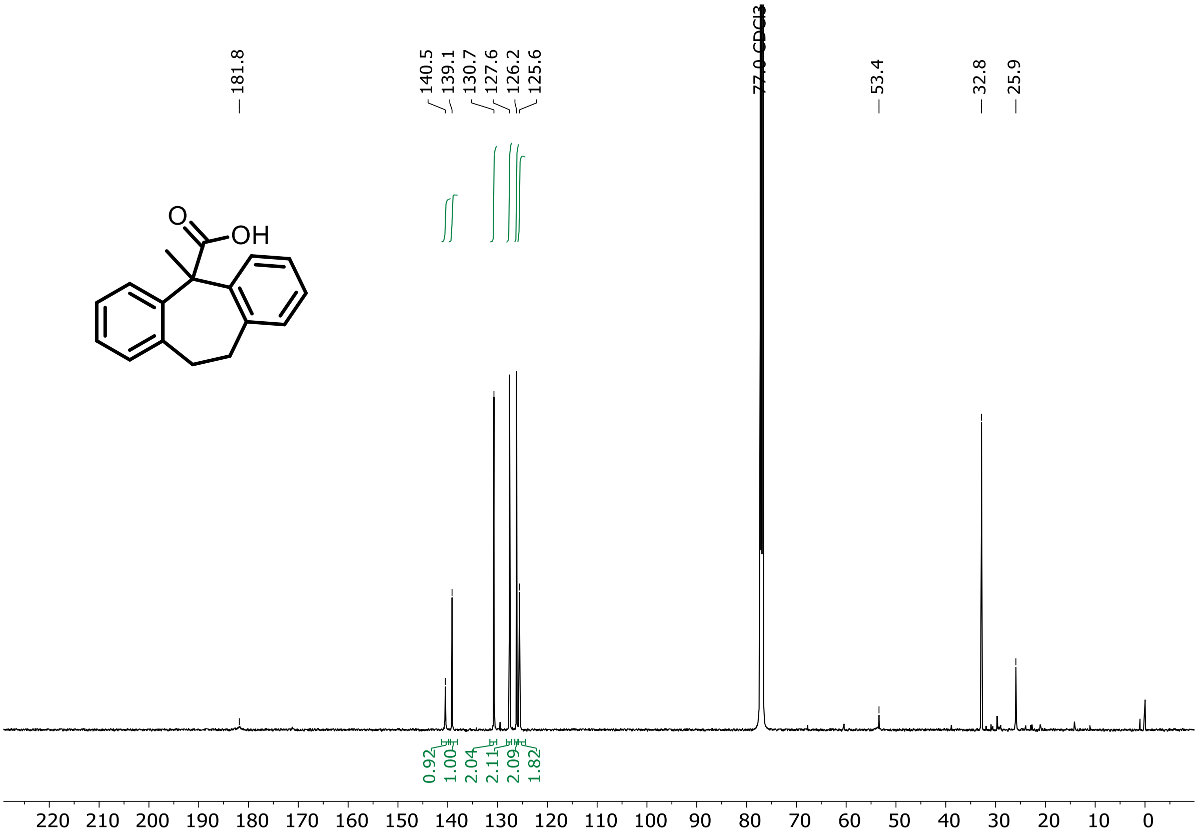

2-methyl-4,4-diphenylbut-3-enoic acid (2z major) and α-Methyl-α-[(1*E*)-3-phenyl-1-propen-1-yl]benzeneacetic acid (2z minor)

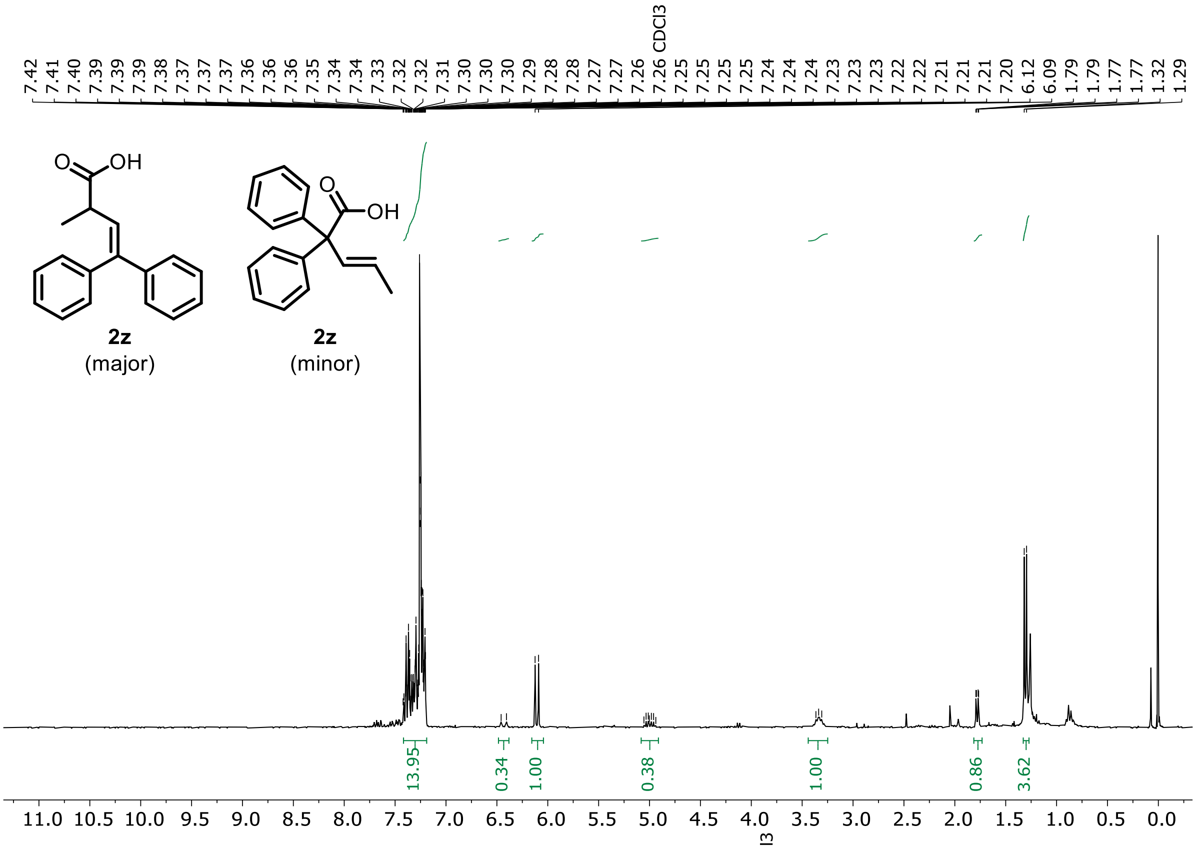

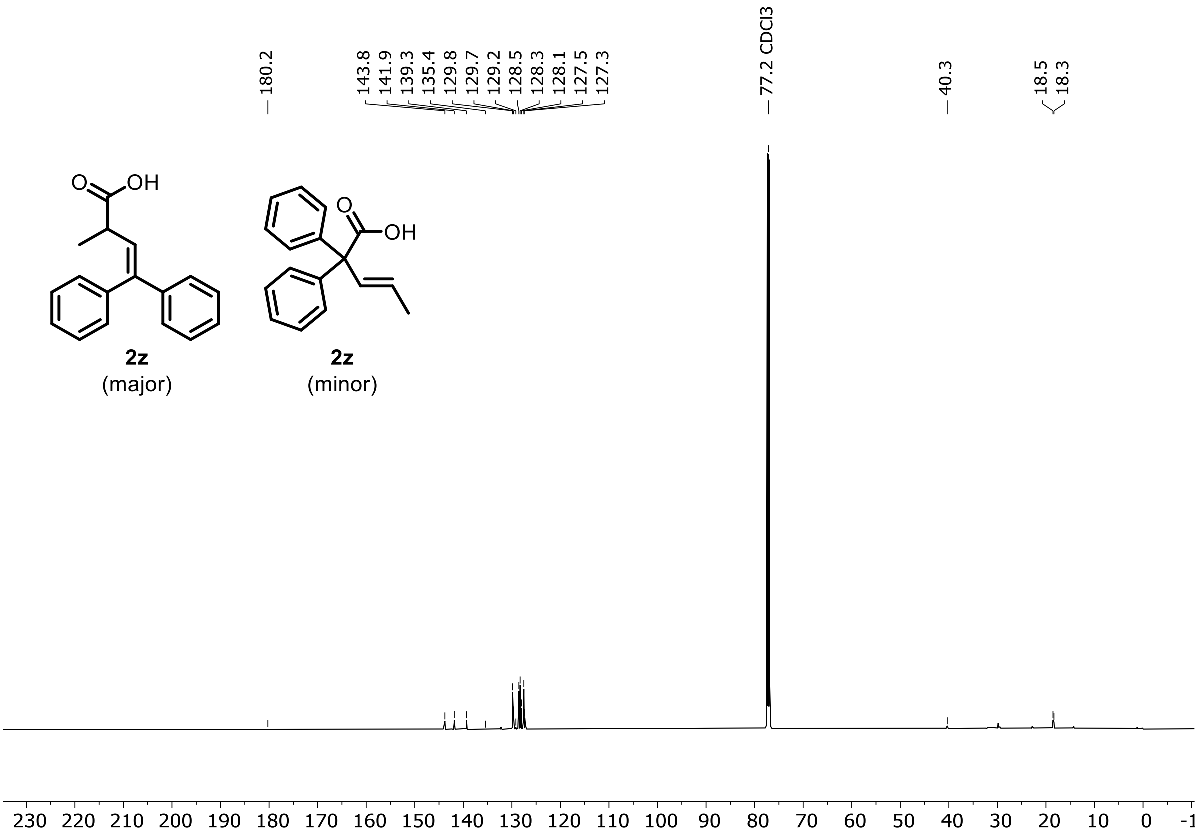

3-methyl-3-phenylindolin-2-one (2aa)

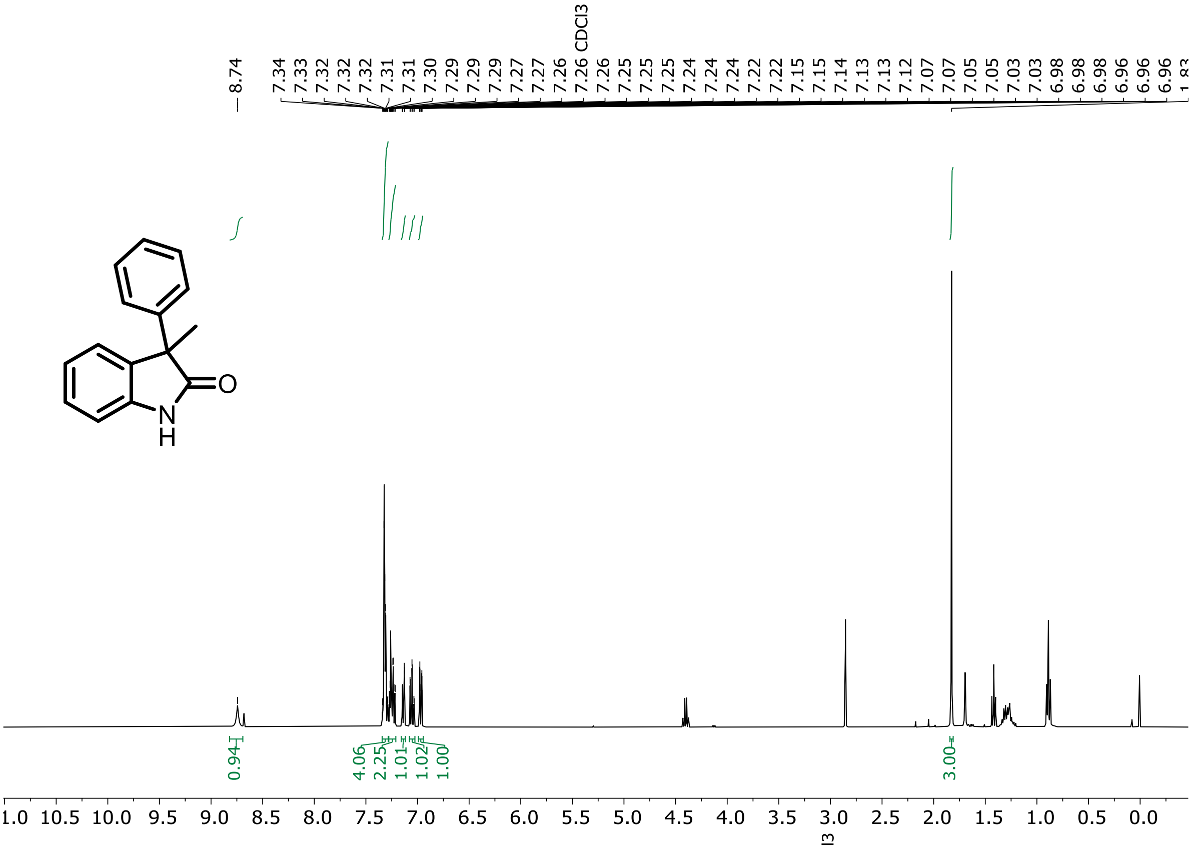

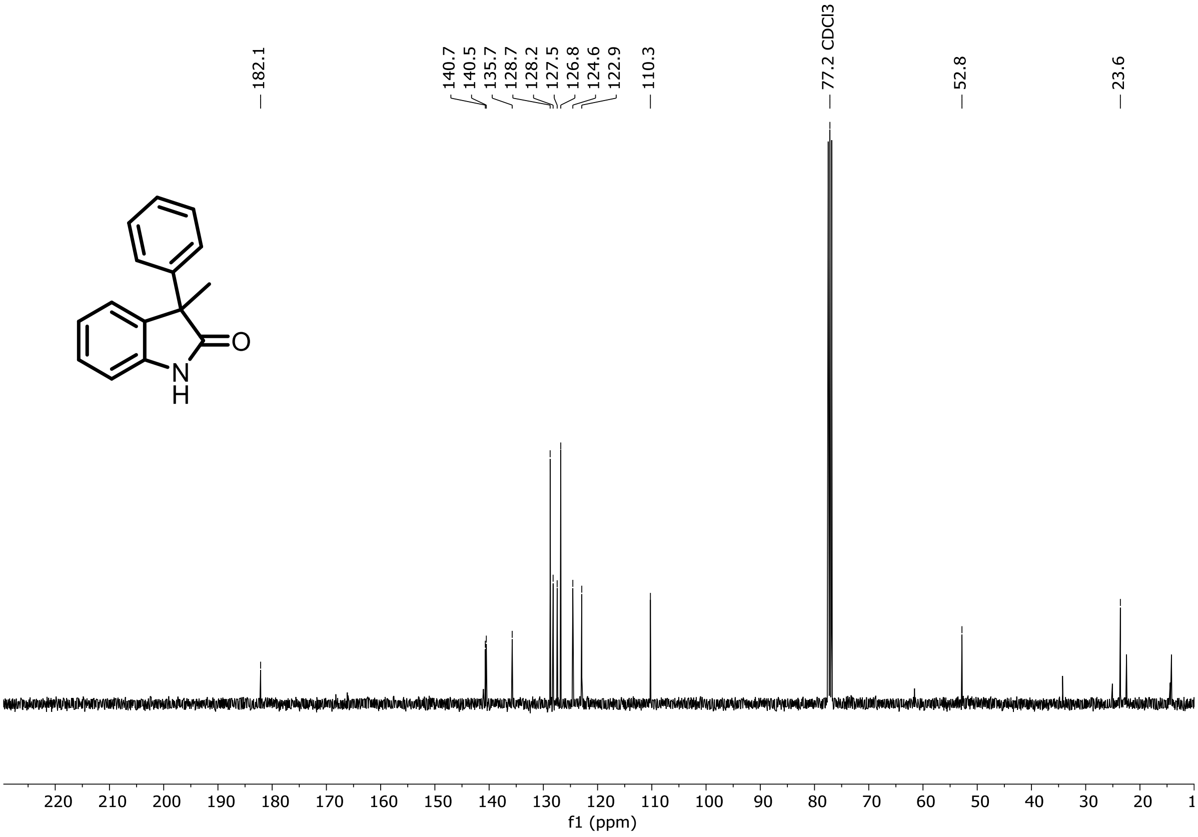

3-(4-chlorophenyl)-3-methylindolin-2-one (2ab)

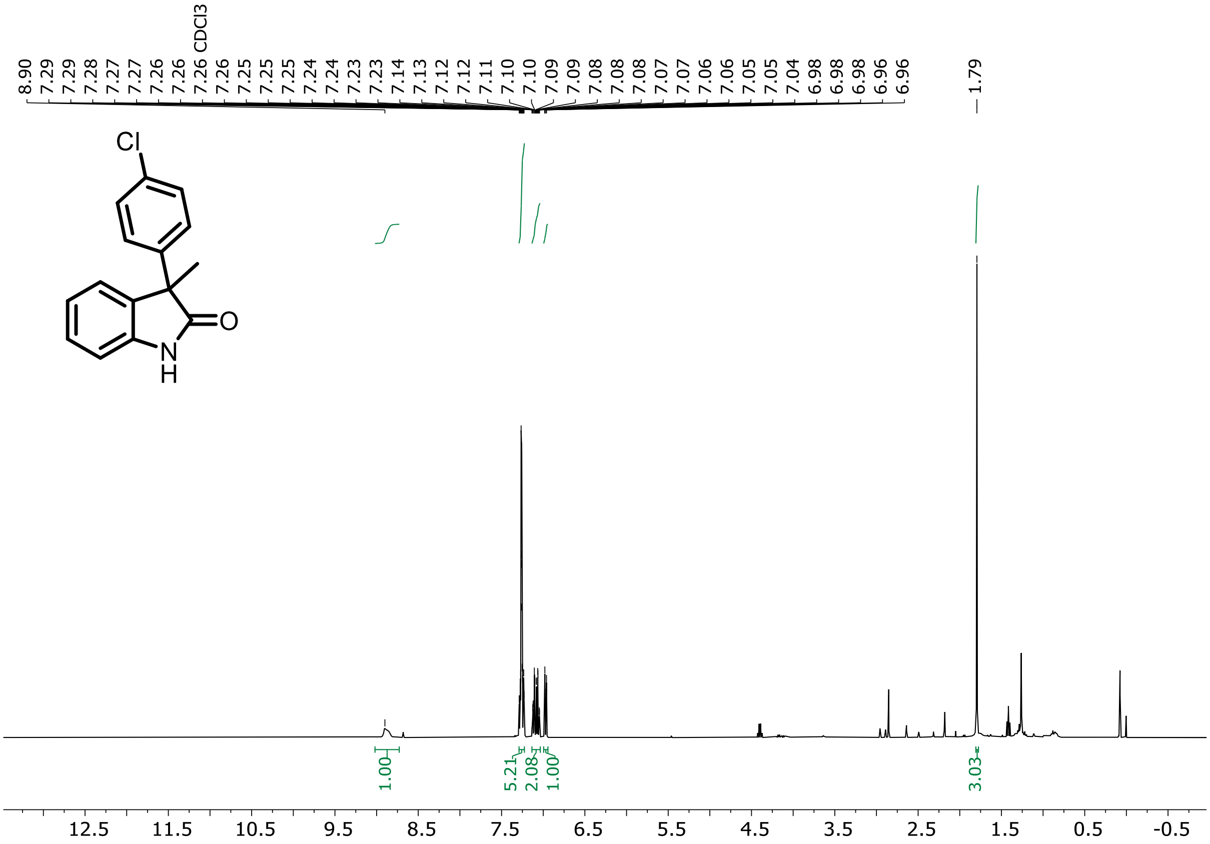

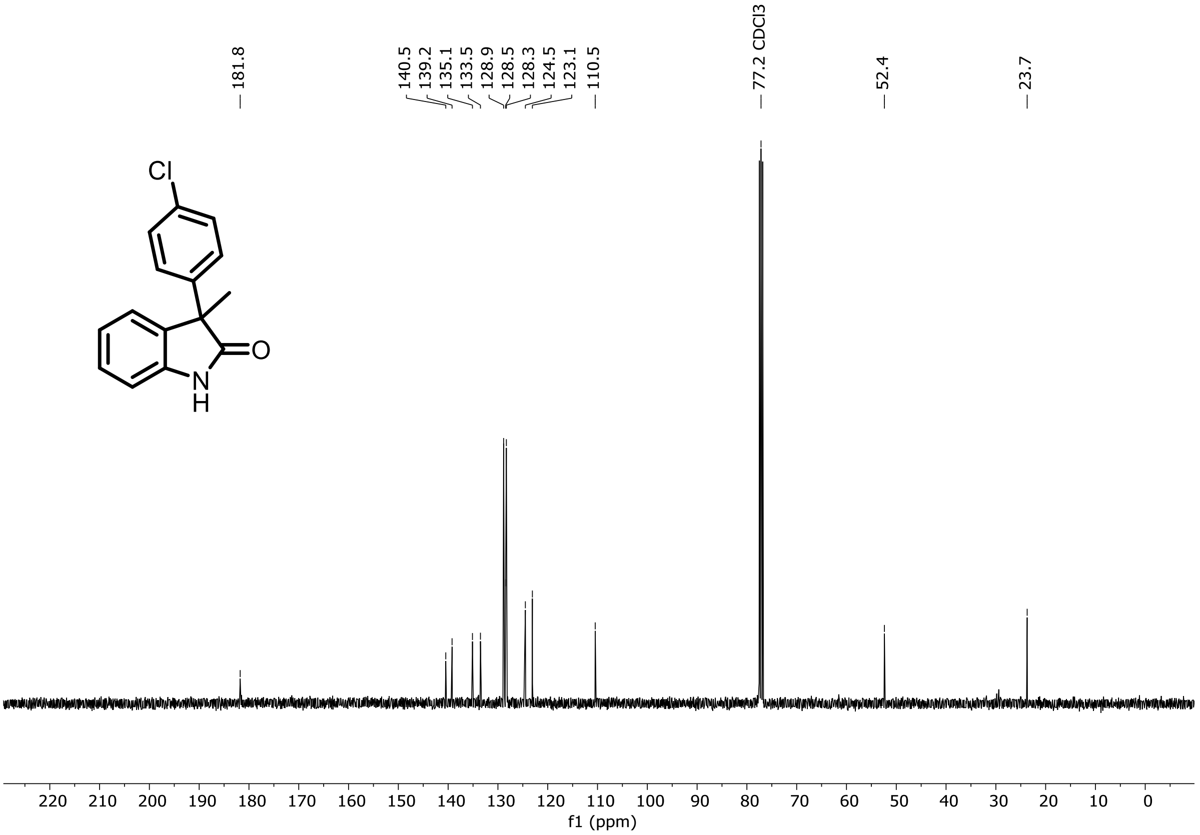

6-chloro-3-methyl-3-phenylindolin-2-one (2ac)

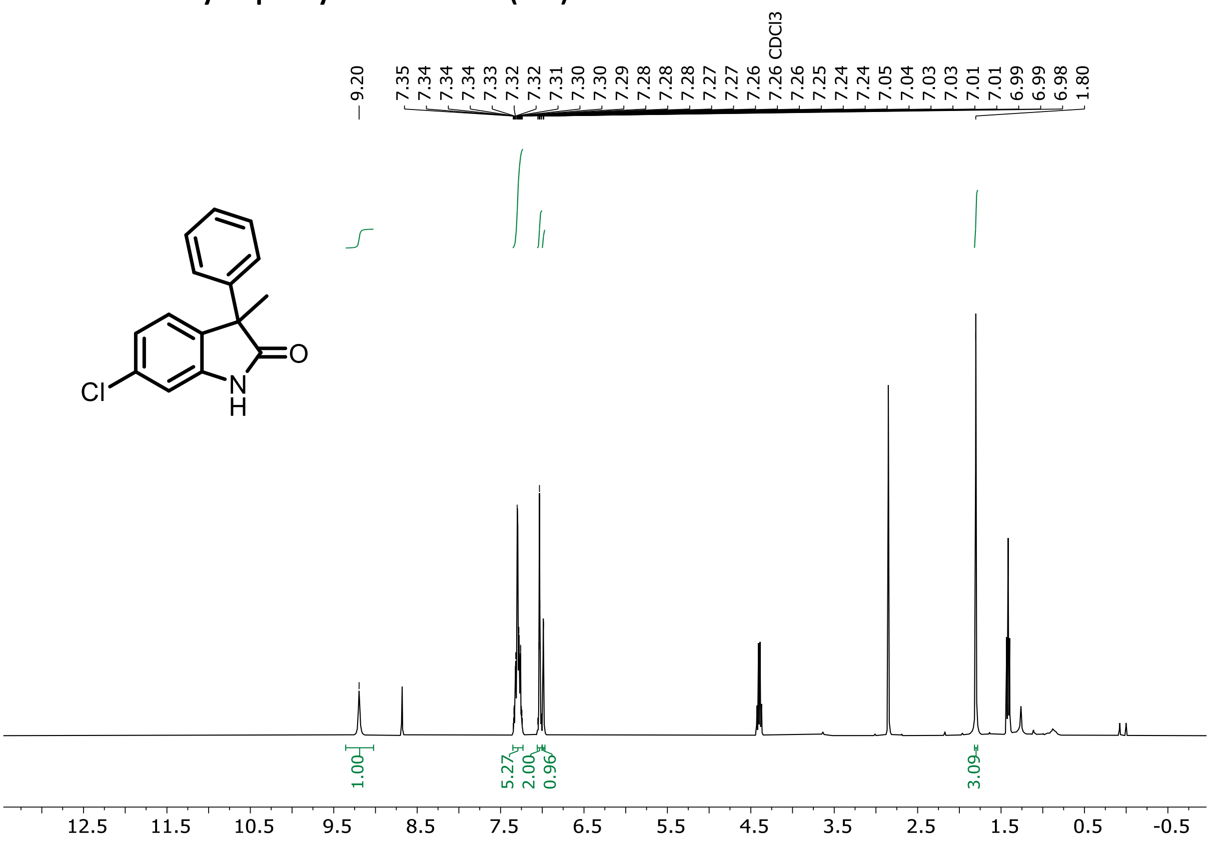

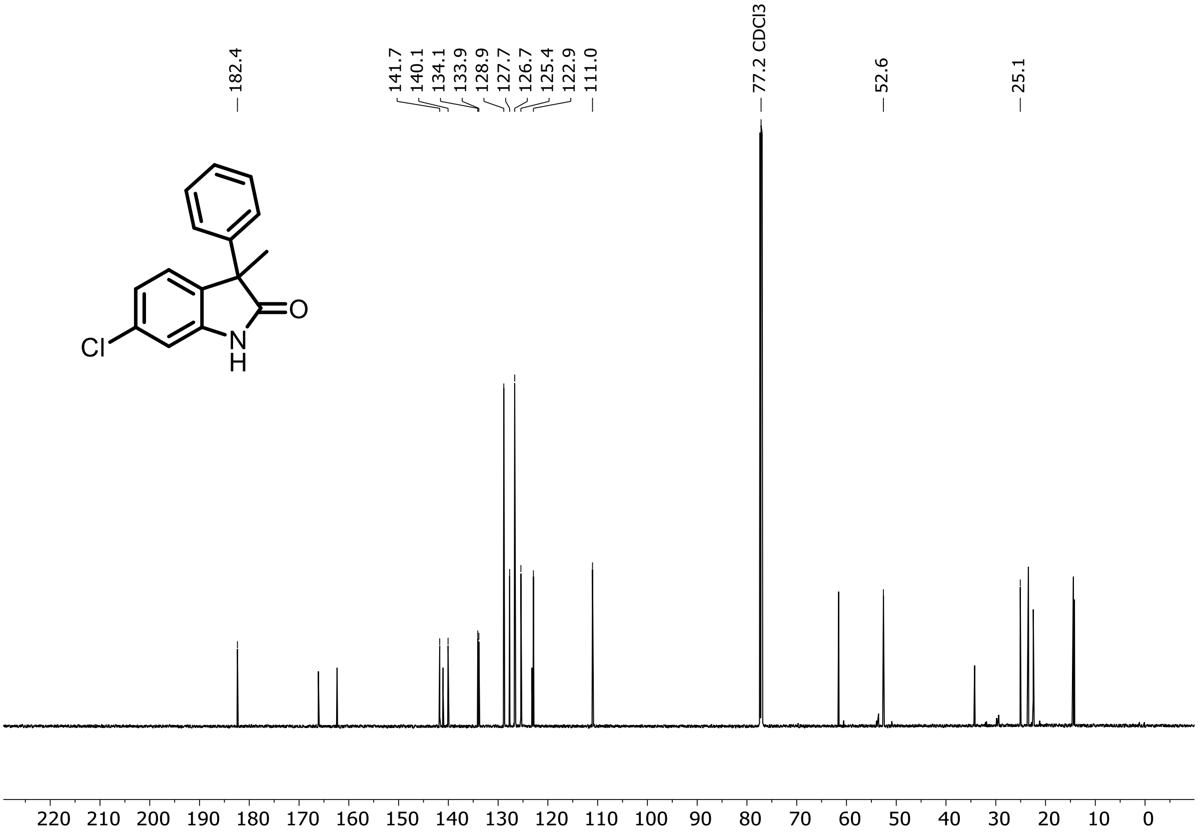

2-(3-fluoro-[1,1'-biphenyl]-4-yl)propanoic acid (2ad)

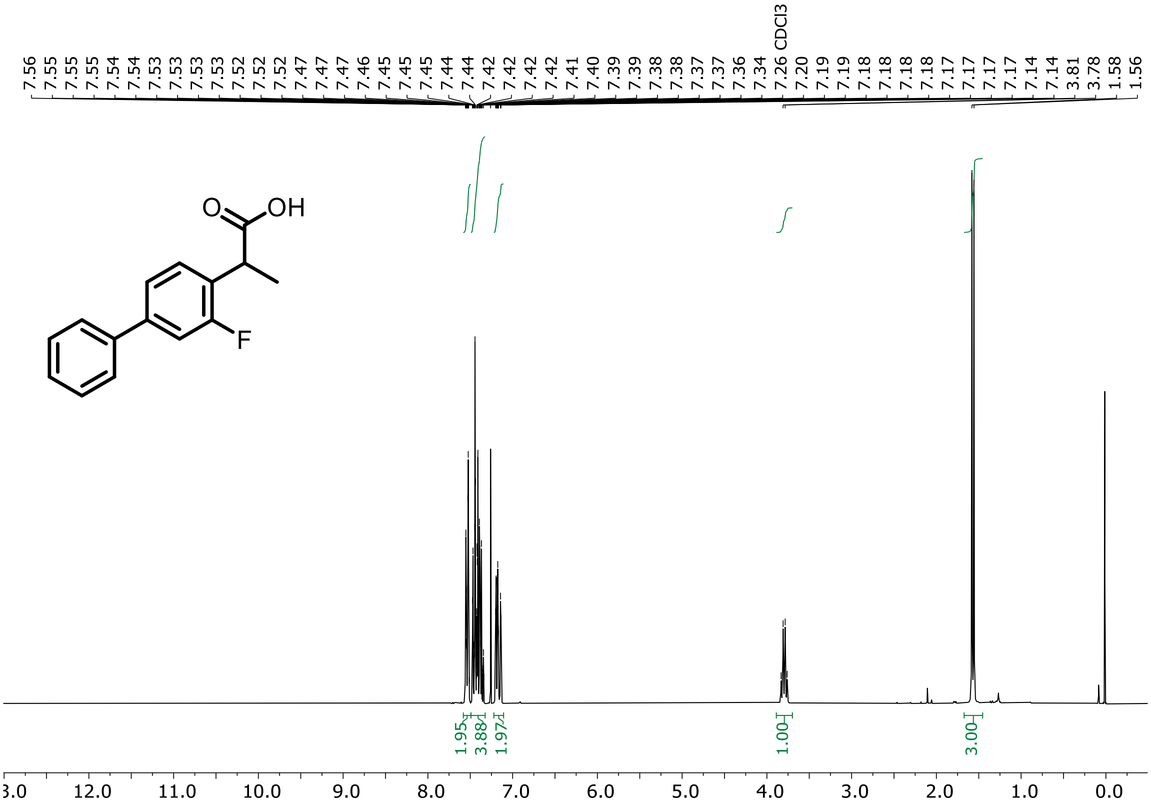

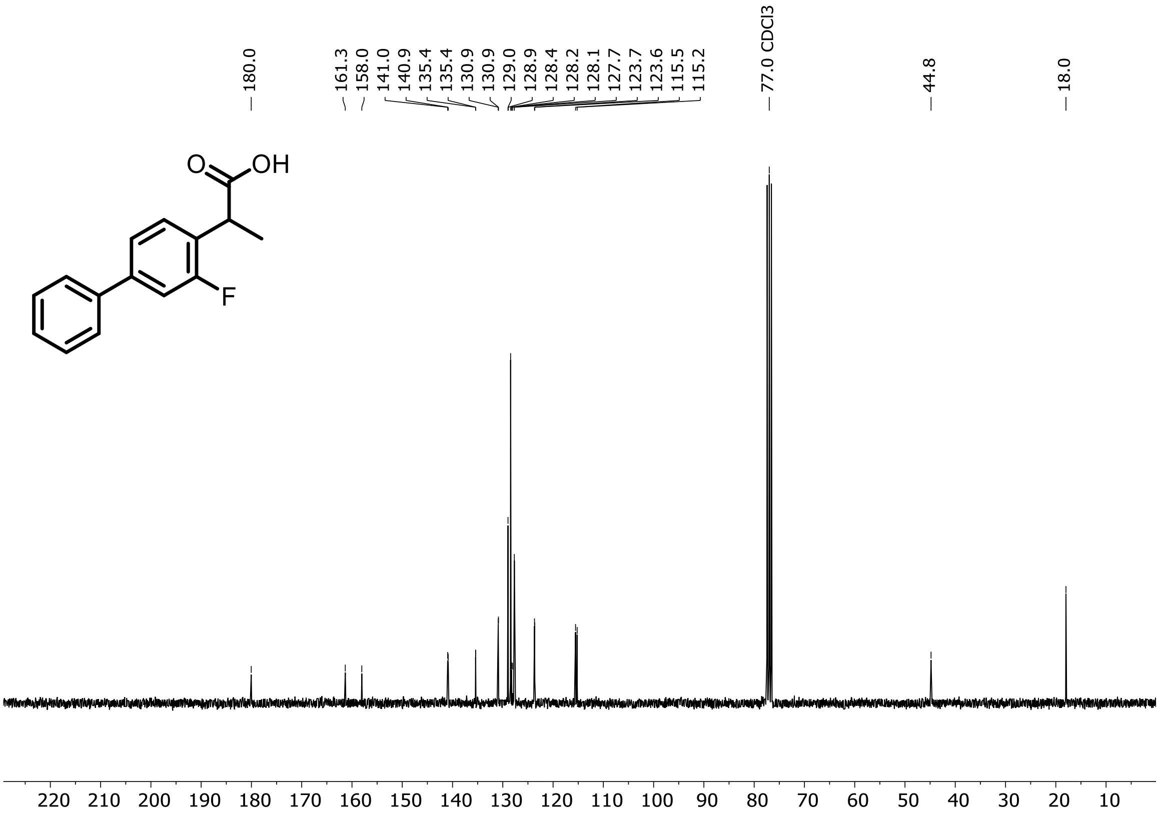

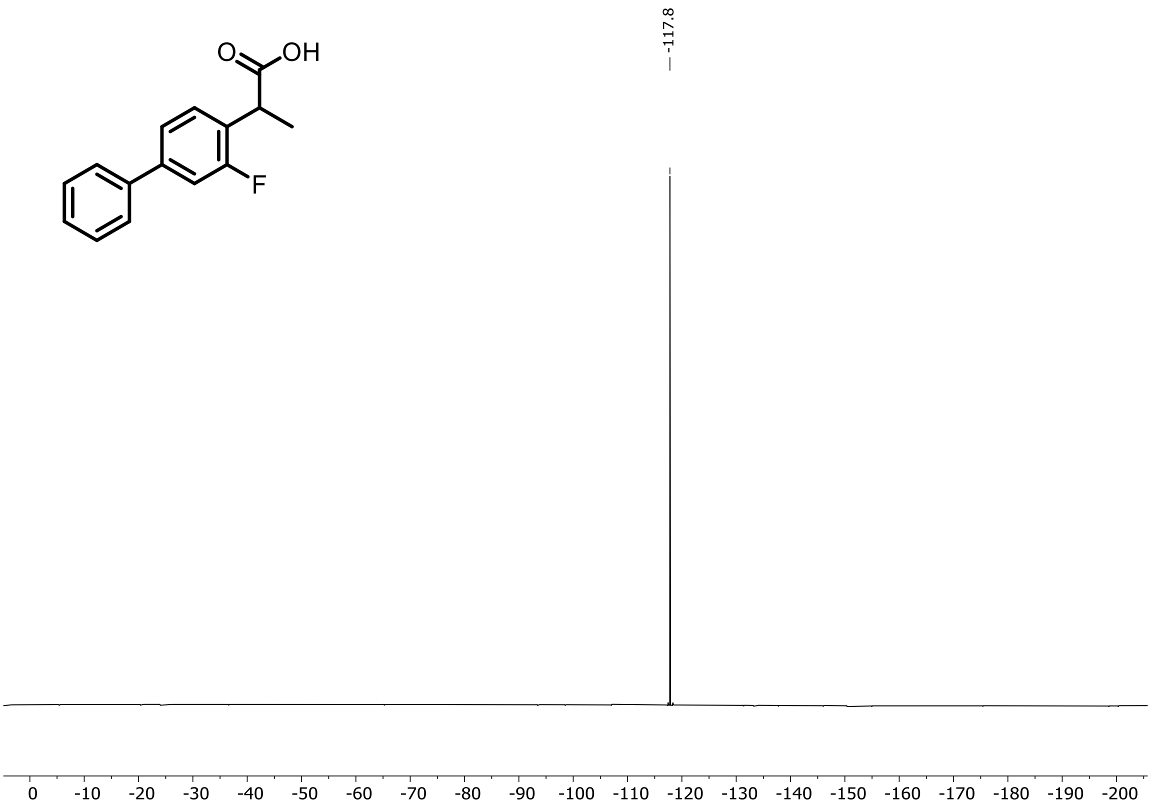

2-(4-chlorophenyl)-2-(4-((1-isopropoxy-2-methyl-1-oxopropan-2-yl)oxy)phenyl)propanoic acid (2ae)

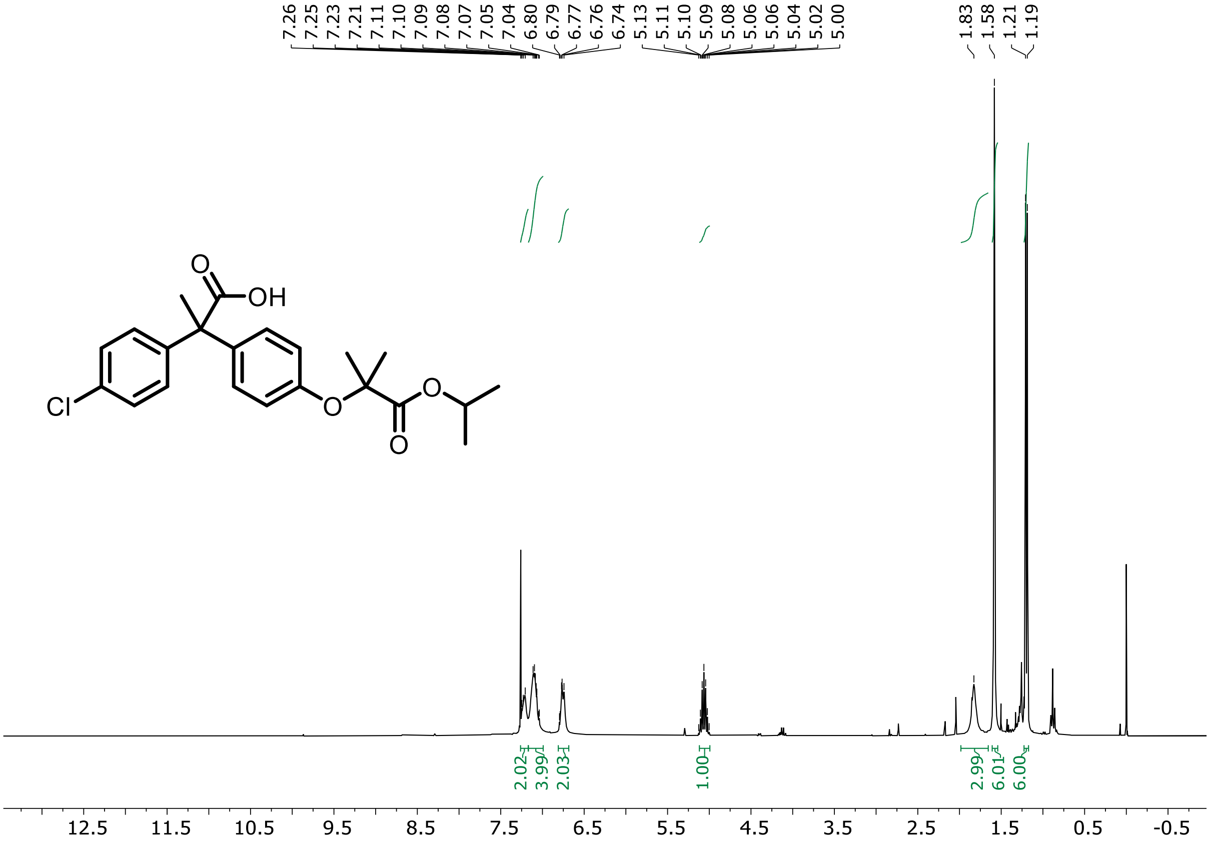

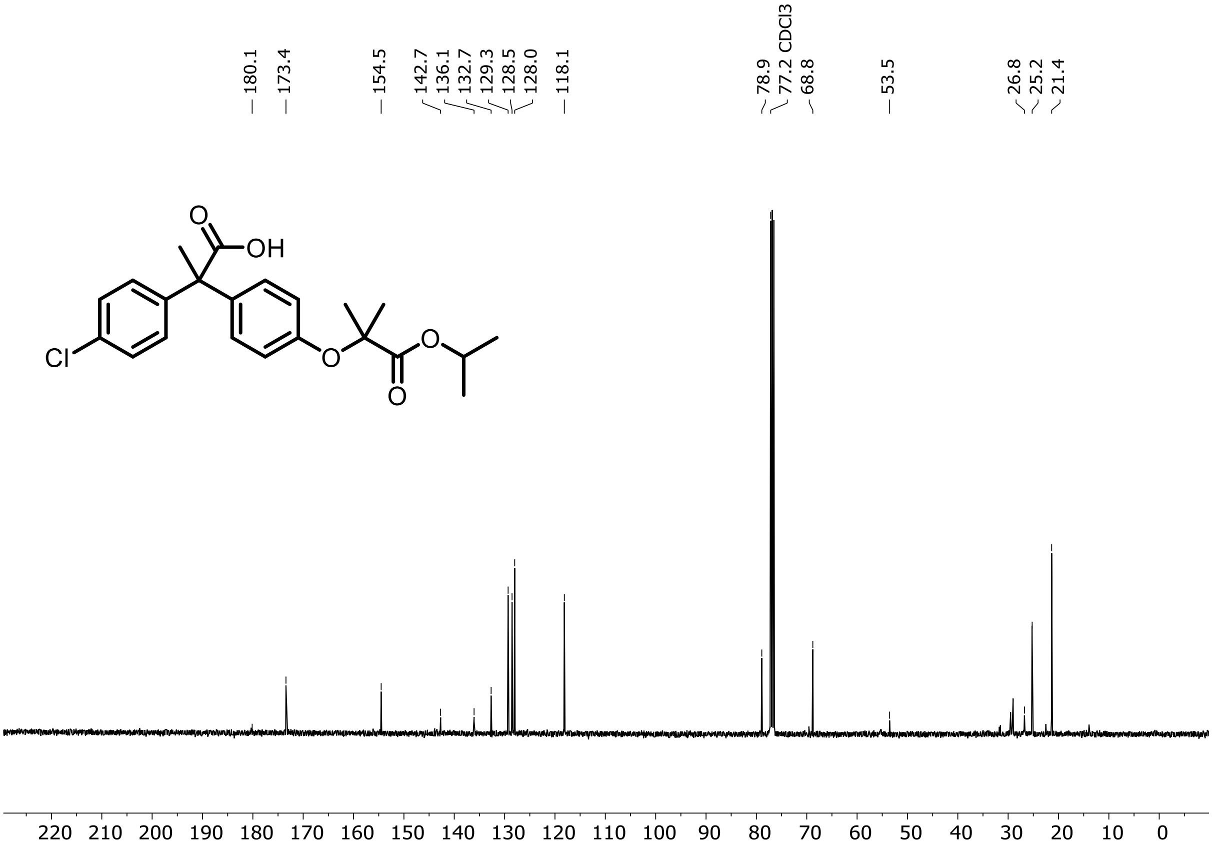

2-(3-(1-ethoxy-1-oxopropan-2-yl)phenyl)-2-phenylpropanoic acid (2af)

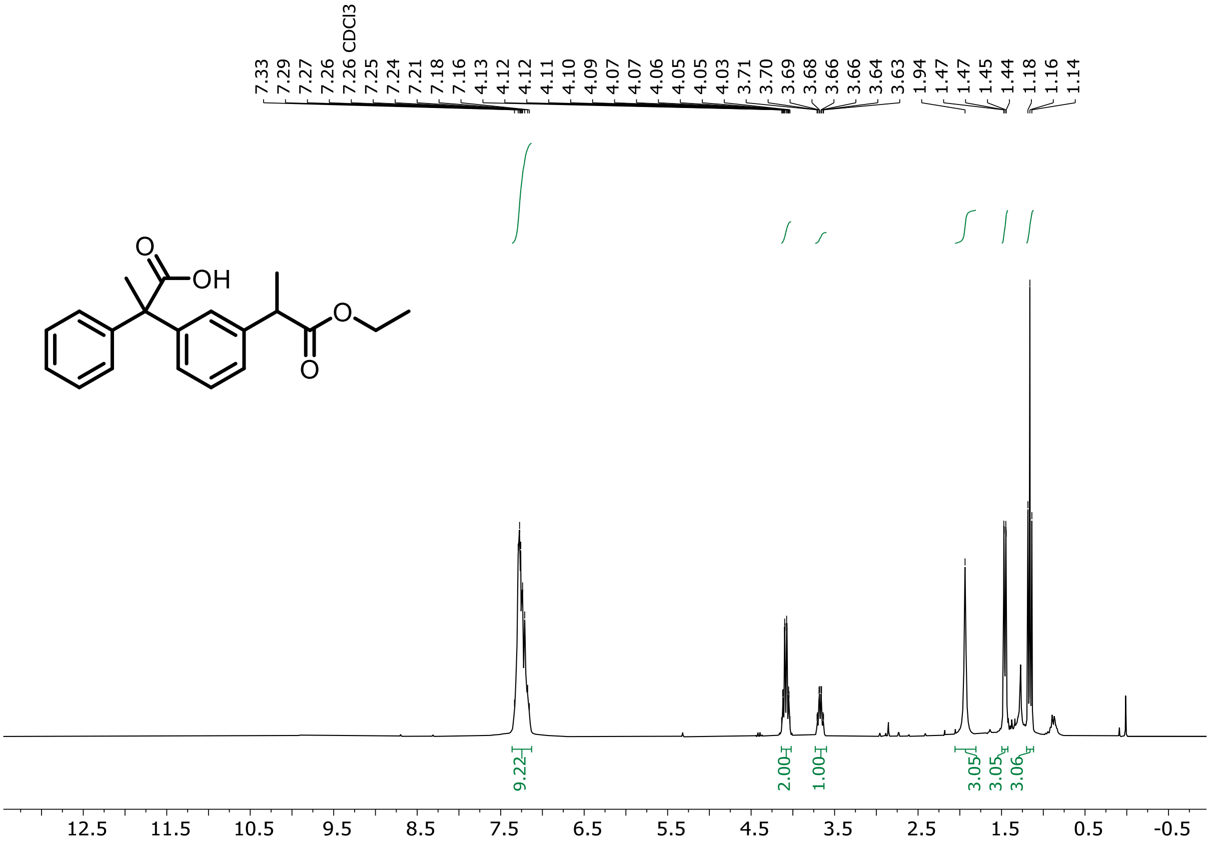

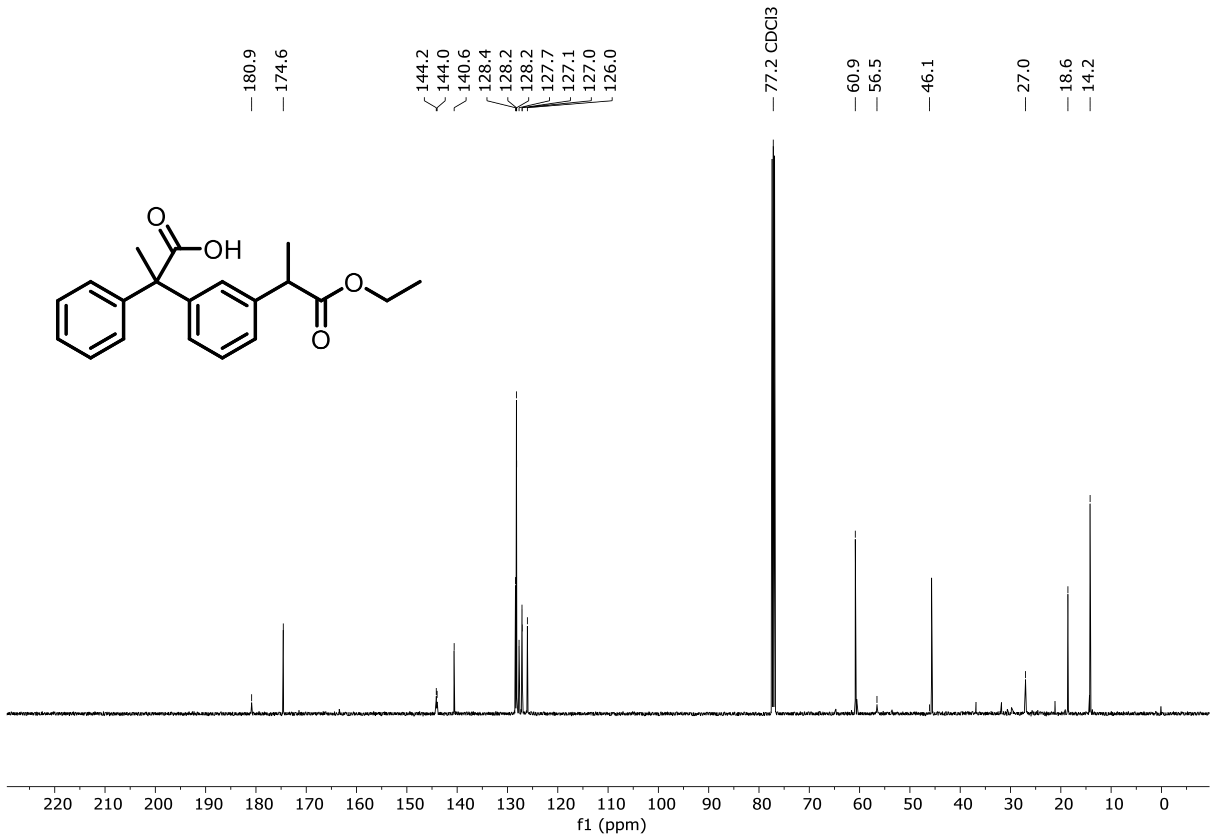

2-(4-((((2aS,5'R,6aS,6bS,8aS,8bR,9S,10R,11aS,12aS,12bR)-5',6a,8a,9-tetramethyldocosahydrospiro[naphtho[2',1':4,5]indeno[2,1-b]furan-10,2'-pyran]-4-yl)oxy)carbonyl)phenyl)propanoic acid (2ag)

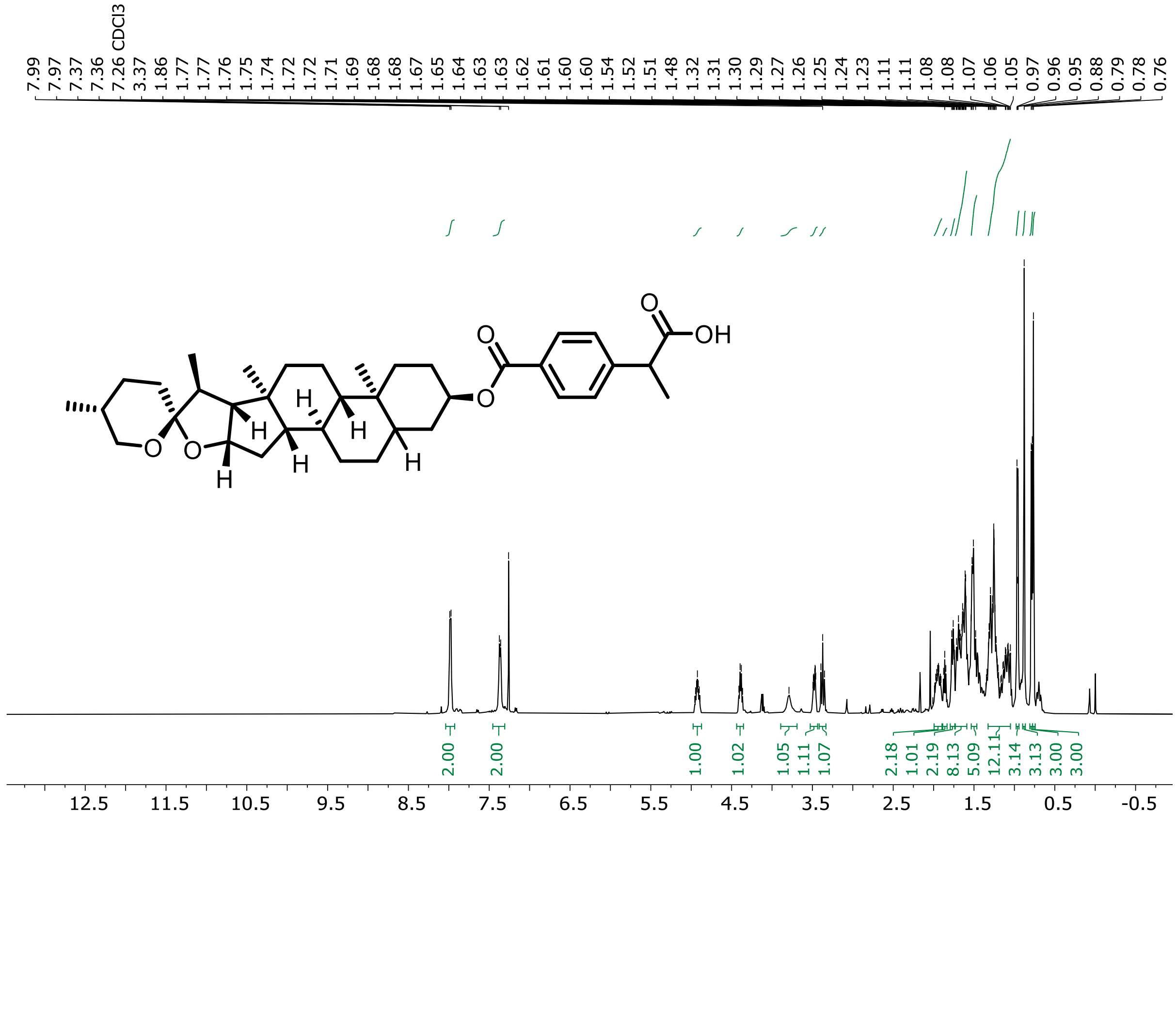

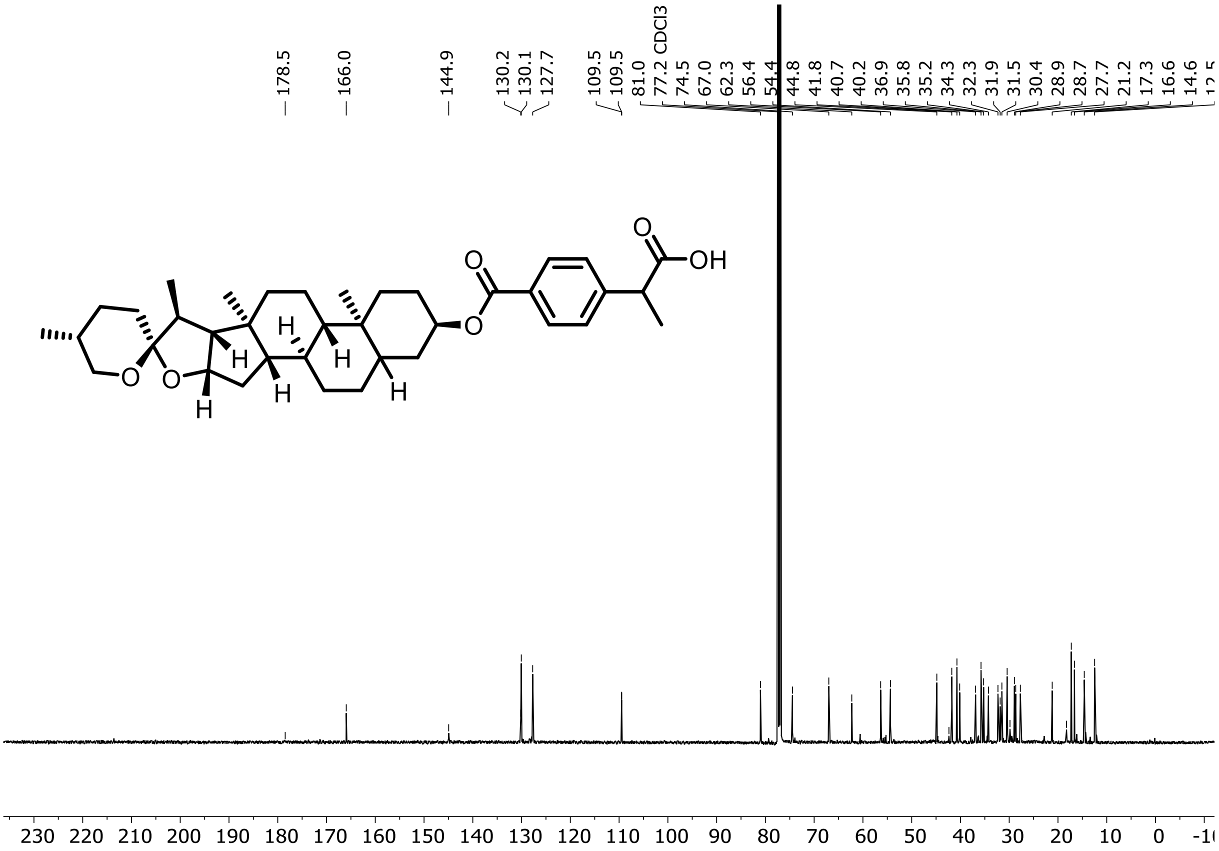

2-(4-((((8R,9S,13S,14S)-13-methyl-17-oxo-7,8,9,11,12,13,14,15,16,17-decahydro-6H-cyclopenta[a]phenanthren-3-yl)oxy)carbonyl)phenyl)propanoic acid (2ah)

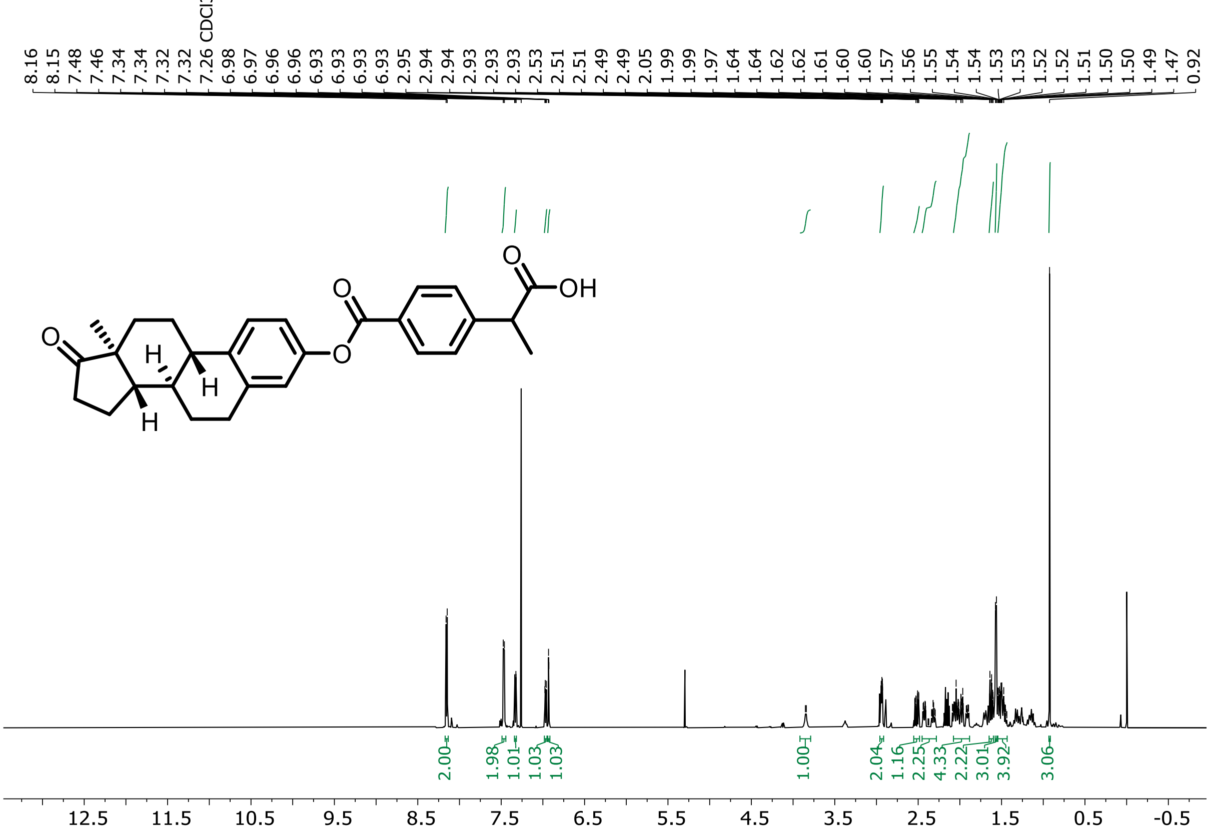

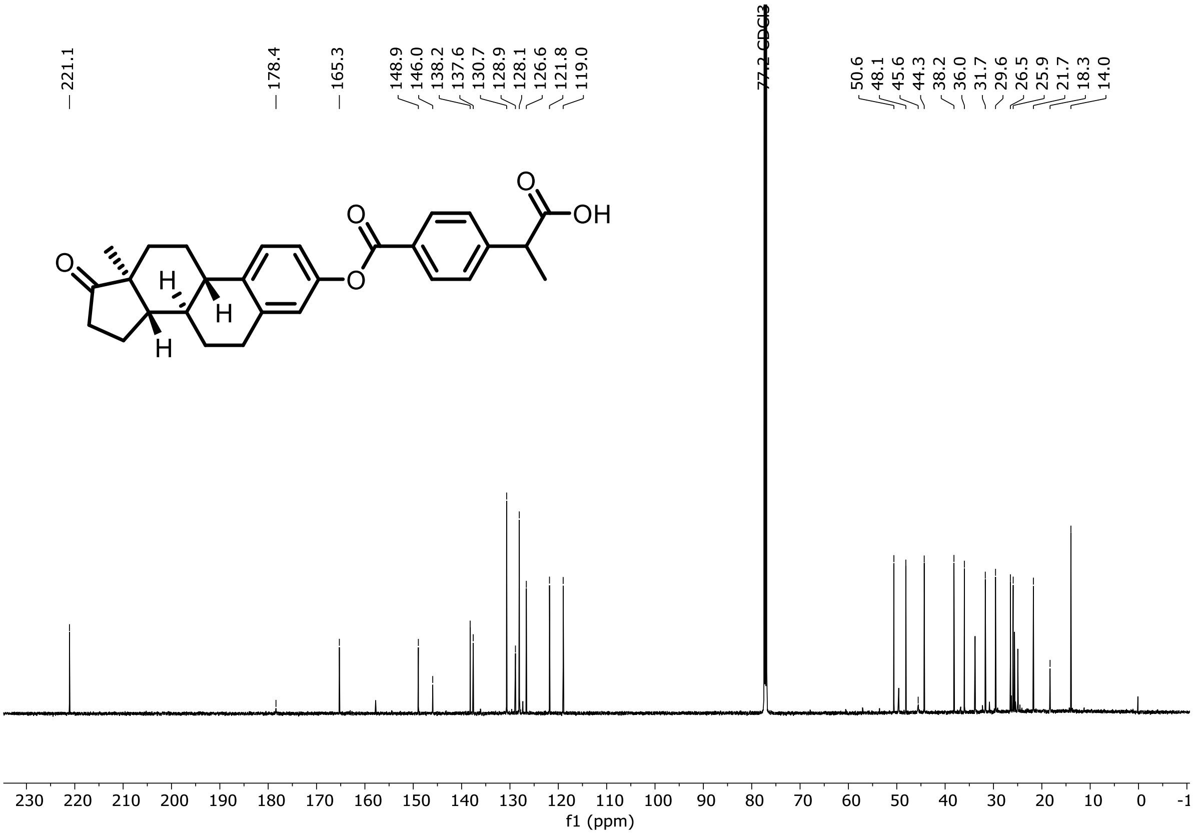

2-(4-((2-(1H-indol-3-yl)ethyl)carbamoyl)phenyl)propanoic acid (2ai)

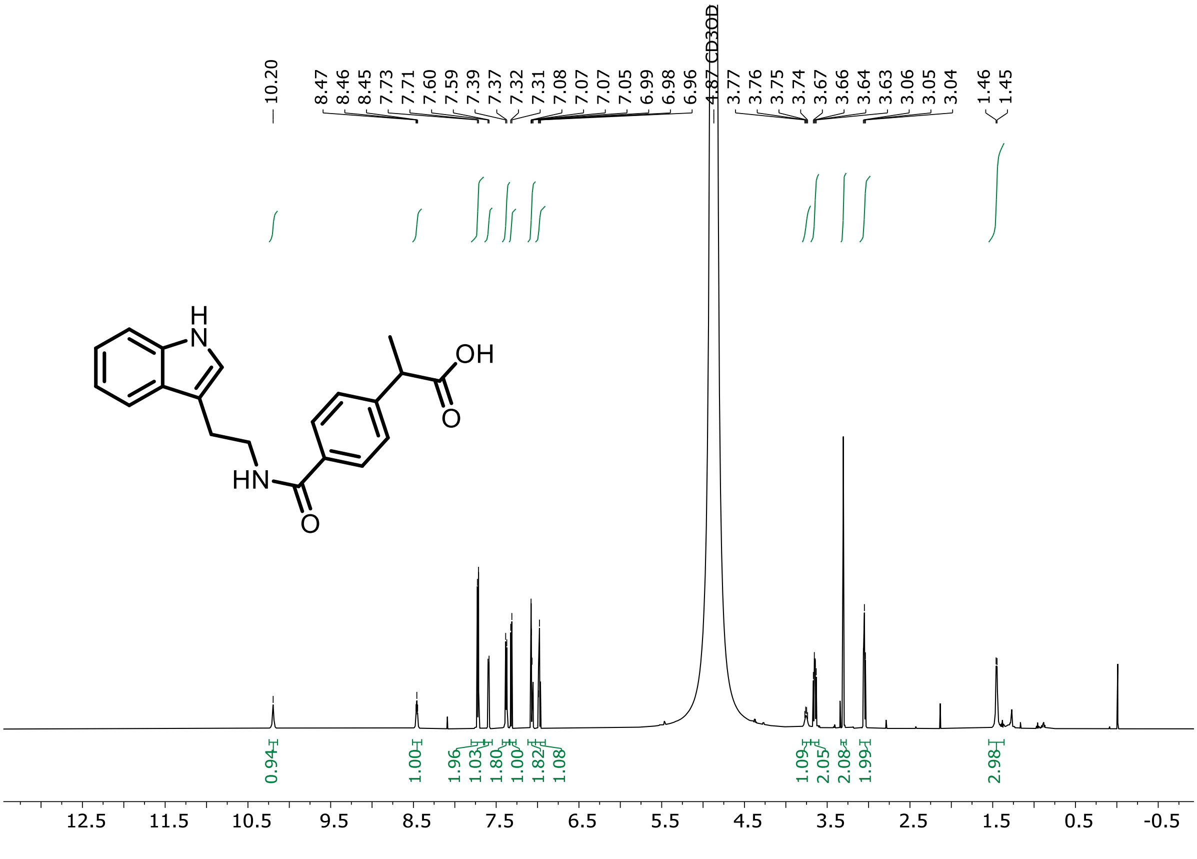

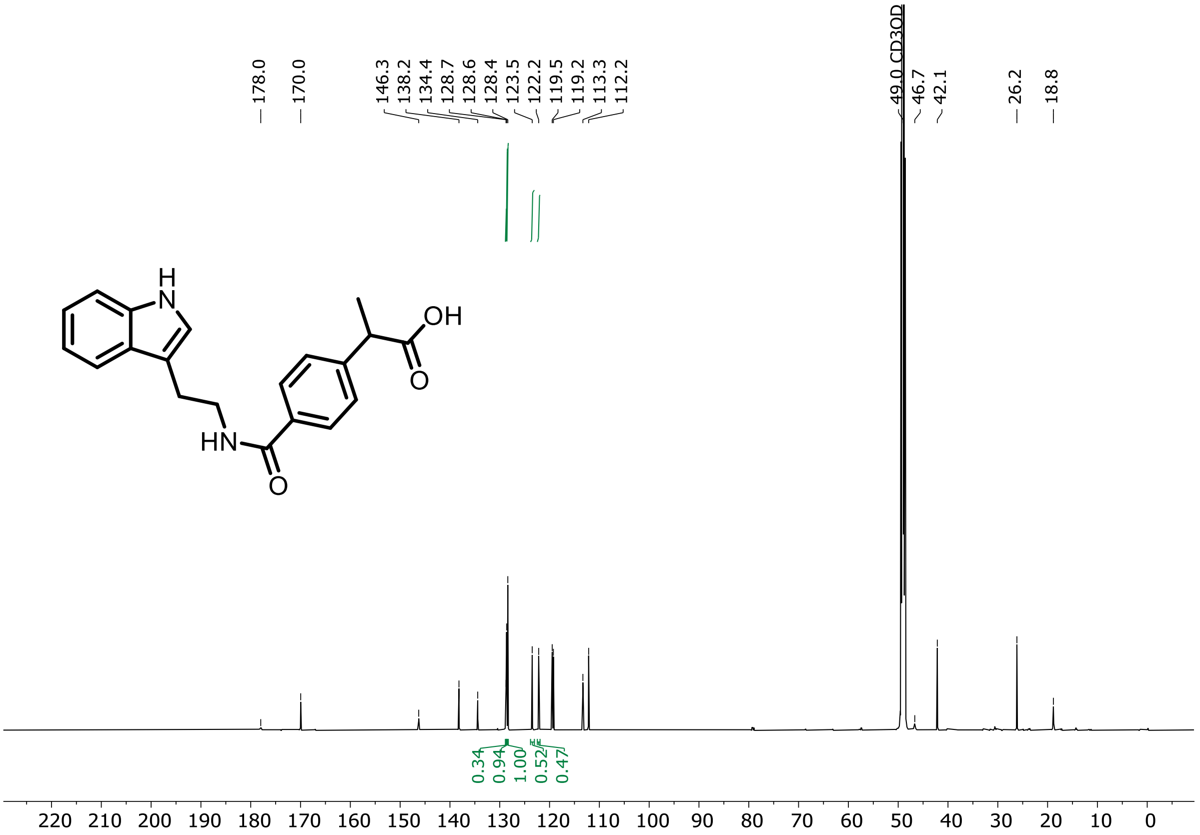

2-(4-((((1S,4aR,10aS)-7-isopropyl-1,4a-dimethyl-1,2,3,4,4a,9,10,10a-octahydrophenanthren-1-yl)methyl)carbamoyl)phenyl)propanoic acid (2aj)

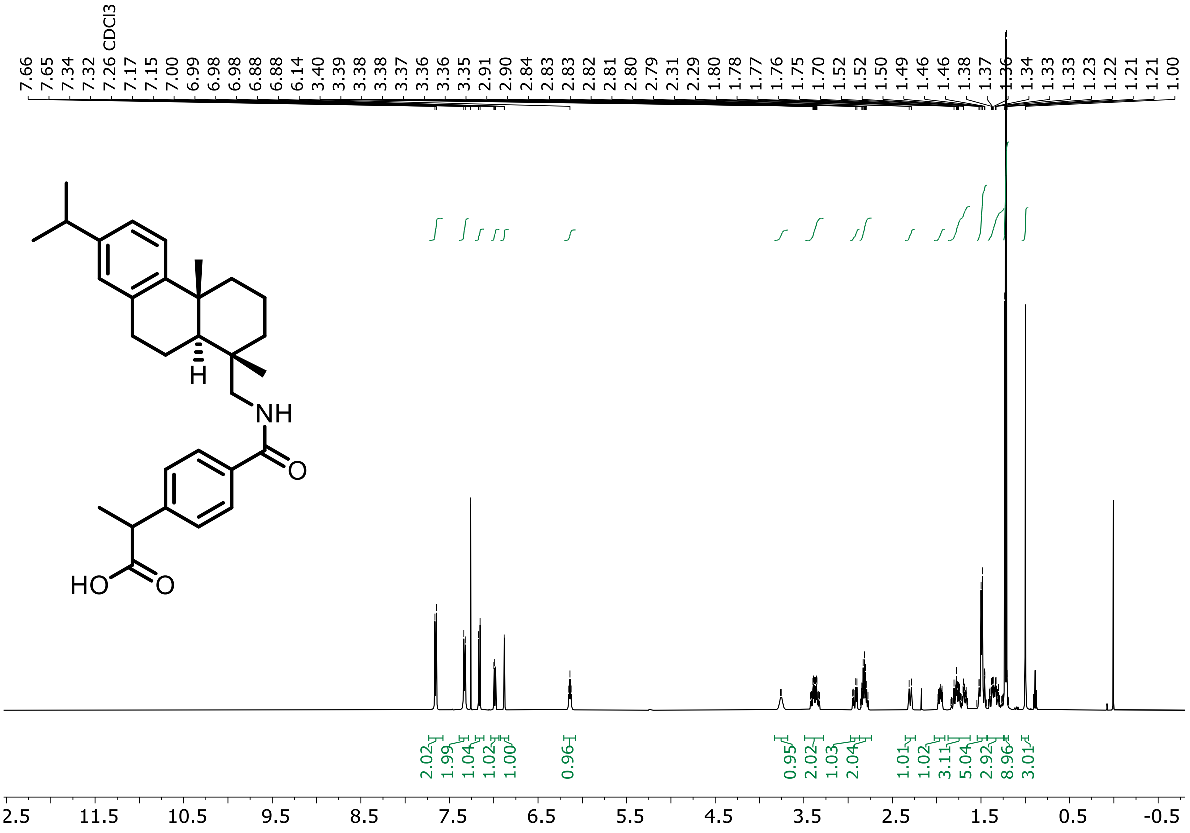

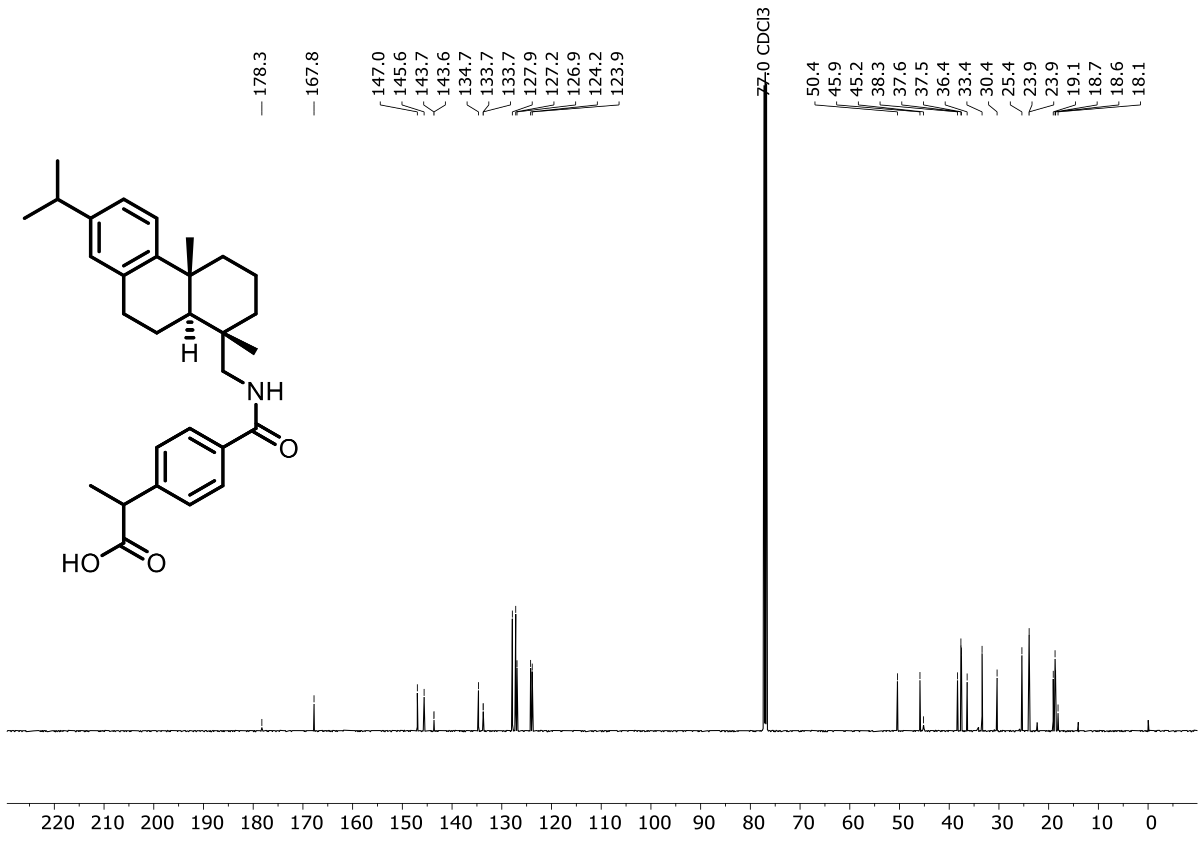
178.3
167.8
147.0
145.6
143.7
143.6
134.7
133.7
133.7
127.9
127.2
126.9
124.2
123.9
77.0 CDCl3
50.4
45.9
45.2
38.3
37.6
37.5
36.4
33.4
30.4
25.4
23.9
23.9
19.1
18.7
18.6
18.1
NH
O
HO
O
H
220 210 200 190 180 170 160 150 140 130 120 110 100 90 80 70 60 50 40 30 20 10 0

Photosubstituted photocatalyst experiments

2,3,4,6-tetra(9H-carbazol-9-yl)-5-methylbenzonitrile (4CzMeBN) (2ao)

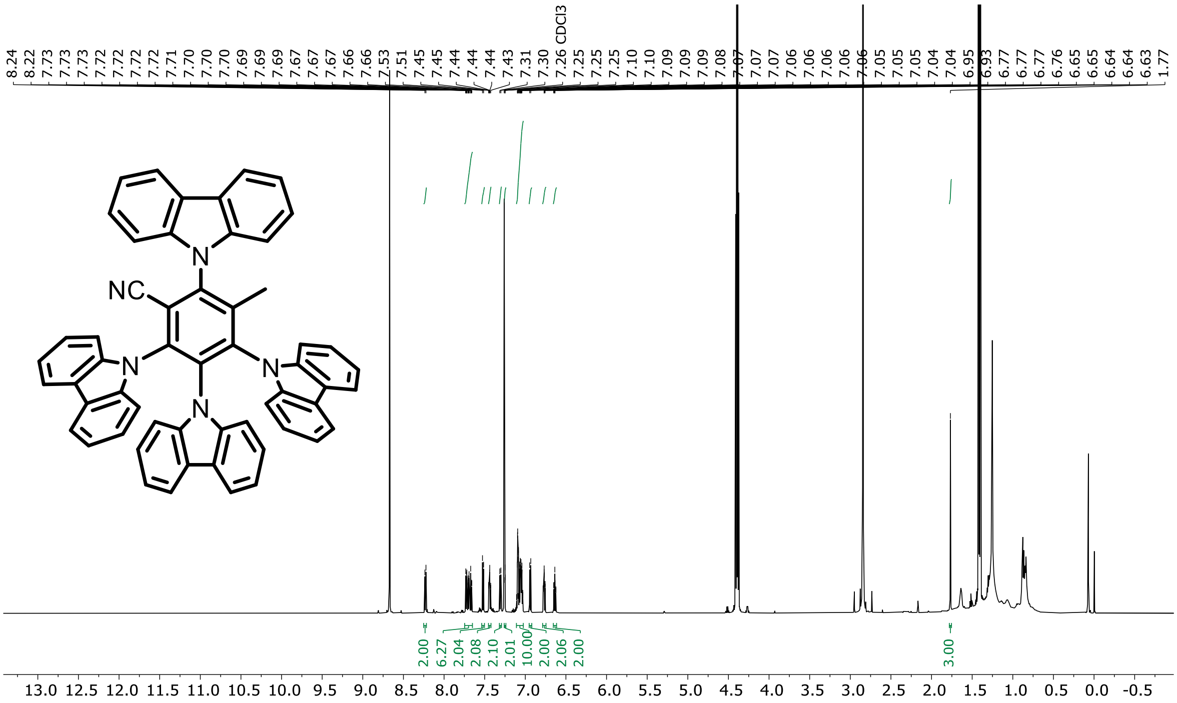

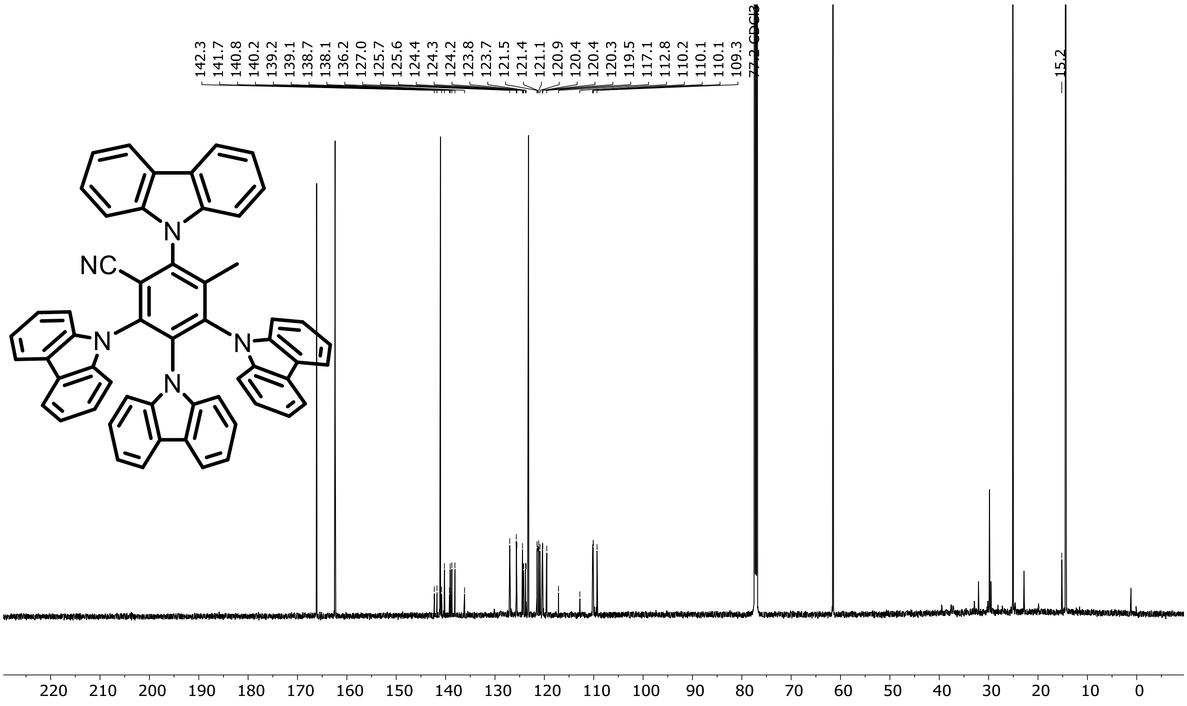

Oxidized-Hantzsch ester (HEH) and 2,3,4,6-tetra(9*H*-carbazol-9-yl)-5-methylbenzonitrile 4CzMeBN (7.7 : 1)

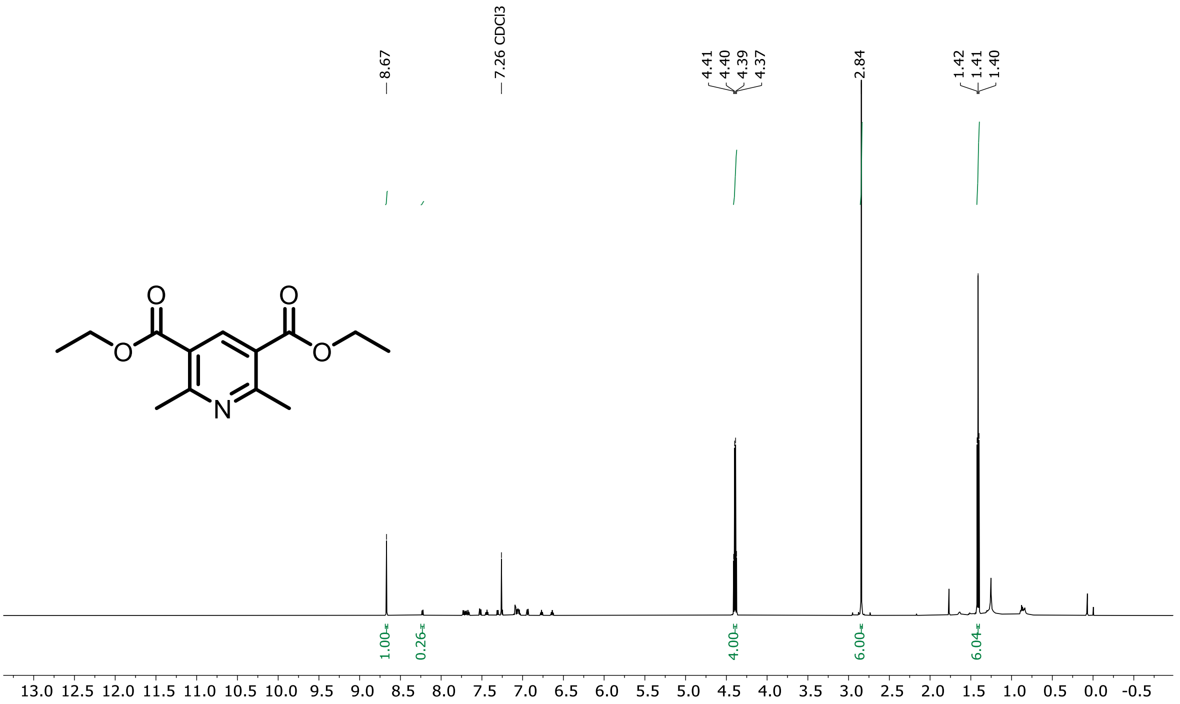

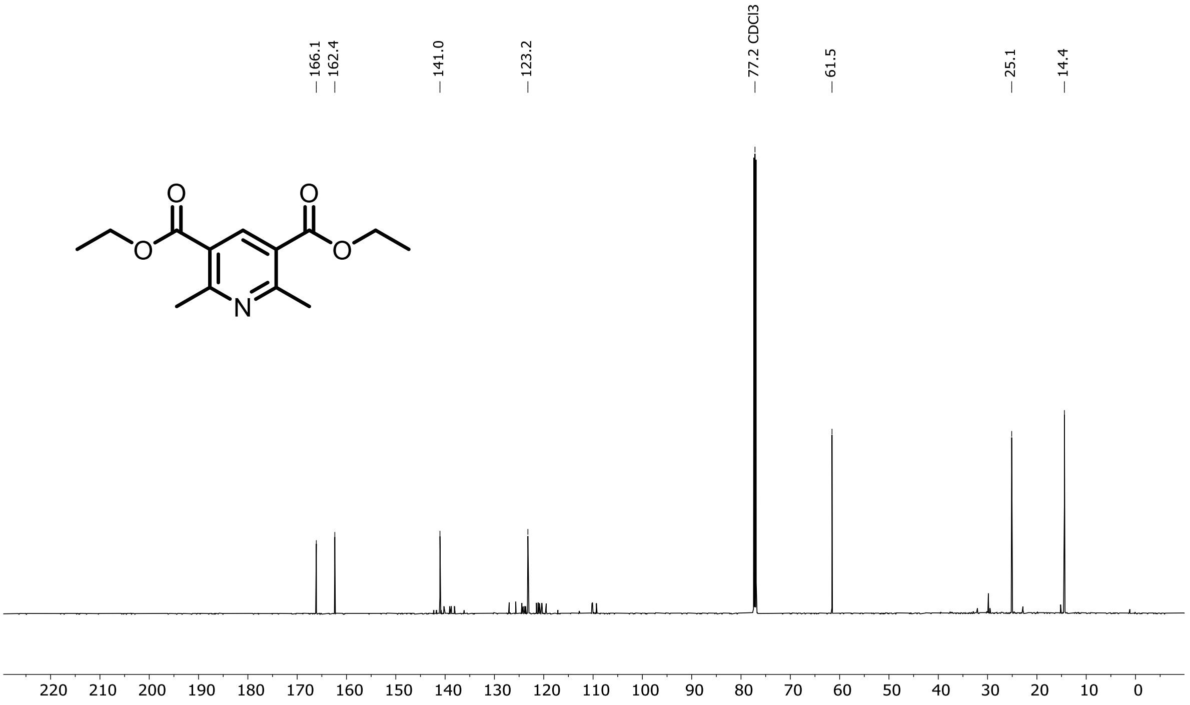

Reaction for the synthesis of 4CzMe(d_3)BN with DMF-d_7

No deuteration was observed.

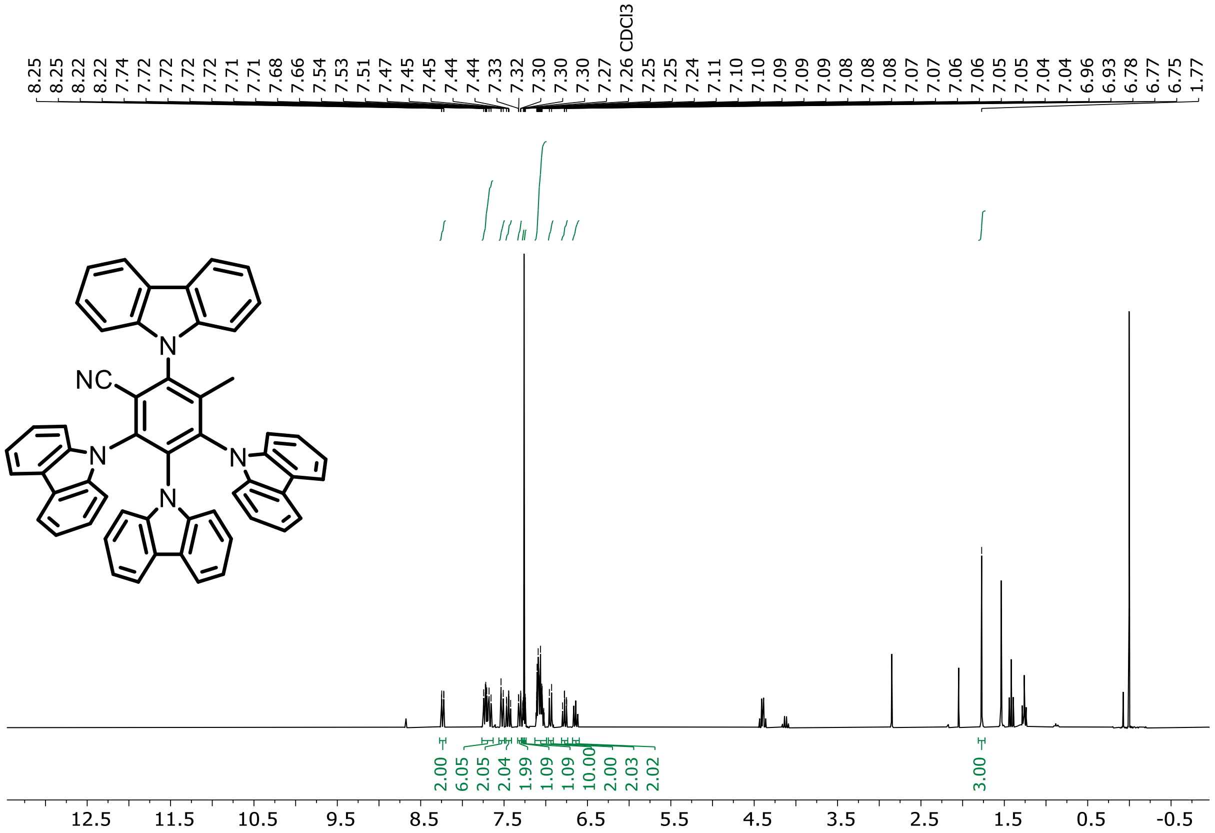

Reaction for the synthesis of 4CzMe(d_3)BN with *C4-D*-HE*D* and DMF-d_7 (2ao-d_3)

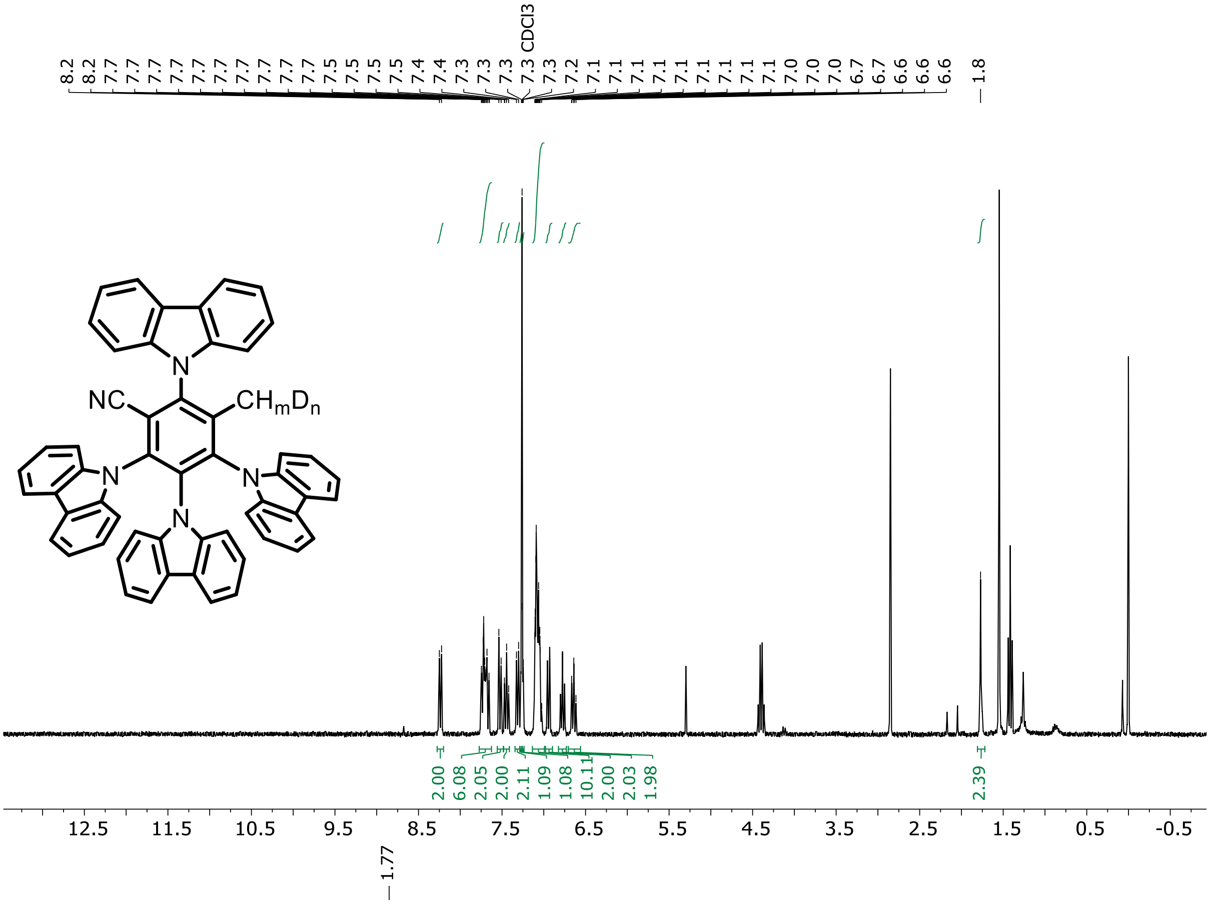

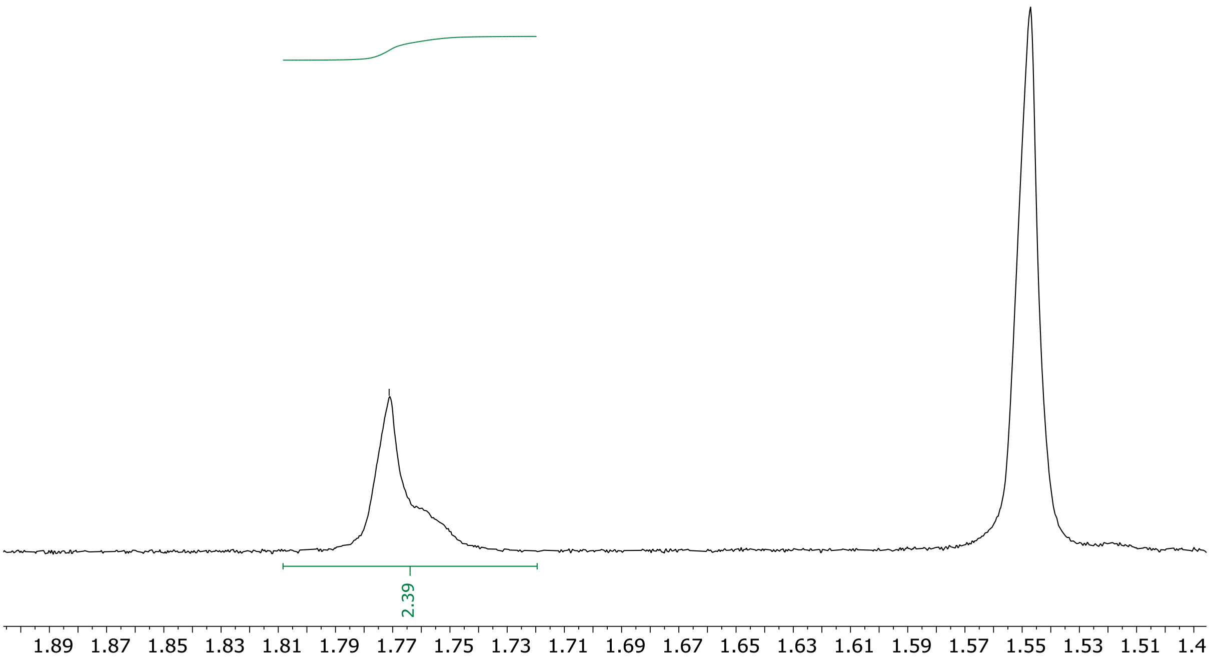

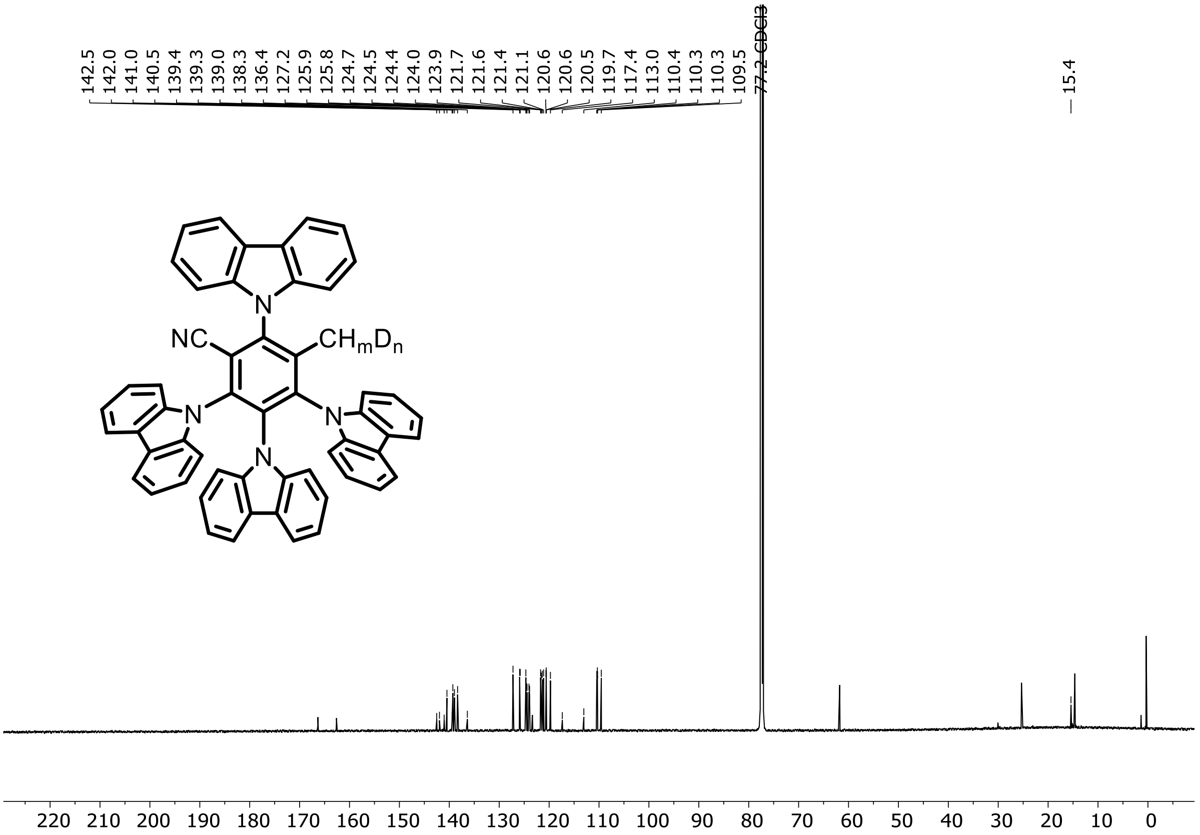

Radical ring opening

2-(4-(trifluoromethyl)phenyl)hex-4-enoic acid (2an)

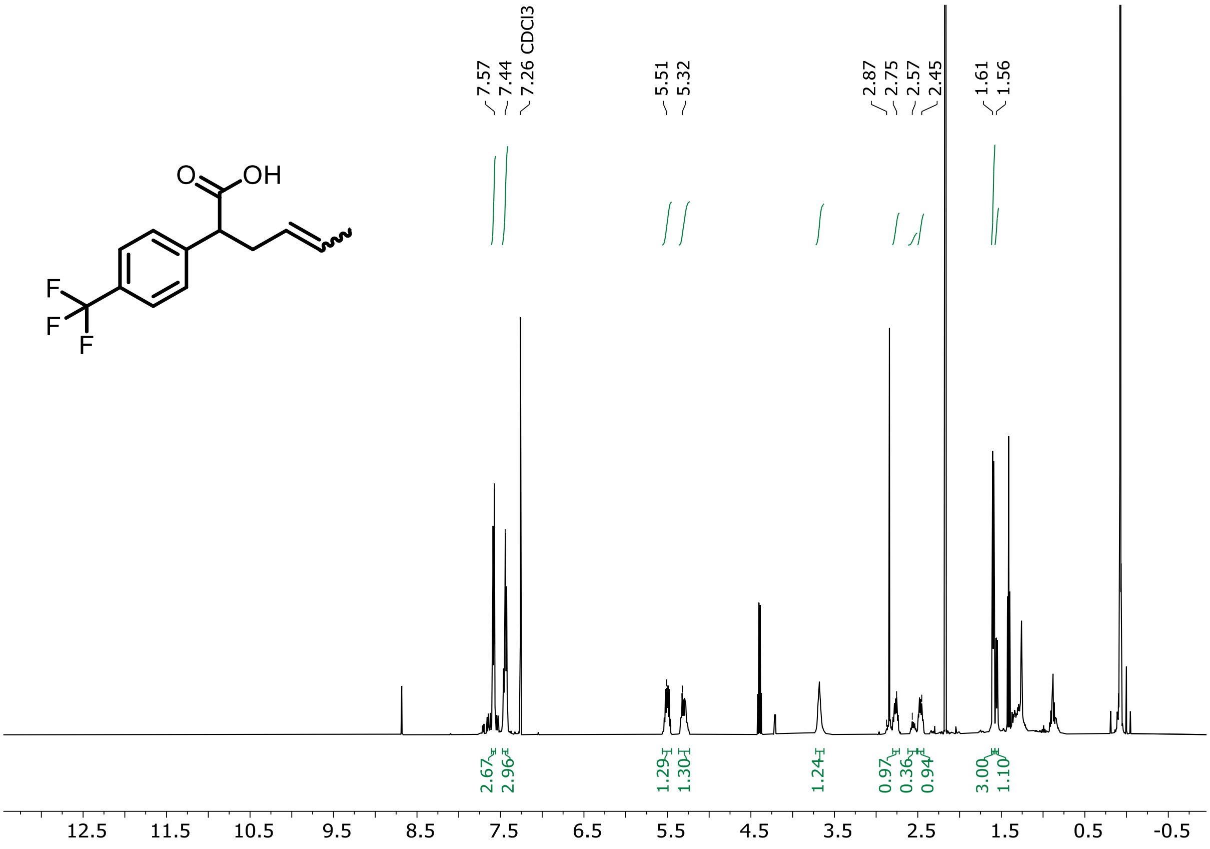

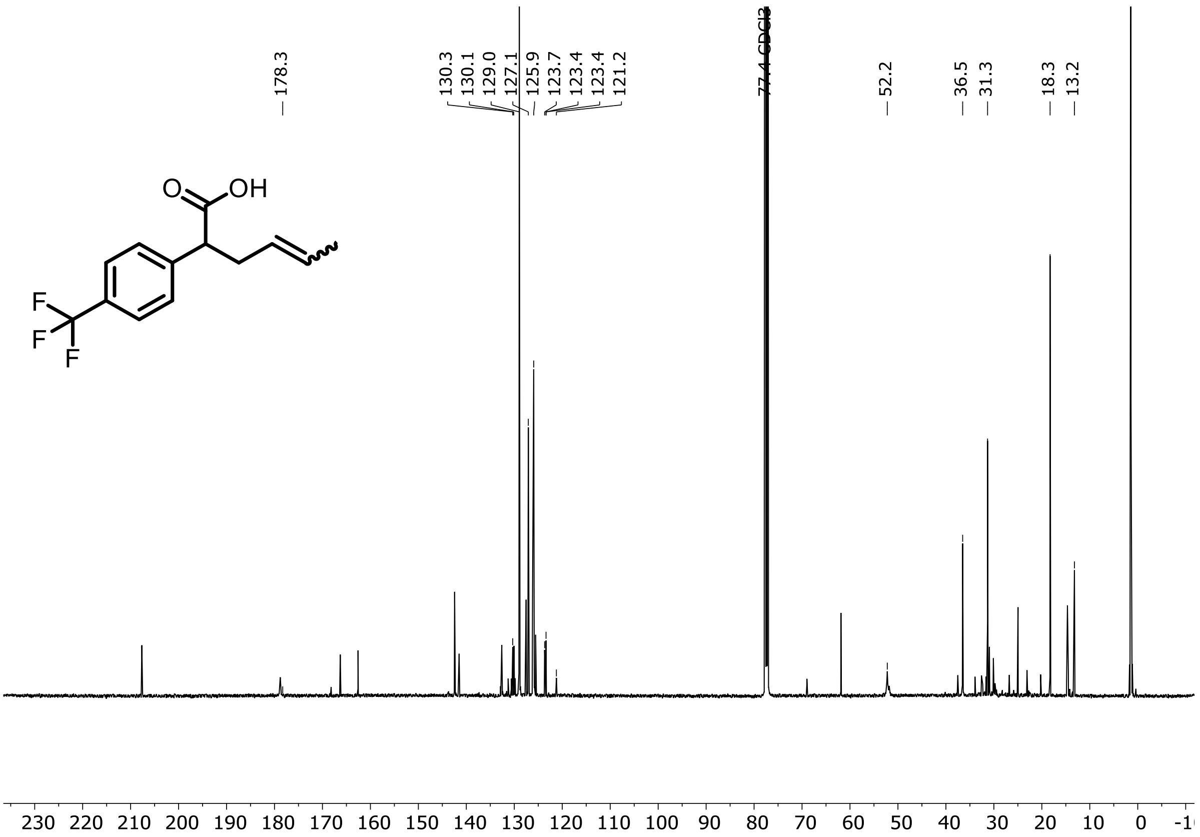
178.3
130.3
130.1
129.0
127.1
125.9
123.7
123.4
123.4
121.2
77.4 CDCl3
52.2
36.5
31.3
18.3
13.2
O
OH
F
F
F
230 220 210 200 190 180 170 160 150 140 130 120 110 100 90 80 70 60 50 40 30 20 10 0

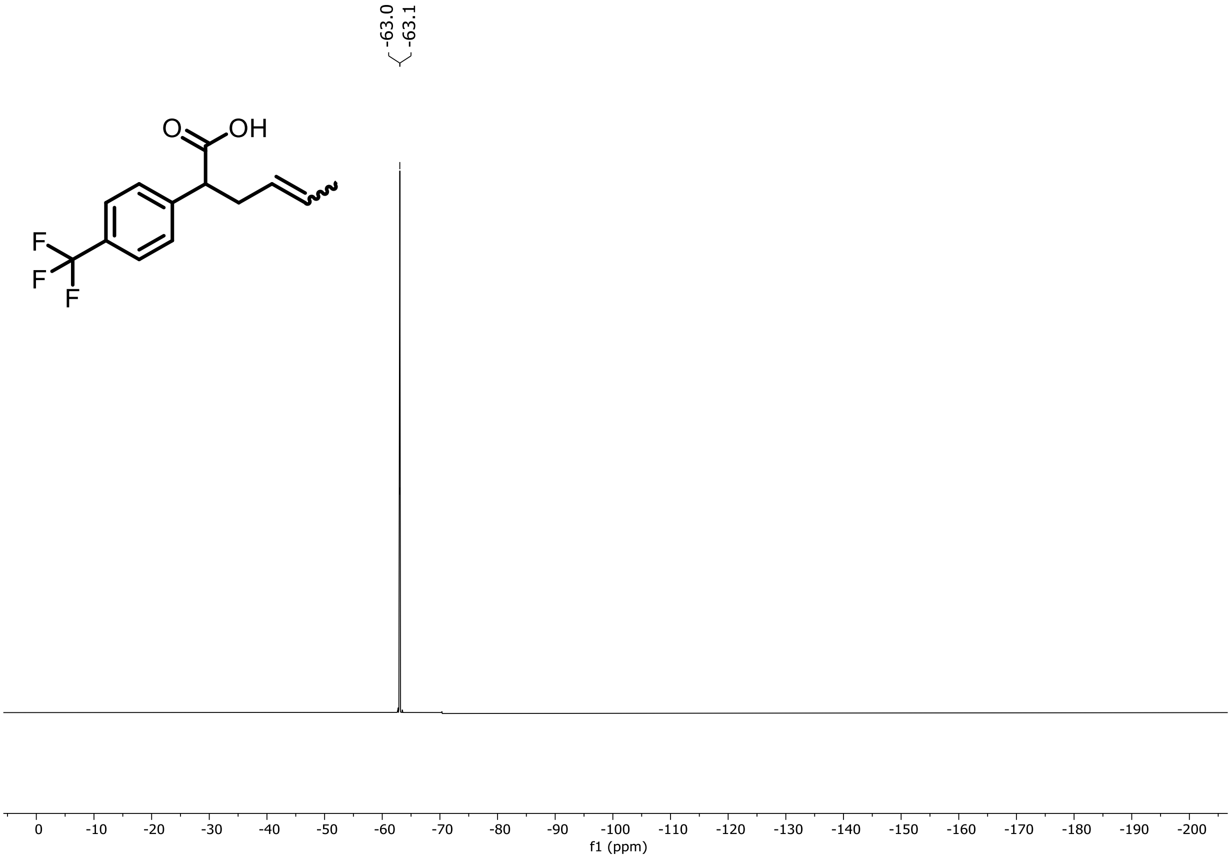
-63.0
-63.1
O
OH
F
F
F
0 -10 -20 -30 -40 -50 -60 -70 -80 -90 -100 -110 -120 -130 -140 -150 -160 -170 -180 -190 -200
f1 (ppm)

Tethered 1,1-disubstituted olefin

4-((2-methylallyl)oxy)-2-(4-(trifluoromethyl)phenyl)butanoic acid (2ar)

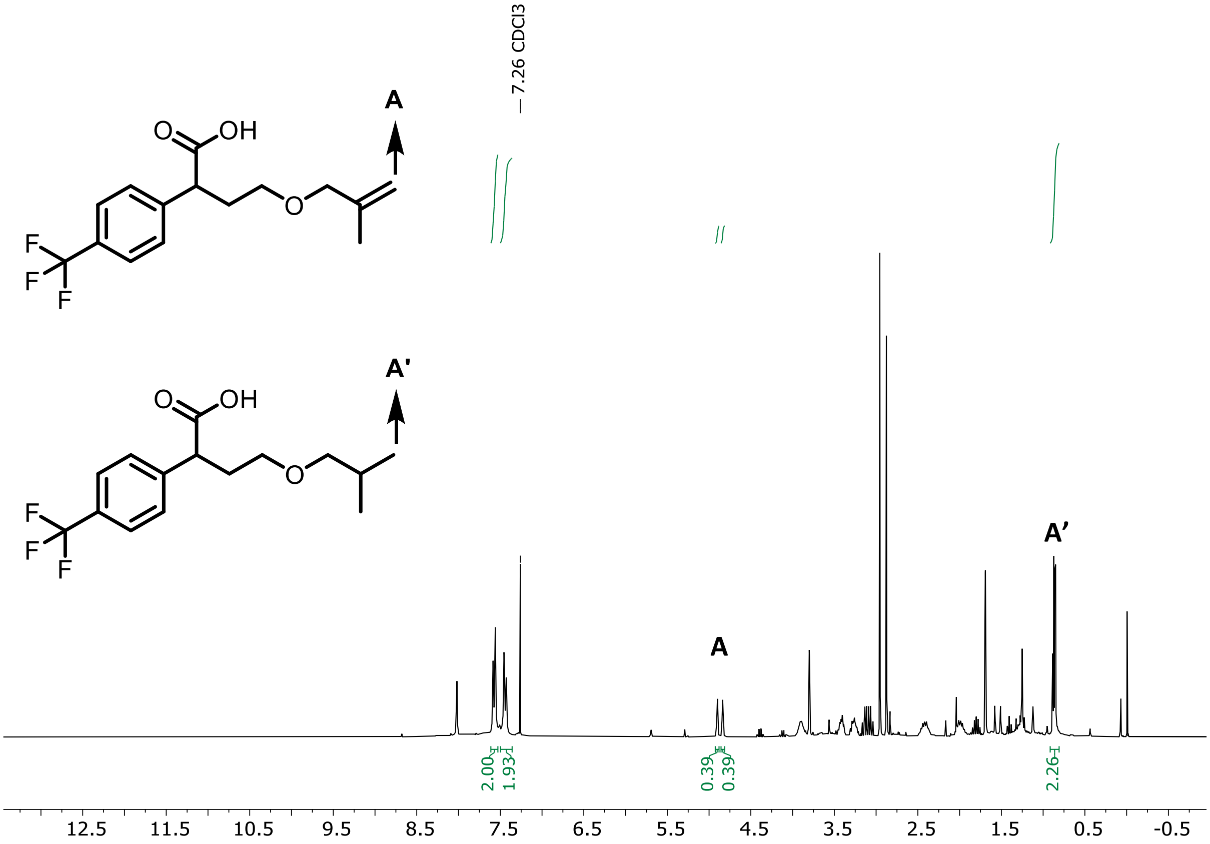

7. References

(1) Lunic, D.; Vystavkin, N.; Qin, J.; Teskey, C. J. Dual-Catalytic Structural Isomerisation as a Route to α-Arylated Ketones. *Angew. Chem. Int. Ed. 63*, e202409388. https://doi.org/10.1002/anie.202409388.

(2) Qin, J.; Barday, M.; Jana, S.; Sanosa, N.; Funes-Ardoiz, I.; Teskey, C. J. Photoinduced Cobalt Catalysis for the Reductive Coupling of Pyridines and Dienes Enabled by Paired Single-Electron Transfer**. *Angew. Chem. Int. Ed.* **2023**, *62* (45), e202310639. https://doi.org/10.1002/anie.202310639.

(3) Scheidt, F.; Neufeld, J.; Schäfer, M.; Thiehoff, C.; Gilmour, R. Catalytic *Geminal* Difluorination of Styrenes for the Construction of Fluorine-Rich Bioisosteres. *Org. Lett.* **2018**, *20* (24), 8073–8076. https://doi.org/10.1021/acs.orglett.8b03794.

(4) Molloy, J. J.; Seath, C. P.; West, M. J.; McLaughlin, C.; Fazakerley, N. J.; Kennedy, A. R.; Nelson, D. J.; Watson, A. J. B. Interrogating Pd(II) Anion Metathesis Using a Bifunctional Chemical Probe: A Transmetalation Switch. *J. Am. Chem. Soc.* **2018**, *140* (1), 126–130. https://doi.org/10.1021/jacs.7b11180.

(5) Littke, A. F.; Schwarz, L.; Fu, G. C. Pd/P(*t* -Bu)$_3$: A Mild and General Catalyst for Stille Reactions of Aryl Chlorides and Aryl Bromides. *J. Am. Chem. Soc.* **2002**, *124* (22), 6343–6348. https://doi.org/10.1021/ja020012f.

(6) Ott, I.; Schmidt, K.; Kircher, B.; Schumacher, P.; Wiglenda, T.; Gust, R. Antitumor-Active Cobalt−Alkyne Complexes Derived from Acetylsalicylic Acid: Studies on the Mode of Drug Action. *J. Med. Chem.* **2005**, *48* (2), 622–629. https://doi.org/10.1021/jm049326z.

(7) Hutchins, K. M.; Sekerak, N. M.; Moore, J. S. Polymerization Initiated by Particle Contact: A Quiescent State Trigger for Materials Synthesis. *J. Am. Chem. Soc.* **2016**, *138* (38), 12336–12339. https://doi.org/10.1021/jacs.6b07742.

(8) Rong, G.; Liu, D.; Lu, L.; Yan, H.; Zheng, Y.; Chen, J.; Mao, J. Iron-Catalyzed Decarboxylative Methylation of α,β-Unsaturated Acids under Ligand-Free Conditions. *Tetrahedron* **2014**, *70* (34), 5033–5037. https://doi.org/10.1016/j.tet.2014.06.014.

(9) Shi, M.; Wang, B.-Y.; Huang, J.-W. Palladium-Catalyzed Isomerization of Methylenecyclopropanes in Acetic Acid. *J. Org. Chem.* **2005**, *70* (14), 5606–5610. https://doi.org/10.1021/jo050560m.

(10) Shin, J. H.; Seong, E. Y.; Mun, H. J.; Jang, Y. J.; Kang, E. J. Electronically Mismatched Cycloaddition Reactions via First-Row Transition Metal, Iron(III)–Polypyridyl Complex. *Org. Lett.* **2018**, *20* (18), 5872–5876. https://doi.org/10.1021/acs.orglett.8b02541.

(11) Nakao, Y.; Imanaka, H.; Sahoo, A. K.; Yada, A.; Hiyama, T. Alkenyl- and Aryl[2-(Hydroxymethyl)Phenyl]Dimethylsilanes: An Entry to Tetraorganosilicon Reagents for the Silicon-Based Cross-Coupling Reaction. *J. Am. Chem. Soc.* **2005**, *127* (19), 6952–6953. https://doi.org/10.1021/ja051281j.

(12) vom Stein, T.; Peréz, M.; Dobrovetsky, R.; Winkelhaus, D.; Caputo, C. B.; Stephan, D. W. Electrophilic Fluorophosphonium Cations in Frustrated Lewis Pair Hydrogen Activation and Catalytic Hydrogenation of Olefins. *Angew. Chem. Int. Ed.* **2015**, *54* (35), 10178–10182. https://doi.org/10.1002/anie.201504109.

(13) Sun, Chang-Liang et. al. Construction of Polysubstituted Olefins through Ni-Catalyzed Direct Activation of Alkenyl C-O of Substituted Alkenyl Acetates. *Chem. – Eur. J.* **2010**, *16* (20), 5844–5847. https://doi.org/10.1002/chem.200902785.

(14) Namai, H.; Ikeda, H.; Kato, N.; Mizuno, K. Substituent Effects on the Energies of the Electronic Transitions of Geminally Diphenyl-Substituted Trimethylenemethane (TMM) Radical Cations. Experimental and Theoretical Evidence for a Twisted Molecular and Localized Electronic Structure. *J. Phys. Chem. A* **2007**, *111* (20), 4436–4442. https://doi.org/10.1021/jp068308l.

(15) Zhao, X.; Jing, J.; Lu, K.; Zhang, Y.; Wang, J. Pd-Catalyzed Oxidative Cross-Coupling of N-Tosylhydrazones with Arylboronic Acids. *Chem. Commun.* **2010**, *46* (10), 1724. https://doi.org/10.1039/b925590g.

(16) Noto, N.; Koike, T.; Akita, M. Visible-Light-Triggered Monofluoromethylation of Alkenes by Strongly Reducing 1,4-Bis(Diphenylamino)Naphthalene Photoredox Catalysis. *ACS Catal.* **2019**, *9* (5), 4382–4387. https://doi.org/10.1021/acscatal.9b00473.
(17) Wang, Z.; Huang, Y.; Guo, J.; Li, Z.; Xu, J.; Lu, J. Q.; Wang, C. Design and Synthesis of Thermal Contracting Polymer with Unique Eight-Membered Carbocycle Unit. *Macromolecules* **2018**, *51* (4), 1377–1385. https://doi.org/10.1021/acs.macromol.7b02705.
(18) Reddy, R. S.; Kiran, I. N. C.; Sudalai, A. CN-Assisted Oxidative Cyclization of Cyano Cinnamates and Styrene Derivatives: A Facile Entry to 3-Substituted Chiral Phthalides. *Org. Biomol. Chem.* **2012**, *10* (18), 3655. https://doi.org/10.1039/c2ob25409c.
(19) Ojha, D. P.; Prabhu, K. R. Palladium Catalyzed Coupling of Tosylhydrazones with Aryl and Heteroaryl Halides in the Absence of External Ligands: Synthesis of Substituted Olefins. *J. Org. Chem.* **2012**, *77* (24), 11027–11033. https://doi.org/10.1021/jo301987c.
(20) Chatupheeraphat, A.; Rueping, M.; Magre, M. Chemo- and Regioselective Magnesium-Catalyzed *Ortho* -Alkenylation of Anilines. *Org. Lett.* **2019**, *21* (22), 9153–9157. https://doi.org/10.1021/acs.orglett.9b03526.
(21) Mameda, N.; Peraka, S.; Kodumuri, S.; Chevella, D.; Marri, M. R.; Nama, N. Ortho-Alkenylation of Anilines with Aromatic Terminal Alkynes over Nanosized Zeolite Beta. *RSC Adv.* **2015**, *5* (95), 78374–78378. https://doi.org/10.1039/C5RA16931C.
(22) Yin, X.; Chen, B.; Qiu, F.; Wang, X.; Liao, Y.; Wang, M.; Lei, X.; Liao, J. Enantioselective Palladium-Catalyzed Hydrofluorination of Alkenylarenes. *ACS Catal.* **2020**, *10* (3), 1954–1960. https://doi.org/10.1021/acscatal.9b05264.
(23) Xu, J.; Liu, B. Metal Free Functionalization of Saturated Heterocycles with Vinylarenes and Pyridine Enabled by Photocatalytic Hydrogen Atom Transfer. *Chem. – Eur. J.* **2024**, *30* (31), e202400612. https://doi.org/10.1002/chem.202400612.
(24) Zeng, Y.; Zheng, X.; Shen, L.; Jing, Y.; Chen, S.; Luo, Z.; Ke, Z.; Xie, H.; Liu, J.; Jiang, H.; Zeng, W. Oxydiazomethylation of Alkenes *via* Photoredox Catalysis. *Chem. – Eur. J.* **2025**, *31* (4), e202403509. https://doi.org/10.1002/chem.202403509.
(25) Oddy et. al. Visible-Light-Mediated Energy Transfer Enables the Synthesis of β-Lactams via Intramolecular Hydrogen Atom Transfer. *Angew. Chem. Int. Ed.* *61* (48). https://doi.org/doi.org/10.1002/anie.202213086.
(26) Mendel, M.; Karl, T. M.; Hamm, J.; Kaldas, S. J.; Sperger, T.; Mondal, B.; Schoenebeck, F. Dynamic Stereomutation of Vinylcyclopropanes with Metalloradicals. *Nature* **2024**, *631* (8019), 80–86. https://doi.org/10.1038/s41586-024-07555-1.
(27) Shunsuke Einaru; Kenta Shitamichi,; Tagui Nagano; Akira Matsumoto; Keisuke Asano; Seijiro Matsubara. Trans-Cyclooctenes as Halolactonization Catalysts. *Angew. Chem. Int. Ed.* **2018**, *57* (42), 13863–13867. https://doi.org/10.1002/anie.201808320.
(28) Okamoto, R.; Tanaka, K. Rhodium-Catalyzed Olefin Isomerization/Allyl Claisen Rearrangement/Intramolecular Hydroacylation Cascade. *Org. Lett.* **2013**, *15* (9), 2112–2115. https://doi.org/10.1021/ol400574s.
(29) Meng, Q.-Y.; Wang, S.; Huff, G. S.; König, B. Ligand-Controlled Regioselective Hydrocarboxylation of Styrenes with CO_2 by Combining Visible Light and Nickel Catalysis. *J. Am. Chem. Soc.* **2018**, *140* (9), 3198–3201. https://doi.org/10.1021/jacs.7b13448.
(30) Ran, C.-K.; Niu, Y.-N.; Song, L.; Wei, M.-K.; Cao, Y.-F.; Luo, S.-P.; Yu, Y.-M.; Liao, L.-L.; Yu, D.-G. Visible-Light Photoredox-Catalyzed Carboxylation of Activated C(Sp^3)–O Bonds with CO_2. *ACS Catal.* **2022**, *12* (1), 18–24. https://doi.org/10.1021/acscatal.1c04921.
(31) Isamu Shiina. Kinetic Resolution of Racemic α-Arylalkanoic Acids with Achiral Alcohols via the Asymmetric Esterification Using Carboxylic Anhydrides and Acyl-Transfer Catalysts. *J. Am. Chem. Soc.* **2010**, *132* (33), 11629–11641. https://doi.org/doi.org/10.1021/ja103490h.
(32) Ren, W.; Sheng, X.; Fan, C.; Shi, Y. Pd-Catalyzed Regiodivergent Hydrocarboxylation of Olefins with Oxalic Acid: A Remarkable Effect of the Counteranion on Regioselectivity. *Org. Lett.* **2023**, *25* (43), 7786–7790. https://doi.org/10.1021/acs.orglett.3c02805.

(33) Fuchikami, T.; Ohishi, K.; Ojima, I. Regioselective Hydroesterification and Hydrocarboxylation of 3,3,3-Trifluoropropene and Pentafluorostyrene Catalyzed by Phosphine-Palladium Complex. *J. Org. Chem.* **1983**, *48* (21), 3803–3807. https://doi.org/10.1021/jo00169a040.

(34) Melvin, P. R.; Ferguson, D. M.; Schimler, S. D.; Bland, D. C.; Sanford, M. S. Room Temperature Deoxyfluorination of Benzaldehydes and α-Ketoesters with Sulfuryl Fluoride and Tetramethylammonium Fluoride. *Org. Lett.* **2019**, *21* (5), 1350–1353. https://doi.org/10.1021/acs.orglett.9b00054.

(35) Gaydou, M.; Moragas, T.; Juliá-Hernández, F.; Martin, R. Site-Selective Catalytic Carboxylation of Unsaturated Hydrocarbons with CO_2 and Water. *J. Am. Chem. Soc.* **2017**, *139* (35), 12161–12164. https://doi.org/10.1021/jacs.7b07637.

(36) Liao, L.-L.; Cao, G.-M.; Ye, J.-H.; Sun, G.-Q.; Zhou, W.-J.; Gui, Y.-Y.; Yan, S.-S.; Shen, G.; Yu, D.-G. Visible-Light-Driven External-Reductant-Free Cross-Electrophile Couplings of Tetraalkyl Ammonium Salts. *J. Am. Chem. Soc.* **2018**, *140* (50), 17338–17342. https://doi.org/10.1021/jacs.8b08792.

(37) Yu, J.; Liu, T.; Sun, W.; Zhang, Y. Electrochemical Decarboxylative Elimination of Carboxylic Acids to Alkenes. *Org. Lett.* **2023**, *25* (43), 7816–7821. https://doi.org/10.1021/acs.orglett.3c02997.

(38) Wei, J.; Gandon, V.; Zhu, Y. Amino Acid-Derived Ionic Chiral Catalysts Enable Desymmetrizing Cross-Coupling to Remote Acyclic Quaternary Stereocenters. *J. Am. Chem. Soc.* **2023**, *145* (30), 16796–16811. https://doi.org/10.1021/jacs.3c04877.

(39) Liu, K.; Li, N.; Ning, Y.; Zhu, C.; Xie, J. Gold-Catalyzed Oxidative Biaryl Cross-Coupling of Organometallics. *Chem* **2019**, *5* (10), 2718–2730. https://doi.org/10.1016/j.chempr.2019.07.023.

(40) Cruz, F. A.; Dong, V. M. Stereodivergent Coupling of Aldehydes and Alkynes via Synergistic Catalysis Using Rh and Jacobsen's Amine. *J. Am. Chem. Soc.* **2017**, *139* (3), 1029–1032. https://doi.org/10.1021/jacs.6b10680.

(41) Kukosha, T.; Trufilkina, N.; Katkevics, M. Synthesis of N-Alkoxyindol-2-Ones by Copper-Catalyzed Intramolecular N-Arylation of Hydroxamates. *Synlett* **2011**, *2011* (17), 2525–2528. https://doi.org/10.1055/s-0030-1260328.

(42) Bergamaschi, E.; Mayerhofer, V. J.; Teskey, C. J. Light-Driven Cobalt Hydride Catalyzed Hydroarylation of Styrenes. *ACS Catal.* **2022**, *12* (24), 14806–14811. https://doi.org/10.1021/acscatal.2c05109.

(43) Wu, Y.; Kim, D.; Teets, T. S. Photophysical Properties and Redox Potentials of Photosensitizers for Organic Photoredox Transformations. *Synlett* **2022**, *33* (12), 1154–1179. https://doi.org/10.1055/a-1390-9065.

(44) Wang, P.-Z.; Chen, J.-R.; Xiao, W.-J. Hantzsch Esters: An Emerging Versatile Class of Reagents in Photoredox Catalyzed Organic Synthesis. *Org. Biomol. Chem.* **2019**, *17* (29), 6936–6951. https://doi.org/10.1039/C9OB01289C.

(45) Nakagawa, M.; Matsuki, Y.; Nagao, K.; Ohmiya, H. A Triple Photoredox/Cobalt/Brønsted Acid Catalysis Enabling Markovnikov Hydroalkoxylation of Unactivated Alkenes. *J. Am. Chem. Soc.* **2022**, *144* (18), 7953–7959. https://doi.org/10.1021/jacs.2c00527.

(46) Roth, H.; Romero, N.; Nicewicz, D. Experimental and Calculated Electrochemical Potentials of Common Organic Molecules for Applications to Single-Electron Redox Chemistry. *Synlett* **2015**, *27* (05), 714–723. https://doi.org/10.1055/s-0035-1561297.
